# Morphological and Molecular Characterization of Human Dermal Lymphatic Collectors

**DOI:** 10.1371/journal.pone.0164964

**Published:** 2016-10-20

**Authors:** Viktoria Hasselhof, Anastasia Sperling, Kerstin Buttler, Philipp Ströbel, Jürgen Becker, Thiha Aung, Gunther Felmerer, Jörg Wilting

**Affiliations:** 1 Institute of Anatomy and Cell Biology, University Medical School Göttingen, Göttingen, Germany; 2 Institute of Pathology, University Medical Center Göttingen, Göttingen, Germany; 3 Division of Trauma Surgery, Plastic and Reconstructive Surgery, University Medical Center Göttingen, Göttingen, Germany; 4 Center of Plastic, Hand and Reconstructive Surgery, University Medical Center Regensburg, Regensburg, Germany; Northwestern University, UNITED STATES

## Abstract

Millions of patients suffer from lymphedema worldwide. Supporting the contractility of lymphatic collectors is an attractive target for pharmacological therapy of lymphedema. However, lymphatics have mostly been studied in animals, while the cellular and molecular characteristics of human lymphatic collectors are largely unknown. We studied epifascial lymphatic collectors of the thigh, which were isolated for autologous transplantations. Our immunohistological studies identify additional markers for LECs (vimentin, CCBE1). We show and confirm differences between initial and collecting lymphatics concerning the markers ESAM1, D2-40 and LYVE-1. Our transmission electron microscopic studies reveal two types of smooth muscle cells (SMCs) in the media of the collectors with dark and light cytoplasm. We observed *vasa vasorum* in the media of the largest collectors, as well as interstitial Cajal-like cells, which are highly ramified cells with long processes, caveolae, and lacking a basal lamina. They are in close contact with SMCs, which possess multiple caveolae at the contact sites. Immunohistologically we identified such cells with antibodies against vimentin and PDGFRα, but not CD34 and cKIT. With Next Generation Sequencing we searched for highly expressed genes in the media of lymphatic collectors, and found therapeutic targets, suitable for acceleration of lymphatic contractility, such as neuropeptide Y receptors 1, and 5; tachykinin receptors 1, and 2; purinergic receptors P2RX1, and 6, P2RY12, 13, and 14; 5-hydroxytryptamine receptors HTR2B, and 3C; and adrenoceptors α_2A,B,C_. Our studies represent the first comprehensive characterization of human epifascial lymphatic collectors, as a prerequisite for diagnosis and therapy.

## Introduction

The lymphatic vascular system is composed of initial lymphatics (sinusoids, capillaries) pre-collectors, collectors, lymph nodes, and trunks [1]. Initial lymphatics take up interstitial fluid, chylomicrons, migrating cells and pathogens, and conduct them via afferent lymphatic collectors to lymph nodes [2, 3]. Efferent lymphatic collectors and trunks finally drain lymph into the jugulo-subclavian venous junction (venous angle). The flow of interstitial fluid into initial lymphatics is directed by delicate endothelial valves, equipped with anchoring filaments and specialized intercellular junctions [4, 5]. Within the lymphatics, the centripetal flow of lymph is achieved by autonomous contractility of the lymphatic system, even in the complete absence of external mechanical stimuli such as skeletal muscle contraction or breathing. This has been shown experimentally in chick embryos, where specialized lymph hearts fulfill these functions [6]. In the human, lymphatic collectors, which are spontaneously contractile and equipped with intraluminal, semilunar valves, induce lymph flow and direct it towards the venous angles. This can be elegantly demonstrated with vital dyes in the human, where the active pumping of dermal lymphatic collectors has been demonstrated with indocyanine green (ICG) injections and detection of lymph flow with an infrared camera [7]. In fact, spontaneous contractility of lymph collectors has been known for quite some time [8], but has never been demonstrated in the human as unequivocally as with the ICG technique [9].

Despite their great importance for the active transport of lymph, human lymphatic collectors have not been characterized well, at both morphological and molecular levels. The majority of studies were performed on animals such as dog, guinea pig, sheep, cow, rabbit, and rat [10–16]. A few studies were performed in men on the thoracic duct, which is the most central part of the lymphovascular system and may already possess intermediate characteristics between lymphatics and veins [17–20]. Like the lymphatic collectors, the thoracic duct possesses peristaltic contractility, which has been attributed to the existence of interstitial Cajal-like cells (ICLCs). ICLCs have mainly been characterized at transmission electron microscopic level as ramified cells with long slender processes [17]. Although these authors also performed immunohistological studies of the thoracic duct, characterization of ICLCs in lymphatic collectors is still missing.

Whereas initial lymphatics do not possess any mural cells, not even pericytes, lymphatic collectors have a tunica media (media) and a tunica externa (adventitia). The phasic, peristaltic contractions of lymphatic collectors are based on the structure and function of smooth muscle cells (SMCs) in the media [21]. The expression of endothelial nitric oxide synthase (eNOS) in LECs of collectors seems to be of importance for the execution of dilatation waves [22]. However, our knowledge of the drug targets for the activation of contraction waves in human lymphatic collectors is extremely poor.

‘Lymphedema is a significant global problem, and its incidence will increase with a population that is living longer. In addition, because of the interrelationship between the lymphatic system and adipose tissue, the obesity epidemic has led to a rapid rise in this complication’ [23]. Inducing and supporting the activity and function of lymphatic collectors in arms and legs appears to be an attractive therapeutic option to treat lymphedema. As a prerequisite, the collectors must be characterized at molecular and morphological levels. Here, we started to characterize human lymphatic collectors isolated from the dermis of the thigh. These completely normal collectors were used for autologous transplantations in patients suffering from secondary lymphedema of the arm after breast cancer treatment. We used LEC markers and compared collectors and initial lymphatics. With electron microscopy and immunohistology we characterized SMCs and interstitial Cajal-like cells (ICLCs) in the media, and also performed global expression analyses of collectors to identify new molecular targets. Our data show that peripheral lymphatic collectors are well-capillarized ‘organs’ with *vasa vasorum* and ICLCs extending into the media. We confirm heterogeneity between LECs of initial lymphatics and collectors, identify new markers for LECs, show expression of vimentin and PDGFRα in ICLCs, and indicate molecular targets in the media of lymphatic collectors for the control of contractility.

## Materials and Methods

### Patients

Autologous lymphatic collector transplantation (n = 22) was performed on breast cancer patients suffering from secondary lymphedema of the arm after standard treatment. A normal lymphatic collector was taken from the hypodermis of the thigh, transplanted into the axillary region and connected proximally and distally to lymphatic collectors as described previously [24]. The collectors of the thigh were identified by injections of 0.2–0.5 ml Patent Blue into the dermis of the first and second interdigital space. A sufficiently long section of a collector was isolated, removed and used for transplantation. The free ends of the thigh lymphatics were sealed. The operations were highly successful. Not a single patient developed lymphedema in the leg where the transplant was taken, and all of them showed significantly less lymphedema in the arm after the transplantation. The clinical data will be presented in a separate study. Small pieces of lymphatic collectors, which were left over after the transplantations, were used for the present study. However, some of the collectors were long enough to be divided into pieces, and could be used for different techniques. Therefore, the total number of experiments was: Immunofluorescence, n = 15; Transmission electron microscopy (TEM), n = 6; Next Generation Sequencing (NGS), n = 6. All patient studies were approved by the ethics committee of the University Medical Center Göttingen (application no. 11/4/05). The patients were informed by the surgeons and gave their written consent. For comparison of lymphatic markers, initial lymphatics were studied in young boys’ foreskin (n = 6), removed for personal reasons. Studies on foreskin were performed with the informed, written consent of the parents of the minors, and also approved by the ethics committee of the University Medical Center Göttingen (application no. 11/4/05).

### Immunohistology

For immunofluorescence studies, specimens were fixed for 20–25 min in 4% paraformaldehyde (PFA), rinsed in PBS, transferred into 10% and 30% sucrose in PBS, and embedded in tissue freeze medium (Tissue Tek, Sakura Finetek Zoeterwoude, NL). Sections of 12–14μm were incubated with the following primary antibodies: Rabbit-anti-human CCBE1 (1:500, Sigma-Aldrich, München, Germany), rabbit-anti-human ESAM-1 (1:100, Sigma-Aldrich, München, Germany), goat-anti-human smooth muscle α-actin (SMA) (Acris Antibodies, Herford, Germany), mouse-anti-human CD31 (1:50, BD Pharmingen), mouse-anti-human CD117 (c-KIT, 1:100, Santa Cruz Biotechnol., and 1:1, Dako, Hamburg, Germany), mouse-anti-human D2-40 (1:200, Dako, Hamburg, Germany), rabbit-anti-human Lyve-1 (1:500, ReliaTech, Braunschweig, Germany), rabbit-anti-human Prox1 (1:500, ReliaTech, Braunschweig, Germany), mouse-anti-human vimentin (1:200, Dako), mouse-anti-human PDGFRα (1:1, Dako), mouse-anti-human CD34 (1:200, Dako). Secondary antibodies were: goat-anti-mouse Alexa 488/594, goat-anti-rabbit Alexa 594, donkey-anti-goat Alexa 488 (MobiTech, Göttingen, Germany). Sections were counter-stained with Dapi and mounted under cover slips with Fluoromount-G (Southern Biotechnology, US). Photos were taken with AxioImagerZ1 (Zeiss, Göttingen, Germany).

### Transmission electron microscopy (TEM)

Specimens were fixed with original Karnovsky’s fixative over-night [25], washed in 0.15 M phosphate buffer for 10 min, transferred into osmium tetroxide solution and incubated for 2 h at 4°C. Then the samples were rinsed with 0.15M phosphate buffer for 10 min and subsequently dehydrated in an ascending series of ethanol. Then the samples were transferred into Epon embedding solution and incubated for 24 h at 60°C. The embedded tissue was cut with an Ultracut E microtome (Reichert-Jung) to 750 nm (semi-thin) and 90 nm (ultra-thin) sections. Semi-thin sections were stained with Richardson’s solution and studied with light microscope. Ultrathin sections were transferred onto formvar-coated grids. After air-drying, samples were incubated 10 min in 1% uranyl acetate solution, 10 min in lead citrate [26] and rinsed with purified water. Specimens were analyzed with a Leo 906E (Zeiss) transmission electron microscope.

### Cell culture

Lymphatic endothelial cells (LECs) were isolated from lymphatic malformations of two young children as described previously. The cells possess molecular characteristics of initial lymphatics [27]. Purity of the culture was close to 100% as determined by anti-CD31 and anti-Prox1 double staining. As a third probe, we used juvenile foreskin-derived HDLECs (PromoCell, Heidelberg, Germany), controlled for Prox1 and CD31 expression. For blood endothelial cells (BECs), we chose a mixture of three juvenile foreskin-derived BEC lines (HDBECs; PromoCell). The cells were CD31-positive and at least 90% Prox1-negative.

### Preparation of RNA

Total RNA was isolated from LECs and BECs using peqGOLD Tri-Fast (PeqLab, Erlangen, Germany) according to the manufacturer’s instructions. Lymphatic collector samples of six female donors (age between 44 and 61 years) were isolated from surrounding connective tissue and snap-frozen in liquid nitrogen. The tissue was finely ground in liquid nitrogen using pestle and mortar, and immediately taken up in Tri-Fast reagent (PeqLab). Subsequently total RNA was prepared according to the manufacturer’s instructions.

### Next Generation Sequencing (NGS)

RNA quality was assessed by measuring the RIN (RNA Integrity Number) using an Agilent 2100 Bioanalyzer (Agilent Technologies, Palo Alto, CA). Library preparation for RNA-Seq was performed using the TruSeq™ RNA Sample Prep Kit v2 (Illumina, Cat. N°RS-122-2002) starting from 1000 ng of total RNA. Accurate quantification of cDNA libraries was performed with the QuantiFluor™ dsDNA System (Promega). The size range of final cDNA libraries was determined applying the DNA 1000 chip on the Bioanalyzer 2100 from Agilent (average 350 bp). cDNA libraries were amplified and sequenced by use of cBot and HiSeq2000 from Illumina (SR; 1x50 bp; ~25–40 million reads per sample). Sequence images were transformed with Illumina software BaseCaller to bcl files, which were de-multiplexed to fastq files with CASAVA v1.8.2.

### Statistical analysis

Quality check was done with fastqc (v. 0.10.0, Babraham Bioinformatics). Read alignment was performed using STAR v2.3.0 to the hg19 reference genome. Data were converted and sorted by samtools 0.1.19 and reads per gene were counted with htseq version 0.5.4.p3. Data analysis was performed using R/Bioconductor (3.0.2/2.12) loading DESeq, gplots and goseq packages. Candidate genes were filtered to a minimum of 1x fold change and FDR-corrected p-value <0.05. For functional analysis gene ontology enrichment was tested accounting for gene length via R-package goseq. Sequence data were deposited in NCBI's Gene Expression Omnibus (GEO) and are accessible through GEO Series accession number GSE87360. A subset of data showing lymphatic collector-expressed genes are presented as S1 and S2 Tables.

## Results

**Fig 1:**
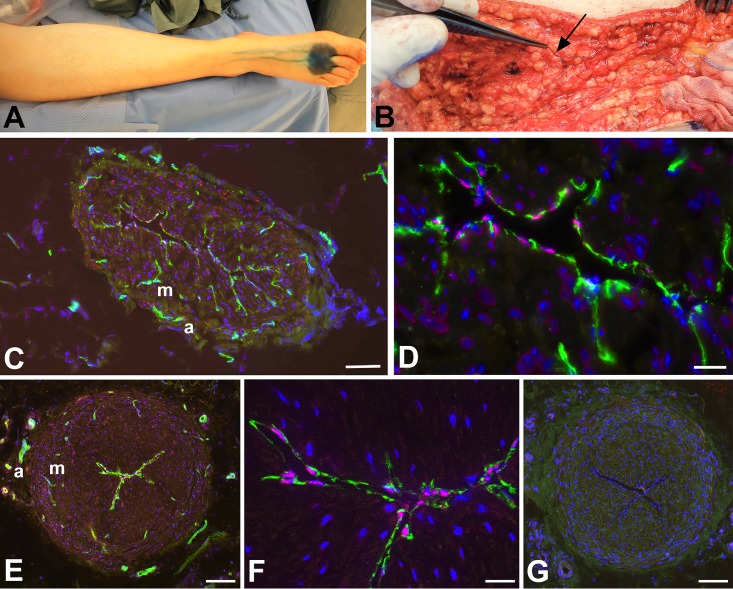
Detection of epifascial lymphatic collectors in patients. **A)** Intradermal injection of Patent Blue into the first two interdigital spaces of a patient’s foot. Note the centripetal flow of the marker in the lymphatics. **B)** Isolation of a lymphatic collector, which is marked blue (arrow), in the hypodermis of the thigh. **C-G**) Staining with antibodies against CD31 (green) and Prox1 (red) (**C-F**), and negative control with only secondary antibodies (**G**). Lymphatic endothelial cell express both of the markers (as seen at higher magnification in D, F). *Vasa vasorum* in the adventitia (a) and media (m) express only CD31. Bars = 150 μm in C; 25 μm in D; 15 μm in F, and 200 μm in E,G.

For the treatment of secondary arm lymphedema in breast cancer patients, autologous transplantation of functional lymphatic collectors was performed. The transplants were taken from the thigh. In the hypodermis of the thigh, there are numerous lymphatic collectors so that a single collector can be removed without the risk of provoking leg lymphedema. The collectors of the thigh were identified surgically after intradermal injection of Patent Blue into the first interdigital spaces (Fig 1A). The epifascial collectors of the thigh could be identified as delicate blue vessels, which were isolated and transplanted (Fig 1B). Spontaneous contractility of the collectors was observed intra-operatively. The collectors were transplanted into the axillary region, and lympho-lymphatic anastomoses were performed to reconstitute lymph flow of the edematous arm. Thereby, the length of the transplant must slightly exceed the gap that has to be bridged in the axilla. The remaining pieces of the collectors were used for histological and molecular studies.

**Fig 2:**
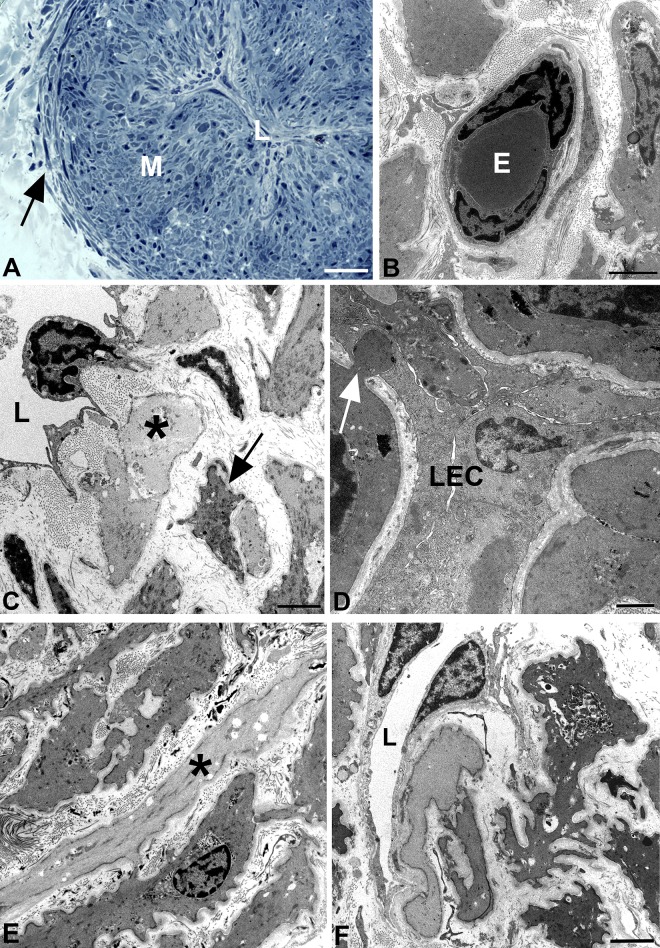
Semi- and ultra-thin sections of human epifascial lymphatic collectors. **A)** Semithin cross-section showing the collapsed lumen (L) of the collector. The media (M) is densely packed with smooth muscle cells (SMCs). Note the thin circular layer of SMCs (arrow) at the border to the adventitia. **B-F**) Ultrathin sections. **B**) TEM picture showing a capillary in the media of a collector, consisting of endothelial cells and pericytes. E, erythrocyte. **C**) TEM picture showing the lumen (L) of a lymphatic collector. The lymphatic endothelial cell possesses slender processes directed towards the SMCs in the media. Note the existence of dark (arrow) and light (asterisk) SMCs. **D**) TEM picture of the collector shown in A). The collapsed lumen is lined by lymphatic endothelial cells (LEC). Note the rivet-like junction (arrow) between a SMC and a LEC. **E)** TEM picture of the media showing SMCs. The majority of the SMCs has a dark cytoplasm, some are light (asterisk). **F**) TEM picture of a dark SMC with ‘starfish morphology’. The lumen (L) of the collector is lined by LECs. Bars = 150 μm in A, 2μm in B,C,E,F, and 1μm in D.

In a first step, the lymphatic nature of the collectors was verified by immunostaining with antibodies against CD31 and PROX1 [28]. The CD31 and PROX1 double-positive endothelial staining unequivocally proved the correctness of the clinical characterization of the collectors. Although the collectors varied in diameter between 1.3–1.8 mm, the LECs lining their lumen were always positive for the two markers (Fig 1C–1G). *Vasa vasorum* of lymphatic collectors have previously been found in the adventitia [29], however, our data clearly show that CD31+/PROX1- blood vessels penetrate the media of collectors that exceed a certain diameter (Fig 1E). These nutritive vessels were also found in semi- and ultra-thin sections. The studies showed that the media of the largest collectors (1.8 mm diameter) consisted of two layers of smooth muscle cells (SMCs), a thick inner layer with predominantly longitudinal orientation, and a thin outer layer with predominantly circular orientation of SMCs (Fig 2A). *Vasa vasorum* in the media were made up of capillaries with pericytes invested by a common basal lamina (Fig 2B). Occasionally a vascular SMC was found in the wall of the nutritive vessels, indicative of a metarteriole (data not shown). The inner lining of the collectors consisted of delicate LECs resting on a basal lamina. A layer of subendothelial connective tissue, which is typical for larger blood vessels, was usually not present. Rather, the LECs were in close proximity to SMCs (Fig 2C). We regularly observed fine processes of SMCs that penetrated the basal lamina of both the SMCs and the LECs and established rivet-like contacts between the two cell types (Fig 2D). At TEM level, we were able to distinguish between two types of SMCs–with dark and light cytoplasm (Fig 2C and 2E). Both types possessed typical features of SMCs such as basal lamina, caveolae, dense bodies and a central nucleus with loose cytoplasm at the nuclear poles. Many SMCs had numerous fine processes, which culminated in a ‘starfish-morphology’ of some of the cells (Fig 2F).

**Fig 3:**
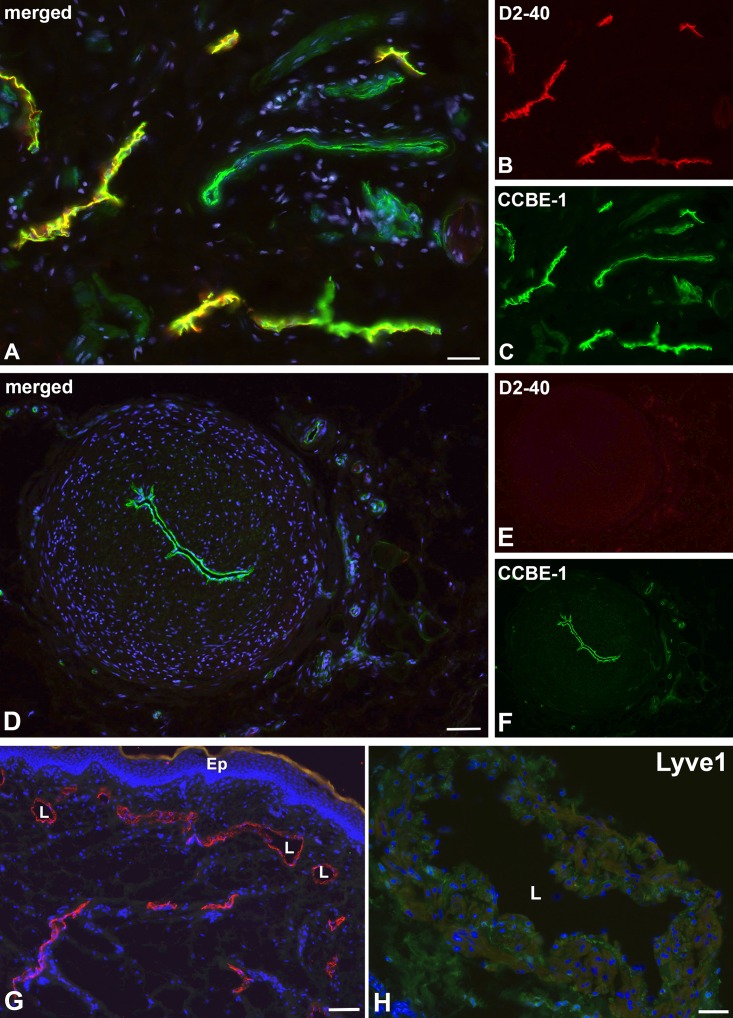
Immunofluorescence studies of initial foreskin lymphatics and epifascial lymphatic collectors. **A-C**) Initial lymphatics. Staining with the antibodies D2-40 (**A, B**) and CCBE1 (**A, C**). D2-40 marks LECs, while CCBE1 demarcates both LECs and blood vascular endothelium. Bar = 50μm. **D-F**) Lymphatic collector. Staining with the antibodies D2-40 (**D, E**) and CCBE1 (**D, F**). D2-40 is not expressed, while CCBE1 demarcates the LECs of collectors. Bar = 200μm. **G**) LYVE-1 (red) marks initial lymphatics in foreskin. L, lumen of lymphatics; Ep, epidermis. Bar = 75μm. **H**) No LYVE-1 signal is detectable in lymphatic collectors. L, lumen of the collector. Dapi (blue) marks all nuclei. Bar = 150μm.

**Fig 4:**
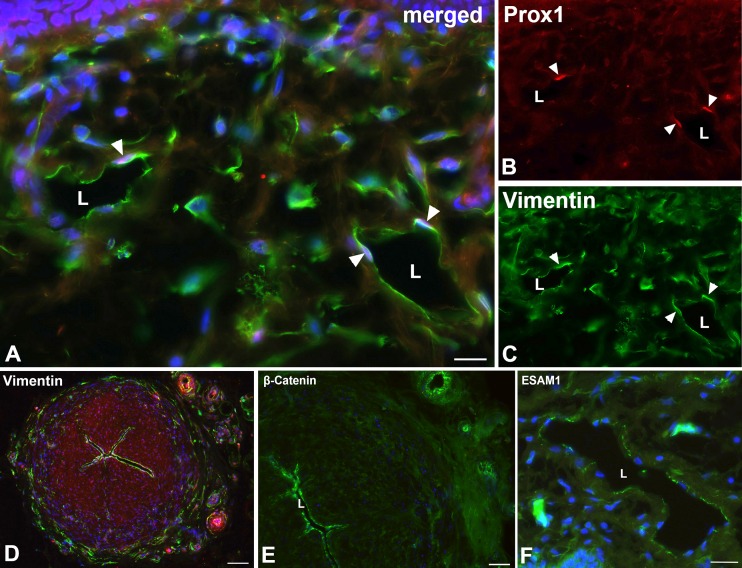
Immunofluorescence studies of initial lymphatics and epifascial lymphatic collectors. **A-C)** Staining of initial lymphatics with the antibodies Prox-1 (**A, B**) and vimentin (**A, C**). Vimentin is expressed in initial lymphatics. L, lumen of the lymphatics. Arrowheads point to the nuclei of LECs. Bar = 25μm. **D)** Staining of a lymphatic collector with antibodies against vimentin (green). Note expression in LECs, in numerous cells of the adventitia and some scattered cells in the media. Bar = 200μm. **E**) Focal expression of β-catenin in lymphatic collectors (L) and in larger vessels of the adventitia. Bar = 100μm. **F**) Focal expression of ESAM-1 in initial lymphatics (L) and strong expression in dermal capillaries. Dapi (blue) marks all nuclei. Bar = 35μm.

For further characterization of the lymphatic collectors, we used markers that are highly characteristic for LECs of initial lymphatics. We found expression of D2-40 in initial lymphatics of foreskin (Fig 3A and 3B), but not in collectors (Fig 3D and 3E). The extra-cellular matrix protein CCBE1 (Collagen and Calcium-binding EGF domains 1) demarcated both the LECs of initial lymphatics (Fig 3A and 3C) and collectors (Fig 3D and 3F). LYVE-1, a typical marker of LECs in initial lymphatics (Fig 3G), was not found in collectors (Fig 3H). The intermediate filament vimentin turned out to be a constant marker of LECs in both initial and collecting lymphatics. It was additionally expressed in cells of the adventitia, and to a much lesser extent in the media (Fig 4A–4D). Furthermore, we found focal expression of β-catenin in LECs of collectors (Fig 4E), but hardly any in initial lymphatics. In contrast, focal ESAM-1-positive staining was found in initial lymphatics, but not in collectors (Fig 4F).

**Fig 5:**
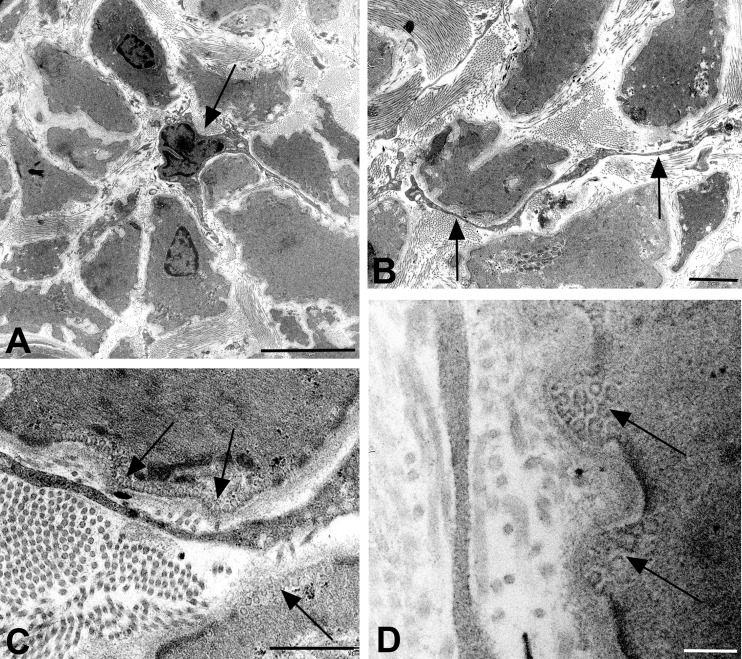
TEM studies of ICLCs in the media of lymphatic collectors. **A**) ICLC (arrow) with slender cell body and several cytoplasmic processes surrounded by SMCs. Bar = 5μm. **B**) Long process of an ICLC (arrows) in close contact to SMCs. Bar = 2μm. **C**) Higher magnification of B), showing accumulations of caveolae (arrows) in SMCs adjacent to the ICLC. Bar = 0.8μm. **D**) Peg-like protrusions of SMCs with caveolae (arrows) separated by dense plaques underlying the plasma membrane, adjacent to an ICLC process. Bar = 0.2μm.

**Fig 6:**
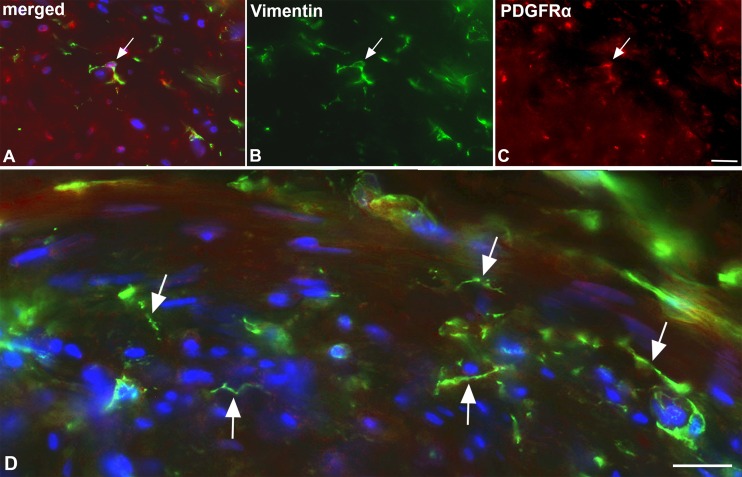
Immunofluorescence studies of ICLC in lymphatic collectors. Staining with antibodies against vimentin (green) (**A, B, D**) and PDGFRα (red) (**A, C**). Note double-positive ramified ICLC (arrow in A-C) in the media of the collector. Bar = 50μm. **D**) Higher magnification showing vimentin-positive cells with long processes (arrows), which possess varicose-like swellings. Dapi (blue) marks all nuclei. Bar = 35μm.

**Fig 7:**
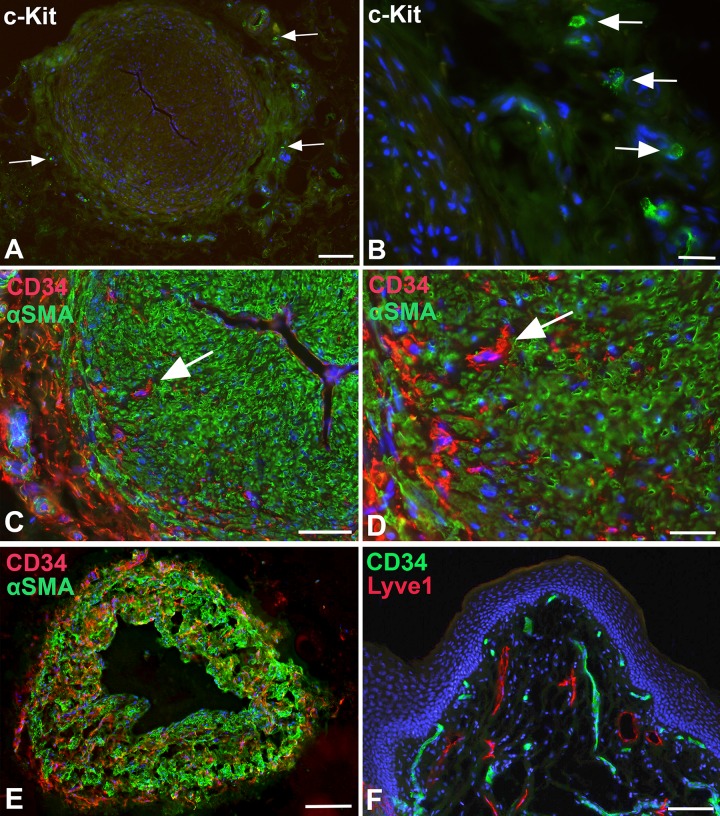
Immunofluorescence studies of lymphatic collectors and dermis. **A, B**) Staining with anti-cKIT (CD117) antibodies of lymphatic collectors. Granulated, round cells that express c-KIT (arrows) are located in the adventitia of the collectors. Bar = 250μm in A, and 25μm in B. **C-E**) Staining of a lymphatic collectors with anti-CD34 (red) and anti-αSMA (green) antibodies. **C,D**) In large collectors, CD34+ cells (arrow) are found in the adventitia, and in the outer parts of the media between the αSMA-positive SMCs. Bar = 150μm. **D**) Higher magnification of C showing nucleated CD34+ cells (arrow). No double-positive cells are visible. Bar = 60μm. **E**) Smaller caliber collector stained with anti-CD34 (red) and anti-αSMA (green) antibodies. CD34+ cells are found in all parts of the media. Bar = 200μm. **F**) Staining of foreskin with anti-CD34 (green) and anti-Lyve-1 (red) antibodies. Blood capillaries beneath the epidermis express CD34. The Lyve-1-positive lymphatics are CD34-negative. Dapi (blue) marks all nuclei. Bar = 150μm.

The autonomous peristaltic contractility of lymphatic collectors is very well established, however, the pace-maker cells that initiate SMC contractions have not been identified unequivocally [21]. A cell, which may be responsible for this activity, is the ICLC. In the thoracic duct, ICLCs have been demonstrated in the outer SMC layer of the media [17]. We used TEM as well as immunohistology for markers that have been described as highly specific for ICLCs such as vimentin, c-Kit and CD34, as well as PDGFRα, a marker for gastro-intestinal stroma tumor (GIST), which originates from ICLCs [30]. In TEM studies, we observed ramified cells with slender perinuclear cell body, and long cytoplasmic processes with varying calibers intermingled with and in close contact to the SMCs of the media (Fig 5A and 5B). The cells did not possess a continuous basal lamina, if any, and were often located immediately adjacent to SMCs, which had a dense facing with caveolae at the contact sites (Fig 5C and 5D). The ramified cells otherwise possessed characteristics that have previously been described for ICLCs in the thoracic duct, e.g. indented nucleus, and a regular but low amount of caveolae. Our immunofluorescence studies revealed a cell type with comparable morphology. Cells with long slender processes and varicose-like swellings were found in the media, preferentially in peripheral parts, with antibodies against vimentin and PDGFRα (Fig 6A–6D). Thereby, the double-staining against vimentin and PDGFRα appears to be the most reliable marker for ICLCs (Fig 6A–6C). The anti-c-KIT (CD117) staining (with antibodies from two companies) did not depict any ramified cells in the media, but round, granulated cells in the adventitia, which most likely represent mast cells (Fig 7A and 7B). The staining pattern with anti-CD34 is highly complex. In the dermis, the blood capillaries right beneath the epidermis were CD34-positive, while the initial lymphatics were negative (Fig 7F). In deeper layers of the dermis, the BECs lost this clear signal whereas multiple cells of the loose connective tissue were positive (data not shown). In lymphatic collectors, fibrocytes of the adventitia were positive, and a meshwork of CD34+ cells, most likely representing fibrocytes, was seen penetrating the outer third of the media of the collectors obviously along *vasa vasorum* (Fig 7C and 7D). We did not observe overlap of the CD34 and the αSMA signals. The two signals were mutually exclusive. In smaller caliber collectors, the CD34-positive fibrocytes were also seen in deeper parts of the media, indicating a larger contribution of fibrocytes to the media of smaller collectors (Fig 7E).

The essential driving force for lymph flow is the peristaltic contractility of SMCs in the media of the collectors, which represents an attractive target for pharmacologic therapy of lymphedema. For a global molecular characterization, we used next generation sequencing and compared lymphatic collectors with i) LECs derived from initial lymphatics, and ii) BECs derived from dermal microvessels. The adventitia of the collectors was removed. Since the majority of cells in the collectors are SMCs, it could be expected that the collector-expressed genes found simultaneously in both of the lists were SMC genes. We set the threshold for the log2 fold change (Log2FC) at 2, and found 2518 candidate genes in collectors as compared to LECs (S1 Table), and 2151 candidate genes as compared to BECs (S2 Table).

The assumption that the majority the collector-expressed genes, which appeared in both of the lists, were SMC genes, was supported by the very high expression of: actin, both the smooth muscle-type (ACTA2) and the enteric-type (ACTG2), as well as desmin (DES), the myokine decorin (DCN) [31], and its closely related lumican (LUM), myosin heavy chain 11 (MYH11), the typical myosin of SMCs [32], phospholamban (PLN), which controls cellular calcium levels [33], ryanodine receptor 2 (RYR2), a sarcoplasmatic calcium release channel mainly described in cardiomyocytes [34], sarcoglycan-alpha (SGCA), a member of the dystrophin-glycoprotein-complex with functions for vascular stability [35], the endothelin receptor-A (EDNRA) [36], the calcium ion reservoir protein calsequestrin-2 (CASQ2) [37], numerous Ca-channel proteins such as calcium channel, voltage-dependent, T-type α1H (CACNA1H) [38], calponin 1 (CNN1), which tonically inhibits ATPase activity of myosin in SMCs [39], CRISPLD2, a glucocorticoid responsive gene that modulates cytokine function in airway SMCs [40], dermatopontin (DPT), an extracellular matrix protein, which is significantly decreased in uterine leiomyoma [41], several EPH receptors, which are involved in vascular differentiation and identity, the ECM protein fibulin-1 (FBLN1), which is important for vascular stability [42], the microRNA143 (mir143), which regulates the proliferative phenotype of SMCs [43], thrombospondin 4 (THBS4), which is associated with premature coronary heart disease [44], synaptopodin 2 (SYNPO2), an actin-associated protein [45], as well as superoxide dismutase 3 (SOD3), an extracellular antioxidant enzyme often associated with the surface of endothelial cells [46].

Improving the contractility of lymphatic collectors is a promising therapeutic option for the treatment of lymphedema, which may be achieved by e.g. activation of the sympathetic, serotonergic, purinergic and neuropeptidergic systems. In superficial human thigh lymphatic collectors, we detected high expression of adrenoceptors α_2A,B,C_ (ADRA2A, ADRA2B, ADRA2C), 5-hydroxytryptamine (serotonin) receptors HTR2B, and 3C; purinergic receptors (P2RX1, P2RY12, 13, 14), neuropeptide Y receptors 1 and 5 (NPY1R, NPY5R), tachykinin receptors 1 and 2 (TACR1, TACR2), and vasoactive intestinal peptide receptor 2 (VIPR2) as well as pituitary adenylate cyclase-activating polypeptide type I receptor 1 (ADCYAP1R1).

## Discussion

### Heterogeneity of lymphatic endothelial cells

The lymphatic vascular system consists of initial lymphatics, pre-collectors, collectors, lymph nodes, and trunks. These sections of the lymphovascular system differ with respect to their morphology, function and molecular setting. Initial lymphatics take up interstitial fluid, collectors and trunks possess transport functions, and lymph nodes facilitate the intimate contact between lymph and immune cells. While initial lymphatics are characterized by inter-endothelial valves, which allow the influx of interstitial fluid [4], lymphatic collectors seem to be very tight [21]. These differences are reflected by different molecular settings [2]. We confirm the expression of the hyaluronan receptor Lyve-1 and the glycoprotein podoplanin—detected by the D2-40 antibody—in initial lymphatics. Additionally, we observed focal expression of the endothelial cell-selective adhesion molecule ESAM-1 in LECs of initial lymphatics, but not in collectors. The most reliable combination of markers for LECs are the antibodies against CD31 (PECAM-1) and the transcription factor PROX1, which demarcate LECs in initial lymphatics, collectors, and lymph nodes of children, adults and elderly people in health and disease [28, 47, 48]. The expression of CD31 and Prox1 proved the correctness of the clinical diagnosis for the collectors, which was made by the uptake and transport of Patent Blue injected intra-dermally into the first and second interdigital spaces of the foot. The expression of Prox1 clearly differentiates lymphatics from blood vessels, such as the *vasa vasorum*, which we observed for the first time in the media of the largest collectors.

Additionally, we identified new markers for LECs of both initial and collecting vessels. These are CCBE1 and vimentin. CCBE1 is a large ECM protein with multiple binding capacities for e.g. calcium, collagen and VEGF-C [49]. Mutations of the *CCBE1* gene cause a highly complex syndrome, the Hennekam syndrome. The affected children show multiple defects including mental retardation, facial dysplasia, hypoproteinemia, as well as generalized lymphedema [50]. The constant expression of CCBE1 along initial and collecting lymphatics argues for a permanent function of VEGF-C-binding proteins for the maintenance of lymphatic networks. The intermediate filament vimentin is typically expressed in so-called mesenchymal cells [51]. The term, however, is poorly defined, since during development almost all cell types undergo mesenchymal as well as epithelial phases, and differentiated endothelial cells are definitely epithelial cells. In pathological diagnosis, vimentin is used as a marker for tumors of soft tissue origin. A secreted form of vimentin is detected by the PAL-E antibody in venous endothelial cells [52]. Here we show that vimentin is a reliable marker for LECs, which should be taken into account in pathological diagnoses.

In LECs of lymphatic collectors, we also identified expression of the WNT-signaling mediator β-catenin. WNT-signaling has rarely been studied in lymphatics (for review see: [53]). We have recently observed a function for WNT5A during normal morphogenesis of dermal lymphatics [54]. Regulation of planar cell polarity by WNTs has been shown to be a morphogenetic component during valve formation in lymphatic collectors [55].

### Autonomous contractility of lymphatic collectors

Autonomous, temperature-dependent contractions of lymphatic collectors have been observed quite some time ago, first in animals [56] and later in man [8]. We observed contractions during the isolation and transplantation of epifascial thigh collectors. Pumping of collectors can be visualized live in patients with infra-red cameras after injection of the tracer ICG [7]. The initiation of collector contractions is only incompletely understood. In sheep, potential pace-maker cells have been found in subendothelial positions, and could be stained with antibodies against vimentin and c-kit [14]. Due to their resemblance with the interstitial cells of Cajal in the gastro-intestinal tract, the cells were called interstitial Cajal-like cells (ICLC). They have been found in the human thoracic duct and characterized at TEM level [17]. The ICLCs which we found in the media of human thigh collectors meet all the criteria established for this cell type: ramified cells with long slender processes variable in diameter, caveolae, absent basal lamina and close contact to neighboring cells. We have found such ICLCs in intimate contact with SMCs. Of note, the SMCs possess numerous caveolae at the contact sites. Caveolae have several functions such as endocytosis, mechanosensing and cell signaling via NO and calcium [57]. Together with the high expression of phospholamban, which controls cellular calcium levels [33], and the sarcoplasmatic calcium release channel ryanodine receptor 2, which we found with NGS in collectors, this points to a regulation of collector contractility via calcium signaling.

In appr. 5% of cells derived from sheep lymphatic collectors, McCloskey and coworkers (1999) detected a hyperpolarization-activated inward current (so-called funny current), indicative of pace-maker cells (ICLCs) [14]. As noted above, immunohistological studies indicated vimentin and c-kit expression in such cells [58]. Our studies suggest the existence of species differences as concerns the molecular equipment of ICLCs. We observed co-expression of vimentin and PDGFRα in ramified cells in the media of human collectors, but not c-KIT, which we studied with two different antibodies. In accordance with other studies, we found c-KIT expression in granulated cells, both in the dermis and in the adventitia of collectors. The cells most likely representing mast cells [59]. CD34 clearly demarcates fibrocytes in the adventitia, which is in line with previous studies [28], and the use of CD34 as a marker for the diagnostics of fibrous tumors [60, 61]. A continuation of this signal is found in the media. In large caliber collectors CD34+ cells are mainly located in the peripheral parts of the media. In smaller collectors such cells are found throughout the media. There, the number of CD34-positive cell was obviously to large as to represent ICLCs. Most likely, CD34 depicts fibroblast in the wall of the collectors.

Interestingly, the expression of PDGFRα in human ICLCs is in line with the observation that mutations in the *PDGFR*α gene cause gastro-intestinal stroma tumor (GIST), a tumor originating from interstitial cells of Cajal in the gastro-intestinal tract [30]. However, the fact that c-KIT mutations may also cause GIST indicates heterogeneity of interstitial cells of Cajal and ICLCs. Co-expression of vimentin and c-KIT has been found in cells of the human thoracic duct [17]. Using the same antibodies, we could not detect c-KIT in human lymphatic collectors. Again, this may indicate heterogeneity, and may also provide an explanation for the observation of mall numbers of c-KIT-negative GIST [62].

### Therapeutic targets in lymphatic collectors

Activation of SMCs in lymphatic collectors represents a promising target for pharmacological treatment of lymphedema. The SMCs of lymphatic collectors represent a functional syncytium [63]. Interestingly, we observed heterogeneity of SMCs at TEM level. The majority of SMCs possess a dark cytoplasm, but some are light. It is tempting to speculate that the two types of SMCs fulfill different functions, and, like in the heart, the light ones may have primarily conductive rather than contractile functions.

A global characterization of human lymphatic collectors has not been performed yet. This knowledge, however, is the prerequisite for targeted therapeutic interventions to improve e.g. inflammatory diseases (lymphangitis) or contractile functions of collectors. The latter may be applicable to millions of patients worldwide suffering from secondary lymphedema. We therefore applied NGS and compared isolated thigh collectors with cultures of isolated LECs and BECs. We found 2518 collector-expressed genes in comparison to LECs, and 2151 collector-expressed genes in comparison to BECs. The overlapping genes in both lists mainly seem to represent SMC-expressed genes, as confirmed by the high expression of typical SMC genes highlighted in the S1 and S2 Tables.

However, although there is evidence that numerous of the genes listed in the S1 and S2 Tables are expressed in SMCs, we must be aware of the fact, that the wall of the collectors is made up of various cell types including SMCs, ICLCs, fibrocytes, adipocytes, mast cells, LECs, BECs, and others, and additionally there is heterogeneity within some of the cell types, e.g. we observed dark and light SMCs. The comparison with cultured LECs and BECs (which possess a molecular equipment that is not identical to their counterparts in the collectors) will therefore not produce a list of genes that can be ascribed to one specific cell type. We are nevertheless convinced that our study is a starting point to look for specific drug targets in lymphatic collectors. Some of the potential therapeutic targets that are discussed below may not be expressed in SMCs, but in other cell types. This assumption is supported e.g. by the high expression of DOG1 (= ANO1, anoctamin-1), a marker used for the diagnosis of GIST [62, 64]. This indicates that a number of target genes may be expressed in ICLCs of the collectors, which, in fact, may even be an advantage for the treatment of lymphedema.

For the treatment of lymphedema adrenergic and peptidergic receptors appear to be of greatest interest. In animals, noradrenergic, purinergic, cholinergic and peptidergic nerves have been found along lymphatic collectors [21], and the activation of the sympathetic chain in animals increases the frequency of spontaneous contractions [65]. Adrenergic innervation has also been found in the human thoracic duct [66]. In collectors, we found expression of the α_2_ adrenoceptors ADRA2A, ADRA2B, and ADRA2C, which are coupled to G-proteins. Besides effects in the central nervous system, they control SMC functions such as contraction of spincters in the gastrointestinal (GI) tract, but also decrease of motility of SMCs in the GI tract, as well as constriction of certain arteries and veins [67–69].

The 5-hydroxytryptamine (serotonin) receptors HTR2B and HTR3C, which we found in the collectors, appear to be of special interest. Serotonin is a potent inducer of contraction of blood vessels, and also causes psychological effects in the central nervous system. Most of the body’s serotonin is located in the enterochromaffin cells of the GI tract, where it promotes motility [70]. A similar effect on the contractility of lymphatic collectors could be beneficial.

Purinergic receptors exist with numerous subtypes (P0, P1 and P2), and are widely distributed in the organism. In lymphatic collectors, we detected the purinergic receptors P2RX1, P2RX6, P2RY12, P2RY13, and P2RY14. P2Y receptors are G-protein-coupled receptors (GPCRs) [71]. P2RY12 antagonist are in clinical use as inhibitors of platelet aggregation [72]. The seven P2X receptor subtypes form homo- and heteromeric ion canals [73].

Neuropeptide Y (NPY) is a 36-amino acid peptide, which exerts a number of divers effects such as control of psychomotor activity, food intake and cardiovascular activity [74, 75]. NPY receptors are GPCRs [76]. We identified NPY1R and NPY5R in human lymphatic collectors.

Vasoactive intestinal peptide (VIP) is a 28-amino acid peptide hormone, which stimulates contractility of the heart and relaxes SMCs of the aorta, trachea, gall bladder and gut [77]. We detected expression of VIP receptor 2 (VIPR2) and its related receptor ADCYAP1R1 (pituitary adenylate cyclase-activating polypeptide type I receptor 1)[78] in lymphatic collectors. Few VIP-positive nerve fibers have been detected in the vicinity of human lymphatics [18].

Furthermore, in the collectors, we identified tachykinin receptors 1 and 2 (TACR1, TACR2 = neurokinin receptors), which are GPCRs that bind substance P (SP). SP, an 11-amino acid peptide, was originally identified by its ability to cause intestinal contractions [79]. SP induces vasodilatation and thereby acts via NO release [80]. Relaxation by SP has been shown *ex vivo* in rat mesenteric lymphatics [81], and, in rats, SP-mediated crosstalk between pro-inflammatory and contractile signaling has been observed [82]. And among others, we identified expression of bradykinin receptors B1 and B2 (BDKRB1, BDKRB2) in the collectors. Bradykinin is a 9-amino acid peptide. It is an endothelium-dependent vasodilator and inflammatory mediator. Antagonists of bradykinin have been tested in hederitary angioedema [83, 84].

The huge number of specific agonists and antagonists that exist for the adrenergic, serotonergic, purinergic and peptidergic receptors in combination with the possibility to visualize pumping of lymphatic collectors live in the dermis of patients with the ICG method opens up a new field of therapeutic approaches for lymphedema.

## Supporting Information

S1 Table List of 2518 genes at least 4-fold higher expressed in human epifascial thigh lymphatic collectors than in cultured human LECs. Genes are listed alphabetically by HGNC Symbol. Log2FC: Log2 fold change; FDR: false discovery rate. Highly expressed SMC genes, which appear in both of the lists and are discussed in the manuscript, are marked in green.
(PDF)

S2 Table List of 2151 genes at least 4-fold higher expressed in human epifascial thigh lymphatic collectors than in cultured human BECs. Genes are listed alphabetically by HGNC Symbol. Log2FC: Log2 fold change; FDR: false discovery rate. Highly expressed SMC genes, which appear in both of the lists and are discussed in the manuscript, are marked in green.
(PDF)
