# Supplementary material for: Morphological and Molecular Characterization of Human Dermal Lymphatic Collectors
Source: PLoS One. 2016 Oct 20;11(10):e0164964. doi: 10.1371/journal.pone.0164964 (PMC5072738; doi:10.1371/journal.pone.0164964)
Supplement: S1 Table — Genes are listed alphabetically by HGNC Symbol. Log2FC: Log2 fold change; FDR: false discovery rate. Highly expressed SMC genes, which appear in both of the lists and are discussed in the manuscript, are marked in green. (PDF) [file pone.0164964.s001.pdf]

HGNC Symbol	HGNC-I	Description	ENSEMBL-	Entrezg	Log2FC	FDR
A2M	7	alpha-2-macroglobulin |	ENSG0000C	2	2,18	4,15E-08
AADAC	17	arylacetamide deacetyla	ENSG0000C	13	2,55	3,70E-02
ABCA10	30	ATP-binding cassette, su	ENSG0000C	10349	5,64	2,47E-24
ABCA8	38	ATP-binding cassette, su	ENSG0000C	10351	7,36	5,16E-39
ABCA9	39	ATP-binding cassette, su	ENSG0000C	10350	4,47	6,15E-10
ABCB1	40	ATP-binding cassette, su	ENSG0000C	5243	2,67	5,71E-04
ABCB5	46	ATP-binding cassette, su	ENSG0000C	340273	5,21	2,58E-08
ABCC8	59	ATP-binding cassette, su	ENSG0000C	6833	6,17	1,18E-11
ABCC9	60	ATP-binding cassette, su	ENSG0000C	10060	9,24	3,94E-63
ABCD2	66	ATP-binding cassette, su	ENSG0000C	225	5,10	1,79E-08
ABCG2	74	ATP-binding cassette, su	ENSG0000C	9429	2,46	5,24E-04
ABHD1	17553	abhydrolase domain cor	ENSG0000C	84696	2,09	3,72E-05
ABRA	30655	actin-binding Rho activa	ENSG0000C	137735	6,59	6,83E-07
ACACB	85	acetyl-CoA carboxylase	ENSG0000C	32	4,55	1,50E-16
ACADL	88	acyl-CoA dehydrogenas	ENSG0000C	33	6,37	2,17E-22
ACAN	319	aggrecan [Source:HGN(	ENSG0000C	176	4,79	1,80E-05
ACAP1	16467	ArfGAP with coiled-coil,	ENSG0000C	9744	2,23	4,17E-06
ACCN2					3,48	1,99E-10
ACE2	13557	angiotensin I convertin	ENSG0000C	59272	2,57	7,94E-04
ACOX2	120	acyl-CoA oxidase 2, bra	ENSG0000C	8309	2,20	9,40E-06
ACP5	124	acid phosphatase 5, tar	ENSG0000C	54	5,84	4,59E-18
ACPL2	26303	acid phosphatase-like 2	ENSG0000C	92370	4,73	6,56E-28
ACR	126	acrosin [Source:HGNC S	ENSG0000C	49	3,00	8,43E-06
ACSL6	16496	acyl-CoA synthetase lor	ENSG0000C	23305	2,92	2,65E-04
ACSM1	18049	acyl-CoA synthetase me	ENSG0000C	116285	4,86	1,38E-10
ACSM5	26060	acyl-CoA synthetase me	ENSG0000C	54988	3,89	2,66E-11
ACSS3	24723	acyl-CoA synthetase sh	ENSG0000C	79611	7,81	1,68E-45
ACTA1	129	actin, alpha 1, skeletal	ENSG0000C	58	5,19	1,75E-06
ACTA2	130	actin, alpha 2, smooth r	ENSG0000C	59	8,21	3,89E-26
ACTC1	143	actin, alpha, cardiac mu	ENSG0000C	70	6,20	1,28E-11
ACTG2	145	actin, gamma 2, smootl	ENSG0000C	72	9,43	1,73E-28
ACTN2	164	actinin, alpha 2 [Source	ENSG0000C	88	5,35	8,43E-09
ACVR1C	18123	activin A receptor, type	ENSG0000C	130399	4,78	4,75E-04
ADAM11	189	ADAM metallopeptidase	ENSG0000C	4185	3,03	6,24E-09
ADAM22	201	ADAM metallopeptidase	ENSG0000C	53616	5,11	4,61E-45
ADAM28	206	ADAM metallopeptidase	ENSG0000C	10863	3,59	3,48E-06
ADAM32	15479	ADAM metallopeptidase	ENSG0000C	203102	2,34	1,68E-07
ADAM33	15478	ADAM metallopeptidase	ENSG0000C	80332	8,58	8,45E-51
ADAM8	215	ADAM metallopeptidase	ENSG0000C	101	3,04	2,79E-05
ADAMTS1	217	ADAM metallopeptidase	ENSG0000C	9510	5,18	5,79E-06
ADAMTS12	14605	ADAM metallopeptidase	ENSG0000C	81792	5,58	5,21E-32
ADAMTS15	16305	ADAM metallopeptidase	ENSG0000C	170689	3,26	4,98E-05
ADAMTS16	17108	ADAM metallopeptidase	ENSG0000C	170690	3,71	3,83E-04
ADAMTS17	17109	ADAM metallopeptidase	ENSG0000C	170691	4,68	3,82E-08
ADAMTS2	218	ADAM metallopeptidase	ENSG0000C	9509	6,62	2,38E-19
ADAMTS4	220	ADAM metallopeptidase	ENSG0000C	9507	2,66	4,46E-02
ADAMTS5	221	ADAM metallopeptidase	ENSG0000C	11096	5,16	1,78E-08
ADAMTS8	224	ADAM metallopeptidase	ENSG0000C	11095	2,82	3,79E-03
ADAMTS9-AS2	42435	ADAMTS9 antisense RN	ENSG0000C	#####	2,89	8,99E-10
ADAMTSL4	19706	ADAMTS-like 4 [Source	ENSG0000C	54507	3,01	4,17E-18
ADAMTSL5	27912	ADAMTS-like 5 [Source	ENSG0000C	339366	4,32	1,28E-07
ADAP2	16487	ArfGAP with dual PH do	ENSG0000C	55803	3,98	9,03E-10

ADCY1	232	adenylate cyclase 1 (bra	ENSG0000C	107	3,78	6,10E-09
ADCY2	233	adenylate cyclase 2 (bra	ENSG0000C	108	6,24	1,74E-25
ADCY5	236	adenylate cyclase 5 [So	ENSG0000C	111	6,58	1,73E-21
ADCY7	238	adenylate cyclase 7 [So	ENSG0000C	113	2,03	1,26E-05
ADCYAP1R1	242	adenylate cyclase activa	ENSG0000C	117	6,20	3,34E-14
ADH1A	249	alcohol dehydrogenase	ENSG0000C	124	4,25	2,32E-07
ADH1B	250	alcohol dehydrogenase	ENSG0000C	125	10,13	1,13E-36
ADH1C	251	alcohol dehydrogenase	ENSG0000C	126	6,58	2,05E-22
ADIPOQ	13633	adiponectin, C1Q and co	ENSG0000C	9370	9,50	2,83E-08
ADRA2A	281	adrenoceptor alpha 2A		ENSG0000C	150	5,50	4,36E-09
ADRA2B	282	adrenoceptor alpha 2B		ENSG0000C	151	6,33	3,10E-14
ADRA2C	283	adrenoceptor alpha 2C		ENSG0000C	152	7,62	2,97E-16
ADRBK2	290	adrenergic, beta, recept	ENSG0000C	157	4,33	6,58E-10
AEBP1	303	AE binding protein 1 [So	ENSG0000C	165	3,21	1,03E-07
AFAP1L2	25901	actin filament associate	ENSG0000C	84632	2,24	8,93E-04
AFF2	3776	AF4/FMR2 family, meml	ENSG0000C	2334	2,07	1,73E-02
AFF3	6473	AF4/FMR2 family, meml	ENSG0000C	3899	4,81	1,66E-12
AGAP11	29421	ankyrin repeat and GTP	ENSG00000151303		5,15	2,36E-22
AGT	333	angiotensinogen (serpir	ENSG0000C	183	7,66	5,17E-22
AGTR1	336	angiotensin II receptor,	ENSG0000C	185	9,06	8,14E-42
AIF1	352	allograft inflammatory f	ENSG0000C	199	7,74	2,64E-32
AK7	20091	adenylate kinase 7 [Sou	ENSG0000C	122481	2,43	5,66E-05
AKNA	24108	AT-hook transcription fa	ENSG0000C	80709	3,11	3,05E-16
AKNAD1	28398	AKNA domain containin	ENSG0000C	254268	2,67	6,10E-07
AKR1B10	382	aldo-keto reductase fan	ENSG0000C	57016	3,46	1,21E-03
AKR1C1	384	aldo-keto reductase fan	ENSG0000C	1645	3,38	6,43E-12
AKR1C2	385	aldo-keto reductase fan	ENSG0000C	1646	2,48	5,69E-06
ALDH1B1	407	aldehyde dehydrogenas	ENSG0000C	219	2,35	2,05E-07
ALDH1L1	3978	aldehyde dehydrogenas	ENSG0000C	10840	4,71	1,77E-05
ALDH1L2	26777	aldehyde dehydrogenas	ENSG0000C	160428	4,87	1,05E-29
ALDH3A1	405	aldehyde dehydrogenas	ENSG0000C	218	3,94	8,50E-10
ALDH8A1	15471	aldehyde dehydrogenas	ENSG0000C	64577	2,35	7,33E-05
ALOX5	435	arachidonate 5-lipoxyge	ENSG0000C	240	6,62	5,15E-21
AMICA1	19084	adhesion molecule, inte	ENSG0000C	120425	3,83	6,48E-07
AMIGO2	24073	adhesion molecule with	ENSG0000C	347902	3,49	2,99E-15
AMOT	17810	angiomotin [Source:HG	ENSG0000C	154796	2,87	2,21E-19
AMPH	471	amphiphysin [Source:H	ENSG0000C	273	6,25	2,62E-31
AMT	473	aminomethyltransferase	ENSG0000C	275	2,32	4,28E-14
AMY2B	478	amylase, alpha 2B (pan	ENSG0000C	280	2,97	2,16E-14
ANGPT1	484	angiopoietin 1 [Source:	ENSG0000C	284	5,44	1,25E-15
ANGPT4	487	angiopoietin 4 [Source:	ENSG0000C	51378	4,31	8,22E-06
ANGPTL1	489	angiopoietin-like 1 [Sou	ENSG0000C	9068	8,53	1,91E-65
ANGPTL2	490	angiopoietin-like 2 [Sou	ENSG0000C	23452	3,06	5,78E-16
ANGPTL5	19705	angiopoietin-like 5 [Sou	ENSG0000C	253935	7,57	2,99E-20
ANGPTL7	24078	angiopoietin-like 7 [Sou	ENSG0000C	10218	2,98	3,53E-03
ANK2	493	ankyrin 2, neuronal [So	ENSG0000C	287	3,37	2,97E-08
ANKDD1A	28002	ankyrin repeat and deat	ENSG0000C	348094	3,64	2,96E-23
ANKRD29	27110	ankyrin repeat domain :	ENSG0000C	147463	2,28	4,02E-09
ANKRD30BL	35167	ankyrin repeat domain :	ENSG0000C	554226	2,45	1,76E-02
ANKRD35	26323	ankyrin repeat domain :	ENSG0000C	148741	3,10	5,48E-06
ANKRD65	42950	ankyrin repeat domain (	ENSG0000C	441869	4,89	2,64E-21
ANKS1B	24600	ankyrin repeat and ster	ENSG0000C	56899	4,75	1,39E-17
ANO1	21625	anoctamin 1, calcium ac	ENSG0000C	55107	6,33	1,59E-19

ANO3	14004	anoctamin 3 [Source:H(	ENSG0000C	63982	3,86	1,50E-08
ANO5	27337	anoctamin 5 [Source:H(	ENSG0000C	203859	6,18	3,42E-24
ANTXR1	21014	anthrax toxin receptor 1	ENSG0000C	84168	3,97	3,76E-07
ANXA6	544	annexin A6 [Source:HG	ENSG0000C	309	3,16	1,15E-10
AOAH	548	acyloxyacyl hydrolase (I	ENSG0000C	313	4,92	9,68E-15
AOC3	550	amine oxidase, copper (	ENSG0000C	8639	8,06	6,44E-42
AOC4					5,37	2,63E-12
AOX1	553	aldehyde oxidase 1 [So	ENSG0000C	316	3,62	8,54E-05
APBB1IP	17379	amyloid beta (A4) precu	ENSG0000C	54518	6,34	3,26E-14
APCDD1	15718	adenomatosis polyposis	ENSG0000C	147495	8,14	4,22E-39
APCDD1L	26892	adenomatosis polyposis	ENSG0000C	164284	5,34	1,28E-09
APLNR	339	apelin receptor [Source	ENSG0000C	187	5,21	8,30E-07
APOA1	600	apolipoprotein A-I [Sou	ENSG0000C	335	3,80	7,22E-07
APOBR	24087	apolipoprotein B recept	ENSG0000C	55911	3,18	4,80E-04
APOC1	607	apolipoprotein C-I [Sou	ENSG0000C	341	3,47	7,88E-05
APOD	612	apolipoprotein D [Sourc	ENSG0000C	347	5,68	3,23E-15
APOE	613	apolipoprotein E [Sourc	ENSG0000C	348	3,75	1,55E-06
APOLD1	25268	apolipoprotein L domair	ENSG0000C	81575	3,91	3,94E-05
AQP1	633	aquaporin 1 (Colton blo	ENSG0000C	358	6,00	2,93E-17
AQP10	16029	aquaporin 10 [Source:H	ENSG0000C	89872	2,97	1,10E-04
AQP7	640	aquaporin 7 [Source:HC	ENSG0000C	364	6,12	3,69E-07
AQP9	643	aquaporin 9 [Source:HC	ENSG0000C	366	5,86	5,38E-05
AQPEP					4,52	4,39E-09
AR	644	androgen receptor [Sou	ENSG0000C	367	2,52	6,50E-06
ARAP2	16924	ArfGAP with RhoGAP do	ENSG0000C	116984	6,68	1,25E-11
ARC	648	activity-regulated cytos	ENSG0000C	23237	6,00	1,81E-08
ARHGAP15	21030	Rho GTPase activating p	ENSG0000C	55843	8,31	8,07E-65
ARHGAP20	18357	Rho GTPase activating p	ENSG0000C	57569	5,81	1,02E-21
ARHGAP30	27414	Rho GTPase activating p	ENSG0000C	257106	5,21	8,05E-19
ARHGAP44	29096	Rho GTPase activating p	ENSG0000C	9912	3,98	3,24E-28
ARHGAP6	676	Rho GTPase activating p	ENSG0000C	395	6,24	1,61E-45
ARHGAP9	14130	Rho GTPase activating p	ENSG0000C	64333	4,55	2,76E-11
ARHGEF10L	25540	Rho guanine nucleotide	ENSG0000C	55160	3,88	3,57E-16
ARHGEF25	30275	Rho guanine nucleotide	ENSG0000C	115557	5,27	2,07E-36
ARHGEF26	24490	Rho guanine nucleotide	ENSG0000C	26084	6,21	4,02E-25
ARHGEF4	684	Rho guanine nucleotide	ENSG0000C	50649	6,04	2,90E-16
ARHGEF5	13209	Rho guanine nucleotide	ENSG0000C	7984	4,05	7,80E-14
ARHGEF9	14561	Cdc42 guanine nucleoti	ENSG0000C	23229	2,44	1,31E-11
ARID5A	17361	AT rich interactive doma	ENSG0000C	10865	3,10	1,82E-05
ARID5B	17362	AT rich interactive doma	ENSG0000C	84159	2,71	7,46E-08
ARL4D	656	ADP-ribosylation factor-	ENSG0000C	379	2,44	6,46E-07
ARNT2	16876	aryl-hydrocarbon recept	ENSG0000C	9915	5,51	3,94E-19
ARSI	32521	arylsulfatase family, me	ENSG0000C	340075	2,01	2,28E-02
ART3	725	ADP-ribosyltransferase	ENSG0000C	419	4,53	3,31E-09
ASB2	16012	ankyrin repeat and SOC	ENSG0000C	51676	7,53	6,43E-28
ASB5	17180	ankyrin repeat and SOC	ENSG0000C	140458	4,61	1,32E-11
ASPA	756	aspartoacylase [Source	ENSG0000C	443	6,38	5,49E-29
ASPG	20123	asparaginase homolog (	ENSG0000C	374569	6,72	3,47E-16
ASPN	14872	asporin [Source:HGNC !	ENSG0000C	54829	9,20	6,29E-47
ASPRV1	26321	aspartic peptidase, retr	ENSG0000C	151516	3,19	1,32E-13
ASXL3	29357	additional sex combs lik	ENSG0000C	80816	3,78	4,27E-08
ATF3	785	activating transcription	ENSG0000C	467	6,33	2,19E-13
ATG9B	21899	autophagy related 9B [S	ENSG0000C	285973	2,51	1,14E-03

ATL1	11231	atlastin GTPase 1 [Sour	ENSG0000C	51062	3,47	5,18E-14
ATP10A	13542	ATPase, class V, type 1C	ENSG0000C	57194	7,00	5,58E-15
ATP13A4	25422	ATPase type 13A4 [Sour	ENSG0000C	84239	3,13	4,89E-04
ATP1A2	800	ATPase, Na+/K+ transp	ENSG0000C	477	9,29	4,46E-41
ATP1B2	805	ATPase, Na+/K+ transp	ENSG0000C	482	6,37	3,24E-25
ATP2A3	813	ATPase, Ca++ transport	ENSG0000C	489	4,89	1,80E-09
ATP6V1G2	862	ATPase, H+ transporting	ENSG0000C	534	2,25	8,17E-04
ATP8B4	13536	ATPase, class I, type 8B	ENSG0000C	79895	5,18	6,21E-23
ATRNL1	29063	attractin-like 1 [Source:	ENSG0000C	26033	5,30	1,12E-13
AUTS2	14262	autism susceptibility ca	ENSG0000C	26053	5,16	1,62E-22
AVIL	14188	advillin [Source:HGNC S	ENSG0000C	10677	2,28	1,17E-04
AVPR1A	895	arginine vasopressin rec	ENSG0000C	552	6,63	1,93E-14
AVPR2	897	arginine vasopressin rec	ENSG0000C	554	4,91	4,87E-14
AXIN2	904	axin 2 [Source:HGNC S	ENSG0000C	8313	2,22	4,80E-08
AXL	905	AXL receptor tyrosine k	ENSG0000C	558	2,57	1,32E-14
AZGP1	910	alpha-2-glycoprotein 1,	ENSG0000C	563	5,76	1,12E-06
AZU1	913	azurocidin 1 [Source:HC	ENSG0000C	566	4,51	2,93E-03
B3GAT2	922	beta-1,3-glucuronyltran	ENSG0000C	135152	4,63	1,46E-09
B4GALNT1	4117	beta-1,4-N-acetyl-galac	ENSG0000C	2583	2,78	4,14E-06
BAALC	14333	brain and acute leukem	ENSG0000C	79870	4,17	5,19E-05
BAG2	938	BCL2-associated athanc	ENSG0000C	9532	3,91	1,25E-12
BAI1	943	brain-specific angiogene	ENSG0000C	575	4,81	1,06E-05
BAI3	945	brain-specific angiogene	ENSG0000C	577	4,73	2,47E-14
BAIAP3	948	BAI1-associated protein	ENSG0000C	8938	2,31	6,95E-06
BASP1	957	brain abundant, membr	ENSG0000C	10409	4,72	1,05E-16
BATF	958	basic leucine zipper tra	ENSG0000C	10538	3,47	4,89E-05
BATF3	28915	basic leucine zipper tra	ENSG0000C	55509	4,33	6,18E-09
BCHE	983	butyrylcholinesterase [S	ENSG0000C	590	2,17	4,98E-05
BCL2	990	B-cell CLL/lymphoma 2	ENSG0000C	596	3,46	1,15E-13
BCL2A1	991	BCL2-related protein A1	ENSG0000C	597	3,64	6,08E-03
BCL3	998	B-cell CLL/lymphoma 3	ENSG0000C	602	2,21	6,96E-05
BCL6	1001	B-cell CLL/lymphoma 6	ENSG0000C	604	4,06	6,24E-11
BCO2	18503	beta-carotene oxygenas	ENSG0000C	83875	2,27	9,07E-04
BDKRB1	1029	bradykinin receptor B1	ENSG0000C	623	3,89	3,18E-04
BDKRB2	1030	bradykinin receptor B2	ENSG0000C	624	4,52	3,06E-07
BEAN1	24160	brain expressed, associ	ENSG0000C	146227	2,82	4,89E-06
BEGAIN	24163	brain-enriched guanylat	ENSG0000C	57596	3,42	3,50E-06
BEND5	25668	BEN domain containing	ENSG0000C	79656	3,74	2,98E-13
BEND6	20871	BEN domain containing	ENSG0000C	221336	2,29	2,30E-03
BEST1	12703	bestrophin 1 [Source:H	ENSG0000C	7439	3,11	6,52E-10
BEX1	1036	brain expressed, X-linke	ENSG0000C	55859	2,90	3,04E-05
BEX2	30933	brain expressed X-linke	ENSG0000C	84707	3,13	6,53E-07
BEX5	27990	brain expressed, X-linke	ENSG0000C	340542	3,48	7,99E-07
BHLHE40	1046	basic helix-loop-helix fa	ENSG0000C	8553	3,62	7,98E-12
BHLHE41	16617	basic helix-loop-helix fa	ENSG0000C	79365	2,82	9,81E-08
BHMT	1047	betaine--homocysteine	ENSG0000C	635	3,42	1,15E-02
BICC1	19351	bicaudal C homolog 1 (I	ENSG0000C	80114	7,17	4,26E-27
BIK	1051	BCL2-interacting killer (	ENSG0000C	638	2,54	1,43E-05
BIN2	1053	bridging integrator 2 [S	ENSG0000C	51411	4,95	6,58E-10
BIRC3	591	baculoviral IAP repeat c	ENSG0000C	330	2,07	7,84E-04
BLNK	14211	B-cell linker [Source:HG	ENSG0000C	29760	3,87	1,21E-07
BMP5	1072	bone morphogenetic pro	ENSG0000C	653	5,39	1,58E-11
BMP8A	21650	bone morphogenetic pro	ENSG0000C	353500	2,47	7,01E-04

BMPR1A	1076	bone morphogenetic pro	ENSG0000C	657	7,10	5,78E-51
BMPR1B	1077	bone morphogenetic pro	ENSG0000C	658	4,81	2,26E-18
BOC	17173	BOC cell adhesion asso	ENSG0000C	91653	8,09	1,25E-43
BRSK1	18994	BR serine/threonine kin	ENSG0000C	84446	2,55	8,88E-07
BRSK2	11405	BR serine/threonine kin	ENSG0000C	9024	2,21	1,05E-03
BSPRY	18232	B-box and SPRY domain	ENSG0000C	54836	4,98	1,78E-08
BST2	1119	bone marrow stromal c	ENSG0000C	684	3,95	1,64E-27
BTC	1121	betacellulin [Source:HG	ENSG0000C	685	4,80	1,53E-14
BTG2	1131	BTG family, member 2 [	ENSG0000C	7832	3,01	1,81E-08
BTK	1133	Bruton agammaglobulin	ENSG0000C	695	4,49	1,11E-09
BTNL9	24176	butyrophilin-like 9 [Sou	ENSG0000C	153579	6,31	4,77E-13
BVES	1152	blood vessel epicardial s	ENSG0000C	11149	2,70	3,29E-04
BVES-AS1	21223	BVES antisense RNA 1 [	ENSG0000C	154442	4,44	3,13E-09
BZRAP1	16831	benzodiazapine recepto	ENSG0000C	9256	7,66	2,70E-24
C10orf105	20304	chromosome 10 open re	ENSG0000C	414152	5,34	4,34E-08
C10orf116					3,18	6,55E-09
C10orf131	31667	chromosome 10 open re	ENSG0000C	#####	2,33	1,02E-03
C10orf82	28500	chromosome 10 open re	ENSG0000C	143379	3,98	7,29E-08
C11orf70	28188	chromosome 11 open re	ENSG0000C	85016	5,16	4,12E-19
C11orf87	33788	chromosome 11 open re	ENSG0000C	399947	3,64	3,65E-04
C11orf92	33789	chromosome 11 open re	ENSG00000196167		4,60	4,74E-12
C11orf93	26978	chromosome 11 open re	ENSG0000C	120376	5,32	9,33E-18
C11orf96	38675	chromosome 11 open re	ENSG0000C	387763	6,80	3,39E-12
C12orf39	28139	chromosome 12 open re	ENSG0000C	80763	2,14	3,70E-02
C12orf57	29521	chromosome 12 open re	ENSG0000C	113246	2,16	3,85E-05
C12orf75	35164	chromosome 12 open re	ENSG0000C	387882	2,21	1,05E-05
C13orf33					2,52	1,17E-03
C14orf132	20346	chromosome 14 open re	ENSG0000C	56967	3,07	3,82E-06
C14orf180	33795	chromosome 14 open re	ENSG0000C	400258	7,55	7,83E-16
C14orf28	19834	chromosome 14 open re	ENSG0000C	122525	2,09	9,02E-09
C14orf64	20111	chromosome 14 open re	ENSG00000246223		4,22	1,83E-07
C16orf54	26649	chromosome 16 open re	ENSG0000C	283897	3,81	4,85E-06
C16orf89	28687	chromosome 16 open re	ENSG0000C	146556	2,46	3,26E-04
C17orf109					3,82	4,69E-09
C17orf57					2,14	7,55E-08
C18orf1					2,02	3,71E-09
C18orf34					3,23	1,95E-06
C19orf21					5,36	6,72E-05
C19orf35	24793	chromosome 19 open re	ENSG0000C	374872	3,08	3,53E-04
C19orf38	34073	chromosome 19 open re	ENSG0000C	255809	2,40	2,66E-03
C19orf76					2,93	1,40E-05
C1orf126					3,57	1,71E-05
C1orf133					3,27	2,05E-07
C1orf140					3,75	4,43E-06
C1orf150					4,10	1,65E-03
C1orf162	28344	chromosome 1 open rea	ENSG0000C	128346	3,27	2,60E-09
C1orf170	28208	chromosome 1 open rea	ENSG0000C	84808	3,03	8,71E-03
C1orf186	25341	chromosome 1 open rea	ENSG0000C	440712	3,66	1,51E-03
C1orf204	27647	chromosome 1 open rea	ENSG0000C	284677	2,74	1,65E-05
C1orf21	15494	chromosome 1 open rea	ENSG0000C	81563	2,00	2,98E-15
C1orf226	34351	chromosome 1 open rea	ENSG0000C	400793	2,19	5,85E-04
C1orf54	26258	chromosome 1 open rea	ENSG0000C	79630	3,50	8,64E-09
C1orf88					4,81	4,68E-14

C1orf95	30491	chromosome 1 open rea	ENSG0000C	375057	4,76	1,62E-06
C1QA	1241	complement component	ENSG0000C	712	9,27	2,35E-40
C1QB	1242	complement component	ENSG0000C	713	9,09	9,19E-43
C1QC	1245	complement component	ENSG0000C	714	9,11	2,84E-33
C1QTNF1	14324	C1q and tumor necrosis	ENSG0000C	114897	3,09	3,67E-05
C1QTNF2	14325	C1q and tumor necrosis	ENSG0000C	114898	5,38	6,72E-18
C1QTNF3	14326	C1q and tumor necrosis	ENSG0000C	114899	3,39	9,84E-11
C1QTNF4	14346	C1q and tumor necrosis	ENSG0000C	114900	4,73	4,48E-11
C1QTNF7	14342	C1q and tumor necrosis	ENSG0000C	114905	8,53	2,14E-36
C1QTNF9	28732	C1q and tumor necrosis	ENSG0000C	338872	3,58	1,83E-07
C1R	1246	complement component	ENSG0000C	715	5,95	2,83E-14
C1S	1247	complement component	ENSG0000C	716	6,45	2,06E-16
C2	1248	complement component	ENSG0000C	717	5,15	3,18E-16
C20orf118					4,02	1,24E-08
C20orf166-AS1	26393	C20orf166 antisense RN	ENSG0000C	253868	6,35	1,84E-15
C20orf202	37254	chromosome 20 open re	ENSG0000C	400831	3,36	6,80E-07
C2CD4B	33628	C2 calcium-dependent c	ENSG0000C	388125	2,17	3,11E-02
C2orf40	24642	chromosome 2 open rea	ENSG0000C	84417	7,48	3,88E-21
C2orf81	34350	chromosome 2 open rea	ENSG0000C	388963	3,49	1,27E-06
C2orf88	28191	chromosome 2 open rea	ENSG0000C	84281	2,18	1,48E-07
C3	1318	complement component	ENSG0000C	718	5,99	2,27E-14
C3AR1	1319	complement component	ENSG0000C	719	3,19	7,41E-05
C3orf15					5,14	7,02E-20
C3orf58	28490	chromosome 3 open rea	ENSG0000C	205428	2,12	2,25E-07
C3orf70	33731	chromosome 3 open rea	ENSG0000C	285382	6,39	4,24E-23
C4orf39					4,22	3,01E-08
C4orf47	34346	chromosome 4 open rea	ENSG0000C	441054	2,58	1,69E-05
C5AR1	1338	complement component	ENSG0000C	728	7,02	3,49E-17
C5orf27	24687	chromosome 5 open rea	ENSG00000236882		2,86	4,07E-03
C5orf38	24226	chromosome 5 open rea	ENSG0000C	153571	2,89	2,13E-05
C5orf62					3,62	9,73E-07
C6	1339	complement component	ENSG0000C	729	7,53	1,34E-23
C6orf25	13937	chromosome 6 open rea	ENSG0000C	80739	3,71	2,46E-05
C6orf97					4,45	4,18E-12
C7	1346	complement component	ENSG0000C	730	8,29	8,47E-36
C7orf10	16001	chromosome 7 open rea	ENSG0000C	79783	2,36	3,02E-04
C7orf53					2,69	5,76E-04
C7orf58					5,10	9,63E-14
C7orf63	26107	chromosome 7 open rea	ENSG0000C	79846	2,86	3,62E-14
C8orf34	30905	chromosome 8 open rea	ENSG0000C	116328	5,17	1,57E-15
C8orf46	28498	chromosome 8 open rea	ENSG0000C	254778	3,69	1,25E-09
C8orf84					6,10	1,12E-25
C9orf129	31116	chromosome 9 open rea	ENSG0000C	445577	3,69	1,96E-06
C9orf131	31418	chromosome 9 open rea	ENSG0000C	138724	3,09	3,00E-04
C9orf71					3,73	3,12E-11
CA12	1371	carbonic anhydrase XII	ENSG0000C	771	4,35	6,77E-16
CA13	14914	carbonic anhydrase XIII	ENSG0000C	377677	4,24	7,82E-05
CA3	1374	carbonic anhydrase III,	ENSG0000C	761	5,13	2,73E-05
CA4	1375	carbonic anhydrase IV [	ENSG0000C	762	2,32	3,70E-03
CA5B	1378	carbonic anhydrase VB,	ENSG0000C	11238	2,28	1,50E-08
CA8	1382	carbonic anhydrase VIII	ENSG0000C	767	6,69	9,43E-14
CAB39L	20290	calcium binding protein	ENSG0000C	81617	2,32	1,81E-11
CABP1	1384	calcium binding protein	ENSG0000C	9478	4,39	4,97E-07

CACNA1C	1390	calcium channel, voltag	ENSG0000C	775	6,11	2,16E-16
CACNA1E	1392	calcium channel, voltag	ENSG0000C	777	3,89	3,73E-09
CACNA1F	1393	calcium channel, voltag	ENSG0000C	778	4,28	1,53E-11
CACNA1G	1394	calcium channel, voltag	ENSG0000C	8913	2,99	1,86E-04
CACNA1H	1395	calcium channel, voltag	ENSG0000C	8912	8,28	9,53E-25
CACNA2D2	1400	calcium channel, voltag	ENSG0000C	9254	3,14	2,44E-11
CACNA2D3	15460	calcium channel, voltag	ENSG0000C	55799	4,96	2,39E-14
CACNB2	1402	calcium channel, voltag	ENSG0000C	783	5,72	3,72E-20
CACNB4	1404	calcium channel, voltag	ENSG0000C	785	4,08	6,27E-15
CADM2	29849	cell adhesion molecule :	ENSG0000C	253559	4,70	3,04E-13
CADM3	17601	cell adhesion molecule :	ENSG0000C	57863	5,27	4,97E-13
CADPS	1426	Ca++-dependent secret	ENSG0000C	8618	5,66	4,35E-07
CALB2	1435	calbindin 2 [Source:HGI	ENSG0000C	794	5,69	1,45E-06
CAMK2B	1461	calcium/calmodulin-dep	ENSG0000C	816	2,49	3,14E-03
CAMK2N1	24190	calcium/calmodulin-dep	ENSG0000C	55450	3,79	1,75E-11
CAND2	30689	cullin-associated and ne	ENSG0000C	23066	2,34	4,51E-07
CAP2	20039	CAP, adenylate cyclase-	ENSG0000C	10486	4,80	1,28E-12
CAPN12	13249	calpain 12 [Source:HGN	ENSG0000C	147968	2,65	3,16E-05
CAPN3	1480	calpain 3, (p94) [Sourc	ENSG0000C	825	2,81	2,55E-16
CAPN6	1483	calpain 6 [Source:HGN(	ENSG0000C	827	6,29	6,24E-09
CAPN9	1486	calpain 9 [Source:HGN(	ENSG0000C	10753	2,11	3,25E-03
CAPS2	16471	calcyphosine 2 [Source:	ENSG0000C	84698	4,05	2,28E-12
CARNS1	29268	carnosine synthase 1 [S	ENSG0000C	57571	2,63	9,46E-05
CASC1	29599	cancer susceptibility car	ENSG0000C	55259	3,54	3,78E-09
CASC2	22933	cancer susceptibility car	ENSG0000C	255082	2,02	1,11E-03
CASQ1	1512	calsequestrin 1 (fast-tw	ENSG0000C	844	5,45	7,34E-14
CASQ2	1513	calsequestrin 2 (cardiac	ENSG0000C	845	9,23	1,46E-40
CASS4	15878	Cas scaffolding protein	ENSG0000C	57091	2,67	1,44E-04
CASZ1	26002	castor zinc finger 1 [So	ENSG0000C	54897	2,87	2,45E-18
CBFA2T3	1537	core-binding factor, run	ENSG0000C	863	5,66	4,87E-16
CBLN1	1543	cerebellin 1 precursor [	ENSG0000C	869	2,78	1,08E-02
CBR3	1549	carbonyl reductase 3 [S	ENSG0000C	874	3,65	6,43E-09
CBR3-AS1	43664	CBR3 antisense RNA 1 [	ENSG0000C	#####	2,43	5,15E-06
CBX7	1557	chromobox homolog 7 [	ENSG0000C	23492	2,88	1,54E-26
CC2D2B	31666	coiled-coil and C2 doma	ENSG0000C	387707	4,05	1,44E-11
CCBE1	29426	collagen and calcium bi	ENSG0000C	147372	4,63	5,92E-06
CCBP2					2,53	5,95E-12
CCDC101	25156	coiled-coil domain conta	ENSG0000C	112869	2,22	6,07E-06
CCDC102B	26295	coiled-coil domain conta	ENSG0000C	79839	2,13	1,03E-05
CCDC121	25833	coiled-coil domain conta	ENSG0000C	79635	2,14	5,24E-06
CCDC136	22225	coiled-coil domain conta	ENSG0000C	64753	3,15	5,56E-15
CCDC141	26821	coiled-coil domain conta	ENSG0000C	285025	4,38	1,13E-23
CCDC146	29296	coiled-coil domain conta	ENSG0000C	57639	3,71	1,83E-31
CCDC152	34438	coiled-coil domain conta	ENSG0000C	#####	3,74	2,00E-13
CCDC158	26374	coiled-coil domain conta	ENSG0000C	339965	4,68	1,33E-14
CCDC17	26574	coiled-coil domain conta	ENSG0000C	149483	3,23	2,00E-13
CCDC3	23813	coiled-coil domain conta	ENSG0000C	83643	5,24	1,30E-08
CCDC30	26103	coiled-coil domain conta	ENSG0000C	728621	2,12	1,31E-04
CCDC69	24487	coiled-coil domain conta	ENSG0000C	26112	3,35	2,05E-11
CCDC8	25367	coiled-coil domain conta	ENSG0000C	83987	5,69	9,59E-29
CCDC80	30649	coiled-coil domain conta	ENSG0000C	151887	2,59	2,65E-06
CCDC81	26281	coiled-coil domain conta	ENSG0000C	60494	4,47	7,79E-14
CCDC89	26762	coiled-coil domain conta	ENSG0000C	220388	4,58	8,35E-14

CCL13	10611	chemokine (C-C motif)	ENSG0000C	6357	6,54	2,41E-24
CCL19	10617	chemokine (C-C motif)	ENSG0000C	6363	5,83	8,53E-06
CCL21	10620	chemokine (C-C motif)	ENSG0000C	6366	3,03	9,94E-05
CCL26	10625	chemokine (C-C motif)	ENSG0000C	10344	2,35	9,12E-03
CCL3	10627	chemokine (C-C motif)	ENSG0000C	6348	7,14	1,28E-11
CCL4	10630	chemokine (C-C motif)	ENSG0000C	6351	6,12	2,61E-08
CCL8	10635	chemokine (C-C motif)	ENSG0000C	6355	4,72	1,62E-06
CCNL1	20569	cyclin L1 [Source:HGNC	ENSG0000C	57018	2,36	7,05E-07
CCR1	1602	chemokine (C-C motif)	ENSG0000C	1230	5,21	2,62E-14
CCR7	1608	chemokine (C-C motif)	ENSG0000C	1236	3,29	1,23E-03
CCRL1					6,02	7,67E-14
CCT6B	1621	chaperonin containing T	ENSG0000C	10693	2,29	1,15E-09
CD14	1628	CD14 molecule [Source	ENSG0000C	929	2,84	1,62E-05
CD160	17013	CD160 molecule [Sourc	ENSG0000C	11126	2,29	2,48E-05
CD163	1631	CD163 molecule [Sourc	ENSG0000C	9332	6,32	7,44E-08
CD1C	1636	CD1c molecule [Source	ENSG0000C	911	4,71	1,05E-07
CD200R1	24235	CD200 receptor 1 [Sour	ENSG0000C	131450	4,29	9,89E-07
CD209	1641	CD209 molecule [Sourc	ENSG0000C	30835	4,86	9,82E-15
CD24	1645	CD24 molecule [Source	ENSG0000C	#####	2,75	3,94E-05
CD248	18219	CD248 molecule, endos	ENSG0000C	57124	7,26	1,83E-42
CD28	1653	CD28 molecule [Source	ENSG0000C	940	3,65	4,06E-08
CD300A	19319	CD300a molecule [Sour	ENSG0000C	11314	5,18	4,81E-11
CD300E	28874	CD300e molecule [Sour	ENSG0000C	342510	4,30	4,64E-07
CD300LG	30455	CD300 molecule-like fa	ENSG0000C	146894	3,56	1,53E-04
CD4	1678	CD4 molecule [Source:I	ENSG0000C	920	7,68	1,73E-30
CD44	1681	CD44 molecule (Indian	ENSG0000C	960	2,80	6,30E-07
CD48	1683	CD48 molecule [Source	ENSG0000C	962	5,37	2,13E-07
CD52	1804	CD52 molecule [Source	ENSG0000C	1043	5,28	4,02E-06
CD53	1686	CD53 molecule [Source	ENSG0000C	963	4,68	1,90E-07
CD69	1694	CD69 molecule [Source	ENSG0000C	969	6,56	1,84E-06
CD7	1695	CD7 molecule [Source:I	ENSG0000C	924	3,84	1,64E-04
CD74	1697	CD74 molecule, major I	ENSG0000C	972	9,41	2,27E-47
CD79B	1699	CD79b molecule, immu	ENSG0000C	974	2,11	1,48E-03
CD83	1703	CD83 molecule [Source	ENSG0000C	9308	2,68	7,50E-06
CD84	1704	CD84 molecule [Source	ENSG0000C	8832	5,61	1,25E-15
CD86	1705	CD86 molecule [Source	ENSG0000C	942	5,16	6,30E-14
CDC37L1	17179	cell division cycle 37-lik	ENSG0000C	55664	2,42	1,59E-08
CDC42BPG	29829	CDC42 binding protein I	ENSG0000C	55561	2,37	6,42E-04
CDC42EP4	17147	CDC42 effector protein	ENSG0000C	23580	2,32	2,72E-04
CDH19	1758	cadherin 19, type 2 [So	ENSG0000C	28513	5,32	1,92E-11
CDH20	1760	cadherin 20, type 2 [So	ENSG0000C	28316	2,24	1,36E-02
CDH23	13733	cadherin-related 23 [So	ENSG0000C	64072	7,82	3,23E-15
CDH26	15902	cadherin 26 [Source:HG	ENSG0000C	60437	3,02	2,76E-06
CDHR3	26308	cadherin-related family	ENSG0000C	222256	3,10	2,30E-04
CDHR5	7521	cadherin-related family	ENSG0000C	53841	4,45	2,56E-08
CDK14	8883	cyclin-dependent kinase	ENSG0000C	5218	4,67	1,56E-25
CDK15	14434	cyclin-dependent kinase	ENSG0000C	65061	3,95	4,17E-09
CDK18	8751	cyclin-dependent kinase	ENSG0000C	5129	2,66	1,77E-07
CDK20	21420	cyclin-dependent kinase	ENSG0000C	23552	2,50	1,29E-06
CDO1	1795	cysteine dioxygenase ty	ENSG0000C	1036	5,02	5,13E-11
CDON	17104	cell adhesion associatec	ENSG0000C	50937	3,59	3,46E-12
CDR1	1798	cerebellar degeneration	ENSG0000C	1038	7,75	4,30E-13
CDS1	1800	CDP-diacylglycerol synt	ENSG0000C	1040	2,21	2,79E-03

CEBPA	1833	CCAAT/enhancer bindin	ENSG0000C	1050	5,13	8,66E-18
CEBPD	1835	CCAAT/enhancer bindin	ENSG0000C	1052	4,14	4,73E-06
CECR7	1845	cat eye syndrome chror	ENSG0000C	#####	5,14	1,81E-13
CELF6	14059	CUGBP, Elav-like family	ENSG0000C	60677	3,33	2,59E-16
CES1	1863	carboxylesterase 1 [Sou	ENSG0000C	1066	7,69	6,13E-21
CES1P1	18546	carboxylesterase 1 pseu	ENSG0000C	51716	3,93	2,87E-02
CFB	1037	complement factor B [S	ENSG0000C	629	5,11	1,98E-16
CFD	2771	complement factor D (a	ENSG0000C	1675	9,21	1,49E-42
CFH	4883	complement factor H [S	ENSG0000C	3075	2,92	1,41E-03
CFHR3	16980	complement factor H-re	ENSG0000C	10878	3,14	2,39E-02
CFP	8864	complement factor prop	ENSG0000C	5199	3,60	4,10E-07
CG030					2,48	1,31E-05
CHAD	1909	chondroadherin [Source	ENSG0000C	1101	2,41	2,74E-04
CHADL	25165	chondroadherin-like [Sc	ENSG0000C	150356	2,06	9,23E-06
CHD5	16816	chromodomain helicase	ENSG0000C	26038	2,91	3,51E-04
CHDH	24288	choline dehydrogenase	ENSG0000C	55349	3,96	9,17E-11
CHI3L2	1933	chitinase 3-like 2 [Sour	ENSG0000C	1117	6,34	2,00E-07
CHL1	1939	cell adhesion molecule I	ENSG0000C	10752	7,72	5,66E-16
CHN2	1944	chimerin 2 [Source:HGI	ENSG0000C	1124	4,65	4,24E-11
CHPF	24291	chondroitin polymerizin	ENSG0000C	79586	3,05	2,17E-05
CHRD	1949	chordin [Source:HGNC :	ENSG0000C	8646	4,76	4,69E-23
CHRDL1	29861	chordin-like 1 [Source:I	ENSG0000C	91851	9,85	8,28E-44
CHRDL2	24168	chordin-like 2 [Source:I	ENSG0000C	25884	2,22	3,87E-04
CHRM3	1952	cholinergic receptor, mu	ENSG0000C	1131	4,59	1,09E-07
CIDEA	1976	cell death-inducing DFF.	ENSG0000C	1149	5,75	2,58E-07
CIDEC	24229	cell death-inducing DFF.	ENSG0000C	63924	7,69	2,21E-07
CIITA	7067	class II, major histocor	ENSG0000C	4261	6,17	2,56E-15
CILP	1980	cartilage intermediate la	ENSG0000C	8483	8,34	2,77E-20
CILP2	24213	cartilage intermediate la	ENSG0000C	148113	3,22	8,62E-04
CISH	1984	cytokine inducible SH2-	ENSG0000C	1154	2,01	5,51E-04
CITED2	1987	Cbp/p300-interacting tr	ENSG0000C	10370	2,19	3,15E-04
CKB	1991	creatine kinase, brain [S	ENSG0000C	1152	5,91	6,74E-12
CKMT2	1996	creatine kinase, mitoch	ENSG0000C	1160	6,45	2,11E-23
CLDN23	17591	claudin 23 [Source:HGN	ENSG0000C	137075	3,13	7,61E-08
CLDND2	28511	claudin domain containi	ENSG0000C	125875	2,91	1,34E-04
CLEC3B	11891	C-type lectin domain fa	ENSG0000C	7123	9,27	1,03E-36
CLEC4A	13257	C-type lectin domain fa	ENSG0000C	50856	3,71	1,21E-12
CLEC4E	14555	C-type lectin domain fa	ENSG0000C	26253	6,17	1,14E-09
CLEC7A	14558	C-type lectin domain fa	ENSG0000C	64581	3,77	7,82E-07
CLIC2	2063	chloride intracellular ch	ENSG0000C	1193	2,41	2,89E-14
CLIC5	13517	chloride intracellular ch	ENSG0000C	53405	5,27	3,09E-15
CLIC6	2065	chloride intracellular ch	ENSG0000C	54102	5,08	9,14E-13
CLMN	19972	calmin (calponin-like, tr	ENSG0000C	79789	3,01	2,06E-06
CLMP	24039	CXADR-like membrane	ENSG0000C	79827	5,70	7,78E-16
CLSTN2	17448	calsyntenin 2 [Source:H	ENSG0000C	64084	2,81	3,51E-15
CLU	2095	clusterin [Source:HGNC	ENSG0000C	1191	3,08	1,26E-06
CLYBL	18355	citrate lyase beta like [S	ENSG0000C	171425	2,62	6,25E-10
CMA1	2097	chymase 1, mast cell [S	ENSG0000C	1215	5,58	1,96E-06
CMAHP	2098	cytidine monophospho-I	ENSG0000C	8418	2,11	1,88E-06
CMKLR1	2121	chemokine-like receptoi	ENSG0000C	1240	7,42	2,82E-24
CMPK2	27015	cytidine monophosphate	ENSG0000C	129607	3,72	4,34E-15
CMTM2	19173	CKLF-like MARVEL trans	ENSG0000C	146225	4,44	1,19E-06
CMTM5	19176	CKLF-like MARVEL trans	ENSG0000C	116173	4,39	3,91E-12

CMYA5	14305	cardiomyopathy associa	ENSG0000C	202333	3,50	4,26E-09
CNIH3	26802	cornichon homolog 3 (D	ENSG0000C	149111	2,23	6,07E-06
CNKSR1	19700	connector enhancer of k	ENSG0000C	10256	3,76	1,51E-04
CNKSR2	19701	connector enhancer of k	ENSG0000C	22866	7,23	1,28E-19
CNN1	2155	calponin 1, basic, smoo	ENSG0000C	1264	9,30	6,00E-27
CNNM1	102	cyclin M1 [Source:HGN(	ENSG0000C	26507	4,19	1,89E-10
CNR1	2159	cannabinoid receptor 1	ENSG0000C	1268	6,31	4,29E-20
CNTFR	2170	ciliary neurotrophic fact	ENSG0000C	1271	8,39	6,01E-18
CNTN1	2171	contactin 1 [Source:HG	ENSG0000C	1272	7,76	9,46E-60
CNTN3	2173	contactin 3 (plasmacytc	ENSG0000C	5067	6,62	8,42E-20
CNTN4	2174	contactin 4 [Source:HG	ENSG0000C	152330	8,30	2,80E-23
COBL	22199	cordon-bleu WH2 repea	ENSG0000C	23242	4,86	2,63E-12
COL10A1	2185	collagen, type X, alpha	ENSG0000C	1300	2,16	1,33E-02
COL14A1	2191	collagen, type XIV, alph	ENSG0000C	7373	5,06	8,33E-08
COL16A1	2193	collagen, type XVI, alph	ENSG0000C	1307	6,71	2,45E-25
COL18A1-AS1	23132	COL18A1 antisense RN/	ENSG0000C	378832	2,53	1,17E-02
COL19A1	2196	collagen, type XIX, alph	ENSG0000C	1310	5,22	2,49E-06
COL1A1	2197	collagen, type I, alpha :	ENSG0000C	1277	8,04	1,62E-18
COL1A2	2198	collagen, type I, alpha 2	ENSG0000C	1278	8,14	1,29E-21
COL21A1	17025	collagen, type XXI, alph	ENSG0000C	81578	3,41	5,13E-11
COL23A1	22990	collagen, type XXIII, alp	ENSG0000C	91522	5,59	2,76E-14
COL25A1	18603	collagen, type XXV, alph	ENSG0000C	84570	3,13	8,64E-07
COL28A1	22442	collagen, type XXVIII, a	ENSG0000C	340267	4,84	2,74E-16
COL3A1	2201	collagen, type III, alpha	ENSG0000C	1281	9,16	2,23E-25
COL4A6	2208	collagen, type IV, alpha	ENSG0000C	1288	4,15	3,51E-05
COL5A3	14864	collagen, type V, alpha :	ENSG0000C	50509	7,39	3,10E-35
COL6A1	2211	collagen, type VI, alpha	ENSG0000C	1291	4,60	4,34E-15
COL6A2	2212	collagen, type VI, alpha	ENSG0000C	1292	7,49	8,10E-29
COL6A3	2213	collagen, type VI, alpha	ENSG0000C	1293	8,55	3,57E-49
COL6A6	27023	collagen, type VI, alpha	ENSG0000C	131873	5,22	1,16E-05
COL7A1	2214	collagen, type VII, alph	ENSG0000C	1294	3,29	3,58E-06
COL8A2	2216	collagen, type VIII, alph	ENSG0000C	1296	3,38	3,74E-15
COMP	2227	cartilage oligomeric mat	ENSG0000C	1311	8,88	2,52E-05
CORIN	19012	corin, serine peptidase	ENSG0000C	10699	2,17	3,41E-03
CORO1A	2252	coronin, actin binding p	ENSG0000C	11151	2,01	1,52E-04
CORO6	21356	coronin 6 [Source:HGN(	ENSG0000C	84940	2,09	6,63E-08
COX4I2	16232	cytochrome c oxidase su	ENSG0000C	84701	5,28	5,16E-16
CPA3	2298	carboxypeptidase A3 (m	ENSG0000C	1359	6,34	1,07E-07
CPB1	2299	carboxypeptidase B1 (ti	ENSG0000C	1360	4,45	1,33E-10
CPE	2303	carboxypeptidase E [So	ENSG0000C	1363	7,28	4,44E-18
CPEB1	21744	cytoplasmic polyadenyla	ENSG0000C	64506	5,54	1,32E-18
CPLX1	2309	complexin 1 [Source:HC	ENSG0000C	10815	3,26	1,96E-05
CPM	2311	carboxypeptidase M [Sc	ENSG0000C	1368	5,30	3,96E-12
CPNE6	2319	copine VI (neuronal) [S	ENSG0000C	9362	5,25	8,12E-10
CPS1	2323	carbamoyl-phosphate sy	ENSG0000C	1373	4,73	1,48E-14
CPVL	14399	carboxypeptidase, vitell	ENSG0000C	54504	6,06	2,05E-30
CPXM1	15771	carboxypeptidase X (M1	ENSG0000C	56265	4,25	1,07E-05
CPZ	2333	carboxypeptidase Z [So	ENSG0000C	8532	6,03	7,89E-12
CR1	2334	complement component	ENSG0000C	1378	4,38	4,65E-10
CRABP2	2339	cellular retinoic acid bin	ENSG0000C	1382	6,00	2,02E-21
CREB3L1	18856	cAMP responsive eleme	ENSG0000C	90993	5,07	7,07E-12
CREB5	16844	cAMP responsive eleme	ENSG0000C	9586	2,70	7,19E-08
CRHBP	2356	corticotropin releasing b	ENSG0000C	1393	5,34	6,73E-06

CRIP1	2360	cysteine-rich protein 1 (	ENSG0000C	1396	6,57	3,24E-11
CRIP3	17751	cysteine-rich protein 3 |	ENSG0000C	401262	3,86	2,88E-09
CRISPLD1	18206	cysteine-rich secretory	ENSG0000C	83690	7,50	1,12E-15
CRISPLD2	25248	cysteine-rich secretory	ENSG0000C	83716	9,43	1,88E-13
CRLF1	2364	cytokine receptor-like fa	ENSG0000C	9244	6,54	2,92E-07
CRYAB	2389	crystallin, alpha B [Sour	ENSG0000C	1410	3,79	4,89E-04
CRYM	2418	crystallin, mu [Source:H	ENSG0000C	1428	5,73	6,23E-12
CSDC2	30359	cold shock domain cont	ENSG0000C	27254	6,14	1,58E-13
CSF1R	2433	colony stimulating facto	ENSG0000C	1436	7,41	1,05E-27
CSF3	2438	colony stimulating facto	ENSG0000C	1440	5,95	4,48E-03
CSF3R	2439	colony stimulating facto	ENSG0000C	1441	7,89	7,82E-11
CSPG4	2466	chondroitin sulfate prot	ENSG0000C	1464	6,02	3,33E-14
CSRNP1	14300	cysteine-serine-rich nuc	ENSG0000C	64651	5,53	3,02E-12
CSRNP3	30729	cysteine-serine-rich nuc	ENSG0000C	80034	5,72	7,26E-22
CSRP1	2469	cysteine and glycine-ric	ENSG0000C	1465	2,72	9,69E-06
CST7	2479	cystatin F (leukocystatir	ENSG0000C	8530	4,74	3,78E-06
CSTA	2481	cystatin A (stefin A) [Sc	ENSG0000C	1475	3,83	5,91E-09
CTF1	2499	cardiotrophin 1 [Source	ENSG0000C	1489	3,04	9,91E-15
CTNND2	2516	catenin (cadherin-assoc	ENSG0000C	1501	5,49	8,66E-07
CTSG	2532	cathepsin G [Source:HG	ENSG0000C	1511	7,28	5,66E-12
CTSH	2535	cathepsin H [Source:HG	ENSG0000C	1512	2,64	1,03E-16
CTSK	2536	cathepsin K [Source:HG	ENSG0000C	1513	6,90	2,56E-40
CTSW	2546	cathepsin W [Source:H(	ENSG0000C	1521	3,28	5,45E-06
CTTNBP2	15679	cortactin binding proteir	ENSG0000C	83992	7,07	2,94E-22
CX3CR1	2558	chemokine (C-X3-C mot	ENSG0000C	1524	3,47	3,11E-05
CXCL10	10637	chemokine (C-X-C motif	ENSG0000C	3627	5,18	8,42E-03
CXCL12	10672	chemokine (C-X-C motif	ENSG0000C	6387	2,28	6,18E-04
CXCL14	10640	chemokine (C-X-C motif	ENSG0000C	9547	9,54	1,93E-45
CXCL2	4603	chemokine (C-X-C motif	ENSG0000C	2920	5,78	2,45E-11
CXCL3	4604	chemokine (C-X-C motif	ENSG0000C	2921	4,32	1,88E-07
CXCL9	7098	chemokine (C-X-C motif	ENSG0000C	4283	5,16	4,85E-06
CXCR1	6026	chemokine (C-X-C motif	ENSG0000C	3577	5,71	1,58E-05
CXCR2	6027	chemokine (C-X-C motif	ENSG0000C	3579	5,78	1,26E-05
CXCR7					3,13	8,00E-11
CXorf21	25667	chromosome X open rea	ENSG0000C	80231	3,59	4,62E-06
CXorf57	25486	chromosome X open rea	ENSG0000C	55086	4,18	1,68E-09
CXorf69					3,23	1,88E-12
CXXC4	24593	CXXC finger protein 4 [S	ENSG0000C	80319	3,75	9,08E-09
CYB5R2	24376	cytochrome b5 reductas	ENSG0000C	51700	2,33	2,30E-02
CYBB	2578	cytochrome b-245, beta	ENSG0000C	1536	7,86	4,15E-33
CYBRD1	20797	cytochrome b reductase	ENSG0000C	79901	4,03	2,90E-14
CYFIP2	13760	cytoplasmic FMR1 intera	ENSG0000C	26999	3,91	1,76E-08
CYP19A1	2594	cytochrome P450, famil	ENSG0000C	1588	3,03	3,07E-03
CYP1B1	2597	cytochrome P450, famil	ENSG0000C	1545	2,88	1,43E-02
CYP21A2	2600	cytochrome P450, famil	ENSG0000C	1589	2,70	1,76E-03
CYP26B1	20581	cytochrome P450, famil	ENSG0000C	56603	3,18	1,50E-05
CYP27B1	2606	cytochrome P450, famil	ENSG0000C	1594	2,06	4,37E-02
CYP27C1	33480	cytochrome P450, famil	ENSG0000C	339761	4,26	2,26E-06
CYP2E1	2631	cytochrome P450, famil	ENSG0000C	1571	2,07	2,12E-04
CYP39A1	17449	cytochrome P450, famil	ENSG0000C	51302	4,86	2,08E-12
CYP4B1	2644	cytochrome P450, famil	ENSG0000C	1580	7,13	9,46E-09
CYP4F12	18857	cytochrome P450, famil	ENSG0000C	66002	6,22	3,81E-18
CYP4F24P	39945	cytochrome P450, famil	ENSG0000C	388514	4,94	9,49E-11

CYP4V2	23198	cytochrome P450, famil	ENSG0000C	285440	3,85	4,68E-47
CYP4X1	20244	cytochrome P450, famil	ENSG0000C	260293	6,33	1,06E-20
CYP4Z1	20583	cytochrome P450, famil	ENSG0000C	199974	3,79	5,88E-09
CYP7B1	2652	cytochrome P450, famil	ENSG0000C	9420	4,61	3,85E-13
CYP8B1	2653	cytochrome P450, famil	ENSG0000C	1582	3,35	4,04E-12
CYS1	18525	cystin 1 [Source:HGNC	ENSG0000C	192668	7,05	1,10E-42
CYSLTR1	17451	cysteinyl leukotriene re	ENSG0000C	10800	6,98	1,92E-25
CYSLTR2	18274	cysteinyl leukotriene re	ENSG0000C	57105	5,39	1,72E-12
CYTH4	9505	cytohesin 4 [Source:HG	ENSG0000C	27128	5,58	2,04E-14
CYTIP	9506	cytohesin 1 interacting	ENSG0000C	9595	5,72	2,01E-09
DAAM2	18143	dishevelled associated a	ENSG0000C	23500	5,30	3,03E-21
DAB1	2661	Dab, reelin signal transo	ENSG0000C	1600	7,65	4,27E-27
DACT1	17748	dishevelled-binding anta	ENSG0000C	51339	6,71	9,64E-23
DACT2	21231	dishevelled-binding anta	ENSG0000C	168002	3,28	7,17E-04
DACT3	30745	dishevelled-binding anta	ENSG0000C	147906	4,23	7,90E-12
DAPP1	16500	dual adaptor of phospho	ENSG0000C	27071	4,05	4,60E-09
DARC	4035	Duffy blood group, atyp	ENSG0000C	2532	8,70	1,47E-20
DBC1	2687	deleted in bladder cance	ENSG0000C	1620	3,96	2,02E-05
DBNDD2	15881	dysbindin (dystrobrevin	ENSG0000C	55861	2,68	4,54E-06
DBP	2697	D site of albumin promo	ENSG0000C	1628	2,32	2,84E-04
DCAF12L1	29395	DDB1 and CUL4 associa	ENSG0000C	139170	5,72	5,65E-18
DCAF12L2	32950	DDB1 and CUL4 associa	ENSG0000C	340578	5,89	5,34E-21
DCDC2	18141	doublecortin domain co	ENSG0000C	51473	4,88	4,87E-05
DCHS2	23111	dachsous 2 (Drosophila	ENSG0000C	54798	3,62	1,44E-05
DCN	2705	decorin [Source:HGNC !	ENSG0000C	1634	10,34	8,88E-44
DCST2	26562	DC-STAMP domain cont	ENSG0000C	127579	3,77	7,89E-13
DDIT3	2726	DNA-damage-inducible	ENSG0000C	1649	2,25	1,87E-07
DDX25	18698	DEAD (Asp-Glu-Ala-Asp	ENSG0000C	29118	3,05	1,67E-05
DDX26B	27334	DEAD/H (Asp-Glu-Ala-A	ENSG0000C	203522	3,52	1,28E-19
DEGS2	20113	delta(4)-desaturase, sp	ENSG0000C	123099	4,39	6,94E-08
DENND1C	26225	DENN/MADD domain co	ENSG0000C	79958	3,28	2,97E-05
DENND2A	22212	DENN/MADD domain co	ENSG0000C	27147	4,88	2,70E-19
DEPDC7	29899	DEP domain containing	ENSG0000C	91614	3,77	7,31E-08
DEPTOR	22953	DEP domain containing	ENSG0000C	64798	4,11	2,97E-12
DES	2770	desmin [Source:HGNC !	ENSG0000C	1674	10,02	7,19E-34
DFNB31	16361	deafness, autosomal re	ENSG0000C	25861	5,19	1,31E-15
DGAT2	16940	diacylglycerol O-acyltra	ENSG0000C	84649	2,35	1,86E-02
DGKG	2853	diacylglycerol kinase, ga	ENSG0000C	1608	2,43	1,17E-03
DIO2	2884	deiodinase, iodothyroni	ENSG0000C	1734	7,22	1,08E-13
DIO3	2885	deiodinase, iodothyroni	ENSG0000C	1735	6,00	5,55E-13
DIO3OS	20348	DIO3 opposite strand/a	ENSG0000C	#####	4,67	1,08E-13
DIRC3	17805	disrupted in renal carcir	ENSG0000C	729582	3,78	5,23E-12
DIXDC1	23695	DIX domain containing	ENSG0000C	85458	2,17	6,15E-10
DKFZP586I1420					2,35	9,07E-08
DKK2	2892	dickkopf WNT signaling	ENSG0000C	27123	3,51	4,71E-04
DLEC1	2899	deleted in lung and eso	ENSG0000C	9940	2,72	5,12E-06
DLG2	2901	discs, large homolog 2 (	ENSG0000C	1740	4,16	3,34E-15
DLG3	2902	discs, large homolog 3 (	ENSG0000C	1741	3,25	2,89E-26
DLGAP2	2906	discs, large (Drosophila	ENSG0000C	9228	6,65	8,17E-26
DLX5	2918	distal-less homeobox 5	ENSG0000C	1749	6,03	1,52E-08
DLX6	2919	distal-less homeobox 6	ENSG0000C	1750	4,94	1,36E-07
DMC1	2927	DNA meiotic recombina	ENSG0000C	11144	2,35	1,24E-05
DMD	2928	dystrophin [Source:HGI	ENSG0000C	1756	2,59	7,82E-11

DMGDH	24475	dimethylglycine dehydr	ENSG0000C	29958	2,04	1,60E-02
DMKN	25063	dermokine [Source:HGI	ENSG0000C	93099	7,50	2,61E-32
DMPK	2933	dystrophia myotonica-p	ENSG0000C	1760	2,07	6,71E-08
DMRT2	2935	doublesex and mab-3 re	ENSG0000C	10655	5,00	1,52E-11
DMRT3	13909	doublesex and mab-3 re	ENSG0000C	58524	6,34	4,65E-15
DMRTA1	13826	DMRT-like family A1 [Sc	ENSG0000C	63951	2,23	1,19E-02
DNAH2	2948	dynein, axonemal, heav	ENSG0000C	146754	3,20	3,86E-04
DNAH7	18661	dynein, axonemal, heav	ENSG0000C	56171	3,61	3,23E-06
DNAJC27	30290	DnaJ (Hsp40) homolog,	ENSG0000C	51277	2,27	3,07E-08
DNM1	2972	dynamin 1 [Source:HGI	ENSG0000C	1759	4,18	2,36E-11
DNM3	29125	dynamin 3 [Source:HGI	ENSG0000C	26052	2,61	1,41E-06
DOCK11	23483	dedicator of cytokinesis	ENSG0000C	139818	7,45	3,88E-48
DOCK2	2988	dedicator of cytokinesis	ENSG0000C	1794	4,91	3,29E-15
DOCK3	2989	dedicator of cytokinesis	ENSG0000C	1795	3,56	8,83E-10
DOK2	2991	docking protein 2, 56kD	ENSG0000C	9046	6,33	5,49E-22
DOK5	16173	docking protein 5 [Sour	ENSG0000C	55816	5,51	4,26E-30
DOK6	28301	docking protein 6 [Sour	ENSG0000C	220164	5,01	2,02E-25
DPEP2	23028	dipeptidase 2 [Source:H	ENSG0000C	64174	3,39	2,76E-09
DPT	3011	dermatopontin [Source:	ENSG0000C	1805	9,71	9,38E-25
DPY19L2	19414	dpy-19-like 2 (C. elegar	ENSG0000C	283417	4,72	1,93E-13
DPYD	3012	dihydropyrimidine dehy	ENSG0000C	1806	2,20	2,48E-12
DPYSL3	3015	dihydropyrimidinase-lik	ENSG0000C	1809	2,97	2,02E-17
DSC2	3036	desmocollin 2 [Source:I	ENSG0000C	1824	4,30	3,04E-12
DSCAML1	14656	Down syndrome cell adl	ENSG0000C	57453	5,21	2,73E-11
DSG2	3049	desmoglein 2 [Source:H	ENSG0000C	1829	2,84	9,72E-04
DSTN	15750	destrin (actin depolyme	ENSG0000C	11034	2,89	6,04E-08
DTNA	3057	dystrobrevin, alpha [So	ENSG0000C	1837	3,29	6,64E-07
DTWD1	30926	DTW domain containing	ENSG0000C	56986	2,09	5,34E-12
DUOX1	3062	dual oxidase 1 [Source:	ENSG0000C	53905	2,16	3,21E-06
DUSP1	3064	dual specificity phospha	ENSG0000C	1843	4,05	2,74E-15
DUSP2	3068	dual specificity phospha	ENSG0000C	1844	5,33	1,16E-09
DUSP26	28161	dual specificity phospha	ENSG0000C	78986	3,32	2,11E-05
DYNC2H1	2962	dynein, cytoplasmic 2, I	ENSG0000C	79659	2,41	4,10E-09
DZIP1L	26551	DAZ interacting zinc fin	ENSG0000C	199221	3,01	3,26E-08
EAF2	23115	ELL associated factor 2	ENSG0000C	55840	2,72	7,92E-07
EBF1	3126	early B-cell factor 1 [So	ENSG0000C	1879	6,04	4,42E-28
EBF2	19090	early B-cell factor 2 [So	ENSG0000C	64641	8,74	1,50E-48
EBF3	19087	early B-cell factor 3 [So	ENSG0000C	253738	3,25	1,31E-04
EBF4	29278	early B-cell factor 4 [So	ENSG0000C	57593	2,74	1,10E-08
ECHDC2	23408	enoyl CoA hydratase do	ENSG0000C	55268	2,27	9,87E-13
ECM1	3153	extracellular matrix pro	ENSG0000C	1893	4,77	3,65E-26
ECM2	3154	extracellular matrix pro	ENSG0000C	1842	8,29	5,26E-58
EDA	3157	ectodysplasin A [Source	ENSG0000C	1896	3,58	2,96E-22
EDIL3	3173	EGF-like repeats and di	ENSG0000C	10085	6,30	2,51E-28
EDNRA	3179	endothelin receptor typ	ENSG0000C	1909	9,42	9,17E-70
EEPD1	22223	endonuclease/exonucle	ENSG0000C	80820	3,32	2,74E-09
EFHA2					2,09	6,86E-13
EFHB	26330	EF-hand domain family,	ENSG0000C	151651	4,34	8,08E-08
EFHC2	26233	EF-hand domain (C-terr	ENSG0000C	80258	6,52	1,94E-18
EFHD1	29556	EF-hand domain family,	ENSG0000C	80303	7,46	2,86E-55
EFS	16898	embryonal Fyn-associat	ENSG0000C	10278	7,82	1,93E-44
EGF	3229	epidermal growth factor	ENSG0000C	1950	2,38	1,33E-03
EGFLAM	26810	EGF-like, fibronectin typ	ENSG0000C	133584	6,32	2,37E-25

EGFR	3236	epidermal growth factor	ENSG0000C	1956	7,10	3,86E-36
EGLN3	14661	egl nine homolog 3 (C.	ENSG0000C	112399	3,27	2,75E-06
EGR1	3238	early growth response 1	ENSG0000C	1958	7,65	3,06E-21
EGR2	3239	early growth response 2	ENSG0000C	1959	7,06	7,75E-18
EGR3	3240	early growth response 3	ENSG0000C	1960	5,75	8,15E-10
EHBP1L1	30682	EH domain binding prot	ENSG0000C	254102	4,12	3,77E-14
EID3	32961	EP300 interacting inhibi	ENSG0000C	493861	3,11	9,75E-08
EIF4E3	31837	eukaryotic translation ir	ENSG0000C	317649	2,38	4,62E-13
ELF3	3318	E74-like factor 3 (ets dc	ENSG0000C	1999	3,41	2,94E-05
ELL2	17064	elongation factor, RNA p	ENSG0000C	22936	2,34	3,76E-02
ELN	3327	elastin [Source:HGNC S	ENSG0000C	2006	6,02	5,59E-16
ELOVL2	14416	ELOVL fatty acid elonga	ENSG0000C	54898	5,84	4,56E-15
ELOVL4	14415	ELOVL fatty acid elonga	ENSG0000C	6785	2,81	3,40E-07
ELOVL7	26292	ELOVL fatty acid elonga	ENSG0000C	79993	6,47	8,93E-13
EMB	30465	embigin [Source:HGNC	ENSG0000C	133418	5,74	1,85E-22
EMILIN2	19881	elastin microfibril interfa	ENSG0000C	84034	2,84	1,25E-07
EMILIN3	16123	elastin microfibril interfa	ENSG0000C	90187	2,92	9,25E-05
EML5	18197	echinoderm microtubule	ENSG0000C	161436	2,09	9,67E-09
EMP1	3333	epithelial membrane pro	ENSG0000C	2012	2,18	2,28E-03
EMP3	3335	epithelial membrane pro	ENSG0000C	2014	3,74	1,03E-09
EMX2	3341	empty spiracles homeob	ENSG0000C	2018	8,25	4,15E-42
EMX2OS	18511	EMX2 opposite strand/a	ENSG0000C	196047	8,47	3,52E-49
EN1	3342	engrailed homeobox 1 [	ENSG0000C	2019	6,32	7,44E-28
ENDOU	14369	endonuclease, polyU-sp	ENSG0000C	8909	6,07	5,64E-21
ENO2	3353	enolase 2 (gamma, neu	ENSG0000C	2026	2,59	1,67E-08
ENOX1	25474	ecto-NOX disulfide-thiol	ENSG0000C	55068	2,60	1,38E-05
ENPEP	3355	glutamyl aminopeptidas	ENSG0000C	2028	5,47	2,99E-22
ENPP1	3356	ectonucleotide pyrophos	ENSG0000C	5167	5,60	4,32E-20
ENPP2	3357	ectonucleotide pyrophos	ENSG0000C	5168	6,57	9,59E-18
ENPP3	3358	ectonucleotide pyrophos	ENSG0000C	5169	3,44	6,58E-09
ENPP4	3359	ectonucleotide pyrophos	ENSG0000C	22875	2,67	7,59E-05
ENPP5	13717	ectonucleotide pyrophos	ENSG0000C	59084	4,41	2,48E-08
ENPP6	23409	ectonucleotide pyrophos	ENSG0000C	133121	3,22	6,01E-08
ENTPD1	3363	ectonucleoside triphosp	ENSG0000C	953	2,10	4,83E-04
ENTPD2	3364	ectonucleoside triphosp	ENSG0000C	954	4,23	3,38E-09
ENTPD3	3365	ectonucleoside triphosp	ENSG0000C	956	4,98	5,37E-09
EPB41L4B	19818	erythrocyte membrane	ENSG0000C	54566	2,82	4,45E-03
EPDR1	17572	ependymin related prote	ENSG0000C	54749	5,97	2,81E-29
EPHA1	3385	EPH receptor A1 [Sourc	ENSG0000C	2041	4,19	1,01E-09
EPHA3	3387	EPH receptor A3 [Sourc	ENSG0000C	2042	8,00	1,16E-30
EPHA5	3389	EPH receptor A5 [Sourc	ENSG0000C	2044	4,16	3,01E-07
EPHB3	3394	EPH receptor B3 [Sourc	ENSG0000C	2049	6,03	1,15E-17
EPHB6	3396	EPH receptor B6 [Sourc	ENSG0000C	2051	6,12	8,62E-29
EPHX2	3402	epoxide hydrolase 2, cy	ENSG0000C	2053	2,81	2,61E-09
EPPK1	15577	epiplakin 1 [Source:HGI	ENSG0000C	83481	4,57	8,66E-07
EPS8	3420	epidermal growth factor	ENSG0000C	2059	4,80	3,59E-09
ERBB2	3430	v-erb-b2 avian erythrob	ENSG0000C	2064	2,18	2,20E-05
ERBB3	3431	v-erb-b2 avian erythrob	ENSG0000C	2065	4,49	6,47E-11
ERBB4	3432	v-erb-b2 avian erythrob	ENSG0000C	2066	2,62	5,58E-06
EREG	3443	epiregulin [Source:HGN	ENSG0000C	2069	4,37	1,02E-04
ESPNL	27937	espin-like [Source:HGN	ENSG0000C	339768	3,72	4,46E-08
ESR1	3467	estrogen receptor 1 [So	ENSG0000C	2099	6,75	2,76E-54
ESRG	39079	embryonic stem cell rela	ENSG0000C	790952	4,28	7,50E-09

ESRRB	3473	estrogen-related recept	ENSG0000C	2103	4,03	3,68E-07
EVI2A	3499	ecotropic viral integratic	ENSG0000C	2123	5,35	5,60E-18
EVI2B	3500	ecotropic viral integratic	ENSG0000C	2124	3,59	4,44E-09
EVPL	3503	envoplakin [Source:HGI	ENSG0000C	2125	4,44	7,54E-10
EXOC3L4	20120	exocyst complex compo	ENSG0000C	91828	4,58	5,55E-13
EXPH5	30578	exophilin 5 [Source:HGI	ENSG0000C	23086	2,95	1,28E-04
EYA2	3520	eyes absent homolog 2	ENSG0000C	2139	6,27	5,31E-10
EYA4	3522	eyes absent homolog 4	ENSG0000C	2070	5,60	1,53E-11
F10	3528	coagulation factor X [Sc	ENSG0000C	2159	6,49	9,40E-25
F13A1	3531	coagulation factor XIII,	ENSG0000C	2162	9,39	8,95E-38
F3	3541	coagulation factor III (t	ENSG0000C	2152	5,54	1,17E-15
F5	3542	coagulation factor V (pr	ENSG0000C	2153	5,17	6,91E-10
F7	3544	coagulation factor VII (s	ENSG0000C	2155	5,47	6,94E-17
F8	3546	coagulation factor VIII,	ENSG0000C	2157	2,15	1,06E-06
FAAH	3553	fatty acid amide hydrola	ENSG0000C	2166	2,54	6,30E-09
FABP3	3557	fatty acid binding protei	ENSG0000C	2170	6,27	6,55E-21
FAIM2	17067	Fas apoptotic inhibitory	ENSG0000C	23017	5,90	2,58E-10
FAM102B	27637	family with sequence si	ENSG0000C	284611	4,07	1,44E-22
FAM105A	25629	family with sequence si	ENSG0000C	54491	4,57	5,92E-15
FAM110C	33340	family with sequence si	ENSG0000C	642273	4,36	3,62E-14
FAM113B					3,10	1,55E-10
FAM129A	16784	family with sequence si	ENSG0000C	116496	6,47	2,45E-25
FAM134B	25964	family with sequence si	ENSG0000C	54463	6,39	1,70E-21
FAM13A	19367	family with sequence si	ENSG0000C	10144	6,36	3,12E-52
FAM13A-AS1	19370	FAM13A antisense RNA	ENSG0000C	285512	3,31	4,80E-12
FAM13C	19371	family with sequence si	ENSG0000C	220965	3,67	7,14E-11
FAM149A	24527	family with sequence si	ENSG0000C	25854	6,75	1,06E-43
FAM150B	27683	family with sequence si	ENSG0000C	285016	8,57	2,18E-09
FAM162B	21549	family with sequence si	ENSG0000C	221303	2,77	3,28E-05
FAM166A	33818	family with sequence si	ENSG0000C	401565	3,41	8,86E-07
FAM166B	34242	family with sequence si	ENSG0000C	730112	3,28	1,26E-05
FAM178B	28036	family with sequence si	ENSG0000C	51252	3,91	1,81E-06
FAM180A	33773	family with sequence si	ENSG0000C	389558	5,65	2,54E-12
FAM180B	34451	family with sequence si	ENSG0000C	399888	7,89	4,70E-25
FAM184A	20991	family with sequence si	ENSG0000C	79632	4,96	1,46E-18
FAM18A					3,02	4,03E-09
FAM190A					5,61	3,65E-20
FAM198A	24485	family with sequence si	ENSG0000C	729085	7,31	2,18E-36
FAM19A5	21592	family with sequence si	ENSG0000C	25817	4,32	1,86E-08
FAM20A	23015	family with sequence si	ENSG0000C	54757	4,87	7,58E-21
FAM20C	22140	family with sequence si	ENSG0000C	56975	3,62	2,83E-05
FAM26F	33391	family with sequence si	ENSG0000C	441168	3,42	1,65E-07
FAM3B	1253	family with sequence si	ENSG0000C	54097	4,56	1,70E-14
FAM43B	31791	family with sequence si	ENSG0000C	163933	4,36	2,40E-07
FAM46A	18345	family with sequence si	ENSG0000C	55603	3,73	6,74E-10
FAM46B	28273	family with sequence si	ENSG0000C	115572	5,75	3,73E-14
FAM46C	24712	family with sequence si	ENSG0000C	54855	3,55	1,47E-06
FAM47E	34343	family with sequence si	ENSG0000C	#####	2,67	5,45E-06
FAM59A					5,97	3,71E-21
FAM65B	13872	family with sequence si	ENSG0000C	9750	3,74	8,16E-05
FAM65C	16168	family with sequence si	ENSG0000C	140876	4,53	2,91E-11
FAM69A	32213	family with sequence si	ENSG0000C	388650	3,19	1,45E-20
FAM70A					5,86	1,74E-11

FAM71A	26541	family with sequence si	ENSG0000C	149647	6,60	1,14E-21
FAM76A	28530	family with sequence si	ENSG0000C	199870	2,16	4,20E-14
FAM82A1					2,26	3,50E-11
FAM84A	20743	family with sequence si	ENSG0000C	151354	7,06	3,82E-07
FAP	3590	fibroblast activation pro	ENSG0000C	2191	4,97	2,92E-09
FAS-AS1					3,67	7,91E-08
FAT1	3595	FAT atypical cadherin 1	ENSG0000C	2195	3,02	2,98E-04
FAT2	3596	FAT atypical cadherin 2	ENSG0000C	2196	2,11	1,39E-02
FAT3	23112	FAT atypical cadherin 3	ENSG0000C	120114	7,39	7,53E-37
FBLN1	3600	fibulin 1 [Source:HGNC	ENSG0000C	2192	9,94	1,53E-24
FBLN2	3601	fibulin 2 [Source:HGNC	ENSG0000C	2199	5,41	2,12E-16
FBLN5	3602	fibulin 5 [Source:HGNC	ENSG0000C	10516	4,74	2,90E-10
FBLN7	26740	fibulin 7 [Source:HGNC	ENSG0000C	129804	2,55	7,06E-11
FBXL13	21658	F-box and leucine-rich r	ENSG0000C	222235	3,68	1,46E-11
FBXL22	27537	F-box and leucine-rich r	ENSG0000C	283807	6,03	1,35E-16
FBXO16	13618	F-box protein 16 [Sourc	ENSG0000C	157574	5,61	3,38E-09
FBXO32	16731	F-box protein 32 [Sourc	ENSG0000C	114907	2,53	2,42E-04
FBXO40	29816	F-box protein 40 [Sourc	ENSG0000C	51725	6,06	4,46E-16
FCAR	3608	Fc fragment of IgA, rece	ENSG0000C	2204	3,38	3,84E-03
FCER1A	3609	Fc fragment of IgE, higl	ENSG0000C	2205	6,60	4,16E-11
FCER1G	3611	Fc fragment of IgE, higl	ENSG0000C	2207	6,06	5,88E-24
FCGBP	13572	Fc fragment of IgG bind	ENSG0000C	8857	3,38	3,46E-04
FCGR2A	3616	Fc fragment of IgG, low	ENSG0000C	2212	4,30	7,54E-16
FCGR2B	3618	Fc fragment of IgG, low	ENSG0000C	2213	7,63	1,61E-26
FCGR3A	3619	Fc fragment of IgG, low	ENSG0000C	2214	5,90	3,89E-12
FCGR3B	3620	Fc fragment of IgG, low	ENSG0000C	2215	6,08	8,44E-06
FCN1	3623	ficolin (collagen/fibrinog	ENSG0000C	2219	5,73	2,39E-08
FFAR2	4501	free fatty acid receptor	ENSG0000C	2867	4,87	1,41E-05
FGD2	3664	FYVE, RhoGEF and PH d	ENSG0000C	221472	6,12	7,21E-16
FGD3	16027	FYVE, RhoGEF and PH d	ENSG0000C	89846	5,08	1,21E-12
FGF1	3665	fibroblast growth factor	ENSG0000C	2246	3,69	1,04E-07
FGF10	3666	fibroblast growth factor	ENSG0000C	2255	3,50	7,43E-06
FGF11	3667	fibroblast growth factor	ENSG0000C	2256	2,52	6,65E-05
FGF18	3674	fibroblast growth factor	ENSG0000C	8817	3,98	4,42E-06
FGF7	3685	fibroblast growth factor	ENSG0000C	2252	9,12	9,63E-70
FGFBP2	29451	fibroblast growth factor	ENSG0000C	83888	4,30	1,47E-08
FGFR2	3689	fibroblast growth factor	ENSG0000C	2263	6,45	4,12E-25
FGFR3	3690	fibroblast growth factor	ENSG0000C	2261	2,49	1,07E-04
FGL2	3696	fibrinogen-like 2 [Sourc	ENSG0000C	10875	8,96	3,98E-28
FGR	3697	feline Gardner-Rasheed	ENSG0000C	2268	4,90	1,04E-07
FHL1	3702	four and a half LIM dom	ENSG0000C	2273	2,05	6,50E-06
FHL5	17371	four and a half LIM dom	ENSG0000C	9457	8,97	7,44E-35
FHOD3	26178	formin homology 2 dom	ENSG0000C	80206	2,35	1,94E-04
FIBIN	33747	fin bud initiation factor	ENSG0000C	387758	8,31	5,63E-32
FIGF	3708	c-fos induced growth fa	ENSG0000C	2277	4,29	4,95E-05
FIGN	13285	fidgetin [Source:HGNC	ENSG0000C	55137	4,86	2,60E-19
FILIP1	21015	filamin A interacting prc	ENSG0000C	27145	4,09	7,57E-10
FLJ13197					2,36	1,73E-05
FLJ31485					6,16	2,57E-13
FLJ34690					4,68	1,11E-08
FLJ42393					2,74	1,77E-03
FLJ42875					4,92	3,07E-12
FLJ43663					2,12	5,98E-08

FLJ43860					4,07	1,53E-06
FMN1	3768	formin 1 [Source:HGNC	ENSG0000C	342184	4,49	1,10E-09
FMN2	14074	formin 2 [Source:HGNC	ENSG0000C	56776	3,74	8,34E-11
FMNL1	1212	formin-like 1 [Source:H	ENSG0000C	752	2,02	2,98E-04
FMO1	3769	flavin containing monoc	ENSG0000C	2326	6,38	5,85E-11
FMO2	3770	flavin containing monoc	ENSG0000C	2327	8,98	6,80E-60
FMO3	3771	flavin containing monoc	ENSG0000C	2328	6,29	1,34E-16
FMOD	3774	fibromodulin [Source:H	ENSG0000C	2331	4,22	4,50E-08
FNDC1	21184	fibronectin type III dom	ENSG0000C	84624	7,64	4,96E-14
FOLH1	3788	folate hydrolase (prosta	ENSG0000C	2346	3,00	7,31E-05
FOLR2	3793	folate receptor 2 (fetal)	ENSG0000C	2350	8,79	1,05E-27
FOS	3796	FBJ murine osteosarcon	ENSG0000C	2353	8,96	3,81E-35
FOSB	3797	FBJ murine osteosarcon	ENSG0000C	2354	9,45	1,99E-28
FOXF2	3810	forkhead box F2 [Sourc	ENSG0000C	2295	4,51	5,75E-13
FOXP2	13875	forkhead box P2 [Sourc	ENSG0000C	93986	3,06	7,13E-04
FOXS1	3735	forkhead box S1 [Sourc	ENSG0000C	2307	2,10	3,83E-02
FP588					2,92	2,35E-04
FPR1	3826	formyl peptide receptor	ENSG0000C	2357	6,37	2,71E-10
FPR2	3827	formyl peptide receptor	ENSG0000C	2358	5,04	1,44E-04
FPR3	3828	formyl peptide receptor	ENSG0000C	2359	4,25	6,83E-09
FREM1	23399	FRAS1 related extracell	ENSG0000C	158326	6,76	1,04E-18
FRMD1	21240	FERM domain containin	ENSG0000C	79981	3,74	4,82E-04
FRMPD4	29007	FERM and PDZ domain	ENSG0000C	9758	3,02	2,26E-04
FRZB	3959	frizzled-related protein	ENSG0000C	2487	7,12	1,11E-23
FSTL3	3973	follistatin-like 3 (secrete	ENSG0000C	10272	2,06	9,40E-03
FXYD1	4025	FXYD domain containin	ENSG0000C	5348	8,53	3,63E-39
FXYD6	4030	FXYD domain containin	ENSG0000C	53826	8,75	3,99E-29
FYB	4036	FYN binding protein [So	ENSG0000C	2533	5,45	6,90E-10
FZD10	4039	frizzled family receptor	ENSG0000C	11211	6,16	2,27E-12
FZD2	4040	frizzled family receptor	ENSG0000C	2535	2,27	4,38E-05
FZD3	4041	frizzled family receptor	ENSG0000C	7976	4,73	6,64E-16
FZD7	4045	frizzled family receptor	ENSG0000C	8324	2,31	2,98E-02
G0S2	30229	G0/G1switch 2 [Source:	ENSG0000C	50486	7,33	4,95E-12
GABBR1	4070	gamma-aminobutyric a	ENSG0000C	2550	4,84	8,56E-22
GABRB2	4082	gamma-aminobutyric a	ENSG0000C	2561	3,84	1,58E-07
GAD1	4092	glutamate decarboxylas	ENSG0000C	2571	4,88	1,97E-06
GADD45B	4096	growth arrest and DNA-	ENSG0000C	4616	2,34	2,36E-05
GADD45G	4097	growth arrest and DNA-	ENSG0000C	10912	5,70	1,06E-12
GAL3ST4	24145	galactose-3-O-sulfotran	ENSG0000C	79690	2,92	1,34E-11
GALM	24063	galactose mutarotase (a	ENSG0000C	130589	2,60	1,63E-12
GALNT13	23242	UDP-N-acetyl-alpha-D-g	ENSG0000C	114805	6,59	1,82E-09
GALNTL1					5,40	1,86E-17
GAPT	26588	GRB2-binding adaptor p	ENSG0000C	202309	4,26	1,73E-05
GARNL3	25425	GTPase activating Rap/I	ENSG0000C	84253	5,50	1,26E-28
GAS1	4165	growth arrest-specific 1	ENSG0000C	2619	6,73	6,00E-63
GAS7	4169	growth arrest-specific 7	ENSG0000C	8522	3,66	1,46E-07
GATA3	4172	GATA binding protein 3	ENSG0000C	2625	2,28	1,99E-03
GATA6	4174	GATA binding protein 6	ENSG0000C	2627	4,46	2,68E-21
GATM	4175	glycine amidinotransfer	ENSG0000C	2628	6,86	1,96E-40
GBP1P1	39561	guanylate binding prote	ENSG0000C	400759	4,53	5,13E-11
GBP4	20480	guanylate binding prote	ENSG0000C	115361	4,27	1,06E-06
GCA	15990	grancalcin, EF-hand cal	ENSG0000C	25801	2,25	8,57E-09
GCK	4195	glucokinase (hexokinas	ENSG0000C	2645	2,67	5,28E-05

GCNT4	17973	glucosaminyl (N-acetyl)	ENSG0000C	51301	4,38	1,46E-11
GDF10	4215	growth differentiation fa	ENSG0000C	2662	8,51	5,16E-26
GDF3	4218	growth differentiation fa	ENSG0000C	9573	2,96	2,18E-05
GDF6	4221	growth differentiation fa	ENSG0000C	392255	5,18	5,28E-06
GDF9	4224	growth differentiation fa	ENSG0000C	2661	3,04	2,13E-03
GEM	4234	GTP binding protein ove	ENSG0000C	2669	8,84	9,09E-47
GFPT2	4242	glutamine-fructose-6-pl	ENSG0000C	9945	7,09	2,66E-09
GFRA2	4244	GDNF family receptor al	ENSG0000C	2675	5,65	1,52E-18
GFRA3	4245	GDNF family receptor al	ENSG0000C	2676	5,36	1,43E-14
GGTA1P	4253	glycoprotein, alpha-gala	ENSG0000C	2681	3,19	3,26E-12
GHRLOS2					2,19	1,15E-03
GLB1L2	25129	galactosidase, beta 1-lil	ENSG0000C	89944	4,01	7,38E-13
GLCCI1	18713	glucocorticoid induced t	ENSG0000C	113263	2,14	2,82E-07
GLI1	4317	GLI family zinc finger 1	ENSG0000C	2735	3,99	3,35E-08
GLI2	4318	GLI family zinc finger 2	ENSG0000C	2736	5,07	3,29E-17
GLI3	4319	GLI family zinc finger 3	ENSG0000C	2737	3,17	2,96E-05
GLIPR1L2	28592	GLI pathogenesis-relate	ENSG0000C	144321	3,43	7,52E-06
GLIS1	29525	GLIS family zinc finger	ENSG0000C	148979	3,80	4,06E-05
GLP1R	4324	glucagon-like peptide 1	ENSG0000C	2740	4,76	1,24E-10
GLT1D1	26483	glycosyltransferase 1 dc	ENSG0000C	144423	3,47	6,69E-04
GLT25D2					3,55	2,27E-03
GLT8D2	24890	glycosyltransferase 8 dc	ENSG0000C	83468	3,07	1,48E-08
GLUL	4341	glutamate-ammonia liga	ENSG0000C	2752	2,54	8,06E-07
GLYAT	13734	glycine-N-acyltransferas	ENSG0000C	10249	4,71	1,80E-04
GNA15	4383	guanine nucleotide bind	ENSG0000C	2769	2,80	2,06E-04
GNAL	4388	guanine nucleotide bind	ENSG0000C	2774	5,44	5,18E-19
GNAO1	4389	guanine nucleotide bind	ENSG0000C	2775	6,45	1,73E-19
GNAZ	4395	guanine nucleotide bind	ENSG0000C	2781	4,75	1,15E-18
GNB3	4400	guanine nucleotide bind	ENSG0000C	2784	2,43	2,83E-05
GNLY	4414	granulysin [Source:HGN	ENSG0000C	10578	5,02	1,62E-10
GOLGA2P5					3,91	4,70E-13
GOLGA8A	31972	golgin A8 family, memb	ENSG0000C	23015	2,12	4,00E-06
GP5	4443	glycoprotein V (platelet	ENSG0000C	2814	2,40	2,60E-04
GPBAR1	19680	G protein-coupled bile a	ENSG0000C	151306	3,62	1,95E-06
GPC3	4451	glypican 3 [Source:HGN	ENSG0000C	2719	10,08	1,15E-52
GPC6	4454	glypican 6 [Source:HGN	ENSG0000C	10082	8,40	6,26E-25
GPD1	4455	glycerol-3-phosphate de	ENSG0000C	2819	8,02	9,17E-08
GPLD1	4459	glycosylphosphatidylino	ENSG0000C	2822	3,48	5,16E-09
GPM6A	4460	glycoprotein M6A [Sour	ENSG0000C	2823	7,08	2,57E-47
GPM6B	4461	glycoprotein M6B [Sour	ENSG0000C	2824	4,68	1,70E-22
GPNMB	4462	glycoprotein (transmem	ENSG0000C	10457	9,14	4,69E-57
GPR123	13838	G protein-coupled recep	ENSG0000C	84435	5,28	6,54E-10
GPR132	17482	G protein-coupled recep	ENSG0000C	29933	3,95	1,37E-07
GPR133	19893	G protein-coupled recep	ENSG0000C	283383	7,03	5,82E-27
GPR17	4471	G protein-coupled recep	ENSG0000C	2840	3,87	3,69E-09
GPR183	3128	G protein-coupled recep	ENSG0000C	1880	7,21	1,42E-39
GPR21	4476	G protein-coupled recep	ENSG0000C	2844	4,29	6,25E-10
GPR3	4484	G protein-coupled recep	ENSG0000C	2827	2,08	9,91E-03
GPR34	4490	G protein-coupled recep	ENSG0000C	2857	7,86	2,70E-28
GPR35	4492	G protein-coupled recep	ENSG0000C	2859	2,42	1,74E-05
GPR63	13302	G protein-coupled recep	ENSG0000C	81491	4,01	3,84E-11
GPR64	4516	G protein-coupled recep	ENSG0000C	10149	2,87	2,35E-03
GPR65	4517	G protein-coupled recep	ENSG0000C	8477	3,36	2,42E-04

GPR85	4536	G protein-coupled recep	ENSG0000C	54329	2,80	8,71E-07
GPR88	4539	G protein-coupled recep	ENSG0000C	54112	2,87	3,26E-03
GPRASP1	24834	G protein-coupled recep	ENSG0000C	9737	3,83	1,81E-35
GPRC5C	13309	G protein-coupled recep	ENSG0000C	55890	3,97	5,54E-06
GPX3	4555	glutathione peroxidase	ENSG0000C	2878	3,46	3,85E-09
GREB1	24885	growth regulation by es	ENSG0000C	9687	5,91	1,61E-47
GREM1	2001	gremlin 1, DAN family E	ENSG0000C	26585	5,07	3,36E-06
GREM2	17655	gremlin 2, DAN family E	ENSG0000C	64388	6,41	2,19E-12
GRHL1	17923	grainyhead-like 1 (Dros	ENSG0000C	29841	2,54	1,70E-03
GRIA1	4571	glutamate receptor, ion	ENSG0000C	2890	5,48	2,48E-07
GRIA2	4572	glutamate receptor, ion	ENSG0000C	2891	3,65	1,04E-04
GRIA3	4573	glutamate receptor, ion	ENSG0000C	2892	6,81	1,14E-25
GRID1	4575	glutamate receptor, ion	ENSG0000C	2894	5,41	7,24E-16
GRIK2	4580	glutamate receptor, ion	ENSG0000C	2898	4,60	4,13E-11
GRIK3	4581	glutamate receptor, ion	ENSG0000C	2899	4,40	3,89E-07
GRIK5	4583	glutamate receptor, ion	ENSG0000C	2901	5,17	1,26E-12
GRIN2A	4585	glutamate receptor, ion	ENSG0000C	2903	6,61	8,85E-07
GRIN2B	4586	glutamate receptor, ion	ENSG0000C	2904	2,11	4,37E-03
GRIP1	18708	glutamate receptor inte	ENSG0000C	23426	3,81	1,53E-08
GRIP2	23841	glutamate receptor inte	ENSG00000144596		5,15	2,76E-13
GRM2	4594	glutamate receptor, me	ENSG0000C	2912	3,48	1,55E-06
GRTP1	20310	growth hormone regula	ENSG0000C	79774	2,43	2,13E-05
GSC	4612	goosecoid homeobox [S	ENSG0000C	145258	5,52	4,27E-14
GSN	4620	gelsolin [Source:HGNC	ENSG0000C	2934	2,44	2,12E-06
GSTA3	4628	glutathione S-transfera	ENSG0000C	2940	3,69	1,29E-05
GSTM2P1	38009	glutathione S-transfera	ENSG0000C	442245	2,07	9,37E-06
GSTM5	4637	glutathione S-transfera	ENSG0000C	2949	8,07	5,02E-48
GTF2IRD2	30775	GTF2I repeat domain cc	ENSG0000C	84163	2,69	1,03E-09
GUCY1A2	4684	guanylate cyclase 1, sol	ENSG0000C	2977	6,23	7,61E-12
GUCY1A3	4685	guanylate cyclase 1, sol	ENSG0000C	2982	2,11	1,01E-08
GULP1	18649	GULP, engulfment adapt	ENSG0000C	51454	3,61	3,24E-12
GXYLT2	33383	glucoside xylosyltransfe	ENSG0000C	727936	2,20	1,11E-02
GYG2	4700	glycogenin 2 [Source:H	ENSG0000C	8908	2,29	2,29E-02
H19	4713	H19, imprinted materna	ENSG0000C	283120	5,45	2,04E-12
H2AFY2	14453	H2A histone family, me	ENSG0000C	55506	3,65	3,66E-06
H3F3B	4765	H3 histone, family 3B (I	ENSG0000C	3020	2,01	1,21E-08
HAAO	4796	3-hydroxyanthranilate 3	ENSG0000C	23498	6,74	2,05E-30
HAL	4806	histidine ammonia-lyase	ENSG0000C	3034	2,44	6,10E-03
HAND2	4808	heart and neural crest c	ENSG0000C	9464	6,60	4,07E-11
HAPLN2	17410	hyaluronan and proteog	ENSG0000C	60484	5,41	1,17E-09
HAS1	4818	hyaluronan synthase 1	ENSG0000C	3036	7,29	1,54E-06
HAS2	4819	hyaluronan synthase 2	ENSG0000C	3037	8,20	3,71E-10
HAS2-AS1	34340	HAS2 antisense RNA 1 |	ENSG0000C	594842	4,09	5,94E-07
HAVCR2	18437	hepatitis A virus cellula	ENSG0000C	84868	3,89	5,01E-10
HBA1	4823	hemoglobin, alpha 1 [S	ENSG0000C	3039	6,04	1,43E-08
HBA2	4824	hemoglobin, alpha 2 [S	ENSG0000C	3039	7,81	2,14E-11
HBB	4827	hemoglobin, beta [Sour	ENSG0000C	3043	9,31	2,48E-15
HCAR2	24827	hydroxycarboxylic acid	ENSG0000C	338442	5,69	2,03E-06
HCAR3	16824	hydroxycarboxylic acid	ENSG0000C	8843	3,86	1,99E-04
HCFC1R1	21198	host cell factor C1 regul	ENSG0000C	54985	2,34	2,88E-08
HCG23	19713	HLA complex group 23	ENSG0000C	414764	2,93	1,49E-05
HCG26					5,40	4,28E-14
HCK	4840	hemopoietic cell kinase	ENSG0000C	3055	4,09	1,80E-08

HCP5	21659	HLA complex P5 (non-p	ENSG0000C	10866	4,50	9,30E-17
HCST	16977	hematopoietic cell signa	ENSG0000C	10870	4,89	1,56E-16
HDC	4855	histidine decarboxylase	ENSG0000C	3067	5,64	6,05E-09
HDDC2	21078	HD domain containing 2	ENSG0000C	51020	2,57	2,47E-13
HERC5	24368	HECT and RLD domain (	ENSG0000C	51191	3,91	4,01E-16
HES6	18254	hairy and enhancer of s	ENSG0000C	55502	2,14	2,94E-03
HEY2	4881	hairy/enhancer-of-split	ENSG0000C	23493	5,85	7,35E-16
HEYL	4882	hairy/enhancer-of-split	ENSG0000C	26508	6,92	9,49E-15
HFM1	20193	HFM1, ATP-dependent D	ENSG0000C	164045	5,22	3,91E-17
HGF	4893	hepatocyte growth facto	ENSG0000C	3082	6,15	4,34E-31
HHATL	13242	hedgehog acyltransfera	ENSG0000C	57467	5,45	1,65E-08
HIF3A	15825	hypoxia inducible factor	ENSG0000C	64344	2,92	8,29E-14
HIST1H1E	4718	histone cluster 1, H1e [	ENSG0000C	3008	2,71	9,74E-04
HIST1H3A	4766	histone cluster 1, H3a [	ENSG0000C	8350	2,25	9,62E-03
HK3	4925	hexokinase 3 (white cel	ENSG0000C	3101	4,65	1,49E-11
HLA-DMA	4934	major histocompatibility	ENSG0000C	3108	2,42	1,06E-05
HLA-DMB	4935	major histocompatibility	ENSG0000C	3109	4,88	9,72E-23
HLA-DOA	4936	major histocompatibility	ENSG0000C	3111	7,10	3,76E-16
HLA-DOB	4937	major histocompatibility	ENSG0000C	3112	3,34	1,29E-04
HLA-DPA1	4938	major histocompatibility	ENSG0000C	3113	4,19	1,34E-14
HLA-DPB1	4940	major histocompatibility	ENSG0000C	3115	4,23	2,33E-15
HLA-DQA1	4942	major histocompatibility	ENSG0000C	3117	8,10	4,06E-25
HLA-DQA2	4943	major histocompatibility	ENSG0000C	3118	6,63	1,10E-06
HLA-DQB1	4944	major histocompatibility	ENSG0000C	3119	9,22	4,74E-23
HLA-DQB2	4945	major histocompatibility	ENSG0000C	3120	4,27	5,57E-05
HLA-DRA	4947	major histocompatibility	ENSG0000C	3122	9,72	6,77E-42
HLA-DRB1	4948	major histocompatibility	ENSG0000C	3123	8,66	1,73E-25
HLA-DRB5	4953	major histocompatibility	ENSG0000C	3127	9,44	2,72E-12
HLA-DRB6	4954	major histocompatibility	ENSG0000C	3128	6,92	5,54E-09
HLA-F	4963	major histocompatibility	ENSG0000C	3134	3,25	1,13E-20
HLF	4977	hepatic leukemia factor	ENSG0000C	3131	8,25	4,81E-60
HMGCLL1	21359	3-hydroxymethyl-3-met	ENSG0000C	54511	5,74	2,40E-19
HMGN5	8013	high mobility group nuc	ENSG0000C	79366	2,13	1,19E-08
HNMT	5028	histamine N-methyltran	ENSG0000C	3176	4,41	5,52E-37
HOMER2	17513	homer homolog 2 (Dros	ENSG0000C	9455	3,02	1,17E-03
HOOK1	19884	hook homolog 1 (Droso	ENSG0000C	51361	5,36	3,20E-11
HOPX	24961	HOP homeobox [Source	ENSG0000C	84525	7,26	1,22E-15
HOTAIR	33510	HOX transcript antisens	ENSG0000C	#####	7,96	1,41E-30
HOXB-AS5	30143	HOXB cluster antisense	ENSG0000C	360205	4,24	3,64E-04
HOXB13	5112	homeobox B13 [Source	ENSG0000C	10481	5,17	1,33E-05
HOXC10	5122	homeobox C10 [Source	ENSG0000C	3226	9,06	7,68E-36
HOXC11	5123	homeobox C11 [Source	ENSG0000C	3227	6,12	5,64E-17
HOXC12	5124	homeobox C12 [Source	ENSG0000C	3228	5,39	6,59E-11
HOXC13	5125	homeobox C13 [Source	ENSG0000C	3229	5,32	1,46E-16
HOXC4	5126	homeobox C4 [Source:I	ENSG0000C	3221	7,54	6,63E-32
HOXC5	5127	homeobox C5 [Source:I	ENSG0000C	3222	5,10	4,22E-15
HOXC6	5128	homeobox C6 [Source:I	ENSG0000C	3223	7,56	7,92E-45
HOXC8	5129	homeobox C8 [Source:I	ENSG0000C	3224	6,14	8,23E-26
HOXC9	5130	homeobox C9 [Source:I	ENSG0000C	3225	5,68	7,09E-21
HPD	5147	4-hydroxyphenylpyruva	ENSG0000C	3242	4,17	1,68E-07
HPGD	5154	hydroxyprostaglandin d	ENSG0000C	3248	7,20	8,46E-27
HPGDS	17890	hematopoietic prostagla	ENSG0000C	27306	6,73	3,51E-16
HPR	5156	haptoglobin-related pro	ENSG0000C	3250	5,16	5,84E-11

HPSE2	18374	heparanase 2 [Source:H	ENSG0000C	60495	3,20	1,32E-06
HR	5172	hair growth associated		ENSG0000C	55806	2,64	3,03E-04
HRASLS5	24978	HRAS-like suppressor fa	ENSG0000C	117245	5,38	5,67E-08
HRC	5178	histidine rich calcium bi	ENSG0000C	3270	6,04	1,34E-20
HRCT1	33872	histidine rich carboxyl te	ENSG0000C	646962	2,11	3,67E-04
HRH2	5183	histamine receptor H2 [	ENSG0000C	3274	5,08	2,04E-10
HS3ST2	5195	heparan sulfate (glucos	ENSG0000C	9956	5,08	6,17E-08
HS6ST3	19134	heparan sulfate 6-O-sul	ENSG0000C	266722	3,96	1,53E-05
HSD11B1	5208	hydroxysteroid (11-beta	ENSG0000C	3290	6,99	1,65E-20
HSD11B2	5209	hydroxysteroid (11-beta	ENSG0000C	3291	3,81	3,10E-08
HSD17B11	22960	hydroxysteroid (17-beta	ENSG0000C	51170	2,19	1,30E-11
HSD17B13	18685	hydroxysteroid (17-beta	ENSG0000C	345275	4,13	1,64E-09
HSD17B3	5212	hydroxysteroid (17-beta	ENSG0000C	3293	2,16	5,68E-03
HSD17B6	23316	hydroxysteroid (17-beta	ENSG0000C	8630	7,11	9,03E-32
HSF4	5227	heat shock transcriptior	ENSG0000C	3299	3,30	8,97E-15
HSH2D	24920	hematopoietic SH2 dom	ENSG0000C	84941	2,36	1,95E-03
HSPA12A	19022	heat shock 70kDa prote	ENSG0000C	259217	2,13	1,67E-04
HSPA1B	5233	heat shock 70kDa prote	ENSG0000C	3303	2,87	3,57E-10
HSPA6	5239	heat shock 70kDa prote	ENSG0000C	3310	4,62	2,49E-06
HSPA7	5240	heat shock 70kDa prote	ENSG0000C	3311	4,61	5,80E-07
HSPB2					7,70	2,82E-29
HSPB3	5248	heat shock 27kDa prote	ENSG0000C	8988	8,77	1,61E-36
HSPB6	26511	heat shock protein, alph	ENSG0000C	126393	7,51	1,22E-24
HSPB7	5249	heat shock 27kDa prote	ENSG0000C	27129	8,99	1,67E-32
HSPB8	30171	heat shock 22kDa prote	ENSG0000C	26353	4,00	5,01E-08
HTR2A	5293	5-hydroxytryptamine (s	ENSG0000C	3356	7,44	5,83E-19
HTR2B	5294	5-hydroxytryptamine (s	ENSG0000C	3357	2,10	3,53E-03
HTR3C	24003	5-hydroxytryptamine (s	ENSG0000C	170572	3,26	1,02E-04
HTR7	5302	5-hydroxytryptamine (s	ENSG0000C	3363	3,46	2,29E-03
HTRA3	30406	HtrA serine peptidase 3	ENSG0000C	94031	4,75	1,33E-15
HUNK	13326	hormonally up-regulate	ENSG0000C	30811	4,78	4,85E-09
HVCN1	28240	hydrogen voltage-gated	ENSG0000C	84329	2,44	8,65E-10
HYDIN	19368	HYDIN, axonemal centr	ENSG0000C	54768	4,70	1,79E-06
HYMAI					3,14	5,62E-06
ICAM4	5347	intercellular adhesion m	ENSG0000C	3386	5,58	2,20E-07
ICAM5	5348	intercellular adhesion m	ENSG0000C	7087	3,95	2,05E-07
ID2	5361	inhibitor of DNA binding	ENSG0000C	3398	3,75	8,25E-18
ID2B					3,68	1,01E-10
ID4	5363	inhibitor of DNA binding	ENSG0000C	3400	8,69	1,11E-33
IDI2-AS1	30885	IDI2 antisense RNA 1 [S	ENSG0000C	55853	2,66	9,50E-05
IDO1	6059	indoleamine 2,3-dioxyg	ENSG0000C	3620	2,96	1,50E-03
IER2	28871	immediate early respon	ENSG0000C	9592	3,10	4,06E-17
IER5	5393	immediate early respon	ENSG0000C	51278	2,15	3,60E-07
IFI44L	17817	interferon-induced prote	ENSG0000C	10964	5,26	5,79E-15
IFIT1	5407	interferon-induced prote	ENSG0000C	3434	3,47	5,86E-09
IFITM1	5412	interferon induced trans	ENSG0000C	8519	7,83	1,76E-74
IGDCC4	13770	immunoglobulin superfa	ENSG0000C	57722	5,12	1,87E-14
IGF1	5464	insulin-like growth facto	ENSG0000C	3479	2,76	4,05E-04
IGF2	5466	insulin-like growth facto	ENSG0000C	3481	5,28	4,19E-06
IGFBP3	5472	insulin-like growth facto	ENSG0000C	3486	2,46	1,23E-06
IGFBP6	5475	insulin-like growth facto	ENSG0000C	3489	5,31	2,28E-14
IGFBPL1	20081	insulin-like growth facto	ENSG0000C	347252	3,56	1,91E-06
IGFN1	24607	immunoglobulin-like an	ENSG0000C	91156	5,60	3,18E-11

IGJ	5713	immunoglobulin J polyp	ENSG0000C	3512	5,01	4,91E-06
IGLL5	38476	immunoglobulin lambda	ENSG0000C	#####	3,42	8,49E-04
IGSF10	26384	immunoglobulin superfa	ENSG0000C	285313	8,38	6,29E-41
IGSF21	28246	immunoglobin superfam	ENSG0000C	84966	5,50	2,92E-09
IGSF6	5953	immunoglobulin superfa	ENSG0000C	10261	2,97	1,14E-04
IGSF9B	32326	immunoglobulin superfa	ENSG0000C	22997	3,51	1,38E-03
IKZF1	13176	IKAROS family zinc fing	ENSG0000C	10320	4,79	4,58E-14
IKZF4	13179	IKAROS family zinc fing	ENSG0000C	64375	2,11	2,66E-05
IL10	5962	interleukin 10 [Source:I	ENSG0000C	3586	4,73	6,62E-10
IL10RA	5964	interleukin 10 receptor,	ENSG0000C	3587	6,34	2,06E-16
IL12RB2	5972	interleukin 12 receptor,	ENSG0000C	3595	3,78	2,81E-07
IL15	5977	interleukin 15 [Source:I	ENSG0000C	3600	3,01	2,98E-08
IL16	5980	interleukin 16 [Source:I	ENSG0000C	3603	2,39	4,95E-05
IL17B	5982	interleukin 17B [Source	ENSG0000C	27190	5,59	2,30E-09
IL17RD	17616	interleukin 17 receptor	ENSG0000C	54756	2,64	4,16E-04
IL17RE	18439	interleukin 17 receptor	ENSG0000C	132014	2,68	2,20E-07
IL18	5986	interleukin 18 (interferc	ENSG0000C	3606	4,87	4,44E-11
IL18R1	5988	interleukin 18 receptor	ENSG0000C	8809	5,07	1,39E-10
IL18RAP	5989	interleukin 18 receptor	ENSG0000C	8807	5,66	1,71E-06
IL1B	5992	interleukin 1, beta [Sou	ENSG0000C	3553	2,31	2,85E-02
IL1R1	5993	interleukin 1 receptor, t	ENSG0000C	3554	2,56	1,04E-04
IL1R2	5994	interleukin 1 receptor, t	ENSG0000C	7850	4,97	4,20E-05
IL1RAPL1	5996	interleukin 1 receptor a	ENSG0000C	11141	2,90	6,32E-04
IL1RL2	5999	interleukin 1 receptor-li	ENSG0000C	8808	4,21	1,64E-07
IL1RN	6000	interleukin 1 receptor a	ENSG0000C	3557	5,63	3,33E-07
IL20RA	6003	interleukin 20 receptor,	ENSG0000C	53832	2,15	5,42E-03
IL28RA					4,43	2,69E-09
IL2RG	6010	interleukin 2 receptor, g	ENSG0000C	3561	2,67	4,72E-05
IL33	16028	interleukin 33 [Source:I	ENSG0000C	90865	2,37	1,22E-02
IL6	6018	interleukin 6 (interferon	ENSG0000C	3569	7,13	7,61E-06
IL8	6025	interleukin 8 [Source:H	ENSG0000C	3576	2,17	1,17E-02
IMPA2	6051	inositol(myo)-1(or 4)-m	ENSG0000C	3613	3,25	1,30E-07
INHBB	6067	inhibin, beta B [Source:	ENSG0000C	3625	3,32	3,47E-05
INMT	6069	indolethylamine N-meth	ENSG0000C	11185	8,51	4,03E-32
INSC	33116	inscuteable homolog (D	ENSG0000C	387755	3,26	4,52E-07
IP6K3	17269	inositol hexakisphospha	ENSG0000C	117283	4,77	1,04E-06
IQSEC3	29193	IQ motif and Sec7 doma	ENSG0000C	440073	3,54	1,37E-05
IRF4	6119	interferon regulatory fa	ENSG0000C	3662	5,24	3,32E-09
IRF5	6120	interferon regulatory fa	ENSG0000C	3663	3,19	1,13E-06
IRF8	5358	interferon regulatory fa	ENSG0000C	3394	4,55	3,17E-07
IRS1	6125	insulin receptor substra	ENSG0000C	3667	5,30	5,38E-20
IRS2	6126	insulin receptor substra	ENSG0000C	8660	3,89	3,50E-05
IRX3	14360	iroquois homeobox 3 [S	ENSG0000C	79191	3,63	3,10E-09
IRX5	14361	iroquois homeobox 5 [S	ENSG0000C	10265	2,99	1,34E-06
IRX6	14675	iroquois homeobox 6 [S	ENSG0000C	79190	5,92	6,17E-14
ISLR	6133	immunoglobulin superfa	ENSG0000C	3671	9,58	3,11E-55
ISLR2	29286	immunoglobulin superfa	ENSG0000C	57611	5,16	4,52E-16
ISM1	16213	isthmin 1, angiogenesis	ENSG0000C	140862	6,60	3,41E-21
ITGA7	6143	integrin, alpha 7 [Sourc	ENSG0000C	3679	8,56	5,48E-39
ITGA8	6144	integrin, alpha 8 [Sourc	ENSG0000C	8516	4,94	5,45E-08
ITGAL	6148	integrin, alpha L (antige	ENSG0000C	3683	3,66	1,45E-04
ITGAM	6149	integrin, alpha M (comp	ENSG0000C	3684	5,29	3,51E-15
ITGB1BP2	6154	integrin beta 1 binding	ENSG0000C	26548	3,97	5,18E-10

ITGB2	6155	integrin, beta 2 (comple	ENSG0000C	3689	4,48	2,99E-15
ITGB4	6158	integrin, beta 4 [Source	ENSG0000C	3691	2,99	7,13E-04
ITGB7	6162	integrin, beta 7 [Source	ENSG0000C	3695	2,39	2,86E-05
ITGBL1	6164	integrin, beta-like 1 (wi	ENSG0000C	9358	2,90	2,40E-06
ITIH1	6166	inter-alpha-trypsin inhit	ENSG0000C	3697	5,24	7,40E-08
ITIH3	6168	inter-alpha-trypsin inhit	ENSG0000C	3699	6,08	3,57E-14
ITIH4	6169	inter-alpha-trypsin inhit	ENSG0000C	3700	4,46	1,88E-12
ITIH5	21449	inter-alpha-trypsin inhit	ENSG0000C	80760	7,56	2,80E-36
ITM2A	6173	integral membrane prot	ENSG0000C	9452	3,82	7,42E-09
ITPKC	14897	inositol-trisphosphate 3	ENSG0000C	80271	2,15	3,78E-02
JAM2	14686	junctional adhesion mol	ENSG0000C	58494	6,06	7,67E-17
JPH2	14202	junctophilin 2 [Source:H	ENSG0000C	57158	5,71	6,56E-11
JUN	6204	jun proto-oncogene [So	ENSG0000C	3725	3,30	4,76E-13
JUNB	6205	jun B proto-oncogene [S	ENSG0000C	3726	5,68	1,91E-21
JUND	6206	jun D proto-oncogene [	ENSG0000C	3727	2,48	4,71E-07
KAL1	6211	Kallmann syndrome 1 s	ENSG0000C	3730	3,09	1,25E-05
KANK2	29300	KN motif and ankyrin re	ENSG0000C	25959	2,37	6,85E-10
KANK4	27263	KN motif and ankyrin re	ENSG0000C	163782	3,78	7,14E-04
KAZALD1	25460	Kazal-type serine peptic	ENSG0000C	81621	3,83	1,11E-07
KAZN	29173	kazrin, periplakin intera	ENSG0000C	23254	2,47	4,12E-06
KBTBD12	25731	kelch repeat and BTB (F	ENSG0000C	166348	3,78	3,23E-07
KCNA5	6224	potassium voltage-gate	ENSG0000C	3741	6,91	2,97E-10
KCNA6	6225	potassium voltage-gate	ENSG0000C	3742	4,69	2,46E-12
KCNAB1	6228	potassium voltage-gate	ENSG0000C	7881	4,63	8,43E-14
KCND3	6239	potassium voltage-gate	ENSG0000C	3752	2,29	8,88E-04
KCNE3	6243	potassium voltage-gate	ENSG0000C	10008	3,48	1,02E-07
KCNE4	6244	potassium voltage-gate	ENSG0000C	23704	3,18	7,62E-04
KCNH2	6251	potassium voltage-gate	ENSG0000C	3757	7,17	5,83E-19
KCNIP2	15522	Kv channel interacting p	ENSG0000C	30819	4,08	5,24E-04
KCNIP3	15523	Kv channel interacting p	ENSG0000C	30818	6,05	8,10E-22
KCNJ1	6255	potassium inwardly-rect	ENSG0000C	3758	3,39	8,53E-05
KCNJ12	6258	potassium inwardly-rect	ENSG0000C	3768	5,98	6,71E-26
KCNJ14	6260	potassium inwardly-rect	ENSG0000C	3770	2,15	1,56E-02
KCNJ15	6261	potassium inwardly-rect	ENSG0000C	3772	2,28	2,32E-03
KCNJ3	6264	potassium inwardly-rect	ENSG0000C	3760	4,99	1,66E-08
KCNJ8	6269	potassium inwardly-rect	ENSG0000C	3764	9,21	3,56E-56
KCNK1	6272	potassium channel, sub	ENSG0000C	3775	3,81	7,08E-13
KCNK15	13814	potassium channel, sub	ENSG0000C	60598	4,11	2,65E-06
KCNK2	6277	potassium channel, sub	ENSG0000C	3776	4,49	1,12E-07
KCNK3	6278	potassium channel, sub	ENSG0000C	3777	4,07	3,31E-06
KCNK5	6280	potassium channel, sub	ENSG0000C	8645	6,22	1,31E-10
KCNK7	6282	potassium channel, sub	ENSG0000C	10089	4,43	6,95E-07
KCNMA1	6284	potassium large conduc	ENSG0000C	3778	8,27	1,22E-36
KCNMB1	6285	potassium large conduc	ENSG0000C	3779	7,44	4,00E-17
KCNN3	6292	potassium intermediate	ENSG0000C	3782	2,13	2,71E-07
KCNN4	6293	potassium intermediate	ENSG0000C	3783	3,68	1,35E-03
KCNQ1	6294	potassium voltage-gate	ENSG0000C	3784	2,75	1,66E-06
KCNQ4	6298	potassium voltage-gate	ENSG0000C	9132	5,26	8,33E-16
KCNS2	6301	potassium voltage-gate	ENSG0000C	3788	6,24	3,28E-19
KCNS3	6302	potassium voltage-gate	ENSG0000C	3790	2,79	3,42E-03
KCNT2	18866	potassium channel, sub	ENSG0000C	343450	5,78	3,92E-13
KCP	17585	kielin/chordin-like prote	ENSG00000135253		5,02	6,96E-08
KDM6B	29012	lysine (K)-specific deme	ENSG0000C	23135	3,05	1,47E-03

KIAA0748					4,27	1,79E-08
KIAA1045	29180	KIAA1045 [Source:HGN	ENSG0000C	23349	4,32	3,69E-09
KIAA1377	29264	KIAA1377 [Source:HGN	ENSG0000C	57562	2,60	1,16E-09
KIAA1456	26725	KIAA1456 [Source:HGN	ENSG0000C	57604	2,22	3,24E-03
KIAA1644	29335	KIAA1644 [Source:HGN	ENSG0000C	85352	7,00	7,53E-17
KIAA1683	29350	KIAA1683 [Source:HGN	ENSG0000C	80726	5,35	2,77E-18
KIAA1755	29372	KIAA1755 [Source:HGN	ENSG0000C	85449	6,43	1,35E-21
KIAA2022	29433	KIAA2022 [Source:HGN	ENSG0000C	340533	4,18	7,90E-10
KIF5C	6325	kinesin family member	ENSG0000C	3800	3,77	2,54E-07
KIRREL2	18816	kin of IRRE like 2 (Dros	ENSG0000C	84063	4,33	9,62E-03
KIT	6342	v-kit Hardy-Zuckerman	ENSG0000C	3815	3,62	2,77E-05
KITLG	6343	KIT ligand [Source:HGN	ENSG0000C	4254	2,02	4,39E-08
KL	6344	klotho [Source:HGNC S	ENSG0000C	9365	5,94	7,64E-21
KLB	15527	klotho beta [Source:HG	ENSG0000C	152831	3,86	3,86E-04
KLF10	11810	Kruppel-like factor 10 [	ENSG0000C	7071	2,61	1,95E-11
KLF11	11811	Kruppel-like factor 11 [	ENSG0000C	8462	2,84	2,62E-19
KLF12	6346	Kruppel-like factor 12 [	ENSG0000C	11278	4,24	1,84E-09
KLF15	14536	Kruppel-like factor 15 [	ENSG0000C	28999	2,59	4,18E-03
KLF2	6347	Kruppel-like factor 2 (lu	ENSG0000C	10365	5,50	4,85E-17
KLF4	6348	Kruppel-like factor 4 (gu	ENSG0000C	9314	7,18	8,62E-36
KLF8	6351	Kruppel-like factor 8 [S	ENSG0000C	11279	5,53	4,57E-29
KLF9	1123	Kruppel-like factor 9 [S	ENSG0000C	687	3,40	6,28E-08
KLHDC1	19836	kelch domain containing	ENSG0000C	122773	2,45	6,05E-17
KLHL13	22931	kelch-like family memb	ENSG0000C	90293	3,10	1,02E-07
KLHL14	29266	kelch-like family memb	ENSG0000C	57565	2,20	7,56E-04
KLHL15	29347	kelch-like family memb	ENSG0000C	80311	2,38	3,05E-04
KLHL30	24770	kelch-like family memb	ENSG0000C	377007	5,88	2,04E-13
KLHL31	21353	kelch-like family memb	ENSG0000C	401265	2,12	3,38E-03
KLKB1	6371	kallikrein B, plasma (Fle	ENSG0000C	3818	2,90	1,37E-06
KRBOX1	38708	KRAB box domain conta	ENSG0000C	#####	5,51	1,14E-10
KREMEN1	17550	kringle containing trans	ENSG0000C	83999	3,22	1,83E-09
KRT17	6427	keratin 17 [Source:HGN	ENSG0000C	3872	4,03	1,23E-03
KY	26576	kyphoscoliosis peptidas	ENSG0000C	339855	3,82	2,46E-08
KYNU	6469	kynureninase [Source:H	ENSG0000C	8942	3,92	3,08E-07
L1CAM	6470	L1 cell adhesion molecu	ENSG0000C	3897	3,70	1,65E-05
LAG3	6476	lymphocyte-activation g	ENSG0000C	3902	3,45	2,60E-06
LAIR1	6477	leukocyte-associated im	ENSG0000C	3903	5,94	1,77E-16
LAMA2	6482	laminin, alpha 2 [Sourc	ENSG0000C	3908	5,15	9,10E-40
LCN10	20892	lipocalin 10 [Source:HG	ENSG0000C	414332	6,54	5,38E-07
LCN6	17337	lipocalin 6 [Source:HGN	ENSG0000C	158062	4,60	7,84E-06
LCNL1	34436	lipocalin-like 1 [Source:	ENSG0000C	401562	4,44	6,30E-06
LCP1	6528	lymphocyte cytosolic pr	ENSG0000C	3936	3,59	1,21E-06
LCP2	6529	lymphocyte cytosolic pr	ENSG0000C	3937	6,78	1,11E-19
LDB3	15710	LIM domain binding 3 [	ENSG0000C	11155	8,92	5,96E-42
LEF1	6551	lymphoid enhancer-binc	ENSG0000C	51176	4,88	1,41E-10
LEFTY2	3122	left-right determination	ENSG0000C	7044	4,97	6,64E-14
LEP	6553	leptin [Source:HGNC Sy	ENSG0000C	3952	7,74	2,04E-05
LGALS12	15788	lectin, galactoside-bindi	ENSG0000C	85329	5,86	2,10E-06
LGALS3BP	6564	lectin, galactoside-bindi	ENSG0000C	3959	4,72	1,29E-13
LGALS4	6565	lectin, galactoside-bindi	ENSG0000C	3960	2,67	6,74E-06
LGI1	6572	leucine-rich, glioma ina	ENSG0000C	9211	6,58	1,34E-28
LGI2	18710	leucine-rich repeat LGI	ENSG0000C	55203	3,29	6,29E-09
LGI3	18711	leucine-rich repeat LGI	ENSG0000C	203190	3,72	1,65E-05

LGI4	18712	leucine-rich repeat LGI	ENSG0000C	163175	7,41	2,47E-54
LGR4	13299	leucine-rich repeat cont	ENSG0000C	55366	3,21	6,47E-10
LGR5	4504	leucine-rich repeat cont	ENSG0000C	8549	4,70	3,72E-05
LHFP	6586	lipoma HMGIC fusion pa	ENSG0000C	10186	2,10	3,71E-12
LIF	6596	leukemia inhibitory fact	ENSG0000C	3976	2,85	4,12E-03
LILRA2	6603	leukocyte immunoglobu	ENSG0000C	11027	4,40	2,84E-07
LILRA5	16309	leukocyte immunoglobu	ENSG0000C	353514	3,95	6,43E-06
LILRA6	15495	leukocyte immunoglobu	ENSG0000C	79168	3,95	2,27E-08
LILRB2	6606	leukocyte immunoglobu	ENSG0000C	10288	5,37	1,03E-16
LILRB3	6607	leukocyte immunoglobu	ENSG0000C	11025	3,22	2,28E-06
LILRB4	6608	leukocyte immunoglobu	ENSG0000C	11006	4,70	2,32E-11
LILRB5	6609	leukocyte immunoglobu	ENSG0000C	10990	6,76	8,63E-18
LIMS2	16084	LIM and senescent cell a	ENSG0000C	55679	2,01	1,33E-09
LINC00087	34500	long intergenic non-pro	ENSG0000C	644596	3,59	2,11E-09
LINC00173	33791	long intergenic non-pro	ENSG0000C	#####	4,51	2,60E-14
LINC00265	28019	long intergenic non-pro	ENSG0000C	349114	2,22	3,36E-06
LINC00273	38595	long intergenic non-pro	ENSG0000C	649159	2,51	2,24E-02
LINC00310	16414	long intergenic non-pro	ENSG0000C	114036	4,59	4,66E-13
LINC00312	6662	long intergenic non-pro	ENSG0000C	29931	5,27	3,06E-14
LINC00324	26628	long intergenic non-pro	ENSG0000C	284029	2,29	1,20E-05
LINC00341	20353	long intergenic non-pro	ENSG0000C	79686	2,51	8,61E-11
LINC00473	21160	long intergenic non-pro	ENSG0000C	90632	4,00	5,33E-03
LINGO3	21206	leucine rich repeat and	ENSG0000C	645191	4,20	1,34E-05
LIPE	6621	lipase, hormone-sensiti	ENSG0000C	3991	2,55	4,22E-03
LITAF	16841	lipopolysaccharide-indu	ENSG0000C	9516	3,25	1,44E-17
LMCD1	6633	LIM and cysteine-rich d	ENSG0000C	29995	2,14	2,92E-07
LMO3	6643	LIM domain only 3 (rho	ENSG0000C	55885	7,79	6,87E-24
LMO4	6644	LIM domain only 4 [Sou	ENSG0000C	8543	2,25	4,74E-09
LMOD1	6647	leiomodin 1 (smooth mu	ENSG0000C	25802	9,31	4,33E-53
LMX1A	6653	LIM homeobox transcrip	ENSG0000C	4009	5,40	1,18E-11
LMX1B	6654	LIM homeobox transcrip	ENSG0000C	4010	4,01	1,13E-07
LNP1	28014	leukemia NUP98 fusion	ENSG0000C	348801	5,68	4,87E-19
LOC100127888					3,53	1,46E-05
LOC100127983					6,04	3,11E-29
LOC100128593					3,16	3,48E-04
LOC100129269					3,93	1,19E-05
LOC100129794					2,21	7,28E-04
LOC100130417					3,96	6,36E-04
LOC100130705					3,87	8,63E-04
LOC100130855					2,05	2,64E-04
LOC100130872					3,82	1,67E-07
LOC100130992					4,84	9,72E-19
LOC100131825					6,04	1,50E-07
LOC100132215					2,43	1,61E-05
LOC100132891					7,05	1,27E-43
LOC100216545					2,11	2,33E-10
LOC100233209					3,61	2,98E-08
LOC100240735					4,08	2,68E-08
LOC100270746					3,32	1,07E-06
LOC100287616					3,01	1,81E-06
LOC100287846					3,60	1,93E-08
LOC100288122					2,31	1,18E-04
LOC100288123					2,15	2,94E-03

LOC100289019	2,07	1,26E-06
LOC100289187	3,98	1,52E-11
LOC100289361	2,81	1,73E-06
LOC100289511	2,36	3,56E-05
LOC100302640	3,54	1,23E-09
LOC100302650	4,13	8,99E-05
LOC100499183	2,05	1,65E-03
LOC100499405	2,47	1,33E-05
LOC100505483	5,31	8,52E-22
LOC100505633	4,76	3,94E-08
LOC100505695	3,39	1,33E-09
LOC100505718	4,82	1,74E-11
LOC100505806	4,84	1,07E-09
LOC100505875	4,27	1,40E-10
LOC100505967	3,10	7,40E-03
LOC100506035	5,74	4,40E-14
LOC100506388	4,40	1,90E-10
LOC100506421	4,80	1,42E-06
LOC100506779	4,13	2,17E-09
LOC100506795	5,51	6,21E-18
LOC100506990	2,51	4,29E-08
LOC100507421	3,77	3,78E-06
LOC100507463	3,32	6,03E-11
LOC100507577	2,11	3,16E-06
LOC100507588	2,11	8,39E-03
LOC100507632	5,35	4,83E-07
LOC100616668	2,79	1,11E-07
LOC100652768	2,21	4,69E-05
LOC144571	2,62	1,16E-12
LOC145820	6,68	1,74E-25
LOC154822	3,05	3,41E-05
LOC219347	2,02	3,22E-04
LOC255167	3,52	4,92E-06
LOC283174	4,33	7,11E-05
LOC283335	4,61	3,27E-27
LOC283392	5,45	1,77E-14
LOC283481	3,43	1,02E-15
LOC283547	2,27	1,27E-02
LOC284276	4,53	8,67E-13
LOC284578	3,15	1,92E-04
LOC284581	4,76	2,79E-02
LOC284648	3,56	1,70E-10
LOC284801	2,87	3,02E-02
LOC286367	3,31	1,99E-10
LOC338758	3,23	3,48E-13
LOC339524	6,08	2,05E-30
LOC375010	3,42	1,67E-05
LOC387723	2,01	1,33E-05
LOC388630	6,87	2,07E-19
LOC400043	6,90	7,01E-28
LOC401093	3,79	7,20E-25
LOC439950	2,85	1,09E-02
LOC439990	2,52	9,73E-07

LOC572558					4,55	5,45E-11
LOC643529					3,39	1,82E-06
LOC643723					2,94	1,59E-06
LOC644554					4,08	2,15E-12
LOC644990					4,18	3,46E-09
LOC650368					4,52	3,50E-10
LOC728392					2,16	9,21E-09
LOC728437					4,26	1,01E-02
LOC728819					3,46	2,54E-09
LOC729950					2,81	6,58E-05
LOC80054					3,04	7,94E-08
LOH12CR2	26524	loss of heterozygosity, 1	ENSG00000205791		2,33	4,34E-05
LONRF1	26302	LON peptidase N-termir	ENSG0000C	91694	3,01	1,58E-07
LONRF2	24788	LON peptidase N-termir	ENSG0000C	164832	7,13	4,24E-18
LOXHD1	26521	lipoxygenase homology	ENSG0000C	125336	3,45	3,02E-04
LOXL1	6665	lysyl oxidase-like 1 [Sou	ENSG0000C	4016	3,89	3,11E-07
LOXL3	13869	lysyl oxidase-like 3 [Sou	ENSG0000C	84695	2,64	5,90E-09
LPAR1	3166	lysophosphatidic acid re	ENSG0000C	1902	8,27	1,96E-43
LPHN3	20974	latrophilin 3 [Source:HC	ENSG0000C	23284	7,41	3,94E-16
LPL	6677	lipoprotein lipase [Sour	ENSG0000C	4023	4,12	2,57E-03
LPPR3					2,62	6,71E-03
LPPR4					7,71	5,53E-34
LRFN5	20360	leucine rich repeat and	ENSG0000C	145581	4,57	8,87E-15
LRMP	6690	lymphoid-restricted mei	ENSG0000C	4033	3,02	7,48E-04
LRP1	6692	low density lipoprotein r	ENSG0000C	4035	5,64	3,08E-16
LRP4	6696	low density lipoprotein r	ENSG0000C	4038	3,14	2,12E-09
LRRC16A	21581	leucine rich repeat cont	ENSG0000C	55604	4,29	9,25E-19
LRRC16B	20272	leucine rich repeat cont	ENSG0000C	90668	3,25	1,01E-05
LRRC17	16895	leucine rich repeat cont	ENSG0000C	10234	5,55	9,29E-41
LRRC2	14676	leucine rich repeat cont	ENSG0000C	79442	4,05	3,88E-09
LRRC25	29806	leucine rich repeat cont	ENSG0000C	126364	5,45	4,50E-17
LRRC3B	28105	leucine rich repeat cont	ENSG0000C	116135	4,54	5,96E-04
LRRC4	15586	leucine rich repeat cont	ENSG0000C	64101	2,33	5,65E-04
LRRC4B	25042	leucine rich repeat cont	ENSG0000C	94030	2,67	1,91E-03
LRRC4C	29317	leucine rich repeat cont	ENSG0000C	57689	4,40	3,11E-09
LRRC7	18531	leucine rich repeat cont	ENSG0000C	57554	3,53	1,80E-05
LRRCC1	29373	leucine rich repeat and	ENSG0000C	85444	3,68	3,00E-26
LRRK2	18618	leucine-rich repeat kina	ENSG0000C	120892	6,79	8,81E-52
LRRN1	20980	leucine rich repeat neur	ENSG0000C	57633	4,51	1,07E-07
LRRN3	17200	leucine rich repeat neur	ENSG0000C	54674	4,78	1,26E-07
LRRN4CL	33724	LRRN4 C-terminal like [	ENSG0000C	221091	7,80	1,79E-25
LRRTM2	19409	leucine rich repeat trans	ENSG0000C	26045	2,40	3,09E-03
LSAMP	6705	limbic system-associate	ENSG0000C	4045	7,32	8,97E-25
LSP1	6707	lymphocyte-specific pro	ENSG0000C	4046	9,03	1,09E-47
LTC4S	6719	leukotriene C4 synthase	ENSG0000C	4056	3,81	2,14E-07
LTF	6720	lactotransferrin [Source	ENSG0000C	4057	3,96	7,36E-12
LUM	6724	lumican [Source:HGNC	ENSG0000C	4060	10,17	8,11E-51
LURAP1	32327	leucine rich adaptor pro	ENSG0000C	541468	3,75	8,34E-11
LURAP1L	31452	leucine rich adaptor pro	ENSG0000C	286343	2,94	4,10E-10
LUZP2	23206	leucine zipper protein 2	ENSG0000C	338645	4,49	1,12E-05
LY6G6C	13936	lymphocyte antigen 6 c	ENSG0000C	80740	4,68	2,35E-06
LY86	16837	lymphocyte antigen 86	ENSG0000C	9450	3,91	3,01E-08
LYPD5	26397	LY6/PLAUR domain cont	ENSG0000C	284348	2,92	2,19E-03

LYZ	6740	lysozyme [Source:HGN(	ENSG0000C	4069	6,48	3,40E-10
LZTS1	13861	leucine zipper, putative	ENSG0000C	11178	4,91	5,12E-15
MAB21L1	6757	mab-21-like 1 (C. elega	ENSG0000C	4081	5,38	1,97E-22
MACROD2	16126	MACRO domain containi	ENSG0000C	140733	4,88	5,06E-20
MAFB	6408	v-maf avian musculoap	ENSG0000C	9935	3,04	6,11E-07
MAGI2	18957	membrane associated g	ENSG0000C	9863	4,60	2,49E-27
MAGI2-AS3	40862	MAGI2 antisense RNA 3	ENSG0000C	#####	2,31	3,16E-20
MAK	6816	male germ cell-associat	ENSG0000C	4117	2,66	6,62E-08
MAL	6817	mal, T-cell differentiatio	ENSG0000C	4118	5,00	1,40E-08
MAMDC2	23673	MAM domain containing	ENSG0000C	256691	5,53	3,51E-15
MAN1C1	19080	mannosidase, alpha, cla	ENSG0000C	57134	3,98	1,42E-27
MAOB	6834	monoamine oxidase B [	ENSG0000C	4129	8,27	1,76E-39
MAP1A	6835	microtubule-associated	ENSG0000C	4130	2,72	1,87E-04
MAP3K6	6858	mitogen-activated prote	ENSG0000C	9064	2,42	1,67E-04
MAP3K8	6860	mitogen-activated prote	ENSG0000C	1326	4,58	2,26E-13
MAP4K1	6863	mitogen-activated prote	ENSG0000C	11184	3,14	5,28E-07
MAP6	6868	microtubule-associated	ENSG0000C	4135	2,01	3,05E-03
MAP7	6869	microtubule-associated	ENSG0000C	9053	3,37	8,85E-15
MAP9	26118	microtubule-associated	ENSG0000C	79884	4,50	6,84E-21
MAPK10	6872	mitogen-activated prote	ENSG0000C	5602	5,39	2,94E-12
MAPK4	6878	mitogen-activated prote	ENSG0000C	5596	7,34	7,47E-20
MAPT	6893	microtubule-associated	ENSG0000C	4137	3,61	6,97E-08
MARCO	6895	macrophage receptor w	ENSG0000C	8685	4,54	1,10E-06
MARK1	6896	MAP/microtubule affinit	ENSG0000C	4139	4,40	1,71E-17
MASP1	6901	mannan-binding lectin s	ENSG0000C	5648	6,72	4,45E-16
MATN2	6908	matrilin 2 [Source:HGN	ENSG0000C	4147	4,33	2,78E-08
MATN4	6910	matrilin 4 [Source:HGN	ENSG0000C	8785	2,38	1,30E-03
MB21D2	30438	Mab-21 domain contain	ENSG0000C	151963	2,90	8,45E-08
MBNL3	20564	muscleblind-like splicin(	ENSG0000C	55796	4,16	7,43E-10
MBOAT1	21579	membrane bound O-acy	ENSG0000C	154141	3,83	6,62E-21
MBOAT4	32311	membrane bound O-acy	ENSG0000C	619373	2,99	2,47E-05
MBP	6925	myelin basic protein [S(	ENSG0000C	4155	2,98	1,18E-14
MCF2L	14576	MCF.2 cell line derived t	ENSG0000C	23263	2,44	1,18E-07
MCHR1	4479	melanin-concentrating I	ENSG0000C	2847	4,52	7,62E-05
MCOLN3	13358	mucolipin 3 [Source:HG	ENSG0000C	55283	5,72	1,42E-19
MDFIC	28870	MyoD family inhibitor d(	ENSG0000C	29969	2,23	9,74E-06
MEFV	6998	Mediterranean fever [S(	ENSG0000C	4210	3,17	8,12E-04
MEGF11	29635	multiple EGF-like-doma	ENSG0000C	84465	2,15	4,51E-03
MEI1	28613	meiosis inhibitor 1 [Sou	ENSG0000C	150365	2,05	4,14E-05
MEIS3	29537	Meis homeobox 3 [Sour	ENSG0000C	56917	4,85	1,16E-10
MEIS3P1	7002	Meis homeobox 3 pseuc	ENSG00000179277		3,65	1,24E-11
MEOX1	7013	mesenchyme homeobo›	ENSG0000C	4222	6,80	1,34E-19
MEST	7028	mesoderm specific tran:	ENSG0000C	4232	3,73	9,58E-07
METTL24	21566	methyltransferase like 2	ENSG0000C	728464	2,01	4,84E-02
MFAP4	7035	microfibrillar-associated	ENSG0000C	4239	9,68	6,38E-70
MFSD4	25433	major facilitator superfa	ENSG0000C	148808	2,47	4,40E-05
MGAM	7043	maltase-glucoamylase (	ENSG0000C	8972	3,51	4,70E-03
MGAT3	7046	mannosyl (beta-1,4-)-g	ENSG0000C	4248	4,87	7,70E-12
MGAT4C	30871	mannosyl (alpha-1,3-)-	ENSG0000C	25834	5,56	3,78E-08
MGP	7060	matrix Gla protein [Sou	ENSG0000C	4256	4,34	2,14E-06
MIAT	33425	myocardial infarction as	ENSG0000C	440823	3,80	3,01E-04
MIDN	16298	midnolin [Source:HGNC	ENSG0000C	90007	2,27	6,85E-04
MIR100HG					4,29	3,27E-13

MIR143HG	42872	MIR143 host gene (non	ENSG00000249669	8,88	1,33E-09
MIR23A	31605	microRNA 23a [Source:	ENSG0000C	407010	3,37	8,94E-06
MIR27A	31613	microRNA 27a [Source:	ENSG0000C	407018	2,84	5,61E-05
MIR3648	38941	microRNA 3648 [Source	ENSG0000C	#####	2,70	3,36E-02
MIR3687	38946	microRNA 3687 [Source	ENSG0000C	#####	4,20	2,09E-02
MIR497HG	39523	mir-497-195 cluster ho:	ENSG0000C	406971	4,14	1,32E-18
MIR614	32870	microRNA 614 [Source:	ENSG0000C	693199	2,56	2,22E-02
MIR663A	32919	microRNA 663a [Source	ENSG0000C	284801	2,52	4,21E-02
MITF	7105	microphthalmia-associa	ENSG0000C	4286	2,98	3,81E-30
MKX	23729	mohawk homeobox [So	ENSG0000C	283078	5,68	2,70E-16
MLF1	7125	myeloid leukemia factor	ENSG0000C	4291	2,13	1,31E-07
MLPH	29643	melanophilin [Source:H	ENSG0000C	79083	6,86	3,80E-37
MLXIPL	12744	MLX interacting protein-	ENSG0000C	51085	6,82	3,77E-07
MME	7154	membrane metallo-end	ENSG0000C	4311	4,37	1,01E-05
MMP23B	7171	matrix metallopeptidase	ENSG0000C	8510	3,83	7,09E-06
MMP27	14250	matrix metallopeptidase	ENSG0000C	64066	4,65	1,80E-06
MMP9	7176	matrix metallopeptidase	ENSG0000C	4318	2,99	5,83E-03
MNDA	7183	myeloid cell nuclear diff	ENSG0000C	4332	7,49	1,54E-11
MNS1	29636	meiosis-specific nuclear	ENSG0000C	55329	2,27	2,72E-05
MOK	9833	MOK protein kinase [So	ENSG0000C	5891	2,12	5,38E-06
MOXD1	21063	monooxygenase, DBH-l	ENSG0000C	26002	3,18	1,26E-05
MPEG1	29619	macrophage expressed	ENSG0000C	219972	7,30	5,67E-26
MPL	7217	myeloproliferative leuke	ENSG0000C	4352	2,62	4,72E-09
MPP2	7220	membrane protein, palr	ENSG0000C	4355	4,34	2,88E-15
MPP3	7221	membrane protein, palr	ENSG0000C	4356	2,41	1,24E-05
MPP7	26542	membrane protein, palr	ENSG0000C	143098	4,19	5,83E-12
MPPED2	1180	metallophosphoesterase	ENSG0000C	744	2,27	1,05E-04
MPV17L	26827	MPV17 mitochondrial m	ENSG0000C	255027	3,69	1,21E-09
MPZL2	3496	myelin protein zero-like	ENSG0000C	10205	2,40	1,19E-08
MRGPRF	24828	MAS-related GPR, mem	ENSG0000C	116535	8,16	2,04E-37
MRO	24121	maestro [Source:HGNC	ENSG0000C	83876	3,11	1,79E-06
MRVI1	7237	murine retrovirus integr	ENSG0000C	10335	6,79	1,16E-21
MS4A14	30706	membrane-spanning 4-	ENSG0000C	84689	4,68	2,11E-08
MS4A2	7316	membrane-spanning 4-	ENSG0000C	2206	5,51	3,77E-07
MS4A4A	13371	membrane-spanning 4-	ENSG0000C	51338	7,90	2,33E-22
MS4A6A	13375	membrane-spanning 4-	ENSG0000C	64231	9,01	2,66E-37
MS4A7	13378	membrane-spanning 4-	ENSG0000C	58475	8,62	5,42E-43
MSC	7321	musculin [Source:HGN(	ENSG0000C	9242	6,15	3,50E-15
MSR1	7376	macrophage scavenger	ENSG0000C	4481	5,05	2,05E-13
MST4					6,77	5,84E-38
MSTN	4223	myostatin [Source:HGN	ENSG0000C	2660	3,36	1,36E-08
MSX1	7391	msh homeobox 1 [Sour	ENSG0000C	4487	3,05	3,45E-06
MSX2	7392	msh homeobox 2 [Sour	ENSG0000C	4488	3,51	6,00E-07
MT1A	7393	metallothionein 1A [Sou	ENSG0000C	4489	8,53	1,19E-06
MT1E	7397	metallothionein 1E [Sou	ENSG0000C	4493	2,83	3,73E-03
MT1F	7398	metallothionein 1F [Sou	ENSG0000C	4494	4,07	7,70E-12
MT1G	7399	metallothionein 1G [Sou	ENSG0000C	4495	5,29	6,47E-04
MT1JP	7402	metallothionein 1J, pseu	ENSG0000C	4498	5,00	1,71E-06
MT1M	14296	metallothionein 1M [Sou	ENSG0000C	4499	8,68	3,53E-08
MT1X	7405	metallothionein 1X [Sou	ENSG0000C	4501	6,44	5,40E-06
MTMR11	24307	myotubularin related pr	ENSG0000C	10903	2,28	2,35E-09
MTMR8	16825	myotubularin related pr	ENSG0000C	55613	4,39	1,08E-14
MTRNR2L1	37155	MT-RNR2-like 1 [Source	ENSG0000C	#####	7,65	1,80E-03

MTUS2	20595	microtubule associated	ENSG0000C	23281	4,23	7,37E-04
MUC1	7508	mucin 1, cell surface as	ENSG0000C	4582	3,26	5,78E-07
MUC16	15582	mucin 16, cell surface a	ENSG0000C	94025	3,09	2,08E-03
MUC20	23282	mucin 20, cell surface a	ENSG0000C	200958	5,19	2,61E-17
MUC4	7514	mucin 4, cell surface as	ENSG0000C	4585	2,08	2,39E-02
MUC5B	7516	mucin 5B, oligomeric m	ENSG0000C	727897	3,88	7,53E-07
MUC6	7517	mucin 6, oligomeric mu	ENSG0000C	4588	3,42	7,51E-05
MUM1L1	26583	melanoma associated a	ENSG0000C	139221	7,30	4,42E-19
MUSTN1	22144	musculoskeletal, embry	ENSG0000C	389125	6,51	2,27E-19
MX1	7532	myxovirus (influenza vi	ENSG0000C	4599	3,06	1,50E-15
MX2	7533	myxovirus (influenza vi	ENSG0000C	4600	4,92	2,87E-25
MXI1	7534	MAX interactor 1, dimer	ENSG0000C	4601	2,40	2,14E-13
MXRA5	7539	matrix-remodelling ass	ENSG0000C	25878	6,49	6,21E-09
MXRA8	7542	matrix-remodelling ass	ENSG0000C	54587	7,26	2,41E-51
MYC	7553	v-myc avian myelocytor	ENSG0000C	4609	2,66	8,02E-05
MYH11	7569	myosin, heavy chain 11	ENSG0000C	4629	9,26	1,94E-29
MYH3	7573	myosin, heavy chain 3,	ENSG0000C	4621	4,64	6,69E-06
MYH7B	15906	myosin, heavy chain 7B	ENSG0000C	57644	2,36	8,26E-06
MYL3	7584	myosin, light chain 3, a	ENSG0000C	4634	4,44	8,33E-10
MYL4	7585	myosin, light chain 4, a	ENSG0000C	4635	3,49	7,48E-07
MYL9	15754	myosin, light chain 9, re	ENSG0000C	10398	5,12	1,54E-08
MYLIP	21155	myosin regulatory light	ENSG0000C	29116	2,64	2,84E-08
MYLK	7590	myosin light chain kinas	ENSG0000C	4638	6,08	5,92E-23
MYLK4	27972	myosin light chain kinas	ENSG0000C	340156	2,99	2,65E-05
MYO15A	7594	myosin XVA [Source:HG	ENSG0000C	51168	2,10	1,29E-04
MYO15B	14083	myosin XVB pseudogen	ENSG0000C	80022	4,63	8,62E-29
MYO18B	18150	myosin XVIIIB [Source:	ENSG0000C	84700	5,10	1,50E-08
MYO1G	13880	myosin IG [Source:HGN	ENSG0000C	64005	3,58	4,91E-05
MYO5B	7603	myosin VB [Source:HGI	ENSG0000C	4645	2,61	7,63E-04
MYO7B	7607	myosin VIIB [Source:H	ENSG0000C	4648	5,03	7,10E-12
MYOC	7610	myocilin, trabecular me	ENSG0000C	4653	9,50	4,36E-24
MYOCD	16067	myocardin [Source:HGI	ENSG0000C	93649	6,03	4,87E-13
MYOT	12399	myotilin [Source:HGNC	ENSG0000C	9499	3,22	5,57E-10
MYOZ1	13752	myozenin 1 [Source:HG	ENSG0000C	58529	7,54	6,91E-15
MYOZ2	1330	myozenin 2 [Source:HG	ENSG0000C	51778	5,05	1,04E-15
MYT1L	7623	myelin transcription fac	ENSG0000C	23040	3,76	3,32E-04
N4BP2L1	25037	NEDD4 binding protein	ENSG0000C	90634	3,00	1,65E-08
NAALAD2	14526	N-acetylated alpha-link	ENSG0000C	10003	5,08	1,19E-16
NAALADL1	23536	N-acetylated alpha-link	ENSG0000C	10004	2,64	6,37E-15
NAALADL2	23219	N-acetylated alpha-link	ENSG0000C	254827	5,08	1,53E-24
NACAP1	24688	nascent-polypeptide-as	ENSG0000C	83955	2,91	3,46E-04
NAP1L2	7638	nucleosome assembly p	ENSG0000C	4674	2,27	1,21E-03
NAP1L3	7639	nucleosome assembly p	ENSG0000C	4675	3,15	1,23E-13
NAPSB	13396	napsin B aspartic peptic	ENSG0000C	256236	3,92	5,04E-07
NAT8L	26742	N-acetyltransferase 8-li	ENSG0000C	339983	5,21	1,37E-04
NBEA	7648	neurobeachin [Source:H	ENSG0000C	26960	2,74	2,61E-12
NBLA00301					4,12	3,48E-07
NCALD	7655	neurocalcin delta [Sour	ENSG0000C	83988	6,22	1,69E-40
NCAM1	7656	neural cell adhesion mo	ENSG0000C	4684	4,83	2,38E-13
NCAM2	7657	neural cell adhesion mo	ENSG0000C	4685	3,27	1,48E-06
NCF4	7662	neutrophil cytosolic fact	ENSG0000C	4689	7,42	1,05E-29
NCKAP1L	4862	NCK-associated protein	ENSG0000C	3071	5,97	8,34E-20
NDN	7675	necdin, melanoma antig	ENSG0000C	4692	2,52	1,37E-04

NDNF	26256	neuron-derived neurotr	ENSG0000C	79625	4,32	7,68E-09
NDP	7678	Norrie disease (pseudog	ENSG0000C	4693	4,77	2,68E-11
NDRG2	14460	NDRG family member 2	ENSG0000C	57447	4,61	1,77E-18
NDUFA4L2	29836	NADH dehydrogenase ((	ENSG0000C	56901	5,19	5,87E-18
NECAB1	20983	N-terminal EF-hand calc	ENSG0000C	64168	6,40	4,57E-35
NECAB2	23746	N-terminal EF-hand calc	ENSG0000C	54550	5,11	1,55E-08
NEGR1	17302	neuronal growth regulat	ENSG0000C	257194	5,18	1,28E-42
NELL2	7751	NEL-like 2 (chicken) [Sc	ENSG0000C	4753	3,53	5,89E-06
NENF	30384	neudesin neurotrophic f	ENSG0000C	29937	2,64	1,35E-05
NEURL1B	35422	neuralized homolog 1B	ENSG0000C	54492	6,75	3,93E-36
NEXN	29557	nexilin (F actin binding	ENSG0000C	91624	2,98	7,42E-15
NFAM1	29872	NFAT activating protein	ENSG0000C	150372	4,83	4,86E-10
NFASC	29866	neurofascin [Source:HG	ENSG0000C	23114	7,00	7,33E-22
NFATC2	7776	nuclear factor of activat	ENSG0000C	4773	2,47	5,78E-06
NFATC4	7778	nuclear factor of activat	ENSG0000C	4776	3,78	1,50E-11
NFIA	7784	nuclear factor I/A [Sour	ENSG0000C	4774	2,04	3,26E-13
NFIL3	7787	nuclear factor, interleuk	ENSG0000C	4783	4,10	5,09E-05
NFIX	7788	nuclear factor I/X (CCA/	ENSG0000C	4784	2,91	8,39E-20
NFKBIA	7797	nuclear factor of kappa	ENSG0000C	4792	2,51	1,70E-06
NFKBID	15671	nuclear factor of kappa	ENSG0000C	84807	3,44	2,09E-07
NFKBIZ	29805	nuclear factor of kappa	ENSG0000C	64332	3,70	2,73E-11
NGEF	7807	neuronal guanine nucle	ENSG0000C	25791	4,21	8,02E-06
NGF	7808	nerve growth factor (be	ENSG0000C	4803	4,48	2,13E-10
NGFR	7809	nerve growth factor rec	ENSG0000C	4804	4,26	7,56E-05
NIPSNAP3B	23641	nipsnap homolog 3B (C.	ENSG0000C	55335	3,59	1,81E-12
NKD1	17045	naked cuticle homolog :	ENSG0000C	85407	7,42	4,32E-20
NKG7	7830	natural killer cell group	ENSG0000C	4818	4,10	8,05E-10
NKX3-1	7838	NK3 homeobox 1 [Sour	ENSG0000C	4824	2,98	1,17E-04
NLGN1	14291	neuroligin 1 [Source:HG	ENSG0000C	22871	4,22	2,55E-11
NLGN3	14289	neuroligin 3 [Source:HG	ENSG0000C	54413	3,25	1,19E-07
NLRP3	16400	NLR family, pyrin domai	ENSG0000C	114548	6,62	3,56E-13
NLRP9	22941	NLR family, pyrin domai	ENSG0000C	338321	2,83	3,21E-06
NMNAT2	16789	nicotinamide nucleotide	ENSG0000C	23057	4,54	8,09E-14
NMNAT3	20989	nicotinamide nucleotide	ENSG0000C	349565	3,79	5,22E-17
NMUR1	4518	neuromedin U receptor	ENSG0000C	10316	5,69	1,74E-18
NOSTRIN	20203	nitric oxide synthase tra	ENSG0000C	115677	2,50	7,21E-05
NOTCH2	7882	notch 2 [Source:HGNC	ENSG0000C	4853	3,28	1,41E-07
NOTCH3	7883	notch 3 [Source:HGNC	ENSG0000C	4854	5,65	1,50E-11
NOV	7885	nephroblastoma overex	ENSG0000C	4856	8,29	1,73E-13
NOVA1	7886	neuro-oncological ventr	ENSG0000C	4857	8,05	3,79E-33
NPAS3	19311	neuronal PAS domain pr	ENSG0000C	64067	3,44	9,31E-08
NPAS4	18983	neuronal PAS domain pr	ENSG0000C	266743	5,35	1,01E-11
NPNT	27405	nephronectin [Source:H	ENSG0000C	255743	2,25	8,79E-03
NPPC	7941	natriuretic peptide C [S	ENSG0000C	4880	5,34	5,30E-08
NPR3	7945	natriuretic peptide rece	ENSG0000C	4883	4,26	4,39E-09
NPTX1	7952	neuronal pentraxin I [S	ENSG0000C	4884	3,22	1,98E-03
NPY1R	7956	neuropeptide Y receptor	ENSG0000C	4886	9,26	6,14E-47
NPY5R	7958	neuropeptide Y receptor	ENSG0000C	4889	6,39	5,53E-26
NR4A1	7980	nuclear receptor subfan	ENSG0000C	3164	7,02	1,40E-13
NR4A2	7981	nuclear receptor subfan	ENSG0000C	4929	7,43	3,86E-17
NR4A3	7982	nuclear receptor subfan	ENSG0000C	8013	6,28	3,94E-06
NRK	25391	Nik related kinase [Sou	ENSG0000C	203447	5,37	1,32E-16
NRXN1	8008	neurexin 1 [Source:HGI	ENSG0000C	9378	6,27	5,26E-21

NRXN2	8009	neurexin 2 [Source:HGI	ENSG0000C	9379	5,54	8,03E-17
NRXN3	8010	neurexin 3 [Source:HGI	ENSG0000C	9369	3,69	1,21E-07
NSUN7	25857	NOP2/Sun domain famil	ENSG0000C	79730	6,57	1,76E-31
NTF3	8023	neurotrophin 3 [Source	ENSG0000C	4908	5,74	3,17E-15
NTM	17941	neurotrimin [Source:HG	ENSG0000C	50863	6,52	1,10E-21
NTN1	8029	netrin 1 [Source:HGNC	ENSG0000C	9423	6,39	7,15E-20
NTNG1	23319	netrin G1 [Source:HGN(	ENSG0000C	22854	5,22	1,57E-09
NTNG2	14288	netrin G2 [Source:HGN(	ENSG0000C	84628	2,16	3,51E-04
NTRK2	8032	neurotrophic tyrosine ki	ENSG0000C	4915	7,04	6,65E-15
NTRK3	8033	neurotrophic tyrosine ki	ENSG0000C	4916	8,57	3,63E-28
NTS	8038	neurotensin [Source:HG	ENSG0000C	4922	6,78	9,52E-12
NUDT10	17621	nudix (nucleoside dipho	ENSG0000C	170685	4,65	3,13E-12
NUDT11	18011	nudix (nucleoside dipho	ENSG0000C	55190	4,25	1,67E-14
NUP210L	29915	nucleoporin 210kDa-like	ENSG0000C	91181	4,13	1,25E-04
NUPR1	29990	nuclear protein, transcr	ENSG0000C	26471	2,81	1,36E-03
NXPH3	8077	neurexophilin 3 [Source	ENSG0000C	11248	3,97	2,24E-09
OASL	8090	2'-5'-oligoadenylate syr	ENSG0000C	8638	2,63	6,51E-03
OBSCN	15719	obscurin, cytoskeletal c	ENSG0000C	84033	2,24	3,12E-07
OCA2	8101	oculocutaneous albinisn	ENSG0000C	4948	4,57	3,10E-09
ODF3B	34388	outer dense fiber of spe	ENSG0000C	440836	2,16	3,61E-05
ODF3L1	28735	outer dense fiber of spe	ENSG0000C	161753	4,74	4,06E-13
ODZ1					6,56	1,91E-28
ODZ4					2,11	6,43E-03
OGN	8126	osteoglycin [Source:HG	ENSG0000C	4969	9,59	8,67E-44
OLFM1	17187	olfactomedin 1 [Source:	ENSG0000C	10439	3,33	2,44E-13
OLFM2	17189	olfactomedin 2 [Source:	ENSG0000C	93145	2,28	5,67E-05
OLFM4	17190	olfactomedin 4 [Source:	ENSG0000C	10562	4,70	1,87E-06
OLFML1	24473	olfactomedin-like 1 [Soı	ENSG0000C	283298	8,25	6,45E-57
OLFML2B	24558	olfactomedin-like 2B [S	ENSG0000C	25903	9,01	3,46E-60
OLFML3	24956	olfactomedin-like 3 [Soı	ENSG0000C	56944	3,24	3,86E-18
OMD	8134	osteomodulin [Source:I	ENSG0000C	4958	7,40	2,01E-16
OPCML	8143	opioid binding protein/c	ENSG0000C	4978	3,93	2,32E-06
OR51E1	15194	olfactory receptor, famil	ENSG0000C	143503	2,94	1,82E-02
OSCAR	29960	osteoclast associated, ir	ENSG0000C	126014	3,24	1,65E-07
OSM	8506	oncostatin M [Source:H	ENSG0000C	5008	4,89	4,50E-08
OSR1	8111	odd-skipped related 1 (	ENSG0000C	130497	4,93	3,93E-14
OSR2	15830	odd-skipped related 2 (	ENSG0000C	116039	9,54	1,33E-55
OSTBETA					4,77	1,17E-14
OTC	8512	ornithine carbamoyltrar	ENSG0000C	5009	6,55	1,27E-17
OTUD1	27346	OTU domain containing	ENSG0000C	220213	2,09	3,20E-08
OXER1	24884	oxoeicosanoid (OXE) re	ENSG0000C	165140	3,98	3,78E-07
OXGR1	4531	oxoglutarate (alpha-ket	ENSG0000C	27199	7,41	6,09E-17
P2RX1	8533	purinergic receptor P2X	ENSG0000C	5023	7,68	4,15E-13
P2RX6	8538	purinergic receptor P2X	ENSG0000C	9127	3,94	7,58E-06
P2RY12	18124	purinergic receptor P2Y,	ENSG0000C	64805	5,54	2,83E-13
P2RY13	4537	purinergic receptor P2Y,	ENSG0000C	53829	5,94	1,20E-14
P2RY14	16442	purinergic receptor P2Y,	ENSG0000C	9934	7,20	8,20E-18
PABPC4L	31955	poly(A) binding protein,	ENSG0000C	132430	2,57	1,16E-06
PABPC5	13629	poly(A) binding protein,	ENSG0000C	140886	3,64	2,37E-10
PAG1	30043	phosphoprotein associa	ENSG0000C	55824	2,77	4,25E-08
PAK1	8590	p21 protein (Cdc42/Rac	ENSG0000C	5058	6,85	1,49E-58
PAK3	8592	p21 protein (Cdc42/Rac	ENSG0000C	5063	3,05	6,94E-09
PALLD	17068	palladin, cytoskeletal as	ENSG0000C	23022	3,34	1,25E-15

PALM2	15845	paralemmin 2 [Source:I	ENSG0000C	114299	2,44	1,40E-03
PAMR1	24554	peptidase domain conta	ENSG0000C	25891	5,58	6,16E-07
PAPPA	8602	pregnancy-associated p	ENSG0000C	5069	3,15	6,51E-04
PAPPA2	14615	pappalysin 2 [Source:H	ENSG0000C	60676	2,82	1,32E-02
PAQR5	29645	progestin and adipoQ re	ENSG0000C	54852	2,33	2,31E-02
PARD6B	16245	par-6 partitioning defec	ENSG0000C	84612	3,57	5,19E-06
PARK2	8607	parkinson protein 2, E3	ENSG0000C	5071	3,19	5,95E-10
PARM1	24536	prostate androgen-regu	ENSG0000C	25849	8,54	8,91E-42
PARP8	26124	poly (ADP-ribose) polyn	ENSG0000C	79668	3,35	1,35E-12
PART1	17263	prostate androgen-regu	ENSG0000C	25859	6,90	4,23E-13
PARVG	14654	parvin, gamma [Source	ENSG0000C	64098	3,95	6,37E-08
PBX1	8632	pre-B-cell leukemia hon	ENSG0000C	5087	2,31	8,23E-07
PCDH10	13404	protocadherin 10 [Sour	ENSG0000C	57575	3,98	2,31E-04
PCDH15	14674	protocadherin-related 1	ENSG0000C	65217	3,84	2,67E-03
PCDH18	14268	protocadherin 18 [Sour	ENSG0000C	54510	8,68	5,79E-23
PCDH19	14270	protocadherin 19 [Sour	ENSG0000C	57526	4,66	3,30E-09
PCDH20	14257	protocadherin 20 [Sour	ENSG0000C	64881	6,59	7,76E-16
PCDH7	8659	protocadherin 7 [Source	ENSG0000C	5099	2,19	4,10E-03
PCDHB18	14548	protocadherin beta 18 p	ENSG0000C	54660	2,05	7,58E-05
PCDHB3	8688	protocadherin beta 3 [S	ENSG0000C	56132	2,79	2,54E-07
PCDHB4	8689	protocadherin beta 4 [S	ENSG0000C	56131	3,43	1,19E-12
PCK1	8724	phosphoenolpyruvate ca	ENSG0000C	5105	5,72	3,32E-06
PCOLCE	8738	procollagen C-endopept	ENSG0000C	5118	7,17	1,74E-30
PCOLCE2	8739	procollagen C-endopept	ENSG0000C	26577	7,39	2,96E-14
PCP4	8742	Purkinje cell protein 4 [	ENSG0000C	5121	7,74	1,80E-15
PCP4L1	20448	Purkinje cell protein 4 li	ENSG0000C	654790	5,92	2,62E-11
PCSK1N	17301	proprotein convertase s	ENSG0000C	27344	2,56	8,23E-03
PCSK2	8744	proprotein convertase s	ENSG0000C	5126	5,40	2,11E-10
PCSK5	8747	proprotein convertase s	ENSG0000C	5125	6,14	2,80E-24
PDE1A	8774	phosphodiesterase 1A, (	ENSG0000C	5136	4,57	9,31E-12
PDE1B	8775	phosphodiesterase 1B, (	ENSG0000C	5153	5,95	2,84E-24
PDE3B	8779	phosphodiesterase 3B, (	ENSG0000C	5140	5,22	3,76E-06
PDE4C	8782	phosphodiesterase 4C, (	ENSG0000C	5143	2,50	6,55E-03
PDE5A	8784	phosphodiesterase 5A, (	ENSG0000C	8654	4,05	5,34E-17
PDGFRA	8803	platelet-derived growth	ENSG0000C	5156	6,95	1,77E-32
PDGFRB	8804	platelet-derived growth	ENSG0000C	5159	7,27	3,26E-39
PDGFRL	8805	platelet-derived growth	ENSG0000C	5157	5,74	1,31E-13
PDK3	8811	pyruvate dehydrogenas	ENSG0000C	5165	3,35	5,65E-20
PDK4	8812	pyruvate dehydrogenas	ENSG0000C	5166	3,56	2,20E-04
PDLIM3	20767	PDZ and LIM domain 3	ENSG0000C	27295	3,24	7,89E-12
PDZD4	21167	PDZ domain containing	ENSG0000C	57595	4,77	7,25E-19
PDZRN3	17704	PDZ domain containing	ENSG0000C	23024	9,01	4,50E-49
PDZRN4	30552	PDZ domain containing	ENSG0000C	29951	7,85	4,14E-24
PEBP4	28319	phosphatidylethanolami	ENSG0000C	157310	5,16	1,35E-07
PEG3	8826	paternally expressed 3	ENSG0000C	5178	4,76	7,59E-24
PENK	8831	proenkephalin [Source:	ENSG0000C	5179	6,13	2,81E-06
PER1	8845	period circadian clock 1	ENSG0000C	5187	3,45	4,88E-10
PER2	8846	period circadian clock 2	ENSG0000C	8864	4,38	1,09E-16
PER3	8847	period circadian clock 3	ENSG0000C	8863	3,26	1,42E-20
PEX5L	30024	peroxisomal biogenesis	ENSG0000C	51555	2,16	4,69E-05
PFKFB1	8872	6-phosphofructo-2-kina	ENSG0000C	5207	2,05	1,50E-02
PFKFB2	8873	6-phosphofructo-2-kina	ENSG0000C	5208	2,52	3,82E-10
PGM5	8908	phosphoglucomutase 5	ENSG0000C	5239	2,08	6,05E-09

PGR	8910	progesterone receptor [	ENSG0000C	5241	7,72	2,52E-45
PHGDH	8923	phosphoglycerate dehy‹	ENSG0000C	26227	5,35	2,33E-11
PHKG1	8930	phosphorylase kinase, ç	ENSG0000C	5260	3,33	1,83E-08
PHYHD1	23396	phytanoyl-CoA dioxyger	ENSG0000C	254295	6,11	3,26E-36
PHYHIP	16865	phytanoyl-CoA 2-hydro:	ENSG0000C	9796	5,65	6,47E-28
PI15	8946	peptidase inhibitor 15 [	ENSG0000C	51050	8,51	5,30E-13
PI16	21245	peptidase inhibitor 16 [	ENSG0000C	221476	8,27	1,41E-21
PID1	26084	phosphotyrosine interac	ENSG0000C	55022	7,30	6,28E-25
PIK3AP1	30034	phosphoinositide-3-kina	ENSG0000C	118788	3,34	3,67E-06
PIK3R5	30035	phosphoinositide-3-kina	ENSG0000C	23533	6,18	6,88E-16
PIK3R6	27101	phosphoinositide-3-kina	ENSG0000C	146850	2,75	2,05E-03
PILRA	20396	paired immunoglobin-lil	ENSG0000C	29992	3,64	5,63E-10
PIM1	8986	pim-1 oncogene [Sourc	ENSG0000C	5292	6,57	1,01E-11
PION					2,76	5,67E-11
PIP5K1B	8995	phosphatidylinositol-4-p	ENSG0000C	8395	5,96	9,37E-18
PITPNM3					2,17	1,35E-02
PITX1	9004	paired-like homeodoma	ENSG0000C	5307	4,39	1,26E-06
PIWIL2	17644	piwi-like RNA-mediated	ENSG0000C	55124	3,56	2,76E-10
PIWIL4	18444	piwi-like RNA-mediated	ENSG0000C	143689	3,25	2,68E-13
PKD1L2	21715	polycystic kidney diseas	ENSG0000C	114780	6,00	4,47E-14
PKDCC	25123	protein kinase domain c	ENSG0000C	91461	3,26	4,67E-10
PKNOX2	16714	PBX/knotted 1 homeob‹	ENSG0000C	63876	4,34	2,03E-10
PKP1	9023	plakophilin 1 (ectoderm	ENSG0000C	5317	5,56	5,16E-33
PLA2G2A	9031	phospholipase A2, grou	ENSG0000C	5320	8,72	1,40E-13
PLA2G5	9038	phospholipase A2, grou	ENSG0000C	5322	2,39	7,06E-05
PLA2G6	9039	phospholipase A2, grou	ENSG0000C	8398	2,05	7,38E-12
PLAC9	19255	placenta-specific 9 [Sou	ENSG0000C	219348	6,98	1,80E-19
PLB1	30041	phospholipase B1 [Sour	ENSG0000C	151056	3,19	2,55E-10
PLBD1	26215	phospholipase B domair	ENSG0000C	79887	4,77	9,46E-09
PLCB2	9055	phospholipase C, beta 2	ENSG0000C	5330	4,39	2,62E-12
PLCB4	9059	phospholipase C, beta 4	ENSG0000C	5332	3,06	2,64E-24
PLCL1	9063	phospholipase C-like 1		ENSG0000C	5334	2,69	2,37E-08
PLCXD3	31822	phosphatidylinositol-spe	ENSG0000C	345557	7,35	1,13E-41
PLD5	26879	phospholipase D family,	ENSG0000C	200150	6,79	9,91E-19
PLEK	9070	pleckstrin [Source:HGN	ENSG0000C	5341	6,36	3,30E-15
PLEKHA4	14339	pleckstrin homology doı	ENSG0000C	57664	2,11	5,68E-09
PLEKHA6	17053	pleckstrin homology doı	ENSG0000C	22874	2,12	5,23E-04
PLEKHF1	20764	pleckstrin homology doı	ENSG0000C	79156	2,74	4,58E-07
PLEKHG3	20364	pleckstrin homology doı	ENSG0000C	26030	3,13	1,14E-09
PLEKHG6	25562	pleckstrin homology doı	ENSG0000C	55200	4,54	2,85E-10
PLEKHH1	17733	pleckstrin homology doı	ENSG0000C	57475	2,19	1,48E-04
PLEKHH2	30506	pleckstrin homology doı	ENSG0000C	130271	6,56	6,00E-50
PLIN1	9076	perilipin 1 [Source:HGN	ENSG0000C	5346	8,50	2,99E-08
PLIN4	29393	perilipin 4 [Source:HGN	ENSG0000C	729359	5,96	3,70E-08
PLIN5	33196	perilipin 5 [Source:HGN	ENSG0000C	440503	2,52	8,80E-05
PLK1S1	15865	polo-like kinase 1 subst	ENSG00000088970		2,00	5,35E-10
PLK5	27001	polo-like kinase 5 [Sour	ENSG0000C	126520	3,26	1,79E-05
PLN	9080	phospholamban [Source	ENSG0000C	5350	9,99	1,29E-57
PLP1	9086	proteolipid protein 1 [S	ENSG0000C	5354	7,65	6,30E-33
PLTP	9093	phospholipid transfer pr	ENSG0000C	5360	3,55	3,84E-09
PLVAP	13635	plasmalemma vesicle as	ENSG0000C	83483	3,84	1,46E-08
PLXDC1	20945	plexin domain containin	ENSG0000C	57125	8,28	2,03E-54
PLXDC2	21013	plexin domain containin	ENSG0000C	84898	5,28	4,21E-20

PLXNC1	9106	plexin C1 [Source:HGN(	ENSG0000C	10154	5,23	2,27E-23
PM20D1	26518	peptidase M20 domain (	ENSG0000C	148811	2,47	8,83E-06
PM20D2	21408	peptidase M20 domain (	ENSG0000C	135293	2,14	3,49E-05
PMEPA1	14107	prostate transmembran	ENSG0000C	56937	2,72	3,53E-04
PMP2	9117	peripheral myelin protei	ENSG0000C	5375	5,04	3,04E-10
PNCK	13415	pregnancy up-regulatec	ENSG0000C	139728	7,64	2,75E-27
PNMAL2	29206	paraneoplastic Ma antig	ENSG0000C	57469	4,58	2,77E-18
PNMT	9160	phenylethanolamine N-ı	ENSG0000C	5409	4,54	5,62E-07
PNPLA7	24768	patatin-like phospholipa	ENSG0000C	375775	2,37	1,62E-15
PNRC1	17278	proline-rich nuclear rec(	ENSG0000C	10957	2,88	1,78E-07
PODN	23174	podocan [Source:HGNC	ENSG0000C	127435	9,23	8,11E-49
POM121L9P	30080	POM121 transmembran	ENSG0000C	29774	4,38	9,65E-10
POPDC2	17648	popeye domain containi	ENSG0000C	64091	4,78	2,49E-12
PP2D1	28406	protein phosphatase 2C	ENSG0000C	151649	2,52	2,55E-05
PPAP2B	9229	phosphatidic acid phosp	ENSG0000C	8613	2,36	8,70E-11
PPARGC1A	9237	peroxisome proliferator·	ENSG0000C	10891	6,27	6,00E-23
PPFIA2	9246	protein tyrosine phosph	ENSG0000C	8499	6,97	1,02E-31
PPFIA4	9248	protein tyrosine phosph	ENSG0000C	8497	2,78	1,01E-06
PPFIBP2	9250	PTPRF interacting protei	ENSG0000C	8495	3,66	1,05E-15
PPIEL	33195	peptidylprolyl isomeras(	ENSG00000243970		2,42	9,68E-06
PPIL6	21557	peptidylprolyl isomeras(	ENSG0000C	285755	2,18	1,30E-04
PPL	9273	periplakin [Source:HGN	ENSG0000C	5493	4,15	6,91E-15
PPP1R12B	7619	protein phosphatase 1,	ENSG0000C	4660	5,81	8,00E-20
PPP1R15A	14375	protein phosphatase 1,	ENSG0000C	23645	3,06	1,04E-08
PPP1R15B	14951	protein phosphatase 1,	ENSG0000C	84919	2,21	2,25E-06
PPP1R1A	9286	protein phosphatase 1,	ENSG0000C	5502	8,73	5,73E-14
PPP1R1B	9287	protein phosphatase 1,	ENSG0000C	84152	5,39	1,71E-05
PPP1R3C	9293	protein phosphatase 1,	ENSG0000C	5507	4,66	4,71E-06
PPP1R3G	14945	protein phosphatase 1,	ENSG0000C	648791	2,71	6,77E-08
PPP2R2B	9305	protein phosphatase 2,	ENSG0000C	5521	5,80	6,03E-15
PPP2R2C	9306	protein phosphatase 2,	ENSG0000C	5522	2,64	6,84E-03
PPT2-EGFL8	48343	PPT2-EGFL8 readthroug	ENSG00000258388		2,03	5,10E-04
PRDM1	9346	PR domain containing 1	ENSG0000C	639	3,25	3,38E-03
PRDM16	14000	PR domain containing 1	ENSG0000C	63976	6,67	1,68E-28
PRDM6	9350	PR domain containing 6	ENSG0000C	93166	3,07	2,66E-07
PRELP	9357	proline/arginine-rich en	ENSG0000C	5549	8,70	7,92E-45
PRF1	9360	perforin 1 (pore forminç	ENSG0000C	5551	4,11	4,46E-07
PRG2	9362	proteoglycan 2, bone m	ENSG0000C	5553	3,10	5,71E-07
PRG4	9364	proteoglycan 4 [Source	ENSG0000C	10216	4,34	5,51E-06
PRICKLE2	20340	prickle homolog 2 (Dros	ENSG0000C	166336	2,97	8,55E-08
PRIMA1	18319	proline rich membrane ;	ENSG0000C	145270	6,27	4,44E-27
PRKAA2	9377	protein kinase, AMP-act	ENSG0000C	5563	3,67	2,88E-16
PRKCB	9395	protein kinase C, beta [	ENSG0000C	5579	7,27	1,90E-23
PRKCG	9402	protein kinase C, gamm	ENSG0000C	5582	2,58	3,74E-02
PRKCQ	9410	protein kinase C, theta	ENSG0000C	5588	3,28	3,59E-08
PRKG1	9414	protein kinase, cGMP-de	ENSG0000C	5592	4,70	7,87E-22
PRKG2	9416	protein kinase, cGMP-de	ENSG0000C	5593	4,97	8,82E-08
PRODH	9453	proline dehydrogenase (	ENSG0000C	5625	2,76	2,22E-05
PROK2	18455	prokineticin 2 [Source:I	ENSG0000C	60675	4,02	6,70E-05
PROM1	9454	prominin 1 [Source:HGI	ENSG0000C	8842	2,42	2,26E-02
PROM2	20685	prominin 2 [Source:HGI	ENSG0000C	150696	2,11	8,44E-04
PRPH	9461	peripherin [Source:HGN	ENSG0000C	5630	5,20	1,88E-08
PRR15	22310	proline rich 15 [Source:	ENSG0000C	222171	3,39	3,35E-04

PRR16	29654	proline rich 16 [Source:	ENSG0000C	51334	5,89	2,09E-11
PRR24	27406	proline rich 24 [Source:	ENSG0000C	255783	2,69	1,45E-10
PRRT1	13943	proline-rich transmemb	ENSG0000C	80863	2,87	1,14E-09
PRRT2	30500	proline-rich transmemb	ENSG0000C	112476	3,00	2,52E-11
PRRT4	37280	proline-rich transmemb	ENSG0000C	401399	3,76	3,46E-06
PRRX1	9142	paired related homeobo	ENSG0000C	5396	9,06	1,33E-83
PRRX2	21338	paired related homeobo	ENSG0000C	51450	7,02	4,52E-29
PRSS35	21387	protease, serine, 35 [Sc	ENSG0000C	167681	4,46	9,49E-08
PSD	9507	pleckstrin and Sec7 dor	ENSG0000C	5662	3,35	2,80E-06
PSD4	19096	pleckstrin and Sec7 dor	ENSG0000C	23550	2,01	1,20E-04
PTAFR	9582	platelet-activating facto	ENSG0000C	5724	4,17	3,93E-14
PTCH2	9586	patched 2 [Source:HGN	ENSG0000C	8643	3,62	2,00E-09
PTCHD1	26392	patched domain contair	ENSG0000C	139411	6,41	2,97E-13
PTCHD3	24776	patched domain contair	ENSG0000C	374308	2,19	3,07E-02
PTGDR	9591	prostaglandin D2 recept	ENSG0000C	5729	6,31	2,15E-22
PTGDS	9592	prostaglandin D2 synth	ENSG0000C	5730	8,03	1,19E-15
PTGER2	9594	prostaglandin E recepto	ENSG0000C	5732	5,54	3,53E-16
PTGER3	9595	prostaglandin E recepto	ENSG0000C	5733	6,44	3,93E-10
PTGER4	9596	prostaglandin E recepto	ENSG0000C	5734	2,49	1,16E-05
PTGES	9599	prostaglandin E synthas	ENSG0000C	9536	5,02	2,56E-15
PTGFR	9600	prostaglandin F recepto	ENSG0000C	5737	4,47	4,27E-10
PTGIR	9602	prostaglandin I2 (prosta	ENSG0000C	5739	5,29	3,40E-09
PTGIS	9603	prostaglandin I2 (prosta	ENSG0000C	5740	7,95	3,41E-54
PTGS2	9605	prostaglandin-endopero	ENSG0000C	5743	4,95	6,62E-11
PTH1R	9608	parathyroid hormone 1	ENSG0000C	5745	6,82	5,15E-32
PTH2R	9609	parathyroid hormone 2	ENSG0000C	5746	4,39	9,19E-09
PTHLH	9607	parathyroid hormone-lik	ENSG0000C	5744	3,61	7,15E-05
PTK2B	9612	protein tyrosine kinase	ENSG0000C	2185	2,25	8,44E-12
PTN	9630	pleiotrophin [Source:HG	ENSG0000C	5764	7,14	6,81E-28
PTPN13	9646	protein tyrosine phosph	ENSG0000C	5783	8,28	1,03E-42
PTPN22	9652	protein tyrosine phosph	ENSG0000C	26191	3,38	1,88E-04
PTPN3	9655	protein tyrosine phosph	ENSG0000C	5774	3,95	1,46E-20
PTPRC	9666	protein tyrosine phosph	ENSG0000C	5788	7,51	1,69E-19
PTPRCAP	9667	protein tyrosine phosph	ENSG0000C	5790	2,27	1,66E-03
PTPRD	9668	protein tyrosine phosph	ENSG0000C	5789	2,30	8,95E-04
PTPRO	9678	protein tyrosine phosph	ENSG0000C	5800	5,02	2,60E-11
PTPRQ	9679	protein tyrosine phosph	ENSG0000C	374462	4,00	4,59E-05
PTPRZ1	9685	protein tyrosine phosph	ENSG0000C	5803	5,12	1,32E-12
PYGM	9726	phosphorylase, glycoge	ENSG0000C	5837	7,55	3,37E-44
PYGO1	30256	pygopus homolog 1 (Dr	ENSG0000C	26108	4,11	2,19E-09
PYHIN1	28894	pyrin and HIN domain f	ENSG0000C	149628	4,84	4,13E-09
QPRT	9755	quinolinate phosphoribo	ENSG0000C	23475	6,46	5,83E-34
RAB20	18260	RAB20, member RAS or	ENSG0000C	55647	2,31	4,38E-04
RAB23	14263	RAB23, member RAS or	ENSG0000C	51715	2,58	1,27E-08
RAB27B	9767	RAB27B, member RAS (	ENSG0000C	5874	5,50	2,21E-09
RAB33A	9773	RAB33A, member RAS (	ENSG0000C	9363	5,09	1,01E-09
RAB34	16519	RAB34, member RAS or	ENSG0000C	83871	2,36	1,47E-07
RAB3IL1	9780	RAB3A interacting prote	ENSG0000C	5866	3,38	1,95E-23
RAB40A	18283	RAB40A, member RAS (	ENSG0000C	142684	3,03	1,60E-04
RAB9B	14090	RAB9B, member RAS or	ENSG0000C	51209	3,98	1,29E-14
RADIL	22226	Ras association and DIL	ENSG0000C	55698	2,70	6,02E-05
RAMP1	9843	receptor (G protein-cou	ENSG0000C	10267	8,30	3,85E-19
RANBP3L	26353	RAN binding protein 3-li	ENSG0000C	202151	4,98	6,66E-16

RAPGEF3	16629	Rap guanine nucleotide	ENSG0000C	10411	2,27	2,39E-06
RARRES1	9867	retinoic acid receptor re	ENSG0000C	5918	2,27	3,62E-04
RARRES2	9868	retinoic acid receptor re	ENSG0000C	5919	6,63	2,13E-39
RARRES3	9869	retinoic acid receptor re	ENSG0000C	5920	3,38	1,04E-12
RASD1	15828	RAS, dexamethasone-ir	ENSG0000C	51655	3,66	1,63E-04
RASD2	18229	RASD family, member 2	ENSG0000C	23551	4,93	3,75E-09
RASGEF1B	24881	RasGEF domain family,	ENSG0000C	153020	6,68	1,32E-35
RASGRP2	9879	RAS guanyl releasing pr	ENSG0000C	10235	5,77	5,15E-32
RASGRP4	18958	RAS guanyl releasing pr	ENSG0000C	115727	4,08	1,88E-12
RASL11A	23802	RAS-like, family 11, me	ENSG0000C	387496	6,27	7,63E-18
RASL11B	23804	RAS-like, family 11, me	ENSG0000C	65997	6,13	1,54E-20
RASL12	30289	RAS-like, family 12 [Sou	ENSG0000C	51285	9,25	1,83E-33
RASSF10	33984	Ras association (RalGDS	ENSG00000189431		3,74	1,70E-05
RASSF5	17609	Ras association (RalGDS	ENSG0000C	83593	4,85	5,53E-15
RBFOX3	27097	RNA binding protein, fo:	ENSG0000C	146713	4,33	1,14E-04
RBM11	9897	RNA binding motif prote	ENSG0000C	54033	4,80	5,60E-15
RBM24	21539	RNA binding motif prote	ENSG0000C	221662	5,59	3,60E-09
RBM47	30358	RNA binding motif prote	ENSG0000C	54502	5,93	1,14E-14
RBMS3	13427	RNA binding motif, sing	ENSG0000C	27303	2,35	4,65E-21
RBP4	9922	retinol binding protein 4	ENSG0000C	5950	5,83	6,07E-07
RBP7	30316	retinol binding protein 7	ENSG0000C	116362	5,54	1,93E-31
RBPMS2	19098	RNA binding protein wit	ENSG0000C	348093	3,72	1,81E-08
RCAN1	3040	regulator of calcineurin	ENSG0000C	1827	2,20	1,70E-03
RCAN2	3041	regulator of calcineurin	ENSG0000C	10231	6,53	4,40E-26
RCN3	21145	reticulocalbin 3, EF-han	ENSG0000C	57333	2,29	2,23E-07
RCOR2	27455	REST corepressor 2 [So	ENSG0000C	283248	2,18	7,40E-06
RDH5	9940	retinol dehydrogenase 5	ENSG0000C	5959	4,47	6,46E-15
REM2	20248	RAS (RAD and GEM)-lik	ENSG0000C	161253	4,28	7,38E-07
RERG	15980	RAS-like, estrogen-regu	ENSG0000C	85004	9,35	8,39E-68
RERGL	26213	RERG/RAS-like [Source	ENSG0000C	79785	6,08	1,10E-04
RFX2	9983	regulatory factor X, 2 (i	ENSG0000C	5990	2,84	8,64E-08
RFX8	37253	RFX family member 8, l	ENSG0000C	731220	2,92	7,35E-06
RGAG4	29430	retrotransposon gag do	ENSG0000C	340526	2,14	8,61E-07
RGMA	30308	RGM domain family, me	ENSG0000C	56963	7,23	1,47E-22
RGN	9989	regucalcin [Source:HGN	ENSG0000C	9104	2,96	8,09E-05
RGS1	9991	regulator of G-protein s	ENSG0000C	5996	8,03	4,15E-16
RGS10	9992	regulator of G-protein s	ENSG0000C	6001	2,79	3,97E-12
RGS13	9995	regulator of G-protein s	ENSG0000C	6003	5,66	6,41E-08
RGS16	9997	regulator of G-protein s	ENSG0000C	6004	7,32	1,30E-25
RGS18	14261	regulator of G-protein s	ENSG0000C	64407	5,08	5,64E-09
RGS2	9998	regulator of G-protein s	ENSG0000C	5997	4,60	1,49E-09
RGS22	24499	regulator of G-protein s	ENSG0000C	26166	5,21	1,30E-16
RGS6	10002	regulator of G-protein s	ENSG0000C	9628	5,35	2,73E-08
RGS9	10004	regulator of G-protein s	ENSG0000C	8787	3,54	1,23E-05
RHBDL3	16502	rhomboid, veinlet-like 3	ENSG0000C	162494	3,55	7,04E-08
RHOH	686	ras homolog family mer	ENSG0000C	399	3,02	2,77E-04
RHOU	17794	ras homolog family mer	ENSG0000C	58480	2,60	8,89E-08
RIC3	30338	resistance to inhibitors	ENSG0000C	79608	5,08	3,45E-14
RIMBP2	30339	RIMS binding protein 2	ENSG0000C	23504	5,18	1,34E-08
RIMS3	21292	regulating synaptic mer	ENSG0000C	9783	2,87	1,48E-07
RIMS4	16183	regulating synaptic mer	ENSG0000C	140730	6,30	5,92E-09
RIPK3	10021	receptor-interacting ser	ENSG0000C	11035	2,74	7,50E-09
RIPPLY2	21390	ripply2 homolog (zebraf	ENSG0000C	134701	3,16	1,04E-07

RMRP	10031	RNA component of mito	ENSG0000C	6023	3,25	6,52E-04
RNASE4	10047	ribonuclease, RNase A f	ENSG0000C	6038	2,67	1,51E-18
RNASE6	10048	ribonuclease, RNase A f	ENSG0000C	6039	5,64	5,68E-16
RNASEL	10050	ribonuclease L (2',5'-oli	ENSG0000C	6041	2,80	1,02E-13
RND1	18314	Rho family GTPase 1 [S	ENSG0000C	27289	3,39	3,32E-03
RND2	18315	Rho family GTPase 2 [S	ENSG0000C	8153	2,25	1,94E-05
RNF112	12968	ring finger protein 112 |	ENSG0000C	7732	4,70	1,87E-22
RNF128	21153	ring finger protein 128,	ENSG0000C	79589	3,55	4,00E-04
RNF138P1	30342	ring finger protein 138,	ENSG0000C	379013	2,75	2,93E-05
RNF150	23138	ring finger protein 150 |	ENSG0000C	57484	2,76	9,23E-06
RNF165	31696	ring finger protein 165 |	ENSG0000C	494470	4,70	2,39E-10
RNF175	27735	ring finger protein 175 |	ENSG0000C	285533	2,81	1,40E-03
RNF180	27752	ring finger protein 180 |	ENSG0000C	285671	4,77	3,06E-27
RNF212	27729	ring finger protein 212 |	ENSG0000C	285498	3,14	3,06E-08
RNLS	25641	renalase, FAD-depender	ENSG0000C	55328	3,72	4,90E-14
ROBO1	10249	roundabout, axon guida	ENSG0000C	6091	2,90	9,09E-06
ROR1	10256	receptor tyrosine kinase	ENSG0000C	4919	3,36	9,66E-09
ROR2	10257	receptor tyrosine kinase	ENSG0000C	4920	7,06	1,86E-27
RORA	10258	RAR-related orphan rec	ENSG0000C	6095	2,01	1,72E-06
RPE65	10294	retinal pigment epitheli	ENSG0000C	6121	5,08	8,90E-12
RPL21P44	33820	ribosomal protein L21 p	ENSG0000C	402176	2,89	9,13E-03
RPPH1	19273	ribonuclease P RNA com	ENSG0000C	85495	2,15	2,87E-02
RPRM	24201	reprimo, TP53 depende	ENSG0000C	56475	5,00	2,47E-05
RPS16P5	36183	ribosomal protein S16 p	ENSG0000C	647190	2,93	4,06E-03
RPS27	10416	ribosomal protein S27 [	ENSG0000C	6232	4,18	4,23E-17
RRAD	10446	Ras-related associated \	ENSG0000C	6236	5,05	9,81E-20
RRAGD	19903	Ras-related GTP binding	ENSG0000C	58528	3,03	2,12E-08
RSAD2	30908	radical S-adenosyl meth	ENSG0000C	91543	4,56	2,15E-19
RSPO3	20866	R-spondin 3 [Source:HC	ENSG0000C	84870	8,63	1,05E-28
RTN1	10467	reticulon 1 [Source:HGI	ENSG0000C	6252	7,07	9,75E-31
RUNDC3B	30286	RUN domain containing	ENSG0000C	154661	3,54	4,10E-10
RUNX1	10471	runt-related transcriptic	ENSG0000C	861	2,94	9,94E-05
RUNX2	10472	runt-related transcriptic	ENSG0000C	860	5,26	4,92E-13
RUNX3	10473	runt-related transcriptic	ENSG0000C	864	4,28	1,39E-07
RXFP1	19718	relaxin/insulin-like fami	ENSG0000C	59350	3,94	3,24E-05
RXRG	10479	retinoid X receptor, gam	ENSG0000C	6258	6,07	3,12E-21
RYR1	10483	ryanodine receptor 1 (s	ENSG0000C	6261	4,31	1,35E-10
RYR2	10484	ryanodine receptor 2 (c	ENSG0000C	6262	7,68	2,71E-23
RYR3	10485	ryanodine receptor 3 [S	ENSG0000C	6263	4,52	8,31E-17
S100A12	10489	S100 calcium binding pı	ENSG0000C	6283	5,51	5,70E-06
S100A4	10494	S100 calcium binding pı	ENSG0000C	6275	6,02	5,28E-16
S100A8	10498	S100 calcium binding pı	ENSG0000C	6279	8,30	1,27E-08
S100A9	10499	S100 calcium binding pı	ENSG0000C	6280	4,73	1,93E-04
S100B	10500	S100 calcium binding pı	ENSG0000C	6285	6,99	2,71E-13
S100P	10504	S100 calcium binding pı	ENSG0000C	6286	4,53	6,60E-06
S1PR2	3169	sphingosine-1-phosphat	ENSG0000C	9294	2,71	5,89E-06
S1PR3	3167	sphingosine-1-phosphat	ENSG0000C	1903	2,30	3,13E-03
SAA1	10513	serum amyloid A1 [Sou	ENSG0000C	6288	5,30	2,14E-05
SALL2	10526	sal-like 2 (Drosophila) [	ENSG0000C	6297	2,75	7,68E-08
SAMD11	28706	sterile alpha motif dom	ENSG0000C	148398	5,09	1,26E-07
SAMD12	31750	sterile alpha motif dom	ENSG0000C	401474	2,64	1,92E-10
SAMHD1	15925	SAM domain and HD do	ENSG0000C	25939	2,09	6,92E-05
SAP25	41908	Sin3A-associated protei	ENSG0000C	#####	2,44	2,57E-05

SARDH	10536	sarcosine dehydrogenas	ENSG0000C	1757	2,86	5,29E-06
SASH3	15975	SAM and SH3 domain c	ENSG0000C	54440	4,74	2,14E-12
SATB1	10541	SATB homeobox 1 [Sou	ENSG0000C	6304	2,37	1,71E-18
SCARA3	19000	scavenger receptor clas	ENSG0000C	51435	3,07	2,67E-07
SCARA5	28701	scavenger receptor clas	ENSG0000C	286133	9,27	2,68E-30
SCARNA10	32567	small Cajal body-specifi	ENSG0000C	692148	6,71	9,99E-15
SCARNA17	32574	small Cajal body-specifi	ENSG0000C	677769	4,53	7,31E-09
SCARNA2	32558	small Cajal body-specifi	ENSG0000C	677766	5,43	2,26E-09
SCARNA5	32561	small Cajal body-specifi	ENSG0000C	677775	5,31	1,74E-08
SCARNA6	32562	small Cajal body-specifi	ENSG0000C	677772	3,81	6,32E-06
SCARNA7	32563	small Cajal body-specifi	ENSG0000C	677767	3,62	1,83E-05
SCG3	13707	secretogranin III [Sourc	ENSG0000C	29106	3,26	9,91E-08
SCN11A	10583	sodium channel, voltage	ENSG0000C	11280	3,20	3,80E-05
SCN2B	10589	sodium channel, voltage	ENSG0000C	6327	4,24	2,51E-12
SCN3B	20665	sodium channel, voltage	ENSG0000C	55800	2,42	1,94E-08
SCN4A	10591	sodium channel, voltage	ENSG0000C	6329	5,51	1,35E-09
SCN4B	10592	sodium channel, voltage	ENSG0000C	6330	4,67	6,24E-09
SCN7A	10594	sodium channel, voltage	ENSG0000C	6332	7,04	8,08E-23
SCN9A	10597	sodium channel, voltage	ENSG0000C	6335	4,22	9,48E-09
SCNN1A	10599	sodium channel, non-vc	ENSG0000C	6337	4,13	1,06E-10
SCRG1	17036	stimulator of chondroge	ENSG0000C	11341	8,47	5,83E-28
SCUBE2	30425	signal peptide, CUB don	ENSG0000C	57758	5,91	2,30E-40
SCUBE3	13655	signal peptide, CUB don	ENSG0000C	222663	3,08	3,34E-08
SDC2	10659	syndecan 2 [Source:HG	ENSG0000C	6383	2,08	1,21E-04
SDK1	19307	sidekick cell adhesion m	ENSG0000C	221935	3,77	1,46E-11
SEC14L5	29032	SEC14-like 5 (S. cerevis	ENSG0000C	9717	3,14	1,03E-07
SEC16B	30301	SEC16 homolog B (S. ce	ENSG0000C	89866	2,47	1,73E-13
SECTM1	10707	secreted and transmem	ENSG0000C	6398	4,16	1,31E-12
SEL1L2	15897	sel-1 suppressor of lin-1	ENSG0000C	80343	4,33	1,49E-08
SELENBP1	10719	selenium binding protei	ENSG0000C	8991	6,90	9,01E-32
SELM					2,44	4,97E-04
SEMA3B	10724	sema domain, immunog	ENSG0000C	7869	5,61	1,63E-29
SEMA3C	10725	sema domain, immunog	ENSG0000C	10512	4,71	1,94E-09
SEMA3E	10727	sema domain, immunog	ENSG0000C	9723	5,63	1,35E-19
SEMA4A	10729	sema domain, immunog	ENSG0000C	64218	4,16	7,18E-05
SEMA4D	10732	sema domain, immunog	ENSG0000C	10507	2,95	1,24E-17
SEMA5A	10736	sema domain, seven th	ENSG0000C	9037	3,96	3,16E-05
SEMA5B	10737	sema domain, seven th	ENSG0000C	54437	4,44	3,19E-10
SEMA6A	10738	sema domain, transmer	ENSG0000C	57556	3,33	7,49E-10
SEPP1	10751	selenoprotein P, plasma	ENSG0000C	6414	5,11	2,17E-20
SEPT1	2879	septin 1 [Source:HGNC	ENSG0000C	1731	2,73	9,61E-07
SEPT4	9165	septin 4 [Source:HGNC	ENSG0000C	5414	4,46	2,88E-21
SEPT7P2	32339	septin 7 pseudogene 2		ENSG0000C	641977	2,69	9,06E-10
SERP2	20607	stress-associated endop	ENSG0000C	387923	2,48	1,56E-03
SERPINA1	8941	serpin peptidase inhibit	ENSG0000C	5265	6,28	7,78E-09
SERPINA3	16	serpin peptidase inhibit	ENSG0000C	12	7,58	3,69E-08
SERPINA5	8723	serpin peptidase inhibit	ENSG0000C	5104	5,16	3,73E-12
SERPINB1	3311	serpin peptidase inhibit	ENSG0000C	1992	3,04	3,90E-09
SERPINF1	8824	serpin peptidase inhibit	ENSG0000C	5176	8,70	4,16E-48
SERPINF2	9075	serpin peptidase inhibit	ENSG0000C	5345	3,71	1,15E-04
SERPING1	1228	serpin peptidase inhibit	ENSG0000C	710	9,83	1,40E-58
SERTAD1	17932	SERTA domain containir	ENSG0000C	29950	2,55	2,06E-04
SERTAD4	25236	SERTA domain containir	ENSG0000C	56256	3,12	3,42E-05

SETBP1	15573	SET binding protein 1 [S	ENSG0000C	26040	3,84	2,45E-10
SFN	10773	stratifin [Source:HGNC	ENSG0000C	2810	2,30	2,18E-02
SFRP1	10776	secreted frizzled-relatec	ENSG0000C	6422	3,86	4,05E-15
SFRP2	10777	secreted frizzled-relatec	ENSG0000C	6423	9,72	6,97E-15
SFRP4	10778	secreted frizzled-relatec	ENSG0000C	6424	8,31	1,92E-18
SGCA	10805	sarcoglycan, alpha (50k	ENSG0000C	6442	9,06	2,77E-24
SGCD	10807	sarcoglycan, delta (35kl	ENSG0000C	6444	5,76	1,28E-19
SGCG	10809	sarcoglycan, gamma (3	ENSG0000C	6445	5,04	3,41E-13
SGK110					2,03	2,42E-02
SGK2	13900	serum/glucocorticoid re	ENSG0000C	10110	3,66	2,85E-07
SGSM1	29410	small G protein signalin	ENSG0000C	129049	2,76	1,71E-12
SH3BGR	10822	SH3 domain binding glu	ENSG0000C	6450	3,25	1,77E-07
SH3RF2	26299	SH3 domain containing	ENSG0000C	153769	2,64	1,35E-03
SHANK2	14295	SH3 and multiple ankyr	ENSG0000C	22941	4,43	8,46E-15
SHC3	18181	SHC (Src homology 2 d	ENSG0000C	53358	4,85	1,10E-12
SHISA6	34491	shisa homolog 6 (Xenop	ENSG0000C	388336	4,16	4,57E-07
SHOX2	10854	short stature homeobox	ENSG0000C	6474	7,92	1,31E-52
SHROOM3	30422	shroom family member	ENSG0000C	57619	6,33	1,21E-09
SIGLEC1	11127	sialic acid binding Ig-lik	ENSG0000C	6614	6,68	2,88E-14
SIGLECP3					3,59	2,29E-06
SIK1	11142	salt-inducible kinase 1 [	ENSG0000C	150094	3,50	5,33E-06
SIM1	10882	single-minded homolog	ENSG0000C	6492	3,41	3,38E-03
SIM2	10883	single-minded homolog	ENSG0000C	6493	2,40	3,63E-08
SIX1	10887	SIX homeobox 1 [Sourc	ENSG0000C	6495	2,26	9,46E-05
SIX2	10888	SIX homeobox 2 [Sourc	ENSG0000C	10736	3,62	9,88E-08
SLA	10902	Src-like-adaptor [Sourc	ENSG0000C	6503	5,52	6,91E-10
SLAIN1	26387	SLAIN motif family, mer	ENSG0000C	122060	2,30	5,56E-05
SLAMF1	10903	signaling lymphocytic a	ENSG0000C	6504	3,52	6,38E-03
SLAMF8	21391	SLAM family member 8	ENSG0000C	56833	3,55	5,94E-07
SLC10A6	30603	solute carrier family 10	ENSG0000C	345274	5,35	1,51E-06
SLC11A1	10907	solute carrier family 11	ENSG0000C	6556	4,98	1,01E-08
SLC13A3	14430	solute carrier family 13	ENSG0000C	64849	2,48	2,28E-06
SLC14A1	10918	solute carrier family 14	ENSG0000C	6563	3,26	3,06E-06
SLC15A2	10921	solute carrier family 15	ENSG0000C	6565	3,41	1,02E-06
SLC15A3	18068	solute carrier family 15,	ENSG0000C	51296	2,70	1,22E-16
SLC16A12	23094	solute carrier family 16,	ENSG0000C	387700	5,22	1,21E-11
SLC16A14	26417	solute carrier family 16,	ENSG0000C	151473	2,07	1,46E-02
SLC16A2	10923	solute carrier family 16,	ENSG0000C	6567	2,98	6,30E-13
SLC16A4	10925	solute carrier family 16,	ENSG0000C	9122	4,73	1,22E-19
SLC16A8	16270	solute carrier family 16,	ENSG0000C	23539	2,04	2,32E-04
SLC17A7	16704	solute carrier family 17	ENSG0000C	57030	3,78	1,38E-16
SLC18A2	10935	solute carrier family 18	ENSG0000C	6571	2,04	3,46E-09
SLC19A2	10938	solute carrier family 19	ENSG0000C	10560	4,64	1,30E-05
SLC19A3	16266	solute carrier family 19,	ENSG0000C	80704	7,16	1,45E-07
SLC1A2	10940	solute carrier family 1 (	ENSG0000C	6506	3,54	6,03E-06
SLC1A7	10945	solute carrier family 1 (	ENSG0000C	6512	5,84	1,80E-20
SLC22A15	20301	solute carrier family 22,	ENSG0000C	55356	5,53	1,17E-16
SLC22A17	23095	solute carrier family 22,	ENSG0000C	51310	4,36	4,08E-11
SLC22A2	10966	solute carrier family 22	ENSG0000C	6582	5,20	4,07E-15
SLC22A3	10967	solute carrier family 22	ENSG0000C	6581	6,99	3,31E-37
SLC24A3	10977	solute carrier family 24	ENSG0000C	57419	6,37	4,52E-29
SLC25A18	10988	solute carrier family 25	ENSG0000C	83733	2,11	6,20E-04
SLC25A21	14411	solute carrier family 25	ENSG0000C	89874	2,07	4,08E-05

SLC25A25	20663	solute carrier family 25	ENSG0000C	114789	3,27	2,54E-08
SLC25A27	21065	solute carrier family 25,	ENSG0000C	9481	4,21	1,69E-13
SLC25A47	20115	solute carrier family 25,	ENSG0000C	283600	3,88	1,33E-05
SLC26A10	14470	solute carrier family 26,	ENSG0000C	65012	6,32	2,88E-22
SLC2A13	15956	solute carrier family 2 (	ENSG0000C	114134	2,47	2,51E-12
SLC2A3	11007	solute carrier family 2 (	ENSG0000C	6515	3,53	3,14E-06
SLC2A4	11009	solute carrier family 2 (	ENSG0000C	6517	6,69	8,20E-21
SLC2A5	11010	solute carrier family 2 (	ENSG0000C	6518	4,86	6,59E-11
SLC35F1	21483	solute carrier family 35,	ENSG0000C	222553	4,76	5,32E-14
SLC35F3	23616	solute carrier family 35,	ENSG0000C	148641	5,58	2,48E-02
SLC38A11	26836	solute carrier family 38,	ENSG0000C	151258	4,63	3,98E-09
SLC38A3	18044	solute carrier family 38,	ENSG00000188338		3,63	1,68E-05
SLC39A8	20862	solute carrier family 39	ENSG0000C	64116	2,38	2,05E-04
SLC46A1	30521	solute carrier family 46	ENSG0000C	113235	2,94	5,40E-12
SLC47A1	25588	solute carrier family 47	ENSG0000C	55244	2,64	3,25E-04
SLC4A3	11029	solute carrier family 4,	ENSG0000C	6508	2,72	3,21E-05
SLC4A4	11030	solute carrier family 4,	ENSG0000C	8671	5,03	3,15E-10
SLC6A1	11042	solute carrier family 6 (	ENSG0000C	6529	6,74	5,91E-14
SLC6A9	11056	solute carrier family 6 (	ENSG0000C	6536	2,83	3,83E-08
SLC7A10	11058	solute carrier family 7 (	ENSG0000C	56301	4,10	2,44E-04
SLC7A2	11060	solute carrier family 7 (	ENSG0000C	6542	2,20	1,56E-03
SLC7A4	11062	solute carrier family 7 (	ENSG0000C	6545	6,21	3,00E-17
SLC7A5P2	24951	solute carrier family 7 (	ENSG00000258186		2,27	6,82E-03
SLC7A8	11066	solute carrier family 7 (	ENSG0000C	23428	4,19	1,13E-07
SLC8A1	11068	solute carrier family 8 (	ENSG0000C	6546	8,67	1,11E-41
SLC8A2	11069	solute carrier family 8 (	ENSG0000C	6543	3,44	2,81E-04
SLCO1C1	13819	solute carrier organic ar	ENSG0000C	53919	4,15	5,55E-11
SLCO2B1	10962	solute carrier organic ar	ENSG0000C	11309	2,14	3,50E-04
SLCO3A1	10952	solute carrier organic ar	ENSG0000C	28232	4,89	9,93E-10
SLCO4A1	10953	solute carrier organic ar	ENSG0000C	28231	4,83	8,32E-08
SLFN13	26481	schlafen family member	ENSG0000C	146857	3,13	9,50E-12
SLIT2	11086	slit homolog 2 (Drosopł	ENSG0000C	9353	7,06	1,18E-29
SLIT3	11087	slit homolog 3 (Drosopł	ENSG0000C	6586	9,24	3,55E-44
SLITRK2	13449	SLIT and NTRK-like fam	ENSG0000C	84631	5,40	7,82E-15
SLITRK4	23502	SLIT and NTRK-like fam	ENSG0000C	139065	6,83	6,56E-22
SLITRK6	23503	SLIT and NTRK-like fam	ENSG0000C	84189	4,80	2,60E-05
SLMAP	16643	sarcolemma associated	ENSG0000C	7871	3,41	2,03E-11
SLN	11089	sarcolipin [Source:HGN	ENSG0000C	6588	7,25	2,61E-07
SLPI	11092	secretory leukocyte pep	ENSG0000C	6590	4,03	3,15E-08
SMARCD3	11108	SWI/SNF related, matri	ENSG0000C	6604	2,60	3,19E-08
SMOC2	20323	SPARC related modular	ENSG0000C	64094	9,46	7,63E-76
SMTNL2	24764	smoothelin-like 2 [Sour	ENSG0000C	342527	4,39	1,71E-10
SNAP25	11132	synaptosomal-associate	ENSG0000C	6616	4,22	6,56E-11
SNAP91	14986	synaptosomal-associate	ENSG0000C	9892	2,19	9,80E-05
SNCAIP	11139	synuclein, alpha interac	ENSG0000C	9627	3,08	3,27E-10
SNORA23	32613	small nucleolar RNA, H/	ENSG0000C	677808	2,69	9,19E-04
SNORA41	32634	small nucleolar RNA, H/	ENSG0000C	619569	2,80	5,54E-08
SNORA44					2,51	1,04E-05
SNORA48	32641	small nucleolar RNA, H/	ENSG0000C	652965	3,08	4,72E-08
SNORA53	32646	small nucleolar RNA, H/	ENSG0000C	677832	2,59	1,90E-04
SNORA57	32651	small nucleolar RNA, H/	ENSG0000C	692158	2,08	5,99E-06
SNORA67	10224	small nucleolar RNA, H/	ENSG0000C	26781	2,49	9,85E-09
SNORD10	32706	small nucleolar RNA, C/	ENSG00000238917		3,38	1,35E-09

SNORD15B	16649	small nucleolar RNA, C/	ENSG0000C	114599	2,38	9,90E-04
SNORD17	32713	small nucleolar RNA, C/	ENSG0000C	692086	3,14	5,81E-05
SNORD32A	10159	small nucleolar RNA, C/	ENSG0000C	26819	2,38	8,39E-06
SNORD34	10161	small nucleolar RNA, C/	ENSG0000C	26817	2,73	8,28E-06
SNORD35A	10162	small nucleolar RNA, C/	ENSG0000C	26816	2,13	2,34E-04
SNORD50A					2,20	3,76E-05
SNORD68	32729	small nucleolar RNA, C/	ENSG00000200084		2,21	5,11E-04
SNORD89	32750	small nucleolar RNA, C/	ENSG0000C	692205	2,56	3,82E-04
SNORD97	32760	small nucleolar RNA, C/	ENSG0000C	692223	2,78	2,02E-05
SNTA1	11167	syntrophin, alpha 1 [So	ENSG0000C	6640	2,63	7,95E-09
SNTB1	11168	syntrophin, beta 1 (dys	ENSG0000C	6641	3,09	4,57E-05
SNTG2	13741	syntrophin, gamma 2 [S	ENSG0000C	54221	6,78	3,04E-28
SNX20	30390	sorting nexin 20 [Sourc	ENSG0000C	124460	2,40	1,23E-03
SOBP	29256	sine oculis binding prote	ENSG0000C	55084	4,12	9,49E-18
SOCS1	19383	suppressor of cytokine s	ENSG0000C	8651	4,66	3,23E-08
SOCS3	19391	suppressor of cytokine s	ENSG0000C	9021	5,82	1,11E-14
SOD3	11181	superoxide dismutase 3	ENSG0000C	6649	9,07	1,11E-35
SORBS1	14565	sorbin and SH3 domain	ENSG0000C	10580	6,32	4,03E-24
SORCS1	16697	sortilin-related VPS10 d	ENSG0000C	114815	4,87	1,80E-12
SORCS2	16698	sortilin-related VPS10 d	ENSG0000C	57537	4,70	1,18E-11
SORL1	11185	sortilin-related receptor,	ENSG0000C	6653	4,61	2,85E-07
SOSTDC1	21748	sclerostin domain conta	ENSG0000C	25928	4,32	2,13E-06
SOX10	11190	SRY (sex determining re	ENSG0000C	6663	3,54	7,62E-07
SOX15	11196	SRY (sex determining re	ENSG0000C	6665	2,54	1,60E-06
SOX5	11201	SRY (sex determining re	ENSG0000C	6660	4,82	2,08E-17
SOX9	11204	SRY (sex determining re	ENSG0000C	6662	5,20	2,36E-09
SP5	14529	Sp5 transcription factor	ENSG0000C	389058	4,28	3,13E-07
SPAG8	14105	sperm associated antige	ENSG0000C	26206	4,16	2,25E-12
SPARCL1	11220	SPARC-like 1 (hevin) [S	ENSG0000C	8404	8,30	1,80E-39
SPATA13	23222	spermatogenesis associ	ENSG0000C	221178	3,15	4,01E-12
SPATA6	18309	spermatogenesis associ	ENSG0000C	54558	3,37	4,39E-13
SPECC1	30615	sperm antigen with calp	ENSG0000C	92521	6,12	1,65E-37
SPEF2	26293	sperm flagellar 2 [Sourc	ENSG0000C	79925	3,15	3,51E-11
SPEG	16901	SPEG complex locus [Sc	ENSG0000C	10290	7,55	2,23E-23
SPI1	11241	spleen focus forming vir	ENSG0000C	6688	6,96	4,53E-26
SPN	11249	sialophorin [Source:HGI	ENSG0000C	6693	3,67	1,00E-05
SPON1	11252	spondin 1, extracellular	ENSG0000C	10418	3,32	3,04E-07
SPON2	11253	spondin 2, extracellular	ENSG0000C	10417	4,56	1,56E-09
SPTB	11274	spectrin, beta, erythroc	ENSG0000C	6710	4,87	1,25E-12
SPTBN4	14896	spectrin, beta, non-eryt	ENSG0000C	57731	2,80	1,30E-04
SPTLC3	16253	serine palmitoyltransfer	ENSG0000C	55304	5,62	2,39E-19
SRGAP3	19744	SLIT-ROBO Rho GTPase	ENSG0000C	9901	2,82	6,50E-08
SRL	11295	sarcalumenin [Source:H	ENSG0000C	6345	4,77	2,93E-17
SRPK3	11402	SRSF protein kinase 3 [	ENSG0000C	26576	2,66	1,05E-04
SRSF12	21220	serine/arginine-rich spli	ENSG0000C	135295	2,02	3,78E-03
SSC5D	26641	scavenger receptor cyst	ENSG0000C	284297	5,61	5,28E-23
SSPN	11322	sarcospan [Source:HGN	ENSG0000C	8082	5,64	3,89E-44
SSTR1	11330	somatostatin receptor 1	ENSG0000C	6751	4,48	1,38E-05
SSTR2	11331	somatostatin receptor 2	ENSG0000C	6752	6,05	4,60E-09
ST14	11344	suppression of tumorige	ENSG0000C	6768	4,21	7,35E-08
ST5	11350	suppression of tumorige	ENSG0000C	6764	2,90	1,51E-14
ST6GAL2	10861	ST6 beta-galactosamide	ENSG0000C	84620	5,22	1,35E-13
ST6GALNAC1	23614	ST6 (alpha-N-acetyl-ne	ENSG0000C	55808	5,53	2,09E-11

ST6GALNAC5	19342	ST6 (alpha-N-acetyl-ne	ENSG0000C	81849	4,77	7,85E-12
ST7-AS1	16000	ST7 antisense RNA 1 [S	ENSG0000C	93653	2,71	2,80E-09
ST8SIA1	10869	ST8 alpha-N-acetyl-neu	ENSG0000C	6489	5,18	4,23E-17
STAC	11353	SH3 and cysteine rich d	ENSG0000C	6769	2,11	1,82E-03
STAMBPL1	24105	STAM binding protein-lil	ENSG0000C	57559	3,81	2,16E-15
STARD9	19162	StAR-related lipid transf	ENSG0000C	57519	3,02	1,87E-12
STBD1					3,97	1,83E-09
STC1	11373	stanniocalcin 1 [Source	ENSG0000C	6781	4,40	4,02E-04
STEAP2	17885	STEAP family member 2	ENSG0000C	261729	2,20	6,53E-08
STEAP4	21923	STEAP family member 4	ENSG0000C	79689	8,70	1,14E-56
STK17B	11396	serine/threonine kinase	ENSG0000C	9262	2,63	4,15E-06
STK31	11407	serine/threonine kinase	ENSG0000C	56164	2,18	5,30E-05
STK32A	28317	serine/threonine kinase	ENSG0000C	202374	3,57	1,76E-06
STK33	14568	serine/threonine kinase	ENSG0000C	65975	2,30	3,99E-03
STMN3	15926	stathmin-like 3 [Source	ENSG0000C	50861	3,58	1,83E-12
STON1	17003	stonin 1 [Source:HGNC	ENSG0000C	11037	2,27	1,63E-11
STON2	30652	stonin 2 [Source:HGNC	ENSG0000C	85439	2,06	2,54E-03
STOX1	23508	storkhead box 1 [Sourc	ENSG0000C	219736	2,11	3,23E-03
STXBP5L	30757	syntaxin binding proteir	ENSG0000C	9515	4,08	1,05E-04
STXBP6	19666	syntaxin binding proteir	ENSG0000C	29091	4,92	5,55E-15
SUCNR1	4542	succinate receptor 1 [S	ENSG0000C	56670	3,08	3,34E-05
SULT1C2	11456	sulfotransferase family,	ENSG0000C	6819	4,59	6,28E-03
SUSD2	30667	sushi domain containing	ENSG0000C	56241	4,61	1,14E-17
SUSD4	25470	sushi domain containing	ENSG0000C	55061	3,22	3,10E-04
SV2B	16874	synaptic vesicle glycopr	ENSG0000C	9899	3,70	1,65E-09
SYCP2L	21537	synaptonemal complex	ENSG0000C	221711	4,74	3,77E-12
SYK	11491	spleen tyrosine kinase [	ENSG0000C	6850	6,53	5,91E-24
SYN2	11495	synapsin II [Source:HG	ENSG00000157152		3,02	5,85E-06
SYNDIG1	15885	synapse differentiation	ENSG0000C	79953	6,99	1,60E-16
SYNM	24466	synemin, intermediate f	ENSG0000C	23336	3,60	2,74E-09
SYNPO2	17732	synaptopodin 2 [Source	ENSG0000C	171024	9,41	8,39E-38
SYNPO2L	23532	synaptopodin 2-like [So	ENSG0000C	79933	4,20	1,10E-07
SYP	11506	synaptophysin [Source:	ENSG0000C	6855	2,93	2,98E-10
SYPL2	27638	synaptophysin-like 2 [S	ENSG0000C	284612	4,25	4,68E-14
SYT15	17167	synaptotagmin XV [Sou	ENSG0000C	83849	4,69	7,90E-08
SYT2	11510	synaptotagmin II [Sour	ENSG0000C	127833	4,13	9,90E-07
SYT7	11514	synaptotagmin VII [Sou	ENSG0000C	9066	5,30	1,99E-14
SYTL1	15584	synaptotagmin-like 1 [S	ENSG0000C	84958	3,35	2,78E-05
SYTL2	15585	synaptotagmin-like 2 [S	ENSG0000C	54843	5,38	7,67E-10
TAC3	11521	tachykinin 3 [Source:H(	ENSG0000C	6866	4,72	1,74E-07
TACC2	11523	transforming, acidic coil	ENSG0000C	10579	2,54	4,16E-06
TACR1	11526	tachykinin receptor 1 [S	ENSG0000C	6869	6,40	7,32E-15
TACR2	11527	tachykinin receptor 2 [S	ENSG0000C	6865	2,65	2,20E-05
TAF4B	11538	TAF4b RNA polymerase	ENSG0000C	6875	2,32	1,48E-04
TAGAP	15669	T-cell activation RhoGTF	ENSG0000C	117289	6,25	1,00E-14
TAGLN	11553	transgelin [Source:HGN	ENSG0000C	6876	5,03	1,56E-05
TBC1D10C	24702	TBC1 domain family, me	ENSG0000C	374403	2,31	5,39E-04
TBX15	11594	T-box 15 [Source:HGNC	ENSG0000C	6913	7,04	4,41E-35
TBX2	11597	T-box 2 [Source:HGNC	ENSG0000C	6909	6,27	6,19E-14
TBX5	11604	T-box 5 [Source:HGNC	ENSG0000C	6910	4,28	6,09E-08
TBXAS1	11609	thromboxane A synthas	ENSG0000C	6916	4,11	5,18E-12
TCEA3	11615	transcription elongation	ENSG0000C	6920	4,81	1,22E-21
TCEAL1	11616	transcription elongation	ENSG0000C	9338	3,25	2,09E-08

TCEAL2	29818	transcription elongation	ENSG0000C	140597	6,29	4,21E-12
TCEAL5	22282	transcription elongation	ENSG0000C	340543	3,26	7,51E-05
TCEAL7	28336	transcription elongation	ENSG0000C	56849	6,40	6,76E-29
TCF21	11632	transcription factor 21 [	ENSG0000C	6943	5,82	1,21E-17
TCF7	11639	transcription factor 7 (T	ENSG0000C	6932	2,49	7,16E-05
TCHH	11791	trichohyalin [Source:HG	ENSG0000C	7062	4,09	1,67E-07
TCTEX1D1	26882	Tctex1 domain containir	ENSG0000C	200132	2,20	2,91E-04
TCTEX1D4	32315	Tctex1 domain containir	ENSG0000C	343521	2,27	2,90E-04
TDRD6	21339	tudor domain containin	ENSG0000C	221400	3,39	7,97E-07
TDRD9	20122	tudor domain containin	ENSG0000C	122402	3,25	1,74E-05
TEAD3	11716	TEA domain family men	ENSG0000C	7005	5,50	2,17E-16
TEKT2	11725	tektin 2 (testicular) [So	ENSG0000C	27285	4,35	1,61E-13
TEKT3	14293	tektin 3 [Source:HGNC	ENSG0000C	64518	3,19	2,66E-04
TENC1	19737	tensin like C1 domain c	ENSG0000C	23371	2,51	1,01E-12
TESC	26065	tescalcin [Source:HGNC	ENSG0000C	54997	2,06	3,00E-03
TET1	29484	tet methylcytosine diox	ENSG0000C	80312	2,14	1,34E-05
TEX14	11737	testis expressed 14 [So	ENSG0000C	56155	4,29	6,74E-05
TF	11740	transferrin [Source:HGN	ENSG0000C	7018	3,72	1,06E-04
TGFB3	11769	transforming growth fac	ENSG0000C	7043	3,99	1,20E-12
TGFBI	11771	transforming growth fac	ENSG0000C	7045	2,52	1,20E-03
TGFBR3	11774	transforming growth fac	ENSG0000C	7049	3,90	5,12E-15
THBD	11784	thrombomodulin [Sourc	ENSG0000C	7056	3,08	2,26E-04
THBS2	11786	thrombospondin 2 [Sou	ENSG0000C	7058	7,04	6,27E-31
THBS4	11788	thrombospondin 4 [Sou	ENSG0000C	7060	8,82	2,98E-26
THNSL2	25602	threonine synthase-like	ENSG0000C	55258	7,03	4,85E-30
THRB	11799	thyroid hormone recept	ENSG0000C	7068	6,90	4,64E-74
THRSP	11800	thyroid hormone respor	ENSG0000C	7069	6,49	3,12E-06
THSD7B	29348	thrombospondin, type I	ENSG0000C	80731	4,16	1,59E-07
THY1	11801	Thy-1 cell surface antig	ENSG0000C	7070	3,56	2,29E-05
TIAM1	11805	T-cell lymphoma invasic	ENSG0000C	7074	5,80	7,57E-10
TICAM2	21354	toll-like receptor adapto	ENSG0000C	353376	2,06	2,27E-05
TIGD3	18334	tigger transposable eler	ENSG0000C	220359	3,78	1,27E-02
TIMP4	11823	TIMP metallopeptidase i	ENSG0000C	7079	2,91	3,67E-04
TLN2	15447	talin 2 [Source:HGNC S	ENSG0000C	83660	4,33	1,57E-17
TLR5	11851	toll-like receptor 5 [Sou	ENSG0000C	7100	4,49	6,12E-17
TLR7	15631	toll-like receptor 7 [Sou	ENSG0000C	51284	4,85	1,52E-10
TLR8	15632	toll-like receptor 8 [Sou	ENSG0000C	51311	4,92	3,78E-10
TMC5	22999	transmembrane channe	ENSG0000C	79838	3,92	1,07E-07
TMC8	20474	transmembrane channe	ENSG0000C	147138	2,29	2,37E-04
TMEM108	28451	transmembrane protein	ENSG0000C	66000	3,00	2,64E-03
TMEM119	27884	transmembrane protein	ENSG0000C	338773	8,24	4,34E-37
TMEM130	25429	transmembrane protein	ENSG0000C	222865	5,39	3,23E-15
TMEM132C	25436	transmembrane protein	ENSG0000C	92293	7,66	3,39E-13
TMEM14E	34386	transmembrane protein	ENSG0000C	645843	2,00	3,85E-02
TMEM158	30293	transmembrane protein	ENSG0000C	25907	2,99	7,05E-05
TMEM159	30136	transmembrane protein	ENSG0000C	57146	2,16	9,76E-04
TMEM176A	24930	transmembrane protein	ENSG0000C	55365	8,95	1,14E-49
TMEM176B	29596	transmembrane protein	ENSG0000C	28959	9,86	1,14E-49
TMEM184A	28797	transmembrane protein	ENSG0000C	202915	4,13	9,34E-05
TMEM198	33704	transmembrane protein	ENSG0000C	130612	4,30	2,24E-11
TMEM200B	33785	transmembrane protein	ENSG0000C	399474	5,47	1,39E-27
TMEM221	21943	transmembrane protein	ENSG0000C	#####	2,09	2,45E-02
TMEM30B	27254	transmembrane protein	ENSG0000C	161291	4,77	2,75E-42

TMEM37	18216	transmembrane protein	ENSG0000C	140738	3,34	2,27E-05
TMEM47	18515	transmembrane protein	ENSG0000C	83604	3,90	3,73E-16
TMEM56	26477	transmembrane protein	ENSG0000C	148534	3,85	1,73E-13
TMEM61	27296	transmembrane protein	ENSG0000C	199964	3,72	9,02E-05
TMEM71	26572	transmembrane protein	ENSG0000C	137835	5,55	2,38E-13
TMEM74	26409	transmembrane protein	ENSG0000C	157753	4,19	1,37E-12
TMEM74B	15893	transmembrane protein	ENSG0000C	55321	2,64	2,00E-06
TMEM8B	21427	transmembrane protein	ENSG0000C	51754	2,46	4,31E-07
TMOD1	11871	tropomodulin 1 [Source	ENSG0000C	7111	4,27	2,99E-22
TMSB15A	30744	thymosin beta 15a [Sou	ENSG0000C	11013	2,30	2,56E-03
TMTC1	24099	transmembrane and tet	ENSG0000C	83857	3,67	3,08E-07
TNC	5318	tenascin C [Source:HGN	ENSG0000C	3371	9,43	7,78E-22
TNF	11892	tumor necrosis factor [S	ENSG0000C	7124	4,40	5,62E-06
TNFAIP6	11898	tumor necrosis factor, a	ENSG0000C	7130	6,44	2,49E-11
TNFRSF19	11915	tumor necrosis factor re	ENSG0000C	55504	2,74	3,68E-05
TNFRSF8	11923	tumor necrosis factor re	ENSG0000C	943	4,14	1,44E-07
TNFSF13B	11929	tumor necrosis factor (l	ENSG0000C	10673	4,55	1,26E-12
TNFSF14	11930	tumor necrosis factor (l	ENSG0000C	8740	6,04	4,99E-08
TNFSF8	11938	tumor necrosis factor (l	ENSG0000C	944	4,80	4,29E-14
TNFSF9	11939	tumor necrosis factor (l	ENSG0000C	8744	2,10	2,68E-03
TNMD	17757	tenomodulin [Source:H(	ENSG0000C	64102	7,28	7,97E-24
TNNC1	11943	troponin C type 1 (slow	ENSG0000C	7134	4,28	7,58E-10
TNNC2	11944	troponin C type 2 (fast)	ENSG0000C	7125	4,56	1,44E-11
TNNT3	11950	troponin T type 3 (skele	ENSG0000C	7140	8,00	1,49E-20
TNS1	11973	tensin 1 [Source:HGNC	ENSG0000C	7145	3,32	6,39E-11
TNS4	24352	tensin 4 [Source:HGNC	ENSG0000C	84951	5,30	2,32E-08
TNXB	11976	tenascin XB [Source:HG	ENSG0000C	7148	4,71	2,64E-12
TOB1	11979	transducer of ERBB2, 1	ENSG0000C	10140	2,60	3,19E-08
TOM1L1	11983	target of myb1 (chicker	ENSG0000C	10040	2,48	1,57E-04
TOX	18988	thymocyte selection-ass	ENSG0000C	9760	2,24	1,31E-06
TPD52L1	12006	tumor protein D52-like	ENSG0000C	7164	2,95	1,38E-12
TPM2	12011	tropomyosin 2 (beta) [S	ENSG0000C	7169	6,93	6,00E-23
TPO	12015	thyroid peroxidase [Sou	ENSG0000C	7173	6,37	5,10E-10
TPPP	24164	tubulin polymerization p	ENSG0000C	11076	4,20	1,95E-08
TPPP3	24162	tubulin polymerization-p	ENSG0000C	51673	8,49	4,97E-50
TPRG1	24759	tumor protein p63 regu	ENSG0000C	285386	4,59	7,11E-09
TPSAB1	12019	tryptase alpha/beta 1 [S	ENSG0000C	7177	6,43	1,09E-09
TPSB2	14120	tryptase beta 2 (gene/p	ENSG0000C	64499	6,78	3,29E-12
TRAF5	12035	TNF receptor-associatec	ENSG0000C	7188	2,81	3,10E-11
TRDN	12261	triadin [Source:HGNC S	ENSG0000C	10345	7,96	3,94E-22
TREH	12266	trehalase (brush-border	ENSG00000118094		3,67	6,89E-05
TREM1	17760	triggering receptor expr	ENSG0000C	54210	6,31	2,54E-12
TRERF1	18273	transcriptional regulatin	ENSG0000C	55809	7,23	1,31E-52
TRHDE	30748	thyrotropin-releasing hc	ENSG0000C	29953	5,37	2,20E-12
TRIB1	16891	tribbles homolog 1 (Dro	ENSG0000C	10221	4,12	1,36E-07
TRIM58	24150	tripartite motif containir	ENSG0000C	25893	2,93	1,26E-05
TRIM7	16278	tripartite motif containir	ENSG0000C	81786	2,22	1,02E-07
TRPC1	12333	transient receptor poter	ENSG0000C	7220	2,96	4,31E-05
TRPC4	12336	transient receptor poter	ENSG0000C	7223	5,44	8,59E-15
TRPM5	14323	transient receptor poter	ENSG0000C	29850	5,84	3,30E-07
TRPS1	12340	trichorhinophalangeal s	ENSG0000C	7227	4,92	5,83E-16
TSC22D3	3051	TSC22 domain family, n	ENSG0000C	1831	4,53	9,26E-12
TSHZ2	13010	teashirt zinc finger hom	ENSG0000C	128553	3,74	2,99E-11

TSHZ3	30700	teashirt zinc finger hom	ENSG0000C	57616	2,58	2,81E-06
TSIX	12377	TSIX transcript, XIST ar	ENSG0000C	9383	4,37	3,09E-07
TSKS	30719	testis-specific serine kir	ENSG0000C	60385	3,60	9,89E-07
TSPAN33	28743	tetraspanin 33 [Source:	ENSG0000C	340348	2,37	1,38E-04
TSPAN7	11854	tetraspanin 7 [Source:H	ENSG0000C	7102	2,21	9,09E-03
TSPAN8	11855	tetraspanin 8 [Source:H	ENSG0000C	7103	8,67	3,09E-20
TSPYL2	24358	TSPY-like 2 [Source:HG	ENSG0000C	64061	2,37	1,10E-09
TTC25	25280	tetratricopeptide repeat	ENSG0000C	83538	2,57	4,56E-06
TTC39C	26595	tetratricopeptide repeat	ENSG0000C	125488	3,42	1,49E-14
TTC9	20267	tetratricopeptide repeat	ENSG0000C	23508	5,02	4,91E-11
TTLL7	26242	tubulin tyrosine ligase-l	ENSG0000C	79739	5,72	1,11E-16
TTN	12403	titin [Source:HGNC Syn	ENSG0000C	7273	2,03	1,49E-08
TTYH1	13476	tweety homolog 1 (Dros	ENSG0000C	57348	2,28	2,48E-03
TTYH2	13877	tweety homolog 2 (Dros	ENSG0000C	94015	3,66	4,91E-16
TUB	12406	tubby homolog (mouse)	ENSG0000C	7275	3,42	4,70E-13
TUBAL3	23534	tubulin, alpha-like 3 [Sc	ENSG0000C	79861	7,03	1,53E-24
TUBB2B	30829	tubulin, beta 2B class II	ENSG0000C	347733	2,35	5,55E-04
TULP2	12424	tubby like protein 2 [So	ENSG0000C	7288	3,95	7,36E-06
TUSC5	29592	tumor suppressor candi	ENSG0000C	286753	7,84	5,49E-09
TWIST1	12428	twist basic helix-loop-he	ENSG0000C	7291	6,53	5,52E-21
TWIST2	20670	twist basic helix-loop-he	ENSG0000C	117581	4,95	8,16E-13
TXNIP	16952	thioredoxin interacting p	ENSG0000C	10628	2,55	1,78E-06
TYROBP	12449	TYRO protein tyrosine k	ENSG0000C	7305	8,22	2,48E-45
UBE2QL1	37269	ubiquitin-conjugating er	ENSG0000C	134111	4,31	1,36E-09
UBXN10	26354	UBX domain protein 10	ENSG0000C	127733	3,45	6,86E-06
UCP3	12519	uncoupling protein 3 (m	ENSG0000C	7352	2,26	4,97E-07
UNC13C	23149	unc-13 homolog C (C. e	ENSG0000C	440279	4,88	1,63E-10
UNC5C	12569	unc-5 homolog C (C. ele	ENSG0000C	8633	5,45	1,30E-13
UNC5CL	21203	unc-5 homolog C (C. ele	ENSG0000C	222643	2,72	8,57E-09
USH1C	12597	Usher syndrome 1C (au	ENSG0000C	10083	5,86	9,07E-11
USP2	12618	ubiquitin specific peptid	ENSG0000C	9099	4,04	5,87E-12
USP53	29255	ubiquitin specific peptid	ENSG0000C	54532	2,48	2,29E-12
USP6	12629	ubiquitin specific peptid	ENSG0000C	9098	4,02	3,70E-04
UST	17223	uronyl-2-sulfotransferas	ENSG0000C	10090	7,85	1,40E-41
VANGL2	15511	VANGL planar cell polar	ENSG0000C	57216	6,08	1,56E-24
VASH2	25723	vasohibin 2 [Source:HG	ENSG0000C	79805	3,13	3,04E-09
VAV1	12657	vav 1 guanine nucleotid	ENSG0000C	7409	5,05	8,01E-13
VCAM1	12663	vascular cell adhesion n	ENSG0000C	7412	2,64	3,82E-03
VCAN	2464	versican [Source:HGNC	ENSG0000C	1462	6,56	4,36E-14
VDR	12679	vitamin D (1,25- dihydr	ENSG0000C	7421	2,79	1,33E-03
VEGFA	12680	vascular endothelial gro	ENSG0000C	7422	2,93	8,33E-05
VENTX	13639	VENT homeobox [Sourc	ENSG0000C	27287	4,56	5,28E-09
VGLL3	24327	vestigial like 3 (Drosopł	ENSG0000C	389136	6,48	4,55E-15
VIPR2	12695	vasoactive intestinal pe	ENSG0000C	7434	5,33	3,64E-21
VIT	12697	vitrin [Source:HGNC Sy	ENSG0000C	5212	7,39	1,35E-31
VMO1	30387	vitelline membrane oute	ENSG0000C	284013	2,39	2,51E-04
VNN1	12705	vanin 1 [Source:HGNC :	ENSG0000C	8876	3,59	2,74E-05
VNN2	12706	vanin 2 [Source:HGNC :	ENSG0000C	8875	6,34	1,01E-08
VSIG4	17032	V-set and immunoglobu	ENSG0000C	11326	8,08	2,85E-22
VSNL1	12722	visinin-like 1 [Source:H	ENSG0000C	7447	2,85	2,87E-05
VSTM4	26470	V-set and transmembra	ENSG0000C	196740	4,12	8,39E-10
VTN	12724	vitronectin [Source:HGI	ENSG0000C	7448	3,46	5,19E-08
VTRNA2-1	37054	vault RNA 2-1 [Source:I	ENSG0000C	#####	2,80	1,78E-03

VWC2	30200	von Willebrand factor C	ENSG0000C	375567	4,09	1,22E-07
WAS	12731	Wiskott-Aldrich syndron	ENSG0000C	7454	3,00	2,02E-06
WBSCR17	16347	Williams-Beuren syndro	ENSG0000C	64409	6,49	3,17E-17
WDFY3-AS2	21603	WDFY3 antisense RNA 2	ENSG0000C	404201	2,13	4,89E-06
WDFY4	29323	WDFY family member 4	ENSG0000C	57705	3,14	9,90E-09
WDR17	16661	WD repeat domain 17 [	ENSG0000C	116966	5,78	5,03E-19
WDR49	26587	WD repeat domain 49 [	ENSG0000C	151790	4,77	3,03E-11
WDR96	26684	WD repeat domain 96 [	ENSG0000C	80217	2,19	1,36E-03
WFDC1	15466	WAP four-disulfide core	ENSG0000C	58189	7,26	1,73E-19
WFDC2	15939	WAP four-disulfide core	ENSG0000C	10406	7,91	4,33E-12
WIPF3	22004	WAS/WASL interacting		ENSG0000C	644150	6,77	2,99E-20
WISP1	12769	WNT1 inducible signalin	ENSG0000C	8840	6,15	4,34E-20
WISP2	12770	WNT1 inducible signalin	ENSG0000C	8839	10,01	3,79E-34
WNK2	14542	WNK lysine deficient prc	ENSG0000C	65268	7,69	1,21E-16
WNK3	14543	WNK lysine deficient prc	ENSG0000C	65267	2,40	2,75E-05
WNT11	12776	wingless-type MMTV int	ENSG0000C	7481	6,61	2,22E-18
WNT2	12780	wingless-type MMTV int	ENSG0000C	7472	6,00	2,12E-10
WNT4	12783	wingless-type MMTV int	ENSG0000C	54361	3,91	2,10E-08
WNT5B	16265	wingless-type MMTV int	ENSG0000C	81029	4,50	2,64E-10
WNT9B	12779	wingless-type MMTV int	ENSG0000C	7484	3,83	1,51E-04
WSCD2	29117	WSC domain containing	ENSG0000C	9671	6,04	1,25E-12
XAF1	30932	XIAP associated factor 1	ENSG0000C	54739	6,24	5,98E-18
XG	12806	Xg blood group [Source	ENSG0000C	7499	8,96	5,36E-36
XIRP1	14301	xin actin-binding repeat	ENSG0000C	165904	4,09	1,50E-05
XIST	12810	X inactive specific trans	ENSG0000C	7503	9,37	2,28E-27
XK	12811	X-linked Kx blood group	ENSG0000C	7504	3,89	9,91E-10
XPNPEP2	12823	X-prolyl aminopeptidase	ENSG0000C	7512	4,53	2,40E-11
XYLT1	15516	xylosyltransferase I [So	ENSG0000C	64131	5,96	5,57E-16
YBX2	17948	Y box binding protein 2	ENSG0000C	51087	4,89	2,06E-10
YJEFN3	24785	YjeF N-terminal domain	ENSG0000C	374887	2,05	1,55E-04
YPEL4	18328	yippee-like 4 (Drosophil	ENSG0000C	219539	2,72	5,07E-06
ZAK					2,52	1,21E-12
ZBTB16	12930	zinc finger and BTB don	ENSG0000C	7704	3,33	9,35E-07
ZBTB7C	31700	zinc finger and BTB don	ENSG0000C	201501	5,81	2,30E-19
ZC3H12A	26259	zinc finger CCCH-type c	ENSG0000C	80149	2,36	1,24E-03
ZC3H12B	17407	zinc finger CCCH-type c	ENSG0000C	340554	3,82	6,96E-13
ZCCHC5	22997	zinc finger, CCHC domai	ENSG0000C	203430	3,95	6,64E-06
ZDHHC15	20342	zinc finger, DHHC-type (	ENSG0000C	158866	5,97	3,46E-36
ZDHHC8P1	26461	zinc finger, DHHC-type (	ENSG0000C	150244	2,35	2,87E-03
ZEB2	14881	zinc finger E-box bindin	ENSG0000C	9839	4,71	9,10E-40
ZFAND5	13008	zinc finger, AN1-type dc	ENSG0000C	7763	2,03	6,80E-04
ZFP2	26138	ZFP2 zinc finger protein	ENSG0000C	80108	2,08	6,98E-05
ZFP36	12862	ZFP36 ring finger protei	ENSG0000C	7538	7,47	1,36E-29
ZFP36L2	1108	ZFP36 ring finger protei	ENSG0000C	678	2,26	1,94E-10
ZIC1	12872	Zic family member 1 [S	ENSG0000C	7545	6,06	3,53E-06
ZIC4	20393	Zic family member 4 [S	ENSG0000C	84107	5,75	2,09E-05
ZIC5	20322	Zic family member 5 [S	ENSG0000C	85416	2,89	2,86E-02
ZMYND15	20997	zinc finger, MYND-type (	ENSG0000C	84225	2,97	1,93E-07
ZNF204P	12995	zinc finger protein 204,	ENSG0000C	7754	5,14	6,27E-31
ZNF208	12999	zinc finger protein 208		ENSG0000C	7757	3,86	1,85E-08
ZNF214	13006	zinc finger protein 214		ENSG0000C	7761	2,37	4,09E-03
ZNF239	13031	zinc finger protein 239		ENSG0000C	8187	2,72	4,70E-07
ZNF331	15489	zinc finger protein 331		ENSG0000C	55422	2,27	2,59E-04

ZNF34	13098	zinc finger protein 34 [S	ENSG0000C	80778	2,13	5,62E-09
ZNF365	18194	zinc finger protein 365 |	ENSG0000C	22891	2,33	6,49E-03
ZNF385A	17521	zinc finger protein 385A	ENSG0000C	25946	2,52	2,56E-03
ZNF385B	26332	zinc finger protein 385B	ENSG0000C	151126	4,02	3,11E-08
ZNF385C	33722	zinc finger protein 385C	ENSG0000C	201181	3,07	1,70E-06
ZNF385D	26191	zinc finger protein 385D	ENSG0000C	79750	2,86	8,91E-05
ZNF415	20636	zinc finger protein 415 |	ENSG0000C	55786	2,58	2,50E-04
ZNF454	21200	zinc finger protein 454 |	ENSG0000C	285676	2,25	1,00E-05
ZNF460	21628	zinc finger protein 460 |	ENSG0000C	10794	2,68	1,54E-04
ZNF483	23384	zinc finger protein 483 |	ENSG0000C	158399	2,24	2,17E-05
ZNF519	30574	zinc finger protein 519 |	ENSG0000C	162655	2,32	7,45E-06
ZNF536	29025	zinc finger protein 536 |	ENSG0000C	9745	5,45	1,36E-16
ZNF558	26422	zinc finger protein 558 |	ENSG0000C	148156	3,57	9,17E-12
ZNF641	31834	zinc finger protein 641 |	ENSG0000C	121274	2,32	2,92E-10
ZNF655	30899	zinc finger protein 655 |	ENSG0000C	79027	3,76	2,05E-30
ZNF660	26720	zinc finger protein 660 |	ENSG0000C	285349	2,61	7,23E-09
ZNF662	31930	zinc finger protein 662 |	ENSG0000C	389114	2,96	6,42E-09
ZNF676	20429	zinc finger protein 676 |	ENSG0000C	163223	3,85	1,57E-09
ZNF703	25883	zinc finger protein 703 |	ENSG0000C	80139	4,22	3,18E-09
ZNF727	22785	zinc finger protein 727 |	ENSG0000C	442319	3,95	8,48E-11
ZNF781	26745	zinc finger protein 781 |	ENSG0000C	163115	2,64	5,78E-11
ZNF833P	33819	zinc finger protein 833,	ENSG0000C	401898	2,93	1,63E-05
ZNF835	34332	zinc finger protein 835 |	ENSG0000C	90485	2,31	1,60E-03
ZNF853	21767	zinc finger protein 853 |	ENSG0000C	54753	4,05	2,10E-22
ZNF878	37246	zinc finger protein 878 |	ENSG0000C	729747	2,30	6,32E-03
ZNF90	13165	zinc finger protein 90 [S	ENSG0000C	7643	2,29	6,56E-04
ZP1	13187	zona pellucida glycoprot	ENSG0000C	22917	2,82	3,63E-04
ZSCAN4	23709	zinc finger and SCAN dc	ENSG0000C	201516	3,92	3,01E-09
