# Supplementary material for: Morphological and Molecular Characterization of Human Dermal Lymphatic Collectors
Source: PLoS One. 2016 Oct 20;11(10):e0164964. doi: 10.1371/journal.pone.0164964 (PMC5072738; doi:10.1371/journal.pone.0164964)
Supplement: S2 Table — Genes are listed alphabetically by HGNC Symbol. Log2FC: Log2 fold change; FDR: false discovery rate. Highly expressed SMC genes, which appear in both of the lists and are discussed in the manuscript, are marked in green. (PDF) [file pone.0164964.s002.pdf]

hgnc_symbol	hgnc_id	description	ensembl_gene_id	entrezgene	log2FC	FDR
ABCA10	30	ATP-binding cassette, su	ENSG00000154263	10349	6,15	2,83E-09
ABCA8	38	ATP-binding cassette, su	ENSG00000141338	10351	5,01	2,15E-08
ABCA9	39	ATP-binding cassette, su	ENSG00000154258	10350	4,15	1,63E-03
ABCB5	46	ATP-binding cassette, su	ENSG00000004846	340273	5,55	1,46E-03
ABCC8	59	ATP-binding cassette, su	ENSG00000006071	6833	7,14	3,50E-05
ABCC9	60	ATP-binding cassette, su	ENSG00000069431	10060	9,13	1,46E-20
ABCD2	66	ATP-binding cassette, su	ENSG00000173208	225	5,37	1,49E-03
ABHD1	17553	abhydrolase domain cor	ENSG00000143994	84696	2,40	1,37E-02
ABO	79	ABO blood group (trans	ENSG00000256062	28	5,57	1,56E-02
ABRA	30655	actin-binding Rho activa	ENSG00000174429	137735	6,85	5,18E-03
ACACB	85	acetyl-CoA carboxylase	ENSG00000076555	32	4,31	1,48E-05
ACADL	88	acyl-CoA dehydrogenas	ENSG00000115361	33	6,37	1,62E-07
ACAN	319	aggrecan [Source:HGN(	ENSG00000157766	176	4,14	4,95E-02
ACAT1	93	acetyl-CoA acetyltransfe	ENSG00000075239	38	2,32	4,49E-05
ACP5	124	acid phosphatase 5, tar	ENSG00000102575	54	3,42	1,41E-03
ACR	126	acrosin [Source:HGNC S	ENSG00000100312	49	2,70	3,02E-02
ACSM1	18049	acyl-CoA synthetase me	ENSG00000166743	116285	5,15	2,92E-04
ACSM5	26060	acyl-CoA synthetase me	ENSG00000183549	54988	2,50	1,17E-02
ACSS3	24723	acyl-CoA synthetase sh	ENSG00000111058	79611	5,40	8,65E-11
ACTA1	129	actin, alpha 1, skeletal	ENSG00000143632	58	5,68	4,59E-03
ACTA2	130	actin, alpha 2, smooth	ENSG00000107796	59	7,81	1,70E-08
ACTC1	143	actin, alpha, cardiac mu	ENSG00000159251	70	5,89	4,37E-04
ACTG2	145	actin, gamma 2, smootl	ENSG00000163017	72	8,86	4,38E-09
ACTN2	164	actinin, alpha 2 [Source	ENSG00000077522	88	4,85	4,82E-03
ACVR1C	18123	activin A receptor, type	ENSG00000123612	130399	6,34	1,12E-02
ADAM11	189	ADAM metallopeptidase	ENSG00000073670	4185	3,25	8,87E-04
ADAM22	201	ADAM metallopeptidase	ENSG00000008277	53616	3,44	2,04E-09
ADAM28	206	ADAM metallopeptidase	ENSG00000042980	10863	2,88	4,08E-02
ADAM32	15479	ADAM metallopeptidase	ENSG00000197140	203102	3,30	2,44E-04
ADAM33	15478	ADAM metallopeptidase	ENSG00000149451	80332	5,90	2,14E-11
ADAMTS1	217	ADAM metallopeptidase	ENSG00000154734	9510	4,11	4,36E-02
ADAMTS17	17109	ADAM metallopeptidase	ENSG00000140470	170691	4,76	3,22E-03
ADAMTS3	219	ADAM metallopeptidase	ENSG00000156140	9508	2,71	4,28E-02
ADAMTS5	221	ADAM metallopeptidase	ENSG00000154736	11096	3,34	3,19E-02
ADAMTS8	224	ADAM metallopeptidase	ENSG00000134917	11095	3,83	4,49E-02
ADAMTS9-AS2	42435	ADAMTS9 antisense RN.	ENSG00000241684	100507098	3,25	2,50E-04
ADAMTSL2	14631	ADAMTS-like 2 [Source:	ENSG00000197859	9719	5,60	1,05E-02
ADAMTSL4	19706	ADAMTS-like 4 [Source:	ENSG00000143382	54507	2,88	3,54E-06
ADAP2	16487	ArfGAP with dual PH do	ENSG00000184060	55803	3,19	5,31E-03
ADCY1	232	adenylate cyclase 1 (bra	ENSG00000164742	107	4,56	2,98E-04
ADCY2	233	adenylate cyclase 2 (bra	ENSG00000078295	108	6,78	2,11E-09
ADCY5	236	adenylate cyclase 5 [So	ENSG00000173175	111	6,56	1,82E-07
ADCYAP1R1	242	adenylate cyclase activa	ENSG00000078549	117	6,75	1,12E-05
ADH1A	249	alcohol dehydrogenase	ENSG00000187758	124	4,59	2,89E-03
ADH1B	250	alcohol dehydrogenase	ENSG00000196616	125	10,12	2,71E-12
ADH1C	251	alcohol dehydrogenase	ENSG00000248144	126	6,58	1,56E-07
ADIPOQ	13633	adiponectin, C1Q and co	ENSG00000181092	9370	9,58	3,13E-03
ADORA3	268	adenosine A3 receptor [	ENSG00000121933	140	5,61	4,65E-03
ADRA2A	281	adrenoceptor alpha 2A	ENSG00000150594	150	7,13	1,36E-04
ADRA2B	282	adrenoceptor alpha 2B	ENSG00000222040	151	7,56	2,63E-06
ADRA2C	283	adrenoceptor alpha 2C	ENSG00000184160	152	5,65	3,41E-04
ADRB2	286	adrenoceptor beta 2, su	ENSG00000169252	154	2,40	2,81E-02

ADRBK2	290	adrenergic, beta, recept	ENSG00000100077	157	3,95	1,86E-03
AEBP1	303	AE binding protein 1 [S	ENSG00000106624	165	2,67	1,38E-02
AFF3	6473	AF4/FMR2 family, meml	ENSG00000144218	3899	2,23	4,05E-02
AGAP11	29421	ankyrin repeat and GTP	ENSG00000151303		5,10	1,00E-07
AGT	333	angiotensinogen (serpir	ENSG00000135744	183	8,25	2,78E-08
AGTR1	336	angiotensin II receptor,	ENSG00000144891	185	7,30	2,30E-11
AIF1	352	allograft inflammatory f	ENSG00000206428	199	8,53	6,12E-12
AK7	20091	adenylate kinase 7 [Sou	ENSG00000140057	122481	2,35	3,59E-02
AKAP6	376	A kinase (PRKA) anchor	ENSG00000151320	9472	4,81	4,39E-02
AKNAD1	28398	AKNA domain containin	ENSG00000162641	254268	4,84	2,43E-05
AKR1C1	384	aldo-keto reductase fan	ENSG00000187134	1645	4,17	9,89E-06
AKR1C2	385	aldo-keto reductase fan	ENSG00000265231	1646	3,54	9,58E-04
ALDH1A2	15472	aldehyde dehydrogenas	ENSG00000128918	8854	3,25	7,36E-04
ALDH1B1	407	aldehyde dehydrogenas	ENSG00000137124	219	2,32	5,18E-03
ALDH1L1	3978	aldehyde dehydrogenas	ENSG00000144908	10840	5,19	1,40E-02
ALDH3A1	405	aldehyde dehydrogenas	ENSG00000108602	218	5,56	2,39E-05
ALDH3A2	403	aldehyde dehydrogenas	ENSG00000072210	224	2,67	8,70E-05
ALDH8A1	15471	aldehyde dehydrogenas	ENSG00000118514	64577	2,74	1,62E-02
ALOX5	435	arachidonate 5-lipoxyge	ENSG00000262552	240	5,76	3,24E-06
ALOX5AP	436	arachidonate 5-lipoxyge	ENSG00000132965	241	3,33	1,49E-02
ALS2CR11	14438	amyotrophic lateral scle	ENSG00000155754	151254	3,12	1,07E-02
AMD1	457	adenosylmethionine de	ENSG00000123505	262	2,18	2,57E-02
AMICA1	19084	adhesion molecule, inte	ENSG00000160593	120425	6,95	2,32E-05
AMOT	17810	angiomotin [Source:HG	ENSG00000126016	154796	2,13	1,04E-04
AMPH	471	amphiphysin [Source:H	ENSG00000078053	273	5,94	6,23E-10
AMT	473	aminomethyltransferase	ENSG00000145020	275	2,17	7,87E-05
AMY2B	478	amylase, alpha 2B (pan	ENSG00000240038	280	2,92	3,47E-05
ANAPC4	19990	anaphase promoting co	ENSG00000053900	29945	2,11	1,16E-06
ANG	483	angiogenin, ribonucleas	ENSG00000214274	283	2,25	6,14E-04
ANGPT1	484	angiopoietin 1 [Source:	ENSG00000154188	284	2,97	6,34E-03
ANGPTL1	489	angiopoietin-like 1 [Sou	ENSG00000116194	9068	8,05	1,20E-20
ANGPTL2	490	angiopoietin-like 2 [Sou	ENSG00000136859	23452	2,12	1,10E-03
ANGPTL5	19705	angiopoietin-like 5 [Sou	ENSG00000187151	253935	7,57	7,23E-07
ANGPTL7	24078	angiopoietin-like 7 [Sou	ENSG00000171819	10218	4,70	1,24E-02
ANK2	493	ankyrin 2, neuronal [So	ENSG00000145362	287	3,86	8,34E-04
ANKDD1A	28002	ankyrin repeat and deat	ENSG00000166839	348094	3,37	2,04E-07
ANKRD29	27110	ankyrin repeat domain :	ENSG00000154065	147463	2,60	3,04E-04
ANKRD30BL	35167	ankyrin repeat domain :	ENSG00000163046	554226	5,84	1,21E-02
ANKRD32	25408	ankyrin repeat domain :	ENSG00000133302	84250	2,03	9,79E-03
ANKRD35	26323	ankyrin repeat domain :	ENSG00000198483	148741	4,32	1,51E-03
ANKRD65	42950	ankyrin repeat domain (	ENSG00000235098	441869	5,32	5,06E-08
ANKS1B	24600	ankyrin repeat and ster	ENSG00000185046	56899	6,26	3,67E-08
ANO1	21625	anoctamin 1, calcium a	ENSG00000131620	55107	7,58	2,55E-08
ANO5	27337	anoctamin 5 [Source:H	ENSG00000171714	203859	6,50	1,21E-08
ANTXR1	21014	anthrax toxin receptor 1	ENSG00000169604	84168	2,80	4,11E-02
AOAH	548	acyloxyacyl hydrolase (	ENSG00000136250	313	5,48	6,27E-06
AOC3	550	amine oxidase, copper (	ENSG00000131471	8639	8,49	6,61E-15
AOC4					5,37	2,23E-04
APBB1IP	17379	amyloid beta (A4) precu	ENSG00000077420	54518	5,49	4,14E-04
APCDD1	15718	adenomatosis polyposis	ENSG00000154856	147495	4,84	1,57E-07
APOA1	600	apolipoprotein A-I [Sou	ENSG00000118137	335	4,09	4,42E-03
APOBEC3G	17357	apolipoprotein B mRNA	ENSG00000239713	60489	2,08	1,68E-02
APOD	612	apolipoprotein D [Sourc	ENSG00000189058	347	5,48	2,92E-05

APOE	613	apolipoprotein E [Sourc	ENSG00000130203	348	3,50	1,50E-02
APOLD1	25268	apolipoprotein L domair	ENSG00000178878	81575	5,15	7,05E-03
AQP1	633	aquaporin 1 (Colton blo	ENSG00000240583	358	3,94	7,50E-04
AQP10	16029	aquaporin 10 [Source:H	ENSG00000143595	89872	4,77	1,57E-03
AQP7	640	aquaporin 7 [Source:H(	ENSG00000165269	364	6,87	1,99E-03
AQP9	643	aquaporin 9 [Source:H(	ENSG00000103569	366	6,15	2,07E-02
AQPEP					5,40	3,27E-04
ARAP2	16924	ArfGAP with RhoGAP do	ENSG00000047365	116984	6,95	1,52E-04
ARC	648	activity-regulated cytos	ENSG00000198576	23237	6,27	1,95E-03
ARHGAP15	21030	Rho GTPase activating ţ	ENSG00000075884	55843	8,61	9,34E-22
ARHGAP20	18357	Rho GTPase activating ţ	ENSG00000137727	57569	3,62	1,98E-04
ARHGAP28	25509	Rho GTPase activating ţ	ENSG00000088756	79822	2,58	2,64E-04
ARHGAP30	27414	Rho GTPase activating ţ	ENSG00000186517	257106	4,51	1,17E-05
ARHGAP44	29096	Rho GTPase activating ţ	ENSG00000006740	9912	4,03	6,91E-10
ARHGAP6	676	Rho GTPase activating ţ	ENSG00000047648	395	3,74	1,58E-08
ARHGEF25	30275	Rho guanine nucleotide	ENSG00000240771	115557	5,21	2,71E-12
ARHGEF26	24490	Rho guanine nucleotide	ENSG00000114790	26084	3,59	1,04E-04
ARHGEF4	684	Rho guanine nucleotide	ENSG00000136002	50649	4,35	4,66E-04
ARHGEF5	13209	Rho guanine nucleotide	ENSG00000050327	7984	2,89	1,64E-03
ARID5A	17361	AT rich interactive doma	ENSG00000196843	10865	2,90	3,03E-02
ARID5B	17362	AT rich interactive doma	ENSG00000150347	84159	2,81	2,66E-03
ARNT2	16876	aryl-hydrocarbon recept	ENSG00000172379	9915	6,18	1,54E-07
ARRDC3	29263	arrestin domain contain	ENSG00000113369	57561	2,94	7,92E-05
ARRDC4	28087	arrestin domain contain	ENSG00000140450	91947	2,71	3,17E-02
ART4	726	ADP-ribosyltransferase ·	ENSG00000111339	420	2,20	1,82E-02
ASB2	16012	ankyrin repeat and SOC	ENSG00000100628	51676	8,64	6,99E-11
ASB5	17180	ankyrin repeat and SOC	ENSG00000164122	140458	4,61	3,75E-04
ASPA	756	aspartoacylase [Source	ENSG00000108381	443	7,46	2,64E-11
ASPG	20123	asparaginase homolog (	ENSG00000166183	374569	7,04	4,55E-06
ASPN	14872	asporin [Source:HGNC !	ENSG00000106819	54829	6,27	1,78E-10
ASPRV1	26321	aspartic peptidase, retr	ENSG00000244617	151516	2,08	4,05E-03
ASXL3	29357	additional sex combs lik	ENSG00000141431	80816	3,59	5,10E-03
ATF3	785	activating transcription	ENSG00000162772	467	4,79	1,19E-03
ATG9B	21899	autophagy related 9B [!	ENSG00000181652	285973	3,13	3,71E-02
ATL1	11231	atlastin GTPase 1 [Sour	ENSG00000198513	51062	3,16	1,26E-04
ATP10A	13542	ATPase, class V, type 1(	ENSG00000206190	57194	3,17	2,26E-02
ATP13A4	25422	ATPase type 13A4 [Sou	ENSG00000127249	84239	4,35	8,75E-03
ATP1A2	800	ATPase, Na+/K+ transp	ENSG00000018625	477	8,74	6,29E-13
ATP1B2	805	ATPase, Na+/K+ transp	ENSG00000129244	482	5,82	9,83E-08
ATP2A3	813	ATPase, Ca++ transport	ENSG00000074370	489	6,60	5,86E-05
ATP8B4	13536	ATPase, class I, type 8E	ENSG00000104043	79895	6,80	1,09E-10
ATRNL1	29063	attractin-like 1 [Source:	ENSG00000107518	26033	4,07	9,42E-04
AURKC	11391	aurora kinase C [Source	ENSG00000105146	6795	2,23	2,42E-02
AUTS2	14262	autism susceptibility ca	ENSG00000158321	26053	4,03	5,85E-06
AVIL	14188	advillin [Source:HGNC !	ENSG00000135407	10677	2,29	3,84E-02
AVPR1A	895	arginine vasopressin re	ENSG00000166148	552	6,63	4,80E-05
AVPR2	897	arginine vasopressin re	ENSG00000126895	554	4,40	1,74E-04
AZGP1	910	alpha-2-glycoprotein 1,	ENSG00000160862	563	5,76	1,15E-02
B3GAT2	922	beta-1,3-glucuronyltran	ENSG00000112309	135152	4,63	1,56E-03
BAG2	938	BCL2-associated athanc	ENSG00000112208	9532	2,36	1,09E-02
BAI1	943	brain-specific angiogene	ENSG00000181790	575	5,13	1,39E-02
BAI3	945	brain-specific angiogene	ENSG00000135298	577	3,35	2,09E-03
BAMBI	30251	BMP and activin membr	ENSG00000095739	25805	2,10	2,28E-02

BATF2	25163	basic leucine zipper trar	ENSG00000168062	116071	2,33	3,95E-02
BATF3	28915	basic leucine zipper trar	ENSG00000123685	55509	3,30	1,20E-02
BBS4	969	Bardet-Biedl syndrome	ENSG00000140463	585	2,06	1,85E-05
BCHE	983	butyrylcholinesterase [S	ENSG00000114200	590	2,87	5,37E-03
BCL2	990	B-cell CLL/lymphoma 2	ENSG00000171791	596	3,17	1,48E-04
BCL6	1001	B-cell CLL/lymphoma 6	ENSG00000113916	604	3,13	4,00E-03
BCO2	18503	beta-carotene oxygenas	ENSG00000197580	83875	2,98	2,60E-02
BEAN1	24160	brain expressed, associ	ENSG00000166546	146227	2,54	2,53E-02
BEGAIN	24163	brain-enriched guanylat	ENSG00000183092	57596	3,27	1,84E-02
BEND5	25668	BEN domain containing	ENSG00000162373	79656	4,08	2,23E-05
BEX5	27990	brain expressed, X-linke	ENSG00000184515	340542	2,58	4,04E-02
BHLHE40	1046	basic helix-loop-helix fa	ENSG00000134107	8553	2,96	1,57E-03
BIN2	1053	bridging integrator 2 [S	ENSG00000110934	51411	5,28	4,36E-04
BLNK	14211	B-cell linker [Source:HG	ENSG00000262509	29760	4,39	1,48E-03
BMP5	1072	bone morphogenetic pr	ENSG00000112175	653	5,67	1,57E-04
BMPR1A	1076	bone morphogenetic pr	ENSG00000107779	657	3,23	1,30E-07
BMPR1B	1077	bone morphogenetic pr	ENSG00000138696	658	4,47	6,35E-06
BOC	17173	BOC cell adhesion asso	ENSG00000144857	91653	5,16	3,04E-09
BRP44L					2,51	5,29E-05
BSN	1117	bassoon presynaptic cyt	ENSG00000164061	8927	2,94	1,92E-02
BSPRY	18232	B-box and SPRY domair	ENSG00000119411	54836	5,32	1,28E-03
BST2	1119	bone marrow stromal ce	ENSG00000130303	684	2,85	3,10E-06
BTC	1121	betacellulin [Source:HG	ENSG00000261530	685	3,96	5,13E-04
BTG2	1131	BTG family, member 2 [	ENSG00000159388	7832	3,04	2,08E-03
BTK	1133	Bruton agammaglobulin	ENSG00000010671	695	5,00	3,99E-04
BTN3A2	1139	butyrophilin, subfamily	ENSG00000186470	11118	4,58	6,87E-09
BTNL9	24176	butyrophilin-like 9 [Sou	ENSG00000165810	153579	8,58	1,46E-06
BVES-AS1	21223	BVES antisense RNA 1 [	ENSG00000203808	154442	3,59	1,27E-02
BZRAP1	16831	benzodiazapine recepto	ENSG00000005379	9256	6,98	1,56E-07
C10orf105	20304	chromosome 10 open re	ENSG00000214688	414152	4,49	1,99E-02
C10orf116					3,97	1,67E-04
C10orf131	31667	chromosome 10 open re	ENSG00000173088	100127889	3,65	1,08E-02
C10orf68	25779	chromosome 10 open re	ENSG00000150076	79741	2,09	1,99E-02
C10orf82	28500	chromosome 10 open re	ENSG00000262564	143379	3,98	5,20E-03
C11orf70	28188	chromosome 11 open re	ENSG00000137691	85016	2,53	3,77E-03
C11orf92	33789	chromosome 11 open re	ENSG00000196167		4,60	2,76E-04
C11orf93	26978	chromosome 11 open re	ENSG00000214290	120376	5,32	4,87E-06
C11orf96	38675	chromosome 11 open re	ENSG00000187479	387763	5,95	6,33E-04
C12orf69					2,21	4,76E-02
C14orf180	33795	chromosome 14 open re	ENSG00000184601	400258	8,09	3,84E-06
C14orf45					2,43	1,90E-05
C14orf64	20111	chromosome 14 open re	ENSG00000246223		4,22	6,81E-03
C16orf54	26649	chromosome 16 open re	ENSG00000185905	283897	4,15	7,53E-03
C16orf89	28687	chromosome 16 open re	ENSG00000153446	146556	5,51	2,78E-04
C17orf109					3,58	2,79E-03
C18orf1					2,49	9,71E-05
C19orf35	24793	chromosome 19 open re	ENSG00000188305	374872	3,41	3,92E-02
C19orf80	24933	chromosome 19 open re	ENSG00000130173	55908	3,13	1,71E-02
C1orf126					3,22	4,27E-02
C1orf140					4,44	3,74E-03
C1orf162	28344	chromosome 1 open rea	ENSG00000143110	128346	4,09	1,33E-04
C1orf186	25341	chromosome 1 open rea	ENSG00000263961	440712	5,23	1,29E-02
C1orf51	25200	chromosome 1 open rea	ENSG00000159208	148523	2,39	4,02E-02

C1orf54	26258	chromosome 1 open rea	ENSG00000118292	79630	2,91	7,51E-03
C1orf88					5,83	3,60E-06
C1orf95	30491	chromosome 1 open rea	ENSG00000203685	375057	4,48	1,54E-02
C1QA	1241	complement component	ENSG00000173372	712	8,71	1,21E-12
C1QB	1242	complement component	ENSG00000173369	713	9,39	1,46E-14
C1QC	1245	complement component	ENSG00000159189	714	8,01	9,62E-10
C1QTNF1	14324	C1q and tumor necrosis	ENSG00000173918	114897	3,80	9,19E-03
C1QTNF2	14325	C1q and tumor necrosis	ENSG00000145861	114898	4,72	1,76E-05
C1QTNF3	14326	C1q and tumor necrosis	ENSG00000082196	114899	3,83	1,09E-04
C1QTNF4	14346	C1q and tumor necrosis	ENSG00000172247	114900	3,54	4,92E-03
C1QTNF7	14342	C1q and tumor necrosis	ENSG00000163145	114905	8,35	7,12E-12
C1QTNF9	28732	C1q and tumor necrosis	ENSG00000240654	338872	3,33	9,79E-03
C1R	1246	complement component	ENSG00000159403	715	6,11	2,59E-05
C1S	1247	complement component	ENSG00000182326	716	7,04	1,99E-06
C2	1248	complement component	ENSG00000204364	717	5,09	1,00E-05
C20orf118					3,08	1,40E-02
C20orf166-AS1	26393	C20orf166 antisense RN	ENSG00000174403	253868	6,63	8,99E-06
C2orf40	24642	chromosome 2 open rea	ENSG00000119147	84417	7,18	6,43E-07
C2orf74	34439	chromosome 2 open rea	ENSG00000237651	339804	2,30	4,94E-04
C2orf81	34350	chromosome 2 open rea	ENSG00000159239	388963	3,02	2,14E-02
C2orf88	28191	chromosome 2 open rea	ENSG00000187699	84281	2,50	1,32E-03
C3	1318	complement component	ENSG00000125730	718	8,20	2,77E-07
C3AR1	1319	complement component	ENSG00000171860	719	5,00	2,66E-03
C3orf15					3,86	4,26E-05
C3orf32					3,79	4,36E-02
C3orf70	33731	chromosome 3 open rea	ENSG00000187068	285382	3,72	2,12E-04
C4orf47	34346	chromosome 4 open rea	ENSG00000205129	441054	2,39	3,13E-02
C5AR1	1338	complement component	ENSG00000197405	728	5,84	5,66E-05
C5orf38	24226	chromosome 5 open rea	ENSG00000186493	153571	3,86	4,21E-03
C5orf62					2,62	4,47E-02
C6	1339	complement component	ENSG00000039537	729	8,69	1,77E-09
C6orf25	13937	chromosome 6 open rea	ENSG00000228090	80739	4,57	8,08E-03
C6orf97					2,51	1,71E-02
C7	1346	complement component	ENSG00000112936	730	8,39	2,87E-12
C7orf31	21722	chromosome 7 open rea	ENSG00000153790	136895	2,20	2,01E-03
C7orf58					4,21	4,61E-04
C7orf63	26107	chromosome 7 open rea	ENSG00000105792	79846	3,86	1,42E-07
C8orf34	30905	chromosome 8 open rea	ENSG00000165084	116328	3,20	2,44E-03
C8orf46	28498	chromosome 8 open rea	ENSG00000169085	254778	4,17	3,52E-04
C8orf84					6,19	5,57E-09
C9orf129	31116	chromosome 9 open rea	ENSG00000204352	445577	3,69	1,41E-02
C9orf131	31418	chromosome 9 open rea	ENSG00000174038	138724	4,64	6,95E-03
C9orf71					4,17	9,72E-05
C9orf93					2,15	9,99E-03
CA8	1382	carbonic anhydrase VIII	ENSG00000178538	767	5,19	1,09E-03
CAB39L	20290	calcium binding protein	ENSG00000102547	81617	3,39	3,66E-07
CABP1	1384	calcium binding protein	ENSG00000157782	9478	3,54	4,29E-02
CACNA1C	1390	calcium channel, voltag	ENSG00000151067	775	6,30	4,87E-06
CACNA1E	1392	calcium channel, voltag	ENSG00000198216	777	5,05	9,62E-05
CACNA1F	1393	calcium channel, voltag	ENSG00000102001	778	3,08	5,18E-03
CACNA1G	1394	calcium channel, voltag	ENSG00000006283	8913	3,86	9,31E-03
CACNA1H	1395	calcium channel, voltag	ENSG00000196557	8912	6,57	1,25E-06
CACNA2D2	1400	calcium channel, voltag	ENSG00000007402	9254	3,56	6,21E-05

CACNA2D3	15460	calcium channel, voltag	ENSG00000157445	55799	4,16	2,97E-04
CACNA2D4	20202	calcium channel, voltag	ENSG00000151062	93589	2,41	1,69E-02
CACNB2	1402	calcium channel, voltag	ENSG00000165995	783	6,86	1,82E-08
CACNB4	1404	calcium channel, voltag	ENSG00000182389	785	4,21	1,49E-05
CADM1	5951	cell adhesion molecule :	ENSG00000182985	23705	3,53	5,54E-03
CADM2	29849	cell adhesion molecule :	ENSG00000175161	253559	4,98	4,35E-05
CADPS	1426	Ca++-dependent secret	ENSG00000163618	8618	5,66	8,64E-03
CALB2	1435	calbindin 2 [Source:HGI	ENSG00000172137	794	6,04	5,56E-03
CAP2	20039	CAP, adenylate cyclase-	ENSG00000112186	10486	3,04	7,33E-03
CAPN12	13249	calpain 12 [Source:HGN	ENSG00000182472	147968	3,14	1,06E-02
CAPN3	1480	calpain 3, (p94) [Sourc	ENSG00000092529	825	3,94	3,65E-09
CAPN6	1483	calpain 6 [Source:HGN(	ENSG00000077274	827	6,29	2,36E-03
CAPN9	1486	calpain 9 [Source:HGN(	ENSG00000135773	10753	3,16	2,56E-02
CAPS2	16471	calcyphosine 2 [Source:	ENSG00000180881	84698	4,61	3,43E-05
CASC1	29599	cancer susceptibility car	ENSG00000118307	55259	3,46	1,90E-03
CASC2	22933	cancer susceptibility car	ENSG00000177640	255082	2,37	4,46E-02
CASQ1	1512	calsequestrin 1 (fast-tw	ENSG00000143318	844	6,59	3,22E-06
CASQ2	1513	calsequestrin 2 (cardiac	ENSG00000118729	845	11,27	2,44E-16
CASS4	15878	Cas scaffolding protein	ENSG00000087589	57091	3,21	1,90E-02
CASZ1	26002	castor zinc finger 1 [Sou	ENSG00000130940	54897	3,23	9,31E-08
CBFA2T3	1537	core-binding factor, run	ENSG00000129993	863	5,88	7,59E-06
CBR3-AS1	43664	CBR3 antisense RNA 1 [	ENSG00000236830	100506428	2,07	3,24E-02
CBWD2	17907	COBW domain containir	ENSG00000136682	55871	2,38	2,47E-03
CBX7	1557	chromobox homolog 7 [	ENSG00000100307	23492	2,48	1,25E-07
CC2D2A	29253	coiled-coil and C2 doma	ENSG00000048342	57545	3,00	2,13E-06
CC2D2B	31666	coiled-coil and C2 doma	ENSG00000188649	387707	6,13	1,16E-06
CCBP2					2,45	2,50E-04
CCDC102B	26295	coiled-coil domain conta	ENSG00000150636	79839	5,40	5,08E-07
CCDC110	28504	coiled-coil domain conta	ENSG00000168491	256309	3,31	1,23E-03
CCDC121	25833	coiled-coil domain conta	ENSG00000176714	79635	3,19	6,17E-04
CCDC136	22225	coiled-coil domain conta	ENSG00000128596	64753	2,61	2,23E-04
CCDC141	26821	coiled-coil domain conta	ENSG00000163492	285025	6,53	2,71E-12
CCDC146	29296	coiled-coil domain conta	ENSG00000135205	57639	4,04	4,34E-12
CCDC152	34438	coiled-coil domain conta	ENSG00000198865	100129792	2,95	8,91E-04
CCDC158	26374	coiled-coil domain conta	ENSG00000163749	339965	4,68	4,67E-05
CCDC17	26574	coiled-coil domain conta	ENSG00000159588	149483	3,44	2,96E-05
CCDC3	23813	coiled-coil domain conta	ENSG00000151468	83643	5,44	1,72E-03
CCDC30	26103	coiled-coil domain conta	ENSG00000186409	728621	4,55	1,41E-04
CCDC36	27945	coiled-coil domain conta	ENSG00000173421	339834	3,52	2,21E-02
CCDC69	24487	coiled-coil domain conta	ENSG00000198624	26112	2,83	1,47E-03
CCDC8	25367	coiled-coil domain conta	ENSG00000169515	83987	3,27	3,12E-05
CCDC89	26762	coiled-coil domain conta	ENSG00000179071	220388	5,16	1,12E-05
CCL13	10611	chemokine (C-C motif)	ENSG00000181374	6357	6,54	4,00E-08
CCL19	10617	chemokine (C-C motif)	ENSG00000172724	6363	6,19	1,01E-02
CCL21	10620	chemokine (C-C motif)	ENSG00000137077	6366	6,79	1,26E-04
CCL28	17700	chemokine (C-C motif)	ENSG00000151882	56477	2,25	5,54E-03
CCL3	10627	chemokine (C-C motif)	ENSG00000006075	6348	7,14	3,46E-04
CCL4	10630	chemokine (C-C motif)	ENSG00000129277	6351	6,63	1,13E-03
CCL8	10635	chemokine (C-C motif)	ENSG00000108700	6355	7,98	1,62E-04
CCNL1	20569	cyclin L1 [Source:HGNC	ENSG00000163660	57018	2,47	5,16E-03
CCR1	1602	chemokine (C-C motif)	ENSG00000163823	1230	4,65	1,93E-04
CCRL1					5,35	1,95E-04
CD14	1628	CD14 molecule [Source	ENSG00000170458	929	5,09	2,45E-04

CD160	17013	CD160 molecule [Sourc	ENSG00000117281	11126	2,95	5,31E-03
CD163	1631	CD163 molecule [Sourc	ENSG00000177575	9332	9,31	1,43E-04
CD1C	1636	CD1c molecule [Source	ENSG00000158481	911	5,00	2,51E-03
CD200R1	24235	CD200 receptor 1 [Sour	ENSG00000163606	131450	4,29	1,13E-02
CD209	1641	CD209 molecule [Sourc	ENSG00000090659	30835	6,20	7,67E-07
CD24	1645	CD24 molecule [Source	ENSG00000272398	100133941	6,17	4,19E-05
CD28	1653	CD28 molecule [Source	ENSG00000178562	940	4,02	1,72E-03
CD300A	19319	CD300a molecule [Sour	ENSG00000167851	11314	5,18	5,42E-04
CD300E	28874	CD300e molecule [Sour	ENSG00000186407	342510	4,57	4,13E-03
CD300LG	30455	CD300 molecule-like fa	ENSG00000161649	146894	7,26	4,86E-04
CD33	1659	CD33 molecule [Source	ENSG00000105383	945	3,83	2,48E-02
CD36	1663	CD36 molecule (thromb	ENSG00000135218	948	3,14	1,63E-02
CD4	1678	CD4 molecule [Source:I	ENSG00000010610	920	7,15	1,72E-09
CD48	1683	CD48 molecule [Source	ENSG00000117091	962	5,37	6,97E-03
CD52	1804	CD52 molecule [Source	ENSG00000169442	1043	5,54	8,80E-03
CD53	1686	CD53 molecule [Source	ENSG00000143119	963	7,69	3,32E-05
CD69	1694	CD69 molecule [Source	ENSG00000110848	969	5,94	2,01E-02
CD74	1697	CD74 molecule, major I	ENSG00000019582	972	7,53	2,23E-12
CD83	1703	CD83 molecule [Source	ENSG00000112149	9308	3,59	2,14E-03
CD84	1704	CD84 molecule [Source	ENSG00000066294	8832	5,61	2,15E-05
CD86	1705	CD86 molecule [Source	ENSG00000114013	942	4,86	1,11E-04
CDC37L1	17179	cell division cycle 37-lik	ENSG00000106993	55664	2,27	3,53E-03
CDC42BPG	29829	CDC42 binding protein I	ENSG00000171219	55561	2,79	3,68E-02
CDH19	1758	cadherin 19, type 2 [So	ENSG00000071991	28513	6,90	1,04E-05
CDH23	13733	cadherin-related 23 [So	ENSG00000107736	64072	8,16	1,18E-05
CDH26	15902	cadherin 26 [Source:HG	ENSG00000124215	60437	3,38	6,46E-03
CDHR5	7521	cadherin-related family	ENSG00000099834	53841	5,07	7,46E-04
CDK15	14434	cyclin-dependent kinase	ENSG00000138395	65061	2,56	3,99E-02
CDK18	8751	cyclin-dependent kinase	ENSG00000117266	5129	3,21	9,99E-04
CDKN2C	1789	cyclin-dependent kinase	ENSG00000123080	1031	2,34	4,23E-03
CDNF	24913	cerebral dopamine neur	ENSG00000185267	441549	2,23	3,13E-02
CDO1	1795	cysteine dioxygenase ty	ENSG00000129596	1036	5,12	3,26E-04
CDON	17104	cell adhesion associatec	ENSG00000064309	50937	2,74	2,36E-03
CDR1	1798	cerebellar degeneration	ENSG00000184258	1038	5,92	1,63E-03
CDS1	1800	CDP-diacylglycerol synt	ENSG00000163624	1040	2,93	4,14E-02
CEBPA	1833	CCAAT/enhancer bindin	ENSG00000245848	1050	3,93	1,10E-04
CEBPD	1835	CCAAT/enhancer bindin	ENSG00000221869	1052	4,27	1,25E-02
CEBPZ	24218	CCAAT/enhancer bindin	ENSG00000115816	10153	2,23	2,27E-05
CELF6	14059	CUGBP, Elav-like family	ENSG00000140488	60677	2,97	3,64E-05
CEP112	28514	centrosomal protein 11	ENSG00000154240	201134	2,30	2,42E-06
CEP70	29972	centrosomal protein 70I	ENSG00000114107	80321	2,27	1,71E-05
CES1	1863	carboxylesterase 1 [Sou	ENSG00000262243	1066	8,61	2,99E-08
CFB	1037	complement factor B [S	ENSG00000204359	629	2,83	4,78E-03
CFD	2771	complement factor D (a	ENSG00000197766	1675	10,15	7,17E-16
CFHR3	16980	complement factor H-re	ENSG00000116785	10878	5,38	3,13E-02
CFP	8864	complement factor prop	ENSG00000126759	5199	3,99	3,56E-03
CG030					2,67	1,27E-02
CHADL	25165	chondroadherin-like [Sc	ENSG00000100399	150356	2,88	1,52E-03
CHDH	24288	choline dehydrogenase	ENSG00000016391	55349	2,65	1,16E-02
CHI3L2	1933	chitinase 3-like 2 [Sour	ENSG00000064886	1117	6,93	2,06E-03
CHL1	1939	cell adhesion molecule I	ENSG00000134121	10752	8,06	5,49E-06
CHODL	17807	chondrolectin [Source:H	ENSG00000154645	140578	4,07	8,17E-04
CHRD	1949	chordin [Source:HGNC :	ENSG00000090539	8646	4,50	1,64E-07

CHRDL1	29861	chordin-like 1 [Source:H	ENSG00000101938	91851	10,11	4,43E-15
CHRM3	1952	cholinergic receptor, mu	ENSG00000133019	1131	4,59	5,76E-03
CHURC1	20099	churchill domain contair	ENSG00000258289	91612	2,08	1,70E-04
CIDEA	1976	cell death-inducing DFF	ENSG00000176194	1149	5,85	5,73E-03
CIDEC	24229	cell death-inducing DFF	ENSG00000187288	63924	8,22	3,74E-03
CIITA	7067	class II, major histocom	ENSG00000179583	4261	5,62	6,33E-05
CILP	1980	cartilage intermediate la	ENSG00000138615	8483	8,52	3,26E-07
CIR1	24217	corepressor interacting	ENSG00000138433	9541	2,21	1,47E-04
CKB	1991	creatine kinase, brain [S	ENSG00000166165	1152	5,78	2,60E-04
CKMT2	1996	creatine kinase, mitoch	ENSG00000131730	1160	6,62	2,78E-08
CLDN10	2033	claudin 10 [Source:HGN	ENSG00000134873	9071	5,87	8,29E-03
CLDN23	17591	claudin 23 [Source:HGN	ENSG00000253958	137075	4,21	3,54E-04
CLDND2	28511	claudin domain containi	ENSG00000160318	125875	2,91	4,19E-02
CLEC10A	16916	C-type lectin domain fa	ENSG00000132514	10462	5,55	5,33E-03
CLEC2D	14351	C-type lectin domain fa	ENSG00000069493	29121	2,24	1,34E-02
CLEC3B	11891	C-type lectin domain fa	ENSG00000163815	7123	8,56	2,27E-11
CLEC4A	13257	C-type lectin domain fa	ENSG00000111729	50856	3,80	9,98E-05
CLEC4E	14555	C-type lectin domain fa	ENSG00000166523	26253	6,80	3,15E-04
CLEC7A	14558	C-type lectin domain fa	ENSG00000172243	64581	4,74	1,67E-03
CLIC2	2063	chloride intracellular ch	ENSG00000155962	1193	3,28	5,04E-08
CLIC5	13517	chloride intracellular ch	ENSG00000112782	53405	5,82	4,81E-06
CLIC6	2065	chloride intracellular ch	ENSG00000159212	54102	4,23	1,37E-03
CLK1	2068	CDC-like kinase 1 [Sour	ENSG00000013441	1195	2,16	5,13E-04
CLMN	19972	calmin (calponin-like, tr	ENSG00000165959	79789	2,59	2,48E-02
CLSTN2	17448	calsyntenin 2 [Source:H	ENSG00000158258	64084	3,32	6,91E-07
CLYBL	18355	citrate lyase beta like [S	ENSG00000125246	171425	3,75	7,14E-06
CMA1	2097	chymase 1, mast cell [S	ENSG00000092009	1215	6,31	5,18E-03
CMAHP	2098	cytidine monophospho-N	ENSG00000168405	8418	2,17	8,15E-03
CMKLR1	2121	chemokine-like receptor	ENSG00000174600	1240	4,95	2,06E-05
CMPK2	27015	cytidine monophosphate	ENSG00000134326	129607	2,72	7,40E-04
CMTM2	19173	CKLF-like MARVEL trans	ENSG00000140932	146225	3,94	2,44E-02
CMTM5	19176	CKLF-like MARVEL trans	ENSG00000166091	116173	4,39	2,63E-04
CMYA5	14305	cardiomyopathy associa	ENSG00000164309	202333	4,46	1,43E-04
CNIH3	26802	cornichon homolog 3 (D	ENSG00000143786	149111	2,32	1,22E-02
CNKSR2	19701	connector enhancer of k	ENSG00000149970	22866	7,23	1,16E-06
CNN1	2155	calponin 1, basic, smoo	ENSG00000130176	1264	7,67	1,80E-07
CNNM1	102	cyclin M1 [Source:HGNC	ENSG00000119946	26507	2,70	1,84E-02
CNTFR	2170	ciliary neurotrophic fact	ENSG00000122756	1271	8,72	1,29E-06
CNTN1	2171	contactin 1 [Source:HG	ENSG00000018236	1272	8,18	1,48E-20
CNTN3	2173	contactin 3 (plasmacyto	ENSG00000113805	5067	6,62	1,05E-06
CNTN4	2174	contactin 4 [Source:HG	ENSG00000144619	152330	7,20	9,37E-07
COBL	22199	cordon-bleu WH2 repea	ENSG00000106078	23242	5,15	8,21E-05
COBLL1	23571	cordon-bleu WH2 repea	ENSG00000082438	22837	2,99	1,07E-03
COL14A1	2191	collagen, type XIV, alph	ENSG00000187955	7373	9,26	1,30E-05
COL15A1	2192	collagen, type XV, alpha	ENSG00000204291	1306	5,01	4,24E-02
COL16A1	2193	collagen, type XVI, alph	ENSG00000084636	1307	5,05	2,51E-06
COL19A1	2196	collagen, type XIX, alph	ENSG00000082293	1310	5,22	1,48E-02
COL21A1	17025	collagen, type XXI, alph	ENSG00000124749	81578	7,59	6,70E-10
COL23A1	22990	collagen, type XXIII, alp	ENSG00000050767	91522	6,88	1,67E-06
COL28A1	22442	collagen, type XXVIII, a	ENSG00000215018	340267	4,20	6,38E-05
COL3A1	2201	collagen, type III, alpha	ENSG00000168542	1281	2,88	1,52E-02
COL4A6	2208	collagen, type IV, alpha	ENSG00000197565	1288	4,64	1,69E-02
COL5A3	14864	collagen, type V, alpha :	ENSG00000080573	50509	3,48	3,66E-05

COL6A6	27023	collagen, type VI, alpha	ENSG00000206384	131873	5,95	9,61E-03
COLEC11	17213	collectin sub-family mei	ENSG00000118004	78989	3,91	9,26E-03
COMP	2227	cartilage oligomeric mat	ENSG00000105664	1311	9,51	1,96E-02
COX4I2	16232	cytochrome c oxidase sı	ENSG00000131055	84701	5,81	2,85E-06
COX7A1	2287	cytochrome c oxidase sı	ENSG00000161281	1346	2,56	8,19E-03
CPA3	2298	carboxypeptidase A3 (m	ENSG00000163751	1359	4,61	2,66E-02
CPB1	2299	carboxypeptidase B1 (ti	ENSG00000153002	1360	4,45	7,57E-04
CPE	2303	carboxypeptidase E [So	ENSG00000109472	1363	7,45	1,55E-06
CPEB1	21744	cytoplasmic polyadenyla	ENSG00000214575	64506	3,53	4,96E-04
CPLX1	2309	complexin 1 [Source:H(	ENSG00000168993	10815	2,88	4,02E-02
CPM	2311	carboxypeptidase M [Sc	ENSG00000135678	1368	5,74	6,94E-05
CPNE6	2319	copine VI (neuronal) [S	ENSG00000100884	9362	4,40	7,44E-03
CPVL	14399	carboxypeptidase, vitell	ENSG00000106066	54504	5,74	9,30E-10
CPXM1	15771	carboxypeptidase X (M1	ENSG00000088882	56265	4,53	1,43E-02
CPXM2	26977	carboxypeptidase X (M1	ENSG00000121898	119587	4,19	6,20E-03
CR1	2334	complement component	ENSG00000203710	1378	3,53	8,10E-03
CRABP2	2339	cellular retinoic acid bin	ENSG00000143320	1382	7,16	8,93E-09
CREB5	16844	cAMP responsive elemei	ENSG00000146592	9586	2,28	1,15E-02
CREG1	2351	cellular repressor of E1/	ENSG00000143162	8804	2,50	3,57E-06
CRHBP	2356	corticotropin releasing I	ENSG00000145708	1393	6,44	3,33E-03
CRIP1	2360	cysteine-rich protein 1 (	ENSG00000213145	1396	7,12	1,58E-04
CRIP3	17751	cysteine-rich protein 3 [	ENSG00000146215	401262	4,18	6,68E-04
CRISPLD1	18206	cysteine-rich secretory	ENSG00000121005	83690	3,11	2,51E-02
CRISPLD2	25248	cysteine-rich secretory	ENSG00000103196	83716	4,97	1,20E-02
CRLF1	2364	cytokine receptor-like fa	ENSG00000006016	9244	5,73	1,35E-02
CRTAC1	14882	cartilage acidic protein	ENSG00000095713	55118	2,55	4,63E-02
CRYM	2418	crystallin, mu [Source:I	ENSG00000103316	1428	5,21	7,26E-04
CRYZ	2419	crystallin, zeta (quinone	ENSG00000116791	1429	2,41	7,69E-05
CSDC2	30359	cold shock domain cont	ENSG00000172346	27254	7,26	5,47E-06
CSF1R	2433	colony stimulating facto	ENSG00000182578	1436	6,74	1,58E-08
CSF3R	2439	colony stimulating facto	ENSG00000119535	1441	7,04	2,68E-03
CSGALNACT1	24290	chondroitin sulfate N-ac	ENSG00000147408	55790	2,17	6,73E-03
CSRNP1	14300	cysteine-serine-rich nuc	ENSG00000144655	64651	4,88	5,75E-04
CSRNP3	30729	cysteine-serine-rich nuc	ENSG00000178662	80034	5,68	1,94E-07
CSRP1	2469	cysteine and glycine-ric	ENSG00000159176	1465	2,39	3,35E-02
CSRP2	2470	cysteine and glycine-ric	ENSG00000175183	1466	2,36	1,56E-05
CST3	2475	cystatin C [Source:HGN	ENSG00000101439	1471	2,49	1,24E-02
CST7	2479	cystatin F (leukocystatir	ENSG00000077984	8530	5,00	8,39E-03
CSTA	2481	cystatin A (stefin A) [Sc	ENSG00000121552	1475	3,29	5,77E-03
CTNND2	2516	catenin (cadherin-assoc	ENSG00000169862	1501	5,49	1,07E-02
CTRL	2524	chymotrypsin-like [Sour	ENSG00000141086	1506	2,07	4,80E-02
CTSG	2532	cathepsin G [Source:HG	ENSG00000100448	1511	6,48	8,08E-04
CTSK	2536	cathepsin K [Source:HG	ENSG00000143387	1513	6,88	1,44E-13
CTSW	2546	cathepsin W [Source:H(	ENSG00000172543	1521	3,30	1,54E-02
CTTNBP2	15679	cortactin binding protei	ENSG00000077063	83992	4,03	4,14E-04
CWC22	29322	CWC22 spliceosome-as:	ENSG00000163510	57703	2,17	5,75E-07
CWF19L2	26508	CWF19-like 2, cell cycle	ENSG00000152404	143884	3,02	3,18E-10
CX3CR1	2558	chemokine (C-X3-C mo	ENSG00000168329	1524	4,45	4,34E-03
CXCL12	10672	chemokine (C-X-C moti	ENSG00000107562	6387	3,46	9,26E-03
CXCL14	10640	chemokine (C-X-C moti	ENSG00000145824	9547	9,22	1,09E-14
CXCL2	4603	chemokine (C-X-C moti	ENSG00000081041	2920	4,94	1,35E-03
CXCL9	7098	chemokine (C-X-C moti	ENSG00000138755	4283	5,16	1,81E-02
CXCR1	6026	chemokine (C-X-C moti	ENSG00000163464	3577	6,00	1,36E-02

CXCR2	6027	chemokine (C-X-C motil	ENSG00000180871	3579	6,07	1,26E-02
CXCR7					3,13	3,87E-04
CXorf21	25667	chromosome X open rea	ENSG00000120280	80231	3,59	1,84E-02
CXorf69					3,17	1,44E-04
CXXC4	24593	CXXC finger protein 4 [S	ENSG00000168772	80319	4,81	1,68E-04
CYBB	2578	cytochrome b-245, beta	ENSG00000165168	1536	8,38	9,08E-12
CYBRD1	20797	cytochrome b reductase	ENSG00000071967	79901	2,08	1,70E-02
CYFIP2	13760	cytoplasmic FMR1 intera	ENSG00000055163	26999	3,50	5,52E-03
CYP1B1	2597	cytochrome P450, famil	ENSG00000138061	1545	8,96	1,87E-03
CYP39A1	17449	cytochrome P450, famil	ENSG00000146233	51302	6,84	1,11E-06
CYP4B1	2644	cytochrome P450, famil	ENSG00000142973	1580	6,65	3,84E-03
CYP4F12	18857	cytochrome P450, famil	ENSG00000186204	66002	6,50	1,24E-06
CYP4F24P	39945	cytochrome P450, famil	ENSG00000267594	388514	5,27	2,36E-04
CYP4V2	23198	cytochrome P450, famil	ENSG00000145476	285440	2,94	2,64E-11
CYP4X1	20244	cytochrome P450, famil	ENSG00000186377	260293	5,01	1,51E-05
CYP4Z1	20583	cytochrome P450, famil	ENSG00000186160	199974	4,12	8,35E-04
CYP7B1	2652	cytochrome P450, famil	ENSG00000172817	9420	4,11	3,52E-04
CYP8B1	2653	cytochrome P450, famil	ENSG00000180432	1582	2,81	1,04E-03
CYS1	18525	cystin 1 [Source:HGNC	ENSG00000205795	192668	4,31	1,05E-08
CYSLTR1	17451	cysteinyl leukotriene re	ENSG00000173198	10800	7,26	5,64E-09
CYSLTR2	18274	cysteinyl leukotriene re	ENSG00000152207	57105	5,39	1,97E-04
CYTH4	9505	cytohesin 4 [Source:HG	ENSG00000100055	27128	6,38	6,25E-06
CYTIP	9506	cytohesin 1 interacting	ENSG00000115165	9595	5,14	4,19E-03
DAAM2	18143	dishevelled associated a	ENSG00000146122	23500	3,29	2,97E-04
DAB1	2661	Dab, reelin signal transo	ENSG00000173406	1600	7,94	1,61E-09
DACT1	17748	dishevelled-binding anta	ENSG00000165617	51339	3,42	8,89E-04
DACT2	21231	dishevelled-binding anta	ENSG00000164488	168002	3,93	2,51E-02
DACT3	30745	dishevelled-binding anta	ENSG00000197380	147906	4,22	2,05E-04
DAPP1	16500	dual adaptor of phosph	ENSG00000070190	27071	3,49	6,66E-03
DARC	4035	Duffy blood group, atyp	ENSG00000213088	2532	5,29	3,07E-04
DBC1	2687	deleted in bladder canc	ENSG00000078725	1620	4,82	8,02E-03
DBNDD2	15881	dysbindin (dystrobrevin	ENSG00000244274	55861	3,76	1,26E-03
DCAF12L1	29395	DDB1 and CUL4 associa	ENSG00000198889	139170	2,68	9,08E-03
DCAF12L2	32950	DDB1 and CUL4 associa	ENSG00000198354	340578	3,16	1,09E-03
DCC	2701	deleted in colorectal car	ENSG00000187323	1630	3,56	4,74E-02
DCHS2	23111	dachsous 2 (Drosophila	ENSG00000197410	54798	3,40	3,02E-02
DCN	2705	decorin [Source:HGNC S	ENSG00000011465	1634	7,78	1,15E-10
DCST2	26562	DC-STAMP domain cont	ENSG00000163354	127579	3,17	7,04E-04
DDO	2727	D-aspartate oxidase [So	ENSG00000203797	8528	2,76	1,17E-03
DDX25	18698	DEAD (Asp-Glu-Ala-Asp	ENSG00000109832	29118	3,91	3,69E-03
DDX3X	2745	DEAD (Asp-Glu-Ala-Asp	ENSG00000215301	1654	2,33	1,59E-03
DEF6	2760	differentially expressed	ENSG00000023892	50619	2,30	3,03E-02
DEGS2	20113	delta(4)-desaturase, sp	ENSG00000168350	123099	5,04	1,38E-03
DENND1C	26225	DENN/MADD domain co	ENSG00000205744	79958	3,82	8,98E-03
DENND2A	22212	DENN/MADD domain co	ENSG00000146966	27147	4,68	2,03E-06
DENND2C	24748	DENN/MADD domain co	ENSG00000175984	163259	2,36	1,72E-02
DEPDC7	29899	DEP domain containing	ENSG00000121690	91614	3,07	1,57E-02
DEPTOR	22953	DEP domain containing	ENSG00000155792	64798	6,16	4,85E-07
DES	2770	desmin [Source:HGNC S	ENSG00000175084	1674	10,06	1,67E-11
DFNB31	16361	deafness, autosomal re	ENSG00000095397	25861	2,15	3,11E-02
DGKG	2853	diacylglycerol kinase, ga	ENSG00000058866	1608	3,16	3,03E-02
DIO2	2884	deiodinase, iodothyronii	ENSG00000211448	1734	3,53	1,88E-02
DIO3	2885	deiodinase, iodothyronii	ENSG00000197406	1735	6,28	5,51E-05

DIO3OS	20348	DIO3 opposite strand/a	ENSG00000258498	100302145	4,67	8,77E-05
DIRC3	17805	disrupted in renal carcir	ENSG00000231672	729582	5,09	4,61E-06
DKK2	2892	dickkopf WNT signaling	ENSG00000155011	27123	4,57	1,26E-02
DLEC1	2899	deleted in lung and eso	ENSG00000008226	9940	3,34	4,19E-03
DLG2	2901	discs, large homolog 2 (	ENSG00000150672	1740	3,83	5,31E-05
DLGAP2	2906	discs, large (Drosophila	ENSG00000198010	9228	7,00	3,73E-09
DLL1	2908	delta-like 1 (Drosophila	ENSG00000198719	28514	2,21	3,75E-03
DLX5	2918	distal-less homeobox 5	ENSG00000105880	1749	5,44	7,02E-03
DLX6	2919	distal-less homeobox 6	ENSG00000006377	1750	4,94	6,09E-03
DMC1	2927	DNA meiotic recombina:	ENSG00000100206	11144	2,64	1,07E-02
DMD	2928	dystrophin [Source:HGI	ENSG00000198947	1756	2,69	2,20E-04
DMGDH	24475	dimethylglycine dehydr	ENSG00000132837	29958	3,68	3,34E-02
DMKN	25063	dermokine [Source:HGI	ENSG00000161249	93099	6,11	9,84E-09
DMRT2	2935	doublesex and mab-3 re	ENSG00000173253	10655	5,00	3,83E-04
DMRT3	13909	doublesex and mab-3 re	ENSG00000064218	58524	6,63	1,16E-05
DNAH6	2951	dynein, axonemal, heav	ENSG00000115423	1768	4,10	2,88E-03
DNAH7	18661	dynein, axonemal, heav	ENSG00000118997	56171	3,65	1,35E-02
DNAJC27	30290	DnaJ (Hsp40) homolog,	ENSG00000115137	51277	2,19	3,41E-03
DNASE1L3	2959	deoxyribonuclease I-like	ENSG00000163687	1776	3,30	3,13E-02
DNHD1	26532	dynein heavy chain don	ENSG00000179532	144132	2,04	1,70E-03
DNM1	2972	dynamin 1 [Source:HGI	ENSG00000106976	1759	3,37	2,23E-03
DNM3	29125	dynamin 3 [Source:HGI	ENSG00000197959	26052	2,07	3,37E-02
DNM3OS	41228	DNM3 opposite strand/a	ENSG00000230630	100628315	2,84	2,82E-02
DNTTIP2	24013	deoxynucleotidyltransfe	ENSG00000067334	30836	2,43	9,80E-04
DOCK11	23483	dedicator of cytokinesis	ENSG00000147251	139818	3,47	6,01E-07
DOCK2	2988	dedicator of cytokinesis	ENSG00000134516	1794	3,70	5,10E-04
DOCK3	2989	dedicator of cytokinesis	ENSG00000260587	1795	3,63	7,82E-04
DOK2	2991	docking protein 2, 56kD	ENSG00000147443	9046	4,00	1,46E-04
DOK5	16173	docking protein 5 [Sour	ENSG00000101134	55816	5,85	5,30E-11
DOK6	28301	docking protein 6 [Sour	ENSG00000206052	220164	4,70	4,43E-08
DPEP2	23028	dipeptidase 2 [Source:H	ENSG00000167261	64174	4,42	8,12E-05
DPT	3011	dermatopontin [Source:	ENSG00000143196	1805	9,04	7,03E-08
DPY19L2	19414	dpy-19-like 2 (C. elegar	ENSG00000177990	283417	2,00	4,66E-02
DPYSL3	3015	dihydropyrimidinase-like	ENSG00000113657	1809	2,82	6,63E-06
DSC2	3036	desmocollin 2 [Source:I	ENSG00000134755	1824	4,18	2,44E-04
DSCAML1	14656	Down syndrome cell adl	ENSG00000177103	57453	5,21	4,57E-04
DSG2	3049	desmoglein 2 [Source:H	ENSG00000046604	1829	3,66	3,09E-02
DSTN	15750	destrin (actin depolyme	ENSG00000125868	11034	2,23	1,92E-02
DTNA	3057	dystrobrevin, alpha [So	ENSG00000134769	1837	3,45	5,43E-03
DTWD1	30926	DTW domain containing	ENSG00000104047	56986	2,42	1,46E-05
DTX1	3060	deltex homolog 1 (Dros	ENSG00000135144	1840	2,98	2,23E-02
DUSP1	3064	dual specificity phospha	ENSG00000120129	1843	3,13	4,29E-04
DUSP15	16236	dual specificity phospha	ENSG00000149599	128853	2,53	3,66E-02
DUSP2	3068	dual specificity phospha	ENSG00000158050	1844	5,40	1,01E-03
DUSP26	28161	dual specificity phospha	ENSG00000133878	78986	3,66	1,19E-02
DYNC2H1	2962	dynein, cytoplasmic 2, I	ENSG00000187240	79659	2,42	1,24E-03
EAF2	23115	ELL associated factor 2	ENSG00000145088	55840	3,27	2,01E-03
EBF1	3126	early B-cell factor 1 [So	ENSG00000164330	1879	4,85	1,81E-07
EBF2	19090	early B-cell factor 2 [So	ENSG00000221818	64641	9,29	7,06E-17
ECM2	3154	extracellular matrix pro	ENSG00000106823	1842	8,94	9,95E-21
EDA	3157	ectodysplasin A [Source	ENSG00000158813	1896	2,66	1,89E-05
EDNRA	3179	endothelin receptor typ	ENSG00000151617	1909	4,05	1,28E-09
EEPD1	22223	endonuclease/exonucle	ENSG00000122547	80820	4,42	5,60E-05

EFCAB7	29379	EF-hand calcium binding	ENSG00000203965	84455	2,30	7,30E-04
EFHC2	26233	EF-hand domain (C-term	ENSG00000183690	80258	6,80	1,00E-06
EFHD1	29556	EF-hand domain family,	ENSG00000115468	80303	4,02	1,55E-09
EFS	16898	embryonal Fyn-associat	ENSG00000100842	10278	7,52	3,61E-14
EGF	3229	epidermal growth factor	ENSG00000138798	1950	4,34	4,67E-03
EGFLAM	26810	EGF-like, fibronectin typ	ENSG00000164318	133584	6,89	1,53E-09
EGFR	3236	epidermal growth factor	ENSG00000146648	1956	2,67	5,82E-04
EGLN3	14661	egl nine homolog 3 (C.	ENSG00000129521	112399	4,28	1,89E-03
EGR1	3238	early growth response 1	ENSG00000120738	1958	6,06	1,02E-05
EGR2	3239	early growth response 2	ENSG00000122877	1959	4,60	5,63E-04
EGR3	3240	early growth response 3	ENSG00000179388	1960	6,50	3,20E-04
EHBP1L1	30682	EH domain binding prot	ENSG00000173442	254102	2,11	1,83E-02
EID3	32961	EP300 interacting inhibi	ENSG00000255150	493861	3,34	2,32E-03
EIF4E3	31837	eukaryotic translation ir	ENSG00000163412	317649	3,00	1,16E-06
ELN	3327	elastin [Source:HGNC S	ENSG00000049540	2006	3,37	4,61E-03
ELOVL2	14416	ELOVL fatty acid elonga	ENSG00000197977	54898	6,34	6,48E-06
ELOVL7	26292	ELOVL fatty acid elonga	ENSG00000164181	79993	6,18	2,00E-04
EMB	30465	embigin [Source:HGNC	ENSG00000170571	133418	7,28	7,98E-10
EML5	18197	echinoderm microtubule	ENSG00000165521	161436	2,87	5,52E-05
EMX2	3341	empty spiracles homeob	ENSG00000170370	2018	8,25	9,05E-14
EMX2OS	18511	EMX2 opposite strand/a	ENSG00000229847	196047	8,17	9,68E-16
EN1	3342	engrailed homeobox 1 [	ENSG00000163064	2019	6,02	6,17E-09
ENDOU	14369	endonuclease, polyU-sp	ENSG00000111405	8909	6,29	1,92E-07
ENPEP	3355	glutamyl aminopeptidas	ENSG00000138792	2028	7,44	2,28E-10
ENPP1	3356	ectonucleotide pyrophos	ENSG00000197594	5167	3,92	8,97E-05
ENPP2	3357	ectonucleotide pyrophos	ENSG00000136960	5168	3,65	2,59E-03
ENPP3	3358	ectonucleotide pyrophos	ENSG00000154269	5169	3,97	5,06E-04
ENPP5	13717	ectonucleotide pyrophos	ENSG00000112796	59084	3,56	2,12E-02
ENPP6	23409	ectonucleotide pyrophos	ENSG00000164303	133121	3,57	1,73E-03
ENTPD1	3363	ectonucleoside triphosp	ENSG00000138185	953	2,49	2,98E-02
ENTPD2	3364	ectonucleoside triphosp	ENSG00000054179	954	5,49	8,72E-05
ENTPD3	3365	ectonucleoside triphosp	ENSG00000168032	956	4,63	3,39E-03
EPHA1	3385	EPH receptor A1 [Sourc	ENSG00000146904	2041	6,01	1,57E-05
EPHA3	3387	EPH receptor A3 [Sourc	ENSG00000044524	2042	6,66	1,19E-08
EPHA7	3390	EPH receptor A7 [Sourc	ENSG00000135333	2045	3,71	4,68E-02
EPHB3	3394	EPH receptor B3 [Sourc	ENSG00000182580	2049	6,32	1,68E-06
EPHB6	3396	EPH receptor B6 [Sourc	ENSG00000260195	2051	4,79	1,29E-07
EPHX2	3402	epoxide hydrolase 2, cy	ENSG00000120915	2053	3,69	5,09E-05
EPPK1	15577	epiplakin 1 [Source:HGI	ENSG00000227184	83481	4,62	8,58E-03
ERBB3	3431	v-erb-b2 avian erythrob	ENSG00000065361	2065	4,86	2,20E-04
ERBB4	3432	v-erb-b2 avian erythrob	ENSG00000178568	2066	5,20	3,80E-05
ESPNL	27937	espin-like [Source:HGN	ENSG00000144488	339768	3,14	1,36E-02
ESR1	3467	estrogen receptor 1 [So	ENSG00000091831	2099	6,39	3,15E-17
ESRG	39079	embryonic stem cell rela	ENSG00000265992	790952	4,28	2,62E-03
ESRRB	3473	estrogen-related recept	ENSG00000119715	2103	4,37	3,31E-03
ETV7	18160	ets variant 7 [Source:H	ENSG00000010030	51513	2,53	4,08E-02
EVI2A	3499	ecotropic viral integratio	ENSG00000126860	2123	3,07	1,72E-03
EVI2B	3500	ecotropic viral integratio	ENSG00000185862	2124	3,98	6,00E-04
EVPL	3503	envoplakin [Source:HGI	ENSG00000167880	2125	5,01	2,47E-04
EXOC3L2	30162	exocyst complex compo	ENSG00000130201	90332	2,77	3,37E-02
EXOC3L4	20120	exocyst complex compo	ENSG00000205436	91828	5,14	2,27E-05
EYA2	3520	eyes absent homolog 2	ENSG00000064655	2139	6,54	4,99E-04
EYA4	3522	eyes absent homolog 4	ENSG00000112319	2070	5,88	1,56E-04

F10	3528	coagulation factor X [Sc	ENSG00000126218	2159	5,26	9,50E-07
F13A1	3531	coagulation factor XIII,	ENSG00000124491	2162	9,77	4,89E-13
F3	3541	coagulation factor III (t	ENSG00000117525	2152	3,41	2,67E-03
F5	3542	coagulation factor V (pr	ENSG00000198734	2153	5,96	2,41E-04
F7	3544	coagulation factor VII (s	ENSG00000057593	2155	4,62	9,68E-05
F8	3546	coagulation factor VIII,	ENSG00000185010	2157	3,23	1,73E-04
FAAH	3553	fatty acid amide hydrola	ENSG00000117480	2166	2,05	7,98E-03
FABP3	3557	fatty acid binding protei	ENSG00000121769	2170	6,46	1,46E-07
FAIM2	17067	Fas apoptotic inhibitory	ENSG00000135472	23017	4,13	1,73E-02
FAM102B	27637	family with sequence si	ENSG00000162636	284611	3,92	1,30E-07
FAM105A	25629	family with sequence si	ENSG00000145569	54491	3,97	1,33E-04
FAM110C	33340	family with sequence si	ENSG00000184731	642273	5,41	2,34E-06
FAM134B	25964	family with sequence si	ENSG00000154153	54463	6,30	3,07E-07
FAM13A	19367	family with sequence si	ENSG00000138640	10144	4,80	8,49E-13
FAM13A-AS1	19370	FAM13A antisense RNA	ENSG00000248019	285512	2,61	1,82E-03
FAM13C	19371	family with sequence si	ENSG00000148541	220965	2,84	4,12E-03
FAM149A	24527	family with sequence si	ENSG00000109794	25854	5,51	4,37E-12
FAM150B	27683	family with sequence si	ENSG00000189292	285016	9,85	2,18E-04
FAM162B	21549	family with sequence si	ENSG00000183807	221303	2,55	3,89E-02
FAM166A	33818	family with sequence si	ENSG00000188163	401565	2,63	3,66E-02
FAM166B	34242	family with sequence si	ENSG00000215187	730112	2,86	3,88E-02
FAM178B	28036	family with sequence si	ENSG00000168754	51252	4,20	5,98E-03
FAM180A	33773	family with sequence si	ENSG00000189320	389558	3,86	5,32E-03
FAM180B	34451	family with sequence si	ENSG00000196666	399888	8,18	7,19E-09
FAM184A	20991	family with sequence si	ENSG00000111879	79632	4,70	3,22E-06
FAM18A					2,88	2,42E-03
FAM190A					4,35	2,44E-05
FAM198A	24485	family with sequence si	ENSG00000144649	729085	6,69	3,44E-11
FAM19A2	21589	family with sequence si	ENSG00000198673	338811	3,01	1,20E-02
FAM19A5	21592	family with sequence si	ENSG00000219438	25817	3,95	5,60E-03
FAM20A	23015	family with sequence si	ENSG00000108950	54757	4,14	4,35E-06
FAM26F	33391	family with sequence si	ENSG00000188820	441168	3,35	5,99E-03
FAM3B	1253	family with sequence si	ENSG00000183844	54097	4,56	5,10E-05
FAM43B	31791	family with sequence si	ENSG00000183114	163933	4,70	2,93E-03
FAM46A	18345	family with sequence si	ENSG00000112773	55603	2,62	1,25E-02
FAM46B	28273	family with sequence si	ENSG00000158246	115572	5,28	1,08E-04
FAM46C	24712	family with sequence si	ENSG00000183508	54855	3,77	6,89E-03
FAM47E	34343	family with sequence si	ENSG00000189157	100129583	2,37	2,92E-02
FAM59A					2,70	3,78E-03
FAM65B	13872	family with sequence si	ENSG00000111913	9750	5,86	3,28E-03
FAM65C	16168	family with sequence si	ENSG00000042062	140876	6,62	3,14E-06
FAM69A	32213	family with sequence si	ENSG00000154511	388650	2,77	4,01E-06
FAM70A					5,83	3,46E-04
FAM71A	26541	family with sequence si	ENSG00000162771	149647	6,60	2,67E-07
FAM71F2	27998	family with sequence si	ENSG00000205085	346653	2,32	4,59E-02
FAM76A	28530	family with sequence si	ENSG00000009780	199870	2,21	1,92E-05
FAM82A1					3,20	1,33E-06
FAS	11920	Fas cell surface death re	ENSG00000026103	355	2,63	9,41E-05
FAS-AS1					3,12	1,53E-02
FAT3	23112	FAT atypical cadherin 3	ENSG00000165323	120114	4,84	5,45E-08
FBLN1	3600	fibulin 1 [Source:HGNC	ENSG00000077942	2192	5,11	3,71E-04
FBLN2	3601	fibulin 2 [Source:HGNC	ENSG00000163520	2199	2,56	1,35E-02
FBLN5	3602	fibulin 5 [Source:HGNC	ENSG00000140092	10516	2,76	2,73E-02

FBLN7	26740	fibulin 7 [Source:HGNC	ENSG00000144152	129804	2,45	5,54E-04
FBXL13	21658	F-box and leucine-rich r	ENSG00000161040	222235	2,89	2,41E-03
FBXL22	27537	F-box and leucine-rich r	ENSG00000197361	283807	5,13	5,75E-05
FBXO16	13618	F-box protein 16 [Sourc	ENSG00000214050	157574	5,61	1,96E-03
FBXO40	29816	F-box protein 40 [Sourc	ENSG00000163833	51725	5,50	5,10E-05
FCAR	3608	Fc fragment of IgA, rece	ENSG00000186431	2204	4,12	4,52E-02
FCER1A	3609	Fc fragment of IgE, high	ENSG00000179639	2205	6,09	1,10E-03
FCER1G	3611	Fc fragment of IgE, high	ENSG00000158869	2207	6,45	9,04E-09
FCGBP	13572	Fc fragment of IgG bind	ENSG00000090920	8857	4,52	1,48E-02
FCGR2A	3616	Fc fragment of IgG, low	ENSG00000143226	2212	5,88	8,39E-08
FCGR2B	3618	Fc fragment of IgG, low	ENSG00000072694	2213	7,91	2,52E-09
FCGR3A	3619	Fc fragment of IgG, low	ENSG00000203747	2214	5,90	2,47E-04
FCGR3B	3620	Fc fragment of IgG, low	ENSG00000162747	2215	6,34	1,16E-02
FCN1	3623	ficolin (collagen/fibrinog	ENSG00000085265	2219	5,73	3,59E-03
FGD2	3664	FYVE, RhoGEF and PH d	ENSG00000146192	221472	6,12	1,78E-05
FGD3	16027	FYVE, RhoGEF and PH d	ENSG00000127084	89846	5,61	3,62E-05
FGF10	3666	fibroblast growth factor	ENSG00000070193	2255	3,50	2,12E-02
FGF18	3674	fibroblast growth factor	ENSG00000156427	8817	4,27	9,72E-03
FGF7	3685	fibroblast growth factor	ENSG00000140285	2252	10,06	1,42E-24
FGFBP2	29451	fibroblast growth factor	ENSG00000137441	83888	4,30	3,21E-03
FGFR2	3689	fibroblast growth factor	ENSG00000066468	2263	6,11	5,07E-08
FGL2	3696	fibrinogen-like 2 [Sourc	ENSG00000127951	10875	9,01	1,19E-09
FGR	3697	feline Gardner-Rasheed	ENSG00000000938	2268	4,72	5,77E-03
FHDC1	29363	FH2 domain containing	ENSG00000137460	85462	3,92	2,66E-04
FHL1	3702	four and a half LIM dom	ENSG00000022267	2273	2,46	4,12E-03
FHL5	17371	four and a half LIM dom	ENSG00000112214	9457	8,88	1,68E-11
FHOD3	26178	formin homology 2 dom	ENSG00000134775	80206	3,61	4,32E-03
FIBIN	33747	fin bud initiation factor	ENSG00000176971	387758	2,64	4,75E-03
FIGF	3708	c-fos induced growth fa	ENSG00000165197	2277	5,29	6,56E-03
FIGN	13285	fidgetin [Source:HGNC	ENSG00000182263	55137	2,32	4,87E-03
FILIP1	21015	filamin A interacting pro	ENSG00000118407	27145	2,74	1,64E-02
FILIP1L	24589	filamin A interacting pro	ENSG00000168386	11259	2,30	3,39E-03
FLJ31485					5,90	1,41E-04
FLJ34690					4,68	2,92E-03
FLJ41484					2,52	2,12E-02
FLJ42875					4,92	2,38E-04
FLJ43663					2,32	1,39E-03
FLJ43860					4,34	5,95E-03
FLRT3	3762	fibronectin leucine rich t	ENSG00000125848	23767	4,05	4,85E-02
FMO1	3769	flavin containing monoo	ENSG00000010932	2326	6,38	5,57E-04
FMO2	3770	flavin containing monoo	ENSG00000094963	2327	8,48	6,73E-19
FMO3	3771	flavin containing monoo	ENSG00000007933	2328	8,18	7,46E-08
FMO4	3772	flavin containing monoo	ENSG00000076258	2329	2,50	2,58E-03
FMO5	3773	flavin containing monoo	ENSG00000131781	2330	2,33	2,61E-03
FMOD	3774	fibromodulin [Source:H	ENSG00000122176	2331	2,58	4,93E-02
FNDC1	21184	fibronectin type III dom	ENSG00000164694	84624	6,77	2,38E-04
FNDC5	20240	fibronectin type III dom	ENSG00000160097	252995	3,13	2,21E-02
FOLH1	3788	folate hydrolase (prosta	ENSG00000086205	2346	3,55	1,13E-02
FOLR2	3793	folate receptor 2 (fetal)	ENSG00000165457	2350	9,11	9,18E-10
FOS	3796	FBJ murine osteosarcon	ENSG00000170345	2353	8,52	3,04E-11
FOSB	3797	FBJ murine osteosarcon	ENSG00000125740	2354	9,17	2,14E-09
FP588					3,18	3,53E-02
FPR1	3826	formyl peptide receptor	ENSG00000171051	2357	4,32	1,27E-02

FPR3	3828	formyl peptide receptor	ENSG00000187474	2359	7,01	5,39E-06
FREM1	23399	FRAS1 related extracell	ENSG00000164946	158326	6,43	4,27E-06
FRK	3955	fyn-related kinase [Sou	ENSG00000111816	2444	5,61	5,57E-04
FRZB	3959	frizzled-related protein	ENSG00000162998	2487	6,82	9,29E-08
FXYD1	4025	FXYD domain containin	ENSG00000266964	5348	7,76	6,38E-12
FXYD6	4030	FXYD domain containin	ENSG00000137726	53826	9,26	2,24E-10
FYB	4036	FYN binding protein [So	ENSG00000082074	2533	5,67	6,69E-04
FZD10	4039	frizzled family receptor	ENSG00000111432	11211	7,23	2,34E-05
FZD3	4041	frizzled family receptor	ENSG00000104290	7976	3,21	1,06E-03
FZD7	4045	frizzled family receptor	ENSG00000155760	8324	4,49	4,66E-02
GABBR1	4070	gamma-aminobutyric a	ENSG00000232569	2550	2,27	4,10E-03
GABRB2	4082	gamma-aminobutyric a	ENSG00000145864	2561	3,84	6,59E-03
GAD1	4092	glutamate decarboxylas	ENSG00000128683	2571	6,34	1,14E-03
GADD45G	4097	growth arrest and DNA-	ENSG00000130222	10912	5,85	8,85E-05
GALM	24063	galactose mutarotase (a	ENSG00000143891	130589	2,11	1,13E-03
GALNT13	23242	UDP-N-acetyl-alpha-D-g	ENSG00000144278	114805	4,61	1,88E-02
GALNTL1					6,40	1,63E-07
GAPT	26588	GRB2-binding adaptor p	ENSG00000175857	202309	4,26	2,68E-02
GARNL3	25425	GTPase activating Rap/I	ENSG00000136895	84253	4,39	1,23E-07
GAS1	4165	growth arrest-specific 1	ENSG00000180447	2619	5,78	7,05E-18
GAS7	4169	growth arrest-specific 7	ENSG00000007237	8522	4,71	5,77E-04
GATA6	4174	GATA binding protein 6	ENSG00000141448	2627	3,83	3,20E-06
GATM	4175	glycine amidinotransfer	ENSG00000171766	2628	7,22	3,99E-14
GBP1P1	39561	guanylate binding prote	ENSG00000225492	400759	4,53	5,67E-04
GCK	4195	glucokinase (hexokinas	ENSG00000106633	2645	2,64	3,50E-02
GDAP1	15968	ganglioside induced diff	ENSG00000104381	54332	3,20	9,46E-05
GDF10	4215	growth differentiation fa	ENSG00000107623	2662	8,53	7,23E-09
GDF3	4218	growth differentiation fa	ENSG00000184344	9573	4,24	2,50E-03
GEM	4234	GTP binding protein ove	ENSG00000164949	2669	6,94	2,81E-12
GFRA2	4244	GDNF family receptor al	ENSG00000168546	2675	5,93	9,17E-07
GFRA3	4245	GDNF family receptor al	ENSG00000146013	2676	4,79	1,64E-04
GGTA1P	4253	glycoprotein, alpha-gala	ENSG00000204136	2681	4,47	6,80E-07
GJC2	17494	gap junction protein, ga	ENSG00000198835	57165	2,82	1,54E-02
GKAP1	17496	G kinase anchoring prot	ENSG00000165113	80318	2,10	3,67E-03
GLB1L2	25129	galactosidase, beta 1-lil	ENSG00000149328	89944	3,85	1,54E-04
GLDN	29514	gliomedin [Source:HGN	ENSG00000186417	342035	4,56	4,46E-02
GLI1	4317	GLI family zinc finger 1	ENSG00000111087	2735	3,70	5,34E-03
GLP1R	4324	glucagon-like peptide 1	ENSG00000112164	2740	4,76	7,33E-04
GLT8D2	24890	glycosyltransferase 8 do	ENSG00000120820	83468	3,94	1,69E-04
GLUL	4341	glutamate-ammonia liga	ENSG00000135821	2752	2,46	9,43E-03
GLYAT	13734	glycine-N-acyltransferas	ENSG00000149124	10249	4,97	3,04E-02
GLYATL2	24178	glycine-N-acyltransferas	ENSG00000156689	219970	4,03	2,57E-02
GNA15	4383	guanine nucleotide bind	ENSG00000060558	2769	3,90	9,55E-03
GNAL	4388	guanine nucleotide bind	ENSG00000141404	2774	6,86	1,80E-08
GNAO1	4389	guanine nucleotide bind	ENSG00000087258	2775	5,57	8,67E-06
GNAZ	4395	guanine nucleotide bind	ENSG00000128266	2781	3,07	4,52E-04
GNG2	4404	guanine nucleotide bind	ENSG00000186469	54331	3,09	6,44E-04
GNLY	4414	granulysin [Source:HGN	ENSG00000115523	10578	5,02	7,84E-04
GOLGA2P5					2,23	1,16E-02
GOLGA8B	31973	golgin A8 family, memb	ENSG00000215252	23015	3,09	3,93E-03
GP5	4443	glycoprotein V (platelet	ENSG00000178732	2814	3,38	1,02E-02
GPBAR1	19680	G protein-coupled bile a	ENSG00000179921	151306	3,71	9,77E-03
GPC3	4451	glypican 3 [Source:HGN	ENSG00000147257	2719	9,81	4,71E-17

GPC4	4452	glypican 4 [Source:HGN	ENSG00000076716	2239	4,92	3,47E-02
GPC6	4454	glypican 6 [Source:HGN	ENSG00000183098	10082	7,88	7,45E-08
GPD1	4455	glycerol-3-phosphate de	ENSG00000167588	2819	6,64	1,42E-02
GPER	4485	G protein-coupled estro	ENSG00000164850	2852	2,71	5,77E-03
GPLD1	4459	glycosylphosphatidylino	ENSG00000112293	2822	3,59	1,46E-03
GPM6A	4460	glycoprotein M6A [Sour	ENSG00000150625	2823	5,11	6,07E-11
GPM6B	4461	glycoprotein M6B [Sour	ENSG00000046653	2824	5,13	1,29E-08
GPNMB	4462	glycoprotein (transmem	ENSG00000136235	10457	5,67	5,13E-11
GPR123	13838	G protein-coupled recep	ENSG00000197177	84435	5,28	1,20E-03
GPR132	17482	G protein-coupled recep	ENSG00000183484	29933	3,14	2,26E-02
GPR133	19893	G protein-coupled recep	ENSG00000111452	283383	7,57	7,29E-10
GPR17	4471	G protein-coupled recep	ENSG00000144230	2840	5,48	3,83E-05
GPR182	13708	G protein-coupled recep	ENSG00000166856	11318	5,46	3,58E-02
GPR183	3128	G protein-coupled recep	ENSG00000169508	1880	7,62	5,49E-14
GPR21	4476	G protein-coupled recep	ENSG00000188394	2844	4,61	4,17E-04
GPR34	4490	G protein-coupled recep	ENSG00000171659	2857	6,98	1,84E-08
GPR35	4492	G protein-coupled recep	ENSG00000178623	2859	2,23	3,18E-02
GPR63	13302	G protein-coupled recep	ENSG00000112218	81491	2,24	2,57E-02
GPR64	4516	G protein-coupled recep	ENSG00000173698	10149	4,09	3,10E-02
GPRASP1	24834	G protein-coupled recep	ENSG00000198932	9737	4,64	9,51E-16
GPRC5C	13309	G protein-coupled recep	ENSG00000170412	55890	3,28	3,87E-02
GPT	4552	glutamic-pyruvate trans	ENSG00000167701	2875	2,17	4,92E-02
GPX3	4555	glutathione peroxidase	ENSG00000211445	2878	5,09	1,67E-05
GREB1	24885	growth regulation by es	ENSG00000196208	9687	5,71	2,33E-15
GREM2	17655	gremlin 2, DAN family E	ENSG00000180875	64388	3,37	3,06E-02
GRHL1	17923	grainyhead-like 1 (Dros	ENSG00000134317	29841	3,45	3,11E-02
GRIA1	4571	glutamate receptor, ion	ENSG00000155511	2890	4,63	2,97E-02
GRIA2	4572	glutamate receptor, ion	ENSG00000120251	2891	3,94	2,28E-02
GRIA3	4573	glutamate receptor, ion	ENSG00000125675	2892	7,10	4,52E-09
GRID1	4575	glutamate receptor, ion	ENSG00000182771	2894	5,43	1,27E-05
GRIK3	4581	glutamate receptor, ion	ENSG00000163873	2899	4,40	8,53E-03
GRIK5	4583	glutamate receptor, ion	ENSG00000105737	2901	4,23	9,00E-04
GRIN2A	4585	glutamate receptor, ion	ENSG00000183454	2903	6,05	1,96E-02
GRIN2B	4586	glutamate receptor, ion	ENSG00000150086	2904	4,57	2,01E-03
GRIN3A	16767	glutamate receptor, ion	ENSG00000198785	116443	2,17	3,31E-02
GRIP1	18708	glutamate receptor inte	ENSG00000155974	23426	2,70	2,26E-02
GRIP2	23841	glutamate receptor inte	ENSG00000144596		5,13	9,16E-05
GRM2	4594	glutamate receptor, me	ENSG00000164082	2912	4,28	1,86E-03
GSC	4612	goosecoid homeobox [S	ENSG00000133937	145258	5,85	2,07E-05
GSN	4620	gelsolin [Source:HGNC	ENSG00000148180	2934	3,76	2,30E-04
GSTA3	4628	glutathione S-transferas	ENSG00000174156	2940	3,69	2,49E-02
GSTM2	4634	glutathione S-transferas	ENSG00000213366	2946	2,67	8,36E-04
GSTM4	4636	glutathione S-transferas	ENSG00000168765	2948	2,07	5,18E-03
GSTM5	4637	glutathione S-transferas	ENSG00000134201	2949	9,42	5,54E-18
GTF2B	4648	general transcription fa	ENSG00000137947	2959	2,19	3,04E-04
GTF2IRD2	30775	GTF2I repeat domain cc	ENSG00000196275	84163	2,34	3,14E-03
GUCY1A2	4684	guanylate cyclase 1, sol	ENSG00000152402	2977	5,74	4,91E-04
GUCY1A3	4685	guanylate cyclase 1, sol	ENSG00000164116	2982	4,61	2,17E-09
GUCY1B3	4687	guanylate cyclase 1, sol	ENSG00000061918	2983	2,45	2,18E-05
GULP1	18649	GULP, engulfment adapt	ENSG00000144366	51454	2,12	1,58E-02
H19	4713	H19, imprinted materna	ENSG00000130600	283120	5,22	2,23E-04
HAAO	4796	3-hydroxyanthranilate 3	ENSG00000162882	23498	6,44	9,96E-10
HAND2	4808	heart and neural crest c	ENSG00000164107	9464	7,81	3,76E-05

HAPLN2	17410	hyaluronan and proteog	ENSG00000132702	60484	6,88	6,07E-05
HAS1	4818	hyaluronan synthase 1	ENSG00000105509	3036	7,01	1,55E-02
HAS3	4820	hyaluronan synthase 3	ENSG00000103044	3038	2,78	1,46E-02
HAVCR2	18437	hepatitis A virus cellular	ENSG00000135077	84868	4,53	2,02E-04
HBA1	4823	hemoglobin, alpha 1 [S	ENSG00000206172	3039	6,04	3,06E-03
HBA2	4824	hemoglobin, alpha 2 [S	ENSG00000188536	3039	7,81	4,00E-04
HBB	4827	hemoglobin, beta [Sour	ENSG00000244734	3043	8,33	8,21E-05
HCAR2	24827	hydroxycarboxylic acid	ENSG00000182782	338442	5,69	1,38E-02
HCG23	19713	HLA complex group 23	ENSG00000226228	414764	4,27	1,22E-03
HCG26					3,84	1,58E-03
HCG27	27366	HLA complex group 27	ENSG00000234079	253018	4,53	1,44E-04
HCK	4840	hemopoietic cell kinase	ENSG00000101336	3055	7,00	5,24E-06
HCP5	21659	HLA complex P5 (non-p	ENSG00000237105	10866	3,32	3,29E-04
HCST	16977	hematopoietic cell signa	ENSG00000126264	10870	4,77	1,08E-05
HDC	4855	histidine decarboxylase	ENSG00000140287	3067	6,26	5,25E-04
HENMT1	26400	HEN1 methyltransferase	ENSG00000162639	113802	2,60	3,98E-03
HERC5	24368	HECT and RLD domain	ENSG00000138646	51191	2,37	3,09E-03
HEXIM1	24953	hexamethylene bis-acet	ENSG00000186834	10614	2,01	1,75E-02
HEY2	4881	hairy/enhancer-of-split	ENSG00000135547	23493	4,17	5,73E-04
HEYL	4882	hairy/enhancer-of-split	ENSG00000163909	26508	4,38	2,80E-03
HFM1	20193	HFM1, ATP-dependent D	ENSG00000162669	164045	5,63	1,78E-06
HGF	4893	hepatocyte growth facto	ENSG00000019991	3082	2,58	6,27E-04
HHATL	13242	hedgehog acyltransferas	ENSG00000010282	57467	5,45	3,24E-03
HIC1	4909	hypermethylated in can	ENSG00000177374	3090	2,64	2,79E-02
HIF3A	15825	hypoxia inducible factor	ENSG00000124440	64344	3,06	1,97E-05
HIST1H1E	4718	histone cluster 1, H1e [	ENSG00000168298	3008	3,67	2,33E-02
HIST1H3A	4766	histone cluster 1, H3a [	ENSG00000198366	8350	3,57	2,35E-02
HK3	4925	hexokinase 3 (white cel	ENSG00000160883	3101	3,55	3,90E-03
HLA-DMA	4934	major histocompatibility	ENSG00000241394	3108	3,56	1,00E-03
HLA-DMB	4935	major histocompatibility	ENSG00000226264	3109	3,76	7,60E-06
HLA-DOA	4936	major histocompatibility	ENSG00000230141	3111	6,98	1,53E-05
HLA-DOB	4937	major histocompatibility	ENSG00000243496	3112	3,98	1,90E-02
HLA-DPA1	4938	major histocompatibility	ENSG00000168384	3113	6,32	1,36E-08
HLA-DPB1	4940	major histocompatibility	ENSG00000230763	3115	4,66	3,17E-06
HLA-DQA1	4942	major histocompatibility	ENSG00000236418	3117	7,80	3,59E-08
HLA-DQA2	4943	major histocompatibility	ENSG00000223793	3118	6,92	5,82E-03
HLA-DQB1	4944	major histocompatibility	ENSG00000233209	3119	9,14	8,82E-08
HLA-DQB2	4945	major histocompatibility	ENSG00000228813	3120	4,27	3,82E-02
HLA-DRA	4947	major histocompatibility	ENSG00000228987	3122	9,88	2,81E-14
HLA-DRB1	4948	major histocompatibility	ENSG00000236884	3123	6,21	4,27E-06
HLA-DRB5	4953	major histocompatibility	ENSG00000198502	3127	8,88	3,70E-04
HLA-DRB6	4954	major histocompatibility	ENSG00000229391	3128	7,18	1,42E-03
HLA-F	4963	major histocompatibility	ENSG00000229698	3134	3,60	1,98E-08
HLA-G	4964	major histocompatibility	ENSG00000233095	3135	4,59	3,15E-02
HLF	4977	hepatic leukemia factor	ENSG00000108924	3131	8,13	1,22E-19
HMGCLL1	21359	3-hydroxymethyl-3-met	ENSG00000146151	54511	6,32	1,80E-07
HMGN5	8013	high mobility group nuc	ENSG00000198157	79366	3,10	1,75E-05
HNMT	5028	histamine N-methyltran	ENSG00000150540	3176	4,99	8,97E-15
HOGA1	25155	4-hydroxy-2-oxoglutara	ENSG00000241935	112817	2,71	3,39E-02
HOOK1	19884	hook homolog 1 (Droso	ENSG00000134709	51361	5,03	8,07E-04
HOPX	24961	HOP homeobox [Source	ENSG00000171476	84525	3,07	2,31E-02
HOTAIR	33510	HOX transcript antisens	ENSG00000228630	100124700	5,59	3,59E-07
HOXC10	5122	homeobox C10 [Source	ENSG00000180818	3226	5,65	2,24E-07

HOXC11	5123	homeobox C11 [Source	ENSG00000123388	3227	3,08	8,21E-03
HOXC12	5124	homeobox C12 [Source	ENSG00000123407	3228	5,39	5,91E-04
HOXC4	5126	homeobox C4 [Source:I	ENSG00000198353	3221	5,77	4,22E-08
HOXC5	5127	homeobox C5 [Source:I	ENSG00000172789	3222	5,42	9,76E-06
HOXC6	5128	homeobox C6 [Source:I	ENSG00000197757	3223	3,92	9,30E-08
HOXC8	5129	homeobox C8 [Source:I	ENSG00000037965	3224	2,89	5,97E-04
HOXC9	5130	homeobox C9 [Source:I	ENSG00000180806	3225	2,63	3,81E-03
HPD	5147	4-hydroxyphenylpyruva	ENSG00000158104	3242	5,42	6,48E-04
HPGD	5154	hydroxyprostaglandin d	ENSG00000164120	3248	7,52	1,76E-09
HPGDS	17890	hematopoietic prostagla	ENSG00000163106	27306	7,01	5,38E-06
HPR	5156	haptoglobin-related pro	ENSG00000261701	3250	5,46	2,25E-04
HPSE2	18374	heparanase 2 [Source:I	ENSG00000172987	60495	6,03	3,21E-05
HRASLS5	24978	HRAS-like suppressor fa	ENSG00000168004	117245	6,01	1,10E-03
HRC	5178	histidine rich calcium bi	ENSG00000130528	3270	6,04	6,09E-07
HRCT1	33872	histidine rich carboxyl t	ENSG00000196196	646962	6,19	6,76E-06
HRH2	5183	histamine receptor H2 [	ENSG00000113749	3274	5,08	8,37E-04
HS3ST2	5195	heparan sulfate (glucos	ENSG00000122254	9956	5,34	2,68E-03
HS6ST3	19134	heparan sulfate 6-O-sul	ENSG00000185352	266722	3,54	3,74E-02
HSD11B1	5208	hydroxysteroid (11-beta	ENSG00000117594	3290	6,46	1,99E-06
HSD11B2	5209	hydroxysteroid (11-beta	ENSG00000176387	3291	4,22	1,45E-03
HSD17B11	22960	hydroxysteroid (17-beta	ENSG00000198189	51170	2,70	7,11E-06
HSD17B13	18685	hydroxysteroid (17-beta	ENSG00000170509	345275	4,60	5,08E-04
HSD17B6	23316	hydroxysteroid (17-beta	ENSG00000025423	8630	5,68	1,50E-08
HSF4	5227	heat shock transcriptior	ENSG00000102878	3299	3,64	4,78E-06
HSPA12A	19022	heat shock 70kDa prote	ENSG00000263052	259217	5,01	5,13E-05
HSPA12B	16193	heat shock 70kD proteii	ENSG00000132622	116835	2,04	2,35E-02
HSPA1B	5233	heat shock 70kDa prote	ENSG00000232804	3303	2,28	5,02E-03
HSPA2	5235	heat shock 70kDa prote	ENSG00000126803	3306	3,64	2,20E-02
HSPA6	5239	heat shock 70kDa prote	ENSG00000173110	3310	4,22	2,13E-02
HSPA7	5240	heat shock 70kDa prote	ENSG00000225217	3311	4,95	3,94E-03
HSPB2					4,18	4,08E-05
HSPB3	5248	heat shock 27kDa prote	ENSG00000169271	8988	9,56	2,94E-13
HSPB6	26511	heat shock protein, alpl	ENSG00000004776	126393	2,65	1,08E-02
HSPB7	5249	heat shock 27kDa prote	ENSG00000173641	27129	7,81	1,69E-09
HSPB8	30171	heat shock 22kDa prote	ENSG00000152137	26353	4,22	2,24E-03
HTR2A	5293	5-hydroxytryptamine (s	ENSG00000102468	3356	6,87	6,28E-06
HTR2B	5294	5-hydroxytryptamine (s	ENSG00000135914	3357	3,93	8,17E-03
HTR3C	24003	5-hydroxytryptamine (s	ENSG00000178084	170572	3,53	2,29E-02
HUNK	13326	hormonally up-regulate	ENSG00000142149	30811	4,78	1,90E-03
HVCN1	28240	hydrogen voltage-gated	ENSG00000122986	84329	2,62	3,59E-04
HYAL1	5320	hyaluronoglucosaminida	ENSG00000262208	3373	2,48	3,08E-05
HYDIN	19368	HYDIN, axonemal centr	ENSG00000157423	54768	4,12	2,96E-02
HYMAI					3,39	1,02E-02
ICAM4	5347	intercellular adhesion m	ENSG00000105371	3386	5,27	8,47E-03
ID2	5361	inhibitor of DNA binding	ENSG00000115738	3398	4,29	1,35E-07
ID2B					3,99	2,36E-04
ID4	5363	inhibitor of DNA binding	ENSG00000172201	3400	6,09	9,91E-08
IDI2-AS1	30885	IDI2 antisense RNA 1 [S	ENSG00000232656	55853	2,81	3,03E-02
IER2	28871	immediate early respon	ENSG00000160888	9592	2,16	6,45E-04
IER5	5393	immediate early respon	ENSG00000162783	51278	2,14	5,74E-03
IFI44L	17817	interferon-induced prote	ENSG00000137959	10964	2,30	2,79E-02
IFIT1	5407	interferon-induced prote	ENSG00000185745	3434	4,96	3,48E-05
IFIT3	5411	interferon-induced prote	ENSG00000119917	3437	2,75	2,05E-04

IFITM1	5412	interferon induced trans	ENSG00000185885	8519	3,91	5,30E-11
IGDCC4	13770	immunoglobulin superfa	ENSG00000103742	57722	4,41	1,82E-04
IGF1	5464	insulin-like growth facto	ENSG00000017427	3479	7,50	5,30E-05
IGFBP5	5474	insulin-like growth facto	ENSG00000115461	3488	8,89	1,77E-03
IGFBP6	5475	insulin-like growth facto	ENSG00000167779	3489	3,24	4,64E-03
IGFBPL1	20081	insulin-like growth facto	ENSG00000137142	347252	4,29	2,43E-03
IGFN1	24607	immunoglobulin-like an	ENSG00000163395	91156	5,90	1,90E-04
IGJ	5713	immunoglobulin J polyp	ENSG00000132465	3512	5,01	1,82E-02
IGSF10	26384	immunoglobulin superfa	ENSG00000152580	285313	6,26	3,11E-10
IGSF21	28246	immunoglobin superfam	ENSG00000117154	84966	5,50	1,88E-03
IGSF6	5953	immunoglobulin superfa	ENSG00000140749	10261	3,27	2,72E-02
IGSF9B	32326	immunoglobulin superfa	ENSG00000080854	22997	4,87	2,62E-02
IKZF1	13176	IKAROS family zinc fing	ENSG00000185811	10320	5,25	1,77E-05
IL10	5962	interleukin 10 [Source:I	ENSG00000136634	3586	4,73	1,22E-03
IL10RA	5964	interleukin 10 receptor,	ENSG00000110324	3587	7,32	7,12E-07
IL12RB2	5972	interleukin 12 receptor,	ENSG00000081985	3595	3,21	1,80E-02
IL16	5980	interleukin 16 [Source:I	ENSG00000172349	3603	4,85	1,17E-04
IL17B	5982	interleukin 17B [Source	ENSG00000127743	27190	5,86	7,77E-04
IL17RD	17616	interleukin 17 receptor	ENSG00000144730	54756	3,80	1,09E-02
IL17RE	18439	interleukin 17 receptor	ENSG00000163701	132014	3,53	5,11E-04
IL18	5986	interleukin 18 (interfero	ENSG00000150782	3606	4,59	8,22E-04
IL18RAP	5989	interleukin 18 receptor	ENSG00000115607	8807	5,66	1,31E-02
IL1B	5992	interleukin 1, beta [Sou	ENSG00000125538	3553	5,42	1,81E-02
IL1R2	5994	interleukin 1 receptor, t	ENSG00000115590	7850	4,51	4,95E-02
IL1RL2	5999	interleukin 1 receptor-li	ENSG00000115598	8808	3,17	3,37E-02
IL1RN	6000	interleukin 1 receptor a	ENSG00000136689	3557	5,63	7,96E-03
IL28RA					4,76	6,71E-04
IL2RG	6010	interleukin 2 receptor, g	ENSG00000147168	3561	2,94	1,91E-02
IL33	16028	interleukin 33 [Source:I	ENSG00000137033	90865	4,59	2,28E-02
IL6	6018	interleukin 6 (interferon	ENSG00000136244	3569	5,72	4,34E-02
IMPA2	6051	inositol(myo)-1(or 4)-m	ENSG00000141401	3613	3,14	5,84E-03
INHBB	6067	inhibin, beta B [Source:	ENSG00000163083	3625	3,02	4,19E-02
INMT	6069	indolethylamine N-meth	ENSG00000241644	11185	8,65	5,36E-11
IPCEF1	21204	interaction protein for c	ENSG00000074706	26034	2,35	2,50E-02
IQGAP2	6111	IQ motif containing GTP	ENSG00000145703	10788	2,60	3,51E-03
IQSEC3	29193	IQ motif and Sec7 doma	ENSG00000261772	440073	3,38	2,77E-02
IRF4	6119	interferon regulatory fa	ENSG00000137265	3662	5,26	1,65E-03
IRF5	6120	interferon regulatory fa	ENSG00000128604	3663	4,17	1,36E-03
IRF8	5358	interferon regulatory fa	ENSG00000140968	3394	3,17	4,07E-02
IRX3	14360	iroquois homeobox 3 [S	ENSG00000177508	79191	2,14	4,07E-02
IRX6	14675	iroquois homeobox 6 [S	ENSG00000159387	79190	6,22	2,62E-05
ISLR	6133	immunoglobulin superfa	ENSG00000129009	3671	5,54	1,95E-10
ISLR2	29286	immunoglobulin superfa	ENSG00000167178	57611	5,87	1,48E-06
ISM1	16213	isthmin 1, angiogenesis	ENSG00000101230	140862	6,58	2,78E-07
ITGA2B	6138	integrin, alpha 2b (plate	ENSG00000005961	3674	3,00	4,48E-03
ITGA7	6143	integrin, alpha 7 [Sourc	ENSG00000135424	3679	8,11	2,23E-12
ITGA8	6144	integrin, alpha 8 [Sourc	ENSG00000077943	8516	8,04	4,08E-05
ITGA9	6145	integrin, alpha 9 [Sourc	ENSG00000144668	3680	2,92	3,22E-06
ITGAM	6149	integrin, alpha M (comp	ENSG00000169896	3684	5,39	1,68E-05
ITGB1BP2	6154	integrin beta 1 binding	ENSG00000147166	26548	4,20	5,08E-04
ITGB2	6155	integrin, beta 2 (comple	ENSG00000160255	3689	4,60	1,07E-05
ITIH1	6166	inter-alpha-trypsin inhit	ENSG00000055957	3697	5,53	2,30E-03
ITIH3	6168	inter-alpha-trypsin inhit	ENSG00000162267	3699	5,97	5,03E-05

ITIH4	6169	inter-alpha-trypsin inhil	ENSG00000055955	3700	6,46	8,15E-07
ITIH5	21449	inter-alpha-trypsin inhil	ENSG00000123243	80760	8,88	1,89E-14
ITM2A	6173	integral membrane prot	ENSG00000078596	9452	3,12	8,09E-03
ITPR1	6180	inositol 1,4,5-trisphospl	ENSG00000150995	3708	2,22	1,04E-02
JAKMIP3	23523	Janus kinase and micro	ENSG00000188385	282973	2,79	2,96E-02
JAM2	14686	junctional adhesion mol	ENSG00000154721	58494	3,83	1,13E-03
JPH2	14202	junctophilin 2 [Source:I	ENSG00000149596	57158	6,65	8,76E-05
JUN	6204	jun proto-oncogene [So	ENSG00000177606	3725	3,11	1,61E-04
JUNB	6205	jun B proto-oncogene [!	ENSG00000171223	3726	4,93	2,36E-06
JUND	6206	jun D proto-oncogene [	ENSG00000130522	3727	2,50	6,05E-03
KANK2	29300	KN motif and ankyrin re	ENSG00000197256	25959	2,16	1,86E-03
KANK4	27263	KN motif and ankyrin re	ENSG00000132854	163782	4,14	4,07E-02
KAZALD1	25460	Kazal-type serine peptic	ENSG00000107821	81621	2,66	3,56E-02
KBTBD12	25731	kelch repeat and BTB (F	ENSG00000187715	166348	3,20	2,23E-02
KCNA5	6224	potassium voltage-gate	ENSG00000130037	3741	6,91	9,02E-04
KCNA6	6225	potassium voltage-gate	ENSG00000151079	3742	4,69	2,26E-04
KCNAB1	6228	potassium voltage-gate	ENSG00000169282	7881	6,52	1,94E-07
KCNB1	6231	potassium voltage-gate	ENSG00000158445	3745	5,12	6,73E-03
KCND2	6238	potassium voltage-gate	ENSG00000184408	3751	3,86	4,91E-02
KCND3	6239	potassium voltage-gate	ENSG00000171385	3752	2,85	3,30E-02
KCNH2	6251	potassium voltage-gate	ENSG00000055118	3757	6,80	3,14E-06
KCNIP3	15523	Kv channel interacting p	ENSG00000115041	30818	2,61	4,30E-03
KCNJ1	6255	potassium inwardly-rect	ENSG00000151704	3758	4,98	2,61E-03
KCNJ12	6258	potassium inwardly-rect	ENSG00000184185	3768	3,75	1,78E-05
KCNJ3	6264	potassium inwardly-rect	ENSG00000162989	3760	4,99	3,27E-03
KCNJ8	6269	potassium inwardly-rect	ENSG00000121361	3764	9,21	2,46E-18
KCNK1	6272	potassium channel, sub	ENSG00000135750	3775	3,73	1,58E-04
KCNK15	13814	potassium channel, sub	ENSG00000124249	60598	4,31	9,34E-03
KCNK2	6277	potassium channel, sub	ENSG00000082482	3776	3,64	2,97E-02
KCNK3	6278	potassium channel, sub	ENSG00000171303	3777	5,66	7,66E-04
KCNK5	6280	potassium channel, sub	ENSG00000164626	8645	6,49	3,18E-04
KCNK7	6282	potassium channel, sub	ENSG00000173338	10089	4,32	1,02E-02
KCNMA1	6284	potassium large conduc	ENSG00000156113	3778	3,69	6,42E-05
KCNMB1	6285	potassium large conduc	ENSG00000145936	3779	9,25	1,26E-07
KCNN3	6292	potassium intermediate	ENSG00000143603	3782	2,52	1,21E-03
KCNQ1	6294	potassium voltage-gate	ENSG00000053918	3784	3,57	1,53E-03
KCNQ4	6298	potassium voltage-gate	ENSG00000117013	9132	4,53	8,75E-05
KCNS2	6301	potassium voltage-gate	ENSG00000156486	3788	5,53	7,07E-06
KCP	17585	kielin/chordin-like prote	ENSG00000135253		5,54	1,43E-03
KHK	6315	ketohexokinase (fructok	ENSG00000138030	3795	2,59	6,38E-05
KIAA0226L	20420	KIAA0226-like [Source:	ENSG00000102445	80183	3,81	4,32E-02
KIAA0748					4,27	3,41E-03
KIAA1045	29180	KIAA1045 [Source:HGN	ENSG00000122733	23349	3,47	1,36E-02
KIAA1377	29264	KIAA1377 [Source:HGN	ENSG00000110318	57562	2,75	4,77E-04
KIAA1644	29335	KIAA1644 [Source:HGN	ENSG00000138944	85352	4,71	6,47E-04
KIAA1683	29350	KIAA1683 [Source:HGN	ENSG00000130518	80726	3,83	1,90E-04
KIAA2022	29433	KIAA2022 [Source:HGN	ENSG00000050030	340533	4,46	5,12E-04
KIF26B	25484	kinesin family member	ENSG00000162849	55083	2,22	2,14E-02
KIF5C	6325	kinesin family member	ENSG00000168280	3800	2,67	4,05E-02
KL	6344	klotho [Source:HGNC S	ENSG00000133116	9365	5,26	2,39E-06
KLB	15527	klotho beta [Source:HG	ENSG00000134962	152831	4,78	2,44E-02
KLF11	11811	Kruppel-like factor 11 [!	ENSG00000172059	8462	2,99	1,73E-07
KLF2	6347	Kruppel-like factor 2 (lu	ENSG00000127528	10365	4,80	3,38E-05

KLF4	6348	Kruppel-like factor 4 (gı	ENSG00000136826	9314	5,74	2,51E-09
KLF8	6351	Kruppel-like factor 8 [S	ENSG00000102349	11279	5,44	9,13E-10
KLF9	1123	Kruppel-like factor 9 [S	ENSG00000119138	687	2,43	2,79E-02
KLHDC1	19836	kelch domain containing	ENSG00000197776	122773	2,03	7,74E-05
KLHL30	24770	kelch-like family membe	ENSG00000168427	377007	5,33	3,01E-04
KLHL4	6355	kelch-like family membe	ENSG00000102271	56062	2,28	2,55E-02
KLKB1	6371	kallikrein B, plasma (Fle	ENSG00000164344	3818	4,69	1,51E-04
KLRG1	6380	killer cell lectin-like rece	ENSG00000139187	10219	2,10	2,23E-02
KRBOX1	38708	KRAB box domain conta	ENSG00000240747	100506243	2,91	3,99E-02
KREMEN1	17550	kringle containing trans	ENSG00000183762	83999	2,32	1,33E-02
KRT222	28695	keratin 222 [Source:HG	ENSG00000213424	125113	4,98	2,08E-03
KY	26576	kyphoscoliosis peptidas	ENSG00000174611	339855	3,30	1,01E-02
KYNU	6469	kynureninase [Source:H	ENSG00000115919	8942	3,98	6,22E-03
LAG3	6476	lymphocyte-activation g	ENSG00000089692	3902	3,86	6,11E-03
LAIR1	6477	leukocyte-associated im	ENSG00000167613	3903	5,86	1,11E-05
LAMA2	6482	laminin, alpha 2 [Sourc	ENSG00000196569	3908	2,65	1,06E-05
LARP7	24912	La ribonucleoprotein do	ENSG00000174720	51574	2,83	1,67E-11
LCN10	20892	lipocalin 10 [Source:HG	ENSG00000187922	414332	5,88	1,68E-02
LCN6	17337	lipocalin 6 [Source:HGN	ENSG00000267206	158062	4,76	1,53E-02
LCP1	6528	lymphocyte cytosolic pr	ENSG00000136167	3936	5,20	5,38E-04
LCP2	6529	lymphocyte cytosolic pr	ENSG00000043462	3937	5,13	4,55E-05
LDB3	15710	LIM domain binding 3 [S	ENSG00000122367	11155	6,44	3,32E-10
LDLRAD2	32071	low density lipoprotein r	ENSG00000187942	401944	2,15	1,27E-02
LEFTY2	3122	left-right determination	ENSG00000143768	7044	5,29	2,33E-05
LEP	6553	leptin [Source:HGNC Sy	ENSG00000174697	3952	8,42	1,05E-02
LGALS12	15788	lectin, galactoside-bindi	ENSG00000133317	85329	5,86	1,39E-02
LGALS4	6565	lectin, galactoside-bindi	ENSG00000171747	3960	3,19	5,43E-03
LGALSL	25012	lectin, galactoside-bindi	ENSG00000119862	29094	2,26	9,85E-06
LGI1	6572	leucine-rich, glioma ina	ENSG00000108231	9211	7,16	1,80E-10
LGI2	18710	leucine-rich repeat LGI	ENSG00000153012	55203	3,67	6,20E-04
LGI3	18711	leucine-rich repeat LGI	ENSG00000168481	203190	3,43	3,34E-02
LGI4	18712	leucine-rich repeat LGI	ENSG00000153902	163175	7,11	2,08E-17
LGR5	4504	leucine-rich repeat cont	ENSG00000139292	8549	4,70	3,37E-02
LHFP	6586	lipoma HMGIC fusion pa	ENSG00000183722	10186	2,27	3,83E-05
LIFR	6597	leukemia inhibitory fact	ENSG00000113594	3977	2,79	1,55E-02
LILRA2	6603	leukocyte immunoglobu	ENSG00000239998	11027	3,55	3,75E-02
LILRA5	16309	leukocyte immunoglobu	ENSG00000187116	353514	3,95	2,01E-02
LILRA6	15495	leukocyte immunoglobu	ENSG00000244482	79168	2,43	4,70E-02
LILRB2	6606	leukocyte immunoglobu	ENSG00000131042	10288	5,37	1,01E-05
LILRB4	6608	leukocyte immunoglobu	ENSG00000186818	11006	4,70	4,43E-04
LILRB5	6609	leukocyte immunoglobu	ENSG00000105609	10990	6,76	4,39E-06
LIMS2	16084	LIM and senescent cell a	ENSG00000072163	55679	2,77	9,50E-06
LINC00087	34500	long intergenic non-prot	ENSG00000196972	644596	2,88	6,99E-03
LINC00173	33791	long intergenic non-prot	ENSG00000196668	100287569	4,19	1,15E-04
LINC00260					2,38	4,97E-02
LINC00265	28019	long intergenic non-prot	ENSG00000188185	349114	2,49	5,83E-03
LINC00273	38595	long intergenic non-prot	ENSG00000256642	649159	6,32	1,18E-02
LINC00310	16414	long intergenic non-prot	ENSG00000227456	114036	4,92	5,02E-05
LINC00312	6662	long intergenic non-prot	ENSG00000237697	29931	5,43	2,60E-05
LINC00324	26628	long intergenic non-prot	ENSG00000178977	284029	2,20	2,32E-02
LINC00341	20353	long intergenic non-prot	ENSG00000229645	79686	2,27	1,09E-03
LINGO3	21206	leucine rich repeat and	ENSG00000220008	645191	4,20	2,49E-02
LIPC	6619	lipase, hepatic [Source:	ENSG00000166035	3990	3,20	4,36E-02

LITAF	16841	lipopolysaccharide-indu	ENSG00000189067	9516	3,27	2,13E-06
LMCD1	6633	LIM and cysteine-rich d	ENSG00000071282	29995	2,33	2,63E-03
LMO3	6643	LIM domain only 3 (rho	ENSG00000048540	55885	8,15	1,52E-08
LMOD1	6647	leiomodin 1 (smooth mu	ENSG00000163431	25802	8,57	3,10E-16
LMX1A	6653	LIM homeobox transcrip	ENSG00000162761	4009	5,40	3,52E-04
LMX1B	6654	LIM homeobox transcrip	ENSG00000136944	4010	3,16	3,35E-02
LNP1	28014	leukemia NUP98 fusion	ENSG00000206535	348801	5,94	5,13E-07
LNX1	6657	ligand of numb-protein	ENSG00000072201	84708	2,74	5,80E-05
LOC100127888					3,53	2,59E-02
LOC100127983					2,78	3,26E-04
LOC100128164					2,59	3,13E-03
LOC100129269					5,17	2,26E-03
LOC100130992					2,99	5,78E-04
LOC100131434					2,48	6,55E-03
LOC100132891					3,76	1,14E-07
LOC100216545					2,15	4,12E-04
LOC100233209					4,20	6,98E-04
LOC100240735					4,08	3,85E-03
LOC100270746					2,76	2,56E-02
LOC100287846					4,13	7,13E-04
LOC100288122					2,23	4,71E-02
LOC100289187					2,01	3,56E-02
LOC100289361					2,70	1,41E-02
LOC100289495					2,05	3,36E-02
LOC100289511					2,37	2,63E-02
LOC100294362					2,38	2,28E-02
LOC100302640					3,38	1,53E-03
LOC100302650					5,34	1,18E-02
LOC100505483					4,75	1,40E-06
LOC100505633					3,48	2,28E-02
LOC100505718					3,44	8,51E-03
LOC100505865					2,33	3,86E-03
LOC100505875					4,83	1,43E-04
LOC100505933					3,26	8,24E-03
LOC100506035					5,74	6,29E-05
LOC100506368					2,22	1,34E-02
LOC100506388					5,12	1,12E-04
LOC100506421					4,80	1,25E-02
LOC100506497					4,49	1,09E-02
LOC100506779					4,31	9,42E-04
LOC100506795					3,03	2,37E-03
LOC100507053					3,38	2,66E-02
LOC100507331					2,20	4,58E-02
LOC100507410					3,03	1,82E-02
LOC100507463					3,54	1,90E-04
LOC100507632					4,85	1,71E-02
LOC100616668					2,43	1,05E-02
LOC100652768					2,24	2,68E-02
LOC113230					2,64	4,36E-02
LOC144571					3,06	1,04E-05
LOC145820					7,51	1,11E-09
LOC154822					2,84	3,88E-02
LOC221442					2,97	4,88E-03

LOC255167					3,60	1,44E-02
LOC283174					6,06	5,99E-03
LOC283335					4,21	1,95E-08
LOC283392					5,45	4,91E-05
LOC283481					2,87	1,35E-04
LOC284276					4,81	5,92E-05
LOC284648					5,17	7,18E-06
LOC284801					6,27	3,35E-02
LOC284837					3,03	2,96E-03
LOC285419					2,72	2,59E-02
LOC286367					3,34	5,42E-04
LOC338758					3,31	5,47E-05
LOC339524					6,39	8,51E-11
LOC339535					3,32	4,70E-02
LOC375010					4,03	8,87E-03
LOC388630					6,87	1,37E-06
LOC400043					3,86	4,41E-05
LOC401093					3,52	5,69E-08
LOC441461					2,67	1,99E-02
LOC572558					3,48	3,94E-03
LOC642846					2,53	3,06E-02
LOC644554					3,41	9,33E-04
LOC644990					4,18	2,06E-03
LOC650368					4,82	3,87E-04
LOC728819					3,03	3,95E-03
LOC729950					3,67	8,05E-03
LOC730102					2,13	2,01E-03
LOH12CR2	26524	loss of heterozygosity, 1	ENSG00000205791		2,87	9,07E-03
LONRF1	26302	LON peptidase N-termir	ENSG00000154359	91694	2,32	2,39E-02
LONRF2	24788	LON peptidase N-termir	ENSG00000170500	164832	8,61	6,66E-08
LOXHD1	26521	lipoxygenase homology	ENSG00000167210	125336	3,71	3,36E-02
LPHN3	20974	latrophilin 3 [Source:HC	ENSG00000262515	23284	7,56	8,11E-06
LPL	6677	lipoprotein lipase [Sour	ENSG00000175445	4023	6,67	2,32E-02
LPPR4					5,70	1,77E-08
LRFN5	20360	leucine rich repeat and	ENSG00000165379	145581	4,00	1,73E-04
LRRC16A	21581	leucine rich repeat cont	ENSG00000079691	55604	2,01	8,94E-03
LRRC16B	20272	leucine rich repeat cont	ENSG00000186648	90668	3,34	1,74E-02
LRRC17	16895	leucine rich repeat cont	ENSG00000128606	10234	4,51	6,49E-11
LRRC2	14676	leucine rich repeat cont	ENSG00000163827	79442	4,26	1,14E-03
LRRC25	29806	leucine rich repeat cont	ENSG00000175489	126364	5,45	7,81E-06
LRRC34	28408	leucine rich repeat cont	ENSG00000171757	151827	3,18	6,28E-04
LRRC4	15586	leucine rich repeat cont	ENSG00000128594	64101	3,02	2,15E-02
LRRC4B	25042	leucine rich repeat cont	ENSG00000131409	94030	3,35	4,61E-02
LRRC4C	29317	leucine rich repeat cont	ENSG00000148948	57689	4,40	1,97E-03
LRRC7	18531	leucine rich repeat cont	ENSG00000033122	57554	3,88	1,38E-02
LRRC70	35155	leucine rich repeat cont	ENSG00000186105	100130733	2,25	3,58E-03
LRRCC1	29373	leucine rich repeat and	ENSG00000133739	85444	4,35	3,03E-11
LRRK2	18618	leucine-rich repeat kina	ENSG00000188906	120892	4,87	3,74E-12
LRRN1	20980	leucine rich repeat neur	ENSG00000175928	57633	4,85	2,25E-03
LRRN3	17200	leucine rich repeat neur	ENSG00000173114	54674	4,16	1,11E-02
LRRN4CL	33724	LRRN4 C-terminal like [	ENSG00000177363	221091	7,91	9,26E-09
LSAMP	6705	limbic system-associate	ENSG00000185565	4045	8,09	2,07E-09
LSP1	6707	lymphocyte-specific pro	ENSG00000130592	4046	8,63	3,45E-15

LST1	14189	leukocyte specific trans	ENSG00000206433	7940	4,34	1,40E-02
LTBP4	6717	latent transforming gro	ENSG00000090006	8425	3,08	2,57E-02
LTC4S	6719	leukotriene C4 synthase	ENSG00000213316	4056	4,37	1,95E-03
LTF	6720	lactotransferrin [Source	ENSG00000012223	4057	3,34	1,13E-03
LUM	6724	lumican [Source:HGNC	ENSG00000139329	4060	7,65	2,17E-12
LUZP2	23206	leucine zipper protein 2	ENSG00000187398	338645	4,49	2,35E-02
LY86	16837	lymphocyte antigen 86	ENSG00000112799	9450	4,43	9,09E-04
LYNX1	29604	Ly6/neurotoxin 1 [Sour	ENSG00000180155	66004	2,20	9,46E-03
LYST	1968	lysosomal trafficking re	ENSG00000143669	1130	2,14	1,63E-03
LYZ	6740	lysozyme [Source:HGN	ENSG00000090382	4069	8,49	2,37E-05
MACROD2	16126	MACRO domain contain	ENSG00000172264	140733	3,49	7,40E-05
MAF	6776	v-maf avian musculoap	ENSG00000178573	4094	2,35	2,27E-02
MAFB	6408	v-maf avian musculoap	ENSG00000204103	9935	3,14	5,84E-03
MAGI2	18957	membrane associated g	ENSG00000187391	9863	2,63	8,83E-05
MAK	6816	male germ cell-associat	ENSG00000111837	4117	2,78	2,49E-03
MAL	6817	mal, T-cell differentiatio	ENSG00000172005	4118	5,00	3,10E-03
MALT1	6819	mucosa associated lym	ENSG00000172175	10892	2,01	2,92E-02
MAOB	6834	monoamine oxidase B [	ENSG00000069535	4129	9,41	3,70E-15
MAP3K8	6860	mitogen-activated prote	ENSG00000107968	1326	5,16	1,30E-05
MAP6	6868	microtubule-associated	ENSG00000171533	4135	3,75	7,04E-03
MAP7	6869	microtubule-associated	ENSG00000135525	9053	3,07	7,91E-05
MAPK10	6872	mitogen-activated prote	ENSG00000109339	5602	6,29	2,16E-05
MAPK4	6878	mitogen-activated prote	ENSG00000141639	5596	7,64	3,39E-07
MAPT	6893	microtubule-associated	ENSG00000186868	4137	4,01	1,65E-03
MARCO	6895	macrophage receptor w	ENSG00000019169	8685	4,81	5,52E-03
MARK1	6896	MAP/microtubule affinit	ENSG00000116141	4139	2,11	1,06E-02
MASP1	6901	mannan-binding lectin s	ENSG00000127241	5648	4,39	1,23E-03
MATN2	6908	matrilin 2 [Source:HGN	ENSG00000132561	4147	4,99	8,87E-04
MATN4	6910	matrilin 4 [Source:HGN	ENSG00000124159	8785	3,74	7,72E-03
MBOAT1	21579	membrane bound O-acy	ENSG00000172197	154141	3,50	1,35E-06
MBOAT4	32311	membrane bound O-acy	ENSG00000177669	619373	3,16	1,93E-02
MCF2L	14576	MCF.2 cell line derived t	ENSG00000126217	23263	3,37	1,60E-04
MCOLN3	13358	mucolipin 3 [Source:HG	ENSG00000055732	55283	5,39	2,78E-06
MCTP2	25636	multiple C2 domains, tr	ENSG00000140563	55784	3,70	6,07E-05
MDFIC	28870	MyoD family inhibitor d	ENSG00000135272	29969	2,44	9,69E-03
MDGA1	19267	MAM domain containing	ENSG00000112139	266727	2,58	2,08E-02
MDH1B	17836	malate dehydrogenase	ENSG00000138400	130752	2,10	4,83E-02
MEFV	6998	Mediterranean fever [S	ENSG00000103313	4210	3,68	3,12E-02
MEOX1	7013	mesenchyme homeobox	ENSG00000005102	4222	2,17	4,17E-02
METTL24	21566	methyltransferase like 2	ENSG00000053328	728464	5,27	2,12E-02
METTL7A	24550	methyltransferase like 7	ENSG00000185432	25840	2,86	2,51E-04
MFAP4	7035	microfibrillar-associated	ENSG00000166482	4239	8,15	2,20E-19
MFAP5	29673	microfibrillar associated	ENSG00000197614	8076	9,20	1,34E-02
MFSD4	25433	major facilitator superfa	ENSG00000174514	148808	2,75	1,63E-02
MGAM	7043	maltase-glucoamylase (	ENSG00000257335	8972	4,42	4,19E-02
MGAT3	7046	mannosyl (beta-1,4-)-g	ENSG00000128268	4248	5,79	2,52E-05
MGAT4C	30871	mannosyl (alpha-1,3-)-	ENSG00000182050	25834	4,98	9,50E-03
MIAT	33425	myocardial infarction as	ENSG00000225783	440823	4,74	2,33E-02
MIR143HG	42872	MIR143 host gene (non	ENSG00000249669		4,68	4,35E-02
MIR27A	31613	microRNA 27a [Source:	ENSG00000207808	407018	3,01	2,54E-02
MIR3648	38941	microRNA 3648 [Source	ENSG00000264462	100500862	6,39	2,51E-02
MIR3687	38946	microRNA 3687 [Source	ENSG00000264063	100500815	7,12	3,44E-02
MIR4466	41726	microRNA 4466 [Source	ENSG00000271899	100616154	6,32	4,69E-02

MIR497HG	39523	mir-497-195 cluster ho:	ENSG00000267532	406971	3,52	1,83E-05
MIR568	32824	microRNA 568 [Source:	ENSG00000207770	693153	2,70	4,13E-03
MIR663A	32919	microRNA 663a [Source	ENSG00000227195	284801	7,93	7,01E-03
MITF	7105	microphthalmia-associa	ENSG00000187098	4286	2,65	5,35E-09
MKX	23729	mohawk homeobox [So	ENSG00000150051	283078	3,15	4,29E-03
MLF1	7125	myeloid leukemia factor	ENSG00000178053	4291	2,18	3,37E-03
MLXIPL	12744	MLX interacting protein-	ENSG00000009950	51085	7,17	3,79E-03
MMP23B	7171	matrix metallopeptidase	ENSG00000189409	8510	3,83	2,07E-02
MMP27	14250	matrix metallopeptidase	ENSG00000137675	64066	4,65	1,35E-02
MMP9	7176	matrix metallopeptidase	ENSG00000100985	4318	4,66	1,82E-02
MNDA	7183	myeloid cell nuclear diff	ENSG00000163563	4332	7,75	1,68E-04
MNS1	29636	meiosis-specific nuclear	ENSG00000138587	55329	3,98	3,61E-04
MOB3B	23825	MOB kinase activator 3I	ENSG00000120162	79817	2,50	1,46E-02
MPEG1	29619	macrophage expressed	ENSG00000197629	219972	6,97	2,20E-08
MPL	7217	myeloproliferative leuke	ENSG00000117400	4352	3,04	4,16E-04
MPP2	7220	membrane protein, palr	ENSG00000108852	4355	2,89	1,62E-03
MPP7	26542	membrane protein, palr	ENSG00000150054	143098	4,10	2,33E-04
MPPED2	1180	metallophosphoesterase	ENSG00000066382	744	2,90	1,18E-02
MPV17L	26827	MPV17 mitochondrial m	ENSG00000156968	255027	3,45	2,00E-03
MRGPRF	24828	MAS-related GPR, mem	ENSG00000172935	116535	8,12	2,19E-12
MRO	24121	maestro [Source:HGNC	ENSG00000134042	83876	4,03	1,34E-03
MRVI1	7237	murine retrovirus integ	ENSG00000072952	10335	6,44	4,27E-07
MS4A14	30706	membrane-spanning 4-	ENSG00000166928	84689	5,02	1,34E-03
MS4A2	7316	membrane-spanning 4-	ENSG00000149534	2206	6,04	2,61E-03
MS4A4A	13371	membrane-spanning 4-	ENSG00000110079	51338	7,65	2,19E-07
MS4A6A	13375	membrane-spanning 4-	ENSG00000110077	64231	3,46	2,31E-04
MS4A7	13378	membrane-spanning 4-	ENSG00000166927	58475	8,62	4,52E-14
MSC	7321	musculin [Source:HGN(	ENSG00000178860	9242	4,52	6,47E-04
MSR1	7376	macrophage scavenger	ENSG00000038945	4481	3,16	5,42E-03
MST4					2,92	4,59E-05
MSTN	4223	myostatin [Source:HGN	ENSG00000138379	2660	4,40	2,21E-04
MSX1	7391	msh homeobox 1 [Sour	ENSG00000163132	4487	3,57	4,59E-03
MSX2	7392	msh homeobox 2 [Sour	ENSG00000120149	4488	2,59	3,77E-02
MT1A	7393	metallothionein 1A [Sou	ENSG00000205362	4489	7,30	2,27E-02
MT1F	7398	metallothionein 1F [Sou	ENSG00000198417	4494	3,03	3,42E-03
MT1JP	7402	metallothionein 1J, pse	ENSG00000255986	4498	5,00	1,32E-02
MT1M	14296	metallothionein 1M [So	ENSG00000205364	4499	8,26	4,81E-03
MT1X	7405	metallothionein 1X [Sou	ENSG00000187193	4501	5,25	3,81E-02
MTMR8	16825	myotubularin related pr	ENSG00000102043	55613	2,40	7,79E-03
MTUS2	20595	microtubule associated	ENSG00000132938	23281	4,89	2,91E-02
MUC1	7508	mucin 1, cell surface as	ENSG00000185499	4582	3,21	7,80E-03
MUC16	15582	mucin 16, cell surface a	ENSG00000181143	94025	3,96	2,73E-02
MUC20	23282	mucin 20, cell surface a	ENSG00000176945	200958	2,88	2,97E-03
MUC5B	7516	mucin 5B, oligomeric m	ENSG00000117983	727897	3,32	2,60E-02
MUC6	7517	mucin 6, oligomeric mu	ENSG00000184956	4588	5,09	3,57E-03
MUM1L1	26583	melanoma associated a	ENSG00000157502	139221	7,02	2,70E-06
MUSTN1	22144	musculoskeletal, embry	ENSG00000243696	389125	7,26	1,43E-07
MX2	7533	myxovirus (influenza vi	ENSG00000183486	4600	2,97	7,74E-05
MXI1	7534	MAX interactor 1, dimer	ENSG00000119950	4601	2,08	3,42E-04
MXRA5	7539	matrix-remodelling ass(	ENSG00000101825	25878	8,11	1,36E-04
MXRA8	7542	matrix-remodelling ass(	ENSG00000162576	54587	2,45	1,88E-04
MYH11	7569	myosin, heavy chain 11	ENSG00000133392	4629	9,31	4,35E-10
MYH3	7573	myosin, heavy chain 3,	ENSG00000109063	4621	3,92	3,65E-02

MYH7B	15906	myosin, heavy chain 7B	ENSG00000078814	57644	3,41	1,17E-03
MYL3	7584	myosin, light chain 3, a	ENSG00000160808	4634	4,71	5,31E-04
MYL4	7585	myosin, light chain 4, a	ENSG00000198336	4635	3,41	9,18E-03
MYL9	15754	myosin, light chain 9, re	ENSG00000101335	10398	3,79	1,62E-02
MYLK	7590	myosin light chain kinas	ENSG00000065534	4638	5,48	4,45E-07
MYO15B	14083	myosin XVB pseudogen	ENSG00000266714	80022	3,93	4,25E-08
MYO18B	18150	myosin XVIIIB [Source:	ENSG00000133454	84700	5,17	2,45E-03
MYO1G	13880	myosin IG [Source:HGN	ENSG00000136286	64005	4,14	1,49E-02
MYO7A	7606	myosin VIIA [Source:H	ENSG00000137474	4647	3,52	7,45E-03
MYO7B	7607	myosin VIIB [Source:H	ENSG00000169994	4648	4,97	2,29E-04
MYOC	7610	myocilin, trabecular me	ENSG00000034971	4653	10,81	1,73E-09
MYOCD	16067	myocardin [Source:HGN	ENSG00000141052	93649	7,56	5,44E-06
MYOM1	7613	myomesin 1 [Source:H	ENSG00000101605	8736	4,75	3,58E-03
MYOT	12399	myotilin [Source:HGNC	ENSG00000120729	9499	3,74	1,64E-04
MYOZ1	13752	myozenin 1 [Source:HG	ENSG00000177791	58529	6,99	9,11E-05
MYOZ2	1330	myozenin 2 [Source:HG	ENSG00000172399	51778	4,75	3,35E-05
N4BP2L1	25037	NEDD4 binding protein	ENSG00000139597	90634	2,38	1,22E-02
NAALAD2	14526	N-acetylated alpha-link	ENSG00000077616	10003	5,16	5,52E-06
NAALADL2	23219	N-acetylated alpha-link	ENSG00000177694	254827	2,29	1,72E-03
NACAP1	24688	nascent-polypeptide-as	ENSG00000228224	83955	3,44	2,92E-02
NAP1L2	7638	nucleosome assembly p	ENSG00000186462	4674	2,88	3,32E-02
NAP1L3	7639	nucleosome assembly p	ENSG00000186310	4675	3,98	1,16E-06
NAPSB	13396	napsin B aspartic peptic	ENSG00000131401	256236	3,92	9,33E-03
NBEA	7648	neurobeachin [Source:H	ENSG00000172915	26960	2,89	5,60E-05
NBL1	7650	neuroblastoma 1, DAN f	ENSG00000158747	4681	2,60	9,63E-03
NBLA00301					4,12	8,31E-03
NCALD	7655	neurocalcin delta [Sourc	ENSG00000104490	83988	5,24	3,02E-11
NCAM1	7656	neural cell adhesion mo	ENSG00000262279	4684	2,71	1,24E-02
NCF4	7662	neutrophil cytosolic fact	ENSG00000100365	4689	7,42	7,84E-10
NCKAP1L	4862	NCK-associated protein	ENSG00000123338	3071	6,11	5,33E-07
NDNF	26256	neuron-derived neurotr	ENSG00000173376	79625	3,88	4,34E-03
NDP	7678	Norrie disease (pseudog	ENSG00000124479	4693	4,71	4,27E-04
NDRG2	14460	NDRG family member 2	ENSG00000165795	57447	6,25	2,10E-09
NDUFA4	7687	NADH dehydrogenase (	ENSG00000189043	4697	2,11	5,17E-03
NECAB1	20983	N-terminal EF-hand calc	ENSG00000123119	64168	3,66	2,22E-06
NECAB2	23746	N-terminal EF-hand calc	ENSG00000103154	54550	3,30	3,87E-02
NELL2	7751	NEL-like 2 (chicken) [Sc	ENSG00000184613	4753	4,10	4,81E-03
NENF	30384	neudesin neurotrophic f	ENSG00000117691	29937	2,94	1,04E-02
NET1	14592	neuroepithelial cell tran	ENSG00000173848	10276	2,74	3,42E-05
NEURL1B	35422	neuralized homolog 1B	ENSG00000214357	54492	5,12	5,52E-09
NEXN	29557	nexilin (F actin binding	ENSG00000162614	91624	2,65	1,02E-04
NFAM1	29872	NFAT activating protein	ENSG00000235568	150372	3,74	7,93E-03
NFASC	29866	neurofascin [Source:HG	ENSG00000163531	23114	8,01	1,21E-08
NFIA	7784	nuclear factor I/A [Sour	ENSG00000162599	4774	2,51	1,30E-06
NFIX	7788	nuclear factor I/X (CCA	ENSG00000008441	4784	2,06	1,66E-04
NFKBIA	7797	nuclear factor of kappa	ENSG00000100906	4792	2,56	8,46E-03
NFKBID	15671	nuclear factor of kappa	ENSG00000167604	84807	3,56	4,18E-03
NFKBIZ	29805	nuclear factor of kappa	ENSG00000144802	64332	4,33	4,27E-05
NGEF	7807	neuronal guanine nucle	ENSG00000066248	25791	6,10	8,14E-04
NGFR	7809	nerve growth factor rec	ENSG00000064300	4804	6,10	4,91E-03
NHS	7820	Nance-Horan syndrome	ENSG00000188158	4810	3,64	3,78E-04
NIPSNAP3B	23641	nipsnap homolog 3B (C.	ENSG00000165028	55335	3,48	2,02E-04
NKD1	17045	naked cuticle homolog 1	ENSG00000140807	85407	7,54	4,78E-07

NKG7	7830	natural killer cell group	ENSG00000105374	4818	3,25	1,01E-02
NKPD1	24739	NTPase, KAP family P-lc	ENSG00000179846	284353	2,31	2,14E-02
NLGN3	14289	neuroligin 3 [Source:HG	ENSG00000196338	54413	2,14	4,51E-02
NLRP3	16400	NLR family, pyrin domai	ENSG00000162711	114548	7,00	4,84E-05
NLRP9	22941	NLR family, pyrin domai	ENSG00000185792	338321	2,21	4,56E-02
NMNAT2	16789	nicotinamide nucleotide	ENSG00000157064	23057	2,63	8,68E-03
NMNAT3	20989	nicotinamide nucleotide	ENSG00000163864	349565	4,88	3,86E-08
NMUR1	4518	neuromedin U receptor	ENSG00000171596	10316	6,13	5,72E-07
NNAT	7860	neuronatin [Source:HGI	ENSG00000053438	4826	3,39	5,37E-03
NOD2	5331	nucleotide-binding oligo	ENSG00000167207	64127	3,67	3,31E-02
NOSTRIN	20203	nitric oxide synthase tra	ENSG00000262773	115677	2,77	2,01E-02
NOV	7885	nephroblastoma overex	ENSG00000136999	4856	7,52	2,02E-04
NOVA1	7886	neuro-oncological ventr	ENSG00000139910	4857	5,17	4,43E-07
NPAS3	19311	neuronal PAS domain pı	ENSG00000151322	64067	3,02	1,03E-02
NPAS4	18983	neuronal PAS domain pı	ENSG00000174576	266743	5,35	3,37E-04
NPFF	7901	neuropeptide FF-amide	ENSG00000139574	8620	2,28	9,96E-03
NPNT	27405	nephronectin [Source:H	ENSG00000168743	255743	8,27	1,36E-04
NPPC	7941	natriuretic peptide C [S	ENSG00000163273	4880	5,34	4,59E-03
NPTX1	7952	neuronal pentraxin I [S	ENSG00000171246	4884	4,19	4,18E-02
NPY1R	7956	neuropeptide Y recepto	ENSG00000164128	4886	10,62	1,62E-17
NPY5R	7958	neuropeptide Y recepto	ENSG00000164129	4889	6,97	1,28E-09
NR4A1	7980	nuclear receptor subfan	ENSG00000123358	3164	7,90	1,49E-05
NR4A2	7981	nuclear receptor subfan	ENSG00000153234	4929	7,60	3,47E-06
NR4A3	7982	nuclear receptor subfan	ENSG00000119508	8013	7,98	3,90E-03
NR5A1	7983	nuclear receptor subfan	ENSG00000136931	2516	4,88	4,31E-02
NRXN1	8008	neurexin 1 [Source:HGI	ENSG00000179915	9378	6,27	4,50E-07
NRXN2	8009	neurexin 2 [Source:HGI	ENSG00000110076	9379	5,60	6,53E-06
NSUN6	23529	NOP2/Sun domain famil	ENSG00000241058	221078	2,03	1,50E-04
NSUN7	25857	NOP2/Sun domain famil	ENSG00000179299	79730	4,81	1,06E-07
NTF3	8023	neurotrophin 3 [Source	ENSG00000185652	4908	6,09	7,76E-06
NTN1	8029	netrin 1 [Source:HGNC	ENSG00000065320	9423	6,33	6,89E-07
NTNG1	23319	netrin G1 [Source:HGN	ENSG00000162631	22854	3,34	2,48E-02
NTNG2	14288	netrin G2 [Source:HGN	ENSG00000196358	84628	2,37	3,85E-02
NTRK2	8032	neurotrophic tyrosine ki	ENSG00000148053	4915	6,63	4,95E-05
NTRK3	8033	neurotrophic tyrosine ki	ENSG00000140538	4916	8,16	4,61E-09
NTS	8038	neurotensin [Source:HG	ENSG00000133636	4922	10,07	1,84E-06
NUDT10	17621	nudix (nucleoside dipho	ENSG00000122824	170685	3,27	6,34E-03
NUP210L	29915	nucleoporin 210kDa-like	ENSG00000143552	91181	4,18	4,26E-02
NXPH3	8077	neurexophilin 3 [Source	ENSG00000182575	11248	3,05	9,34E-03
OCA2	8101	oculocutaneous albinism	ENSG00000104044	4948	3,19	2,82E-02
ODF3B	34388	outer dense fiber of spe	ENSG00000177989	440836	2,14	2,78E-02
ODF3L1	28735	outer dense fiber of spe	ENSG00000182950	161753	4,74	1,31E-04
ODZ1					7,11	2,36E-10
OGN	8126	osteoglycin [Source:HG	ENSG00000106809	4969	9,61	1,07E-14
OLFM1	17187	olfactomedin 1 [Source:	ENSG00000130558	10439	3,26	8,18E-05
OLFML1	24473	olfactomedin-like 1 [Sou	ENSG00000183801	283298	8,46	3,29E-19
OLFML2A	27270	olfactomedin-like 2A [S	ENSG00000185585	169611	2,95	1,53E-02
OLFML2B	24558	olfactomedin-like 2B [S	ENSG00000162745	25903	5,99	2,85E-13
OLFML3	24956	olfactomedin-like 3 [Sou	ENSG00000116774	56944	2,83	1,72E-05
OMD	8134	osteomodulin [Source:H	ENSG00000127083	4958	8,19	1,72E-06
OPCML	8143	opioid binding protein/c	ENSG00000183715	4978	3,93	1,48E-02
OSM	8506	oncostatin M [Source:H	ENSG00000099985	5008	4,89	4,40E-03
OSR1	8111	odd-skipped related 1 (	ENSG00000143867	130497	5,15	2,23E-05

OSR2	15830	odd-skipped related 2 (	ENSG00000164920	116039	5,82	6,40E-11
OSTBETA					4,77	4,49E-05
OTC	8512	ornithine carbamoyltran	ENSG00000036473	5009	8,05	1,21E-07
OXER1	24884	oxoeicosanoid (OXE) re	ENSG00000162881	165140	3,16	2,93E-02
OXGR1	4531	oxoglutarate (alpha-ket	ENSG00000165621	27199	7,96	1,52E-06
P2RX1	8533	purinergic receptor P2X	ENSG00000108405	5023	7,98	5,90E-05
P2RY12	18124	purinergic receptor P2Y,	ENSG00000169313	64805	5,83	4,22E-05
P2RY13	4537	purinergic receptor P2Y,	ENSG00000181631	53829	5,94	4,27E-05
P2RY14	16442	purinergic receptor P2Y,	ENSG00000174944	9934	7,25	3,20E-06
PADI2	18341	peptidyl arginine deimir	ENSG00000117115	11240	4,47	3,57E-03
PAIP2B	29200	poly(A) binding protein	ENSG00000124374	400961	2,20	3,88E-03
PAK3	8592	p21 protein (Cdc42/Rac	ENSG00000077264	5063	2,87	3,16E-03
PALLD	17068	palladin, cytoskeletal as	ENSG00000129116	23022	3,10	3,48E-05
PAR-SN					3,18	6,79E-03
PARD6B	16245	par-6 partitioning defec	ENSG00000124171	84612	3,10	3,41E-02
PARD6G	16076	par-6 partitioning defec	ENSG00000178184	84552	2,98	8,40E-07
PARK2	8607	parkinson protein 2, E3	ENSG00000185345	5071	3,33	4,87E-04
PARM1	24536	prostate androgen-regu	ENSG00000169116	25849	9,12	5,03E-15
PARP8	26124	poly (ADP-ribose) polyn	ENSG00000151883	79668	3,50	6,72E-05
PART1	17263	prostate androgen-regu	ENSG00000152931	25859	5,41	1,47E-03
PARVG	14654	parvin, gamma [Source	ENSG00000138964	64098	2,80	2,91E-02
PATL2	33630	protein associated with	ENSG00000229474	197135	2,33	3,82E-02
PAX6	8620	paired box 6 [Source:H	ENSG00000007372	5080	2,31	8,44E-04
PBX1	8632	pre-B-cell leukemia hon	ENSG00000185630	5087	2,66	2,62E-03
PCCA	8653	propionyl CoA carboxyla	ENSG00000175198	5095	2,14	9,36E-04
PCDH15	14674	protocadherin-related 1	ENSG00000150275	65217	5,10	2,54E-02
PCDH18	14268	protocadherin 18 [Sour	ENSG00000189184	54510	3,00	1,38E-02
PCDH19	14270	protocadherin 19 [Sour	ENSG00000165194	57526	2,89	3,03E-02
PCDH20	14257	protocadherin 20 [Sour	ENSG00000197991	64881	7,14	3,33E-06
PCDH7	8659	protocadherin 7 [Source	ENSG00000169851	5099	3,20	3,42E-02
PCDHB16	14546	protocadherin beta 16 [	ENSG00000196963	57717	2,68	1,05E-03
PCDHB4	8689	protocadherin beta 4 [S	ENSG00000081818	56131	5,50	9,17E-08
PCK1	8724	phosphoenolpyruvate ca	ENSG00000124253	5105	5,72	1,61E-02
PCOLCE	8738	procollagen C-endopept	ENSG00000106333	5118	4,53	4,00E-06
PCP4	8742	Purkinje cell protein 4 [	ENSG00000183036	5121	7,74	2,23E-05
PCP4L1	20448	Purkinje cell protein 4 li	ENSG00000248485	654790	5,67	5,45E-04
PCSK2	8744	proprotein convertase s	ENSG00000125851	5126	5,90	2,16E-04
PCSK5	8747	proprotein convertase s	ENSG00000099139	5125	3,11	7,37E-04
PDE1A	8774	phosphodiesterase 1A, (	ENSG00000115252	5136	6,01	6,39E-06
PDE1B	8775	phosphodiesterase 1B, (	ENSG00000123360	5153	7,10	6,78E-10
PDE3B	8779	phosphodiesterase 3B, (	ENSG00000261923	5140	4,30	3,78E-02
PDE4C	8782	phosphodiesterase 4C, (	ENSG00000105650	5143	4,00	3,05E-02
PDE5A	8784	phosphodiesterase 5A, (	ENSG00000138735	8654	2,42	2,62E-03
PDE8B	8794	phosphodiesterase 8B [	ENSG00000113231	8622	2,10	1,91E-02
PDGFD	30620	platelet derived growth	ENSG00000170962	80310	3,23	1,21E-07
PDGFRA	8803	platelet-derived growth	ENSG00000134853	5156	3,75	2,43E-05
PDGFRB	8804	platelet-derived growth	ENSG00000113721	5159	2,99	1,65E-04
PDGFRL	8805	platelet-derived growth	ENSG00000104213	5157	3,98	2,34E-03
PDK3	8811	pyruvate dehydrogenas	ENSG00000067992	5165	3,65	5,21E-08
PDK4	8812	pyruvate dehydrogenas	ENSG00000004799	5166	4,34	2,22E-02
PDLIM3	20767	PDZ and LIM domain 3	ENSG00000154553	27295	4,15	5,04E-06
PDZD2	18486	PDZ domain containing	ENSG00000133401	23037	3,54	5,72E-03
PDZD4	21167	PDZ domain containing	ENSG00000067840	57595	4,15	1,07E-05

PDZRN3	17704	PDZ domain containing	ENSG00000121440	23024	5,95	7,41E-11
PDZRN4	30552	PDZ domain containing	ENSG00000165966	29951	7,85	4,38E-08
PEBP4	28319	phosphatidylethanolami	ENSG00000134020	157310	5,01	6,81E-03
PEG3	8826	paternally expressed 3	ENSG00000198300	5178	4,02	6,87E-07
PENK	8831	proenkephalin [Source:	ENSG00000181195	5179	6,13	1,52E-02
PER1	8845	period circadian clock 1	ENSG00000179094	5187	3,33	9,94E-04
PER2	8846	period circadian clock 2	ENSG00000132326	8864	4,27	8,06E-06
PER3	8847	period circadian clock 3	ENSG00000049246	8863	2,83	4,39E-06
PEX5L	30024	peroxisomal biogenesis	ENSG00000114757	51555	3,06	4,07E-03
PFKFB2	8873	6-phosphofructo-2-kina	ENSG00000123836	5208	2,12	3,28E-03
PGM5	8908	phosphoglucomutase 5	ENSG00000266607	5239	2,27	5,64E-04
PGR	8910	progesterone receptor [	ENSG00000082175	5241	7,26	3,22E-14
PHKG1	8930	phosphorylase kinase, g	ENSG00000164776	5260	2,63	1,27E-02
PHOSPHO1	16815	phosphatase, orphan 1	ENSG00000173868	162466	3,04	4,65E-02
PHYHD1	23396	phytanoyl-CoA dioxyger	ENSG00000175287	254295	5,33	1,35E-10
PHYHIP	16865	phytanoyl-CoA 2-hydro:	ENSG00000168490	9796	5,94	3,80E-10
PI15	8946	peptidase inhibitor 15 [	ENSG00000137558	51050	9,14	4,52E-05
PI16	21245	peptidase inhibitor 16 [	ENSG00000164530	221476	8,87	4,60E-08
PIBF1	23352	progesterone immunom	ENSG00000083535	10464	2,10	1,98E-06
PID1	26084	phosphotyrosine interac	ENSG00000153823	55022	3,76	3,76E-04
PIK3AP1	30034	phosphoinositide-3-kina	ENSG00000155629	118788	5,20	4,74E-04
PIK3R5	30035	phosphoinositide-3-kina	ENSG00000141506	23533	6,18	1,75E-05
PILRA	20396	paired immunoglobin-lik	ENSG00000085514	29992	3,06	3,74E-03
PIM1	8986	pim-1 oncogene [Sourc	ENSG00000137193	5292	4,02	1,09E-02
PION					2,22	3,10E-03
PIP5K1B	8995	phosphatidylinositol-4-p	ENSG00000107242	8395	6,32	1,47E-06
PIWIL2	17644	piwi-like RNA-mediated	ENSG00000197181	55124	2,53	9,04E-03
PIWIL4	18444	piwi-like RNA-mediated	ENSG00000134627	143689	2,52	1,06E-03
PKD1L2	21715	polycystic kidney diseas	ENSG00000269323	114780	5,72	8,51E-05
PKDCC	25123	protein kinase domain c	ENSG00000162878	91461	4,38	1,94E-05
PKHD1L1	20313	polycystic kidney and h	ENSG00000205038	93035	4,72	3,08E-05
PKNOX2	16714	PBX/knotted 1 homeobo	ENSG00000165495	63876	4,46	4,27E-04
PKP1	9023	plakophilin 1 (ectoderm	ENSG00000081277	5317	5,76	9,37E-12
PLA2G2A	9031	phospholipase A2, grou	ENSG00000188257	5320	9,25	3,42E-05
PLA2G4A	9035	phospholipase A2, grou	ENSG00000116711	5321	2,53	4,61E-03
PLA2G5	9038	phospholipase A2, grou	ENSG00000127472	5322	6,70	2,74E-06
PLA2G6	9039	phospholipase A2, grou	ENSG00000184381	8398	2,49	7,14E-06
PLAC9	19255	placenta-specific 9 [Sou	ENSG00000189129	219348	4,91	1,15E-04
PLB1	30041	phospholipase B1 [Sour	ENSG00000163803	151056	2,53	4,49E-03
PLBD1	26215	phospholipase B domair	ENSG00000121316	79887	6,76	7,34E-05
PLCB4	9059	phospholipase C, beta 4	ENSG00000101333	5332	3,43	4,61E-10
PLCL1	9063	phospholipase C-like 1 [	ENSG00000115896	5334	2,06	1,61E-02
PLCXD3	31822	phosphatidylinositol-spe	ENSG00000182836	345557	8,13	1,84E-15
PLD5	26879	phospholipase D family,	ENSG00000180287	200150	6,22	8,05E-06
PLEK	9070	pleckstrin [Source:HGN	ENSG00000115956	5341	6,81	8,96E-06
PLEKHA4	14339	pleckstrin homology do	ENSG00000105559	57664	2,41	3,39E-04
PLEKHA5	30036	pleckstrin homology do	ENSG00000052126	54477	2,02	5,20E-04
PLEKHG3	20364	pleckstrin homology do	ENSG00000126822	26030	4,13	3,83E-05
PLEKHG6	25562	pleckstrin homology do	ENSG00000008323	55200	3,94	2,33E-03
PLEKHH2	30506	pleckstrin homology do	ENSG00000152527	130271	4,85	3,95E-12
PLIN1	9076	perilipin 1 [Source:HGN	ENSG00000166819	5346	8,39	3,66E-03
PLIN4	29393	perilipin 4 [Source:HGN	ENSG00000167676	729359	5,20	7,90E-03
PLK1S1	15865	polo-like kinase 1 subst	ENSG00000088970		3,53	3,44E-08

PLK5	27001	polo-like kinase 5 [Sour	ENSG00000185988	126520	4,25	3,12E-03
PLN	9080	phospholamban [Source	ENSG00000198523	5350	10,20	1,52E-19
PLP1	9086	proteolipid protein 1 [S	ENSG00000123560	5354	8,60	2,39E-12
PLTP	9093	phospholipid transfer pr	ENSG00000100979	5360	3,02	5,18E-03
PLXDC1	20945	plexin domain containin	ENSG00000161381	57125	8,56	1,74E-18
PLXDC2	21013	plexin domain containin	ENSG00000120594	84898	4,36	1,23E-05
PLXNC1	9106	plexin C1 [Source:HGN	ENSG00000136040	10154	5,25	4,25E-08
PM20D1	26518	peptidase M20 domain	ENSG00000162877	148811	5,38	1,38E-05
PMP2	9117	peripheral myelin protei	ENSG00000147588	5375	4,95	9,54E-04
PNCK	13415	pregnancy up-regulatec	ENSG00000130822	139728	6,61	5,46E-08
PNMAL2	29206	paraneoplastic Ma antig	ENSG00000204851	57469	3,63	5,02E-05
PNMT	9160	phenylethanolamine N-r	ENSG00000141744	5409	5,05	2,79E-03
PNPLA7	24768	patatin-like phospholipa	ENSG00000130653	375775	2,86	2,20E-07
PNRC1	17278	proline-rich nuclear rec	ENSG00000146278	10957	2,75	6,64E-03
POC5	26658	POC5 centriolar protein	ENSG00000152359	134359	2,53	2,83E-04
PODN	23174	podocan [Source:HGNC	ENSG00000174348	127435	6,45	4,89E-11
POF1B	13711	premature ovarian failu	ENSG00000124429	79983	4,01	7,31E-07
POM121L9P	30080	POM121 transmembran	ENSG00000128262	29774	2,60	3,65E-02
POPDC2	17648	popeye domain containi	ENSG00000121577	64091	4,94	1,02E-04
PP2D1	28406	protein phosphatase 2C	ENSG00000183977	151649	3,09	8,08E-03
PPAP2A	9228	phosphatidic acid phosp	ENSG00000067113	8611	2,49	2,00E-03
PPAP2B	9229	phosphatidic acid phosp	ENSG00000162407	8613	2,54	1,43E-04
PPARGC1A	9237	peroxisome proliferator-	ENSG00000109819	10891	4,29	3,12E-05
PPARGC1B	30022	peroxisome proliferator-	ENSG00000155846	133522	2,37	1,25E-02
PPFIA2	9246	protein tyrosine phosph	ENSG00000139220	8499	6,97	1,91E-10
PPFIA4	9248	protein tyrosine phosph	ENSG00000143847	8497	2,35	2,33E-02
PPFIBP2	9250	PTPRF interacting protei	ENSG00000166387	8495	3,29	5,51E-05
PPIEL	33195	peptidylprolyl isomerase	ENSG00000243970		2,09	3,58E-02
PPL	9273	periplakin [Source:HGN	ENSG00000118898	5493	4,14	2,05E-05
PPP1R12B	7619	protein phosphatase 1,	ENSG00000077157	4660	5,68	8,73E-07
PPP1R14A	14871	protein phosphatase 1,	ENSG00000167641	94274	5,42	6,44E-04
PPP1R15A	14375	protein phosphatase 1,	ENSG00000087074	23645	2,34	1,38E-02
PPP1R1A	9286	protein phosphatase 1,	ENSG00000135447	5502	8,73	6,34E-05
PPP1R1B	9287	protein phosphatase 1,	ENSG00000131771	84152	6,16	7,08E-03
PPP1R36	20097	protein phosphatase 1,	ENSG00000165807	145376	4,67	1,87E-03
PPP1R3G	14945	protein phosphatase 1,	ENSG00000219607	648791	2,27	1,18E-02
PPP1R9A	14946	protein phosphatase 1,	ENSG00000158528	55607	2,33	5,74E-03
PPP2R2B	9305	protein phosphatase 2,	ENSG00000156475	5521	4,79	2,48E-04
PRDM16	14000	PR domain containing 1	ENSG00000142611	63976	2,80	8,05E-04
PRDM6	9350	PR domain containing 6	ENSG00000061455	93166	5,27	3,00E-05
PRELP	9357	proline/arginine-rich en	ENSG00000188783	5549	8,85	2,33E-15
PRF1	9360	perforin 1 (pore forming	ENSG00000180644	5551	4,81	1,66E-03
PRG2	9362	proteoglycan 2, bone m	ENSG00000186652	5553	3,31	4,72E-03
PRG4	9364	proteoglycan 4 [Source	ENSG00000116690	10216	4,99	6,89E-03
PRICKLE2	20340	prickle homolog 2 (Dros	ENSG00000163637	166336	3,48	9,79E-04
PRICKLE4	16805	prickle homolog 4 (Dros	ENSG00000124593	29964	2,06	9,22E-04
PRIMA1	18319	proline rich membrane a	ENSG00000175785	145270	6,56	1,70E-09
PRKAA2	9377	protein kinase, AMP-act	ENSG00000162409	5563	3,54	1,22E-05
PRKCB	9395	protein kinase C, beta [	ENSG00000166501	5579	7,95	8,76E-09
PRKCQ	9410	protein kinase C, theta	ENSG00000065675	5588	2,33	2,58E-02
PRKCZ	9412	protein kinase C, zeta [	ENSG00000067606	5590	2,04	2,69E-03
PRKG1	9414	protein kinase, cGMP-de	ENSG00000185532	5592	4,34	5,93E-07
PRKG2	9416	protein kinase, cGMP-de	ENSG00000138669	5593	3,58	4,84E-02

PRODH	9453	proline dehydrogenase (	ENSG00000100033	5625	4,61	4,65E-04
PROK2	18455	prokineticin 2 [Source:H	ENSG00000163421	60675	4,02	4,05E-02
PRPH	9461	peripherin [Source:HGN	ENSG00000135406	5630	5,20	3,38E-03
PRR24	27406	proline rich 24 [Source:	ENSG00000257704	255783	2,49	1,03E-03
PRRG4	30799	proline rich Gla (G-carb	ENSG00000135378	79056	2,04	4,54E-02
PRRT1	13943	proline-rich transmemb	ENSG00000235956	80863	2,35	5,36E-03
PRRT2	30500	proline-rich transmemb	ENSG00000167371	112476	3,87	8,37E-06
PRRT4	37280	proline-rich transmemb	ENSG00000224940	401399	4,58	2,62E-03
PRRX1	9142	paired related homeobo	ENSG00000116132	5396	6,29	8,77E-19
PRRX2	21338	paired related homeobo	ENSG00000167157	51450	2,41	4,54E-03
PRSS35	21387	protease, serine, 35 [Sc	ENSG00000146250	167681	5,91	4,13E-04
PRUNE2	25209	prune homolog 2 (Dros	ENSG00000106772	158471	2,95	1,56E-03
PSD	9507	pleckstrin and Sec7 dom	ENSG00000059915	5662	2,86	2,91E-02
PSTPIP1	9580	proline-serine-threonine	ENSG00000140368	9051	4,08	1,54E-03
PTAFR	9582	platelet-activating facto	ENSG00000169403	5724	4,86	4,75E-06
PTBP2	17662	polypyrimidine tract bin	ENSG00000117569	58155	2,23	5,73E-05
PTCH2	9586	patched 2 [Source:HGN	ENSG00000117425	8643	3,52	1,48E-03
PTCHD1	26392	patched domain contair	ENSG00000165186	139411	5,85	3,09E-04
PTCHD3	24776	patched domain contair	ENSG00000182077	374308	5,59	1,31E-02
PTGDR	9591	prostaglandin D2 recept	ENSG00000168229	5729	5,46	2,19E-06
PTGDS	9592	prostaglandin D2 synth	ENSG00000107317	5730	6,13	3,11E-04
PTGER3	9595	prostaglandin E recepto	ENSG00000050628	5733	5,59	4,94E-03
PTGFR	9600	prostaglandin F recepto	ENSG00000122420	5737	4,59	5,90E-04
PTGIS	9603	prostaglandin I2 (prost	ENSG00000124212	5740	4,39	3,83E-09
PTGS2	9605	prostaglandin-endopero	ENSG00000073756	5743	5,36	1,87E-04
PTH1R	9608	parathyroid hormone 1	ENSG00000160801	5745	6,89	8,78E-11
PTH2R	9609	parathyroid hormone 2	ENSG00000144407	5746	4,39	2,78E-03
PTN	9630	pleiotrophin [Source:HG	ENSG00000105894	5764	6,34	2,17E-08
PTPN13	9646	protein tyrosine phosph	ENSG00000163629	5783	3,03	1,21E-04
PTPN3	9655	protein tyrosine phosph	ENSG00000070159	5774	3,87	4,40E-07
PTPRC	9666	protein tyrosine phosph	ENSG00000262418	5788	3,49	5,00E-03
PTPRO	9678	protein tyrosine phosph	ENSG00000151490	5800	4,17	3,32E-03
PTPRZ1	9685	protein tyrosine phosph	ENSG00000106278	5803	5,12	1,83E-04
PYGM	9726	phosphorylase, glycoge	ENSG00000068976	5837	7,20	4,11E-14
PYHIN1	28894	pyrin and HIN domain f	ENSG00000163564	149628	4,28	5,53E-03
RAB20	18260	RAB20, member RAS or	ENSG00000139832	55647	2,61	3,72E-02
RAB27B	9767	RAB27B, member RAS (	ENSG00000041353	5874	4,12	1,89E-02
RAB3IL1	9780	RAB3A interacting prote	ENSG00000167994	5866	2,28	6,22E-05
RAB40A	18283	RAB40A, member RAS (	ENSG00000172476	142684	3,46	2,58E-02
RAB9B	14090	RAB9B, member RAS or	ENSG00000123570	51209	4,37	5,94E-06
RADIL	22226	Ras association and DIL	ENSG00000157927	55698	3,92	3,73E-03
RAI2	9835	retinoic acid induced 2 |	ENSG00000131831	10742	2,52	3,87E-06
RAMP1	9843	receptor (G protein-cou	ENSG00000132329	10267	7,49	6,07E-06
RAMP3	9845	receptor (G protein-cou	ENSG00000122679	10268	4,29	2,24E-02
RANBP3L	26353	RAN binding protein 3-li	ENSG00000164188	202151	4,98	1,84E-05
RAP1A	9855	RAP1A, member of RAS	ENSG00000116473	5906	2,10	3,74E-03
RAPGEF4	16626	Rap guanine nucleotide	ENSG00000091428	11069	2,56	3,74E-05
RARRES2	9868	retinoic acid receptor re	ENSG00000106538	5919	9,71	1,36E-19
RARRES3	9869	retinoic acid receptor re	ENSG00000133321	5920	2,99	4,35E-04
RASGEF1B	24881	RasGEF domain family,	ENSG00000138670	153020	7,90	1,05E-13
RASGRP2	9879	RAS guanyl releasing pr	ENSG00000068831	10235	5,21	1,12E-09
RASGRP4	18958	RAS guanyl releasing pr	ENSG00000171777	115727	4,20	1,20E-04
RASL11A	23802	RAS-like, family 11, me	ENSG00000122035	387496	5,02	5,97E-05

RASL11B	23804	RAS-like, family 11, me	ENSG00000128045	65997	6,27	3,07E-07
RASL12	30289	RAS-like, family 12 [Sou	ENSG00000103710	51285	9,41	1,84E-11
RASSF10	33984	Ras association (RalGDS	ENSG00000189431		6,83	3,10E-04
RASSF5	17609	Ras association (RalGDS	ENSG00000266094	83593	4,63	4,17E-05
RASSF9	15739	Ras association (RalGDS	ENSG00000198774	9182	4,28	1,54E-02
RBFOX3	27097	RNA binding protein, fo:	ENSG00000267483	146713	4,97	2,20E-02
RBM11	9897	RNA binding motif prote	ENSG00000185272	54033	4,80	3,60E-05
RBM24	21539	RNA binding motif prote	ENSG00000112183	221662	4,29	9,60E-03
RBM47	30358	RNA binding motif prote	ENSG00000163694	54502	6,50	7,33E-06
RBP4	9922	retinol binding protein 4	ENSG00000138207	5950	4,76	2,32E-02
RBP7	30316	retinol binding protein 7	ENSG00000162444	116362	6,31	2,16E-12
RBPMS2	19098	RNA binding protein wit	ENSG00000166831	348093	2,60	2,45E-02
RCAN2	3041	regulator of calcineurin	ENSG00000172348	10231	6,58	4,37E-09
RCSD1	28310	RCSD domain containin	ENSG00000198771	92241	2,45	4,44E-02
RDH5	9940	retinol dehydrogenase 5	ENSG00000135437	5959	5,47	1,51E-06
REEP1	25786	receptor accessory prot	ENSG00000068615	65055	3,45	1,13E-07
REM1	15922	RAS (RAD and GEM)-lik	ENSG00000088320	28954	4,82	5,72E-03
REM2	20248	RAS (RAD and GEM)-lik	ENSG00000139890	161253	3,60	2,52E-02
RERG	15980	RAS-like, estrogen-regu	ENSG00000134533	85004	10,07	1,43E-23
RERGL	26213	RERG/RAS-like [Source	ENSG00000111404	79785	7,57	8,49E-03
RFX8	37253	RFX family member 8, l	ENSG00000196460	731220	2,38	4,43E-02
RGAG4	29430	retrotransposon gag do	ENSG00000242732	340526	2,38	3,38E-03
RGMA	30308	RGM domain family, me	ENSG00000182175	56963	8,66	1,86E-09
RGN	9989	regucalcin [Source:HGN	ENSG00000130988	9104	5,10	1,22E-03
RGS1	9991	regulator of G-protein s	ENSG00000090104	5996	8,78	2,96E-06
RGS13	9995	regulator of G-protein s	ENSG00000127074	6003	5,66	4,82E-03
RGS16	9997	regulator of G-protein s	ENSG00000143333	6004	9,08	4,31E-11
RGS18	14261	regulator of G-protein s	ENSG00000150681	64407	5,41	8,75E-04
RGS2	9998	regulator of G-protein s	ENSG00000116741	5997	6,69	1,99E-05
RGS22	24499	regulator of G-protein s	ENSG00000132554	26166	5,53	3,22E-06
RGS5	10001	regulator of G-protein s	ENSG00000143248	8490	5,87	1,31E-02
RGS6	10002	regulator of G-protein s	ENSG00000182732	9628	5,35	3,75E-03
RGS7BP	23271	regulator of G-protein s	ENSG00000186479	401190	7,63	1,70E-04
RHBDL3	16502	rhomboid, veinlet-like 3	ENSG00000141314	162494	3,44	5,52E-03
RHOH	686	ras homolog family mer	ENSG00000168421	399	3,30	3,84E-02
RHOU	17794	ras homolog family mer	ENSG00000116574	58480	2,95	1,27E-03
RIBC1	26537	RIB43A domain with co	ENSG00000158423	158787	3,17	2,52E-04
RIC3	30338	resistance to inhibitors	ENSG00000166405	79608	5,03	5,35E-05
RIMBP2	30339	RIMS binding protein 2	ENSG00000060709	23504	5,18	3,04E-03
RIMS3	21292	regulating synaptic mer	ENSG00000117016	9783	3,95	2,70E-04
RIMS4	16183	regulating synaptic mer	ENSG00000101098	140730	6,30	2,32E-03
RIPK3	10021	receptor-interacting ser	ENSG00000129465	11035	2,47	4,08E-03
RIPPLY2	21390	ripply2 homolog (zebrat	ENSG00000203877	134701	4,98	5,60E-05
RMRP	10031	RNA component of mito	ENSG00000269900	6023	5,13	9,96E-03
RNASE4	10047	ribonuclease, RNase A f	ENSG00000258818	6038	3,85	4,39E-11
RNASE6	10048	ribonuclease, RNase A f	ENSG00000169413	6039	5,86	7,33E-06
RND2	18315	Rho family GTPase 2 [S	ENSG00000108830	8153	3,03	3,15E-03
RNF112	12968	ring finger protein 112 |	ENSG00000128482	7732	4,61	1,28E-07
RNF125	21150	ring finger protein 125,	ENSG00000101695	54941	2,02	5,87E-03
RNF13	10057	ring finger protein 13 [S	ENSG00000082996	11342	2,10	2,11E-06
RNF138P1	30342	ring finger protein 138,	ENSG00000250853	379013	2,95	1,75E-02
RNF165	31696	ring finger protein 165 |	ENSG00000141622	494470	5,70	6,34E-05
RNF175	27735	ring finger protein 175 |	ENSG00000145428	285533	3,52	3,99E-02

RNF180	27752	ring finger protein 180 |	ENSG00000164197	285671	3,15	1,08E-05
RNLS	25641	renalase, FAD-depender	ENSG00000184719	55328	2,25	5,80E-03
ROR2	10257	receptor tyrosine kinase	ENSG00000169071	4920	7,81	2,72E-10
RPE65	10294	retinal pigment epitheliu	ENSG00000116745	6121	5,37	1,23E-04
RPPH1	19273	ribonuclease P RNA com	ENSG00000259001	85495	5,23	1,69E-02
RPRM	24201	reprimo, TP53 depender	ENSG00000177519	56475	5,65	9,02E-03
RPS27	10416	ribosomal protein S27 [	ENSG00000177954	6232	3,62	3,66E-05
RRAD	10446	Ras-related associated \	ENSG00000166592	6236	3,31	2,92E-04
RRAGD	19903	Ras-related GTP binding	ENSG00000025039	58528	4,08	1,21E-04
RSAD2	30908	radical S-adenosyl meth	ENSG00000134321	91543	3,94	8,12E-06
RSPH4A	21558	radial spoke head 4 hon	ENSG00000111834	345895	2,69	4,94E-02
RSPO3	20866	R-spondin 3 [Source:HC	ENSG00000146374	84870	3,37	1,66E-03
RSRC2	30559	arginine/serine-rich coil	ENSG00000111011	65117	2,11	1,47E-05
RTN1	10467	reticulon 1 [Source:HGI	ENSG00000139970	6252	6,45	1,56E-09
RTN4RL1	21329	reticulon 4 receptor-like	ENSG00000185924	146760	4,88	2,22E-03
RXRG	10479	retinoid X receptor, gam	ENSG00000143171	6258	6,07	3,86E-07
RYR1	10483	ryanodine receptor 1 (s	ENSG00000196218	6261	5,62	2,23E-05
RYR2	10484	ryanodine receptor 2 (c	ENSG00000198626	6262	6,85	4,52E-07
RYR3	10485	ryanodine receptor 3 [S	ENSG00000198838	6263	4,73	2,49E-06
S100A12	10489	S100 calcium binding pı	ENSG00000163221	6283	5,51	1,90E-02
S100A4	10494	S100 calcium binding pı	ENSG00000196154	6275	5,06	9,79E-05
S100A8	10498	S100 calcium binding pı	ENSG00000143546	6279	6,97	8,61E-03
S100A9	10499	S100 calcium binding pı	ENSG00000163220	6280	7,68	4,96E-03
S100B	10500	S100 calcium binding pı	ENSG00000160307	6285	7,85	1,29E-05
SAA1	10513	serum amyloid A1 [Sou	ENSG00000173432	6288	5,56	1,54E-02
SAP25	41908	Sin3A-associated protei	ENSG00000205307	100316904	2,73	1,45E-02
SASH3	15975	SAM and SH3 domain cı	ENSG00000122122	54440	4,66	2,09E-04
SCAPER	13081	S-phase cyclin A-associ	ENSG00000140386	49855	2,18	5,08E-07
SCARA5	28701	scavenger receptor clas	ENSG00000168079	286133	10,14	3,24E-11
SCARNA10	32567	small Cajal body-specifi	ENSG00000239002	692148	8,60	4,90E-07
SCARNA17	32574	small Cajal body-specifi	ENSG00000251992	677769	5,00	8,18E-04
SCARNA2	32558	small Cajal body-specifi	ENSG00000270066	677766	6,05	5,13E-04
SCARNA5	32561	small Cajal body-specifi	ENSG00000252010	677775	6,35	3,50E-04
SCARNA6	32562	small Cajal body-specifi	ENSG00000251791	677772	4,39	5,14E-03
SCARNA7	32563	small Cajal body-specifi	ENSG00000238741	677767	6,08	3,51E-04
SCARNA9	32566	small Cajal body-specifi	ENSG00000254911	619383	2,35	3,54E-02
SCG3	13707	secretogranin III [Sourc	ENSG00000104112	29106	5,32	3,50E-05
SCN11A	10583	sodium channel, voltage	ENSG00000168356	11280	3,82	1,18E-02
SCN2B	10589	sodium channel, voltage	ENSG00000149575	6327	5,23	1,10E-05
SCN3B	20665	sodium channel, voltage	ENSG00000166257	55800	3,89	5,95E-06
SCN4A	10591	sodium channel, voltage	ENSG00000007314	6329	6,42	2,63E-04
SCN4B	10592	sodium channel, voltage	ENSG00000177098	6330	6,79	4,50E-05
SCN7A	10594	sodium channel, voltage	ENSG00000136546	6332	7,55	1,96E-08
SCNN1A	10599	sodium channel, non-vo	ENSG00000111319	6337	3,30	3,69E-03
SCRG1	17036	stimulator of chondroge	ENSG00000164106	11341	4,80	3,90E-05
SCUBE2	30425	signal peptide, CUB dor	ENSG00000175356	57758	5,27	5,81E-12
SEC14L5	29032	SEC14-like 5 (S. cerevis	ENSG00000103184	9717	2,78	1,01E-02
SECTM1	10707	secreted and transmem	ENSG00000141574	6398	3,68	4,74E-04
SEL1L2	15897	sel-1 suppressor of lin-1	ENSG00000101251	80343	4,33	3,22E-03
SELENBP1	10719	selenium binding protei	ENSG00000143416	8991	4,03	9,17E-06
SELM					2,78	3,75E-02
SEMA3B	10724	sema domain, immunog	ENSG00000012171	7869	4,50	7,43E-08
SEMA3D	10726	sema domain, immunog	ENSG00000153993	223117	3,42	8,82E-04

SEMA3E	10727	sema domain, immunog	ENSG00000170381	9723	4,13	6,16E-05
SEMA4A	10729	sema domain, immunog	ENSG00000196189	64218	4,29	3,16E-02
SEMA4D	10732	sema domain, immunog	ENSG00000187764	10507	2,97	1,97E-06
SEMA5B	10737	sema domain, seven thı	ENSG00000082684	54437	4,97	2,60E-04
SEMA6A	10738	sema domain, transmer	ENSG00000092421	57556	2,66	5,57E-03
SEPP1	10751	selenoprotein P, plasma	ENSG00000250722	6414	5,08	3,96E-07
SEPT1	2879	septin 1 [Source:HGNC	ENSG00000180096	1731	2,67	1,02E-02
SEPT4	9165	septin 4 [Source:HGNC	ENSG00000108387	5414	2,55	6,97E-04
SEPT7	1717	septin 7 [Source:HGNC	ENSG00000122545	989	2,52	2,00E-07
SEPT7P2	32339	septin 7 pseudogene 2	ENSG00000214765	641977	2,48	1,87E-03
SERINC1	13464	serine incorporator 1 [S	ENSG00000111897	57515	2,13	2,55E-05
SERPINA1	8941	serpin peptidase inhibit	ENSG00000197249	5265	5,73	5,43E-03
SERPINA5	8723	serpin peptidase inhibit	ENSG00000188488	5104	4,41	8,26E-04
SERPINB1	3311	serpin peptidase inhibit	ENSG00000021355	1992	4,10	4,75E-05
SERPINF1	8824	serpin peptidase inhibit	ENSG00000132386	5176	3,41	2,91E-05
SERPINF2	9075	serpin peptidase inhibit	ENSG00000167711	5345	4,70	1,38E-02
SERPING1	1228	serpin peptidase inhibit	ENSG00000149131	710	9,59	4,04E-19
SERPINI1	8943	serpin peptidase inhibit	ENSG00000163536	5274	5,84	2,38E-06
SETBP1	15573	SET binding protein 1 [S	ENSG00000152217	26040	3,06	4,36E-03
SETMAR	10762	SET domain and marine	ENSG00000170364	6419	2,03	1,78E-02
SFN	10773	stratifin [Source:HGNC	ENSG00000175793	2810	4,26	1,94E-02
SFRP1	10776	secreted frizzled-relatec	ENSG00000104332	6422	2,36	4,29E-03
SFRP2	10777	secreted frizzled-relatec	ENSG00000145423	6423	9,40	4,39E-05
SFRP4	10778	secreted frizzled-relatec	ENSG00000106483	6424	6,29	7,82E-05
SGCA	10805	sarcoglycan, alpha (50k	ENSG00000108823	6442	9,16	2,23E-08
SGCD	10807	sarcoglycan, delta (35kl	ENSG00000170624	6444	5,26	3,13E-06
SGCG	10809	sarcoglycan, gamma (3	ENSG00000102683	6445	5,27	5,60E-05
SGSM1	29410	small G protein signalin	ENSG00000167037	129049	2,56	2,85E-04
SH3BGR	10822	SH3 domain binding glu	ENSG00000185437	6450	3,67	1,92E-03
SH3BGRL	10823	SH3 domain binding glu	ENSG00000131171	6451	2,46	7,11E-04
SHISA6	34491	shisa homolog 6 (Xenop	ENSG00000188803	388336	4,16	9,02E-03
SHOX2	10854	short stature homeobox	ENSG00000168779	6474	3,29	9,44E-07
SHROOM3	30422	shroom family member	ENSG00000138771	57619	4,95	6,63E-03
SIDT1	25967	SID1 transmembrane fa	ENSG00000072858	54847	3,72	2,53E-02
SIGLEC1	11127	sialic acid binding Ig-lik	ENSG00000088827	6614	6,97	2,10E-05
SIGLECP3					3,59	1,49E-02
SLA	10902	Src-like-adaptor [Sourc	ENSG00000155926	6503	7,05	4,56E-05
SLAMF8	21391	SLAM family member 8	ENSG00000158714	56833	3,14	1,72E-02
SLC10A6	30603	solute carrier family 10	ENSG00000145283	345274	4,95	1,80E-02
SLC11A1	10907	solute carrier family 11	ENSG00000018280	6556	5,04	2,10E-03
SLC13A3	14430	solute carrier family 13	ENSG00000158296	64849	2,54	9,79E-03
SLC15A2	10921	solute carrier family 15	ENSG00000163406	6565	3,81	4,46E-03
SLC15A3	18068	solute carrier family 15,	ENSG00000110446	51296	2,36	4,28E-05
SLC16A14	26417	solute carrier family 16,	ENSG00000163053	151473	4,21	1,80E-02
SLC16A2	10923	solute carrier family 16,	ENSG00000147100	6567	2,27	1,72E-03
SLC16A8	16270	solute carrier family 16,	ENSG00000100156	23539	2,13	4,08E-02
SLC16A9	23520	solute carrier family 16,	ENSG00000165449	220963	6,58	1,05E-02
SLC17A7	16704	solute carrier family 17	ENSG00000104888	57030	4,67	1,56E-07
SLC19A3	16266	solute carrier family 19,	ENSG00000135917	80704	7,52	2,82E-03
SLC1A2	10940	solute carrier family 1 (	ENSG00000110436	6506	3,25	2,88E-02
SLC1A3	10941	solute carrier family 1 (	ENSG00000079215	6507	7,34	9,14E-03
SLC1A7	10945	solute carrier family 1 (	ENSG00000162383	6512	3,30	6,88E-04
SLC22A17	23095	solute carrier family 22,	ENSG00000092096	51310	3,81	1,23E-03

SLC22A2	10966	solute carrier family 22	ENSG00000112499	6582	5,20	3,17E-05
SLC22A3	10967	solute carrier family 22	ENSG00000146477	6581	5,18	2,94E-09
SLC24A3	10977	solute carrier family 24	ENSG00000185052	57419	8,39	8,51E-13
SLC25A12	10982	solute carrier family 25	ENSG00000115840	8604	2,09	9,05E-06
SLC25A21	14411	solute carrier family 25	ENSG00000183032	89874	2,90	3,92E-03
SLC25A25	20663	solute carrier family 25	ENSG00000148339	114789	2,64	1,19E-02
SLC25A27	21065	solute carrier family 25,	ENSG00000153291	9481	3,04	1,85E-03
SLC25A4	10990	solute carrier family 25	ENSG00000151729	291	2,02	2,62E-03
SLC25A47	20115	solute carrier family 25,	ENSG00000140107	283600	4,96	3,01E-03
SLC26A10	14470	solute carrier family 26,	ENSG00000135502	65012	6,57	6,54E-08
SLC26A4	8818	solute carrier family 26,	ENSG00000091137	5172	2,55	3,60E-02
SLC26A7	14467	solute carrier family 26,	ENSG00000147606	115111	3,98	4,78E-02
SLC27A2	10996	solute carrier family 27	ENSG00000140284	11001	4,13	3,32E-02
SLC2A4	11009	solute carrier family 2 (	ENSG00000181856	6517	6,09	1,63E-06
SLC2A5	11010	solute carrier family 2 (	ENSG00000142583	6518	4,97	3,98E-04
SLC35F1	21483	solute carrier family 35,	ENSG00000196376	222553	5,34	9,82E-06
SLC38A11	26836	solute carrier family 38,	ENSG00000169507	151258	4,96	7,65E-04
SLC38A3	18044	solute carrier family 38,	ENSG00000188338		4,77	2,75E-03
SLC39A8	20862	solute carrier family 39	ENSG00000138821	64116	2,58	3,29E-02
SLC40A1	10909	solute carrier family 40	ENSG00000138449	30061	2,42	4,79E-02
SLC6A1	11042	solute carrier family 6 (	ENSG00000157103	6529	7,55	8,05E-06
SLC6A16	13622	solute carrier family 6,	ENSG00000063127	28968	2,17	1,48E-02
SLC7A4	11062	solute carrier family 7 (	ENSG00000099960	6545	6,54	2,05E-06
SLC7A8	11066	solute carrier family 7 (	ENSG00000092068	23428	6,49	9,11E-05
SLC8A1	11068	solute carrier family 8 (	ENSG00000183023	6546	3,00	4,01E-04
SLC9A9	20653	solute carrier family 9,	ENSG00000181804	285195	4,03	8,08E-07
SLCO1C1	13819	solute carrier organic ar	ENSG00000139155	53919	2,77	1,53E-02
SLCO2B1	10962	solute carrier organic ar	ENSG00000137491	11309	3,35	5,01E-03
SLCO3A1	10952	solute carrier organic ar	ENSG00000176463	28232	2,69	4,10E-02
SLCO4A1	10953	solute carrier organic ar	ENSG00000101187	28231	3,95	1,46E-02
SLFN13	26481	schlafen family member	ENSG00000154760	146857	2,73	8,97E-04
SLIT3	11087	slit homolog 3 (Drosoph	ENSG00000184347	6586	5,04	1,46E-07
SLITRK2	13449	SLIT and NTRK-like fam	ENSG00000185985	84631	2,86	1,24E-02
SLITRK4	23502	SLIT and NTRK-like fam	ENSG00000179542	139065	7,35	3,86E-08
SLITRK6	23503	SLIT and NTRK-like fam	ENSG00000184564	84189	4,80	3,02E-02
SLMAP	16643	sarcolemma associated	ENSG00000163681	7871	3,42	2,43E-04
SLN	11089	sarcolipin [Source:HGN	ENSG00000170290	6588	5,98	2,28E-02
SLPI	11092	secretory leukocyte pep	ENSG00000124107	6590	6,81	1,34E-05
SMARCD3	11108	SWI/SNF related, matri	ENSG00000082014	6604	2,52	3,53E-03
SMOC2	20323	SPARC related modular	ENSG00000112562	64094	8,28	2,79E-22
SMTNL2	24764	smoothelin-like 2 [Sour	ENSG00000188176	342527	4,39	8,18E-04
SNCAIP	11139	synuclein, alpha interac	ENSG00000064692	9627	2,94	9,74E-04
SNCG	11141	synuclein, gamma (brea	ENSG00000173267	6623	2,08	1,94E-02
SNORA23	32613	small nucleolar RNA, H/	ENSG00000201998	677808	3,35	3,34E-02
SNORA33	32623	small nucleolar RNA, H/	ENSG00000200534	594839	2,31	4,98E-03
SNORA41	32634	small nucleolar RNA, H/	ENSG00000207406	619569	2,04	2,53E-02
SNORA44					2,82	9,88E-03
SNORA48	32641	small nucleolar RNA, H/	ENSG00000209582	652965	3,56	9,92E-04
SNORA53	32646	small nucleolar RNA, H/	ENSG00000212443	677832	3,57	9,45E-03
SNORA64	10221	small nucleolar RNA, H/	ENSG00000207405	26784	2,40	2,47E-02
SNORA67	10224	small nucleolar RNA, H/	ENSG00000264772	26781	2,31	3,46E-03
SNORD10	32706	small nucleolar RNA, C/	ENSG00000238917		2,85	4,27E-03
SNORD100	32763	small nucleolar RNA, C/	ENSG00000221500	594838	2,96	6,02E-03

SNORD15B	16649	small nucleolar RNA, C/	ENSG00000207445	114599	4,58	1,61E-03
SNORD17	32713	small nucleolar RNA, C/	ENSG00000212232	692086	4,19	6,87E-03
SNORD32A	10159	small nucleolar RNA, C/	ENSG00000201675	26819	2,20	2,56E-02
SNORD34	10161	small nucleolar RNA, C/	ENSG00000202503	26817	2,38	3,44E-02
SNORD81					2,08	3,94E-02
SNORD97	32760	small nucleolar RNA, C/	ENSG00000238622	692223	2,86	2,04E-02
SNTA1	11167	syntrophin, alpha 1 [So	ENSG00000101400	6640	2,28	5,39E-03
SNTB1	11168	syntrophin, beta 1 (dys	ENSG00000172164	6641	3,31	2,16E-02
SNTG2	13741	syntrophin, gamma 2 [S	ENSG00000172554	54221	6,78	2,31E-09
SNX2	11173	sorting nexin 2 [Source	ENSG00000205302	6643	2,31	1,47E-05
SOBP	29256	sine oculis binding prot	ENSG00000112320	55084	2,86	3,69E-04
SOCS3	19391	suppressor of cytokine	ENSG00000184557	9021	4,09	1,25E-03
SOD3	11181	superoxide dismutase 3	ENSG00000109610	6649	9,00	6,81E-12
SORBS1	14565	sorbin and SH3 domain	ENSG00000095637	10580	7,79	1,31E-10
SORCS1	16697	sortilin-related VPS10 d	ENSG00000108018	114815	4,87	2,04E-04
SORL1	11185	sortilin-related receptor	ENSG00000137642	6653	6,19	5,92E-04
SOX10	11190	SRY (sex determining r	ENSG00000100146	6663	3,58	8,59E-03
SOX15	11196	SRY (sex determining r	ENSG00000129194	6665	3,68	5,14E-04
SOX5	11201	SRY (sex determining r	ENSG00000134532	6660	3,59	1,53E-04
SOX9	11204	SRY (sex determining r	ENSG00000125398	6662	3,96	1,15E-02
SP5	14529	Sp5 transcription factor	ENSG00000204335	389058	4,28	8,02E-03
SPAG8	14105	sperm associated antig	ENSG00000137098	26206	3,19	1,95E-03
SPARCL1	11220	SPARC-like 1 (hevin) [S	ENSG00000152583	8404	7,64	3,95E-12
SPATA6	18309	spermatogenesis associ	ENSG00000132122	54558	2,77	6,88E-04
SPECC1	30615	sperm antigen with calp	ENSG00000128487	92521	3,70	4,61E-07
SPEF2	26293	sperm flagellar 2 [Sour	ENSG00000152582	79925	3,39	1,25E-04
SPEG	16901	SPEG complex locus [Sc	ENSG00000072195	10290	5,42	1,30E-05
SPI1	11241	spleen focus forming vi	ENSG00000066336	6688	6,96	1,11E-08
SPINK5	15464	serine peptidase inhibit	ENSG00000133710	11005	3,84	2,65E-02
SPOCK2	13564	sparc/osteonectin, cwcv	ENSG00000107742	9806	2,57	3,62E-03
SPON1	11252	spondin 1, extracellular	ENSG00000262655	10418	4,40	5,92E-04
SPTB	11274	spectrin, beta, erythroc	ENSG00000070182	6710	6,22	5,75E-06
SPTBN4	14896	spectrin, beta, non-eryt	ENSG00000160460	57731	3,94	6,86E-03
SPTLC3	16253	serine palmitoyltransfer	ENSG00000172296	55304	2,64	5,04E-03
SRGAP3	19744	SLIT-ROBO Rho GTPase	ENSG00000196220	9901	3,05	1,76E-03
SRL	11295	sarcalumenin [Source:H	ENSG00000185739	6345	3,54	1,94E-04
SRSF12	21220	serine/arginine-rich spli	ENSG00000154548	135295	3,06	2,61E-02
SSC5D	26641	scavenger receptor cyst	ENSG00000179954	284297	2,57	2,96E-03
SSPN	11322	sarcospan [Source:HGN	ENSG00000123096	8082	4,85	1,54E-12
SSPO	21998	SCO-spondin homolog (	ENSG00000197558		2,66	7,37E-04
SSTR2	11331	somatostatin receptor 2	ENSG00000180616	6752	5,49	4,83E-03
ST14	11344	suppression of tumorig	ENSG00000149418	6768	3,36	2,88E-02
ST5	11350	suppression of tumorig	ENSG00000166444	6764	2,98	1,46E-05
ST6GAL2	10861	ST6 beta-galactosamide	ENSG00000144057	84620	6,09	8,91E-06
ST6GALNAC1	23614	ST6 (alpha-N-acetyl-ne	ENSG00000070526	55808	5,01	1,05E-03
ST6GALNAC5	19342	ST6 (alpha-N-acetyl-ne	ENSG00000117069	81849	4,77	3,18E-04
ST7-AS1	16000	ST7 antisense RNA 1 [S	ENSG00000227199	93653	3,04	4,10E-04
ST8SIA1	10869	ST8 alpha-N-acetyl-neu	ENSG00000111728	6489	4,52	3,64E-05
STAB2	18629	stabilin 2 [Source:HGN(	ENSG00000136011	55576	6,18	3,92E-02
STAC	11353	SH3 and cysteine rich d	ENSG00000144681	6769	3,03	2,27E-02
STAMBPL1	24105	STAM binding protein-lil	ENSG00000138134	57559	4,27	3,03E-06
STARD5	18065	StAR-related lipid trans	ENSG00000172345	80765	2,44	4,81E-03
STAT4	11365	signal transducer and a	ENSG00000138378	6775	2,91	1,35E-02

STBD1					3,43	3,81E-03
STEAP2	17885	STEAP family member 2	ENSG00000157214	261729	3,04	1,03E-04
STEAP4	21923	STEAP family member 4	ENSG00000127954	79689	8,16	8,22E-18
STK31	11407	serine/threonine kinase	ENSG00000196335	56164	2,66	1,18E-02
STK32A	28317	serine/threonine kinase	ENSG00000169302	202374	3,28	2,06E-02
STOX1	23508	storkhead box 1 [Sourc	ENSG00000165730	219736	5,07	6,87E-04
STOX2	25450	storkhead box 2 [Sourc	ENSG00000173320	56977	3,29	9,62E-05
STXBP5L	30757	syntaxin binding protein	ENSG00000145087	9515	4,35	2,46E-02
STXBP6	19666	syntaxin binding protein	ENSG00000168952	29091	6,54	2,39E-07
SUSD4	25470	sushi domain containing	ENSG00000143502	55061	3,49	3,34E-02
SV2B	16874	synaptic vesicle glycopr	ENSG00000185518	9899	2,94	6,86E-03
SVEP1	15985	sushi, von Willebrand fa	ENSG00000165124	79987	6,16	2,81E-04
SYCP2	11490	synaptonemal complex	ENSG00000196074	10388	2,36	2,38E-02
SYCP2L	21537	synaptonemal complex	ENSG00000153157	221711	4,48	3,74E-04
SYK	11491	spleen tyrosine kinase [	ENSG00000165025	6850	6,82	1,61E-08
SYN2	11495	synapsin II [Source:HG	ENSG00000157152		2,37	4,77E-02
SYNDIG1	15885	synapse differentiation	ENSG00000101463	79953	7,54	2,04E-06
SYNM	24466	synemin, intermediate f	ENSG00000182253	23336	4,87	4,87E-05
SYNPO2	17732	synaptopodin 2 [Source	ENSG00000172403	171024	9,01	3,73E-12
SYNPO2L	23532	synaptopodin 2-like [So	ENSG00000166317	79933	4,49	2,47E-03
SYP	11506	synaptophysin [Source:	ENSG00000102003	6855	3,35	1,51E-04
SYPL2	27638	synaptophysin-like 2 [S	ENSG00000143028	284612	4,24	4,71E-05
SYT2	11510	synaptotagmin II [Sour	ENSG00000143858	127833	5,77	7,11E-04
SYT7	11514	synaptotagmin VII [Sou	ENSG00000011347	9066	2,92	8,94E-03
SYTL2	15585	synaptotagmin-like 2 [S	ENSG00000137501	54843	6,64	1,20E-04
TAC3	11521	tachykinin 3 [Source:H(	ENSG00000166863	6866	5,25	1,82E-03
TACR1	11526	tachykinin receptor 1 [S	ENSG00000115353	6869	4,77	7,01E-04
TACR2	11527	tachykinin receptor 2 [S	ENSG00000075073	6865	4,58	4,83E-04
TAGAP	15669	T-cell activation RhoGTI	ENSG00000164691	117289	4,68	7,90E-04
TBC1D10C	24702	TBC1 domain family, m	ENSG00000175463	374403	2,99	2,15E-02
TBX15	11594	T-box 15 [Source:HGNC	ENSG00000092607	6913	4,82	7,01E-08
TBXA2R	11608	thromboxane A2 recept	ENSG00000006638	6915	2,23	2,04E-02
TBXAS1	11609	thromboxane A synthas	ENSG00000059377	6916	2,57	1,02E-02
TC2N	19859	tandem C2 domains, nu	ENSG00000165929	123036	2,67	5,58E-05
TCAP	11610	titin-cap [Source:HGNC	ENSG00000173991	8557	2,25	3,92E-02
TCEA3	11615	transcription elongation	ENSG00000204219	6920	4,05	3,28E-06
TCEAL1	11616	transcription elongation	ENSG00000172465	9338	3,16	3,06E-03
TCEAL2	29818	transcription elongation	ENSG00000184905	140597	6,56	1,07E-04
TCEAL5	22282	transcription elongation	ENSG00000204065	340543	3,26	4,25E-02
TCEAL7	28336	transcription elongation	ENSG00000182916	56849	2,99	3,46E-04
TCF21	11632	transcription factor 21 [	ENSG00000118526	6943	4,44	1,90E-04
TCHH	11791	trichohyalin [Source:HG	ENSG00000159450	7062	3,94	7,19E-03
TCTEX1D4	32315	Tctex1 domain containin	ENSG00000188396	343521	3,22	9,28E-03
TDRD6	21339	tudor domain containing	ENSG00000180113	221400	2,84	2,22E-02
TEAD3	11716	TEA domain family men	ENSG00000007866	7005	2,89	6,66E-03
TEKT2	11725	tektin 2 (testicular) [So	ENSG00000092850	27285	4,22	1,17E-04
TEKT3	14293	tektin 3 [Source:HGNC	ENSG00000125409	64518	4,54	5,53E-03
TENC1	19737	tensin like C1 domain c	ENSG00000111077	23371	2,54	7,21E-05
TET1	29484	tet methylcytosine diox	ENSG00000138336	80312	2,56	6,33E-03
TF	11740	transferrin [Source:HGN	ENSG00000091513	7018	4,20	2,35E-02
TGFB3	11769	transforming growth fac	ENSG00000119699	7043	4,19	5,73E-05
TGFBR3	11774	transforming growth fac	ENSG00000069702	7049	2,81	1,05E-03
THBD	11784	thrombomodulin [Sourc	ENSG00000178726	7056	3,90	1,74E-02

THBS4	11788	thrombospondin 4 [Sou	ENSG00000113296	7060	8,79	5,95E-09
THNSL2	25602	threonine synthase-like	ENSG00000144115	55258	5,54	5,50E-08
THRB	11799	thyroid hormone recept	ENSG00000151090	7068	4,80	6,42E-17
THRSP	11800	thyroid hormone respor	ENSG00000151365	7069	6,78	8,18E-03
THSD7B	29348	thrombospondin, type I	ENSG00000144229	80731	4,45	2,79E-03
THY1	11801	Thy-1 cell surface antig	ENSG00000154096	7070	4,83	4,29E-03
TIAM1	11805	T-cell lymphoma invasic	ENSG00000156299	7074	3,21	4,00E-02
TIMP3	11822	TIMP metallopeptidase i	ENSG00000100234	7078	2,65	2,98E-04
TIMP4	11823	TIMP metallopeptidase i	ENSG00000157150	7079	3,99	1,41E-02
TLN2	15447	talin 2 [Source:HGNC S	ENSG00000171914	83660	2,38	4,06E-03
TLR5	11851	toll-like receptor 5 [Sou	ENSG00000187554	7100	3,76	6,18E-05
TLR7	15631	toll-like receptor 7 [Sou	ENSG00000196664	51284	4,85	7,75E-04
TLR8	15632	toll-like receptor 8 [Sou	ENSG00000101916	51311	4,92	1,02E-03
TMC5	22999	transmembrane channe	ENSG00000103534	79838	4,89	5,34E-04
TMEM119	27884	transmembrane protein	ENSG00000183160	338773	9,53	3,17E-14
TMEM130	25429	transmembrane protein	ENSG00000261149	222865	5,39	2,91E-05
TMEM132C	25436	transmembrane protein	ENSG00000181234	92293	7,93	4,99E-05
TMEM133	24033	transmembrane protein	ENSG00000170647	83935	2,35	2,60E-04
TMEM176A	24930	transmembrane protein	ENSG00000002933	55365	8,85	3,28E-16
TMEM176B	29596	transmembrane protein	ENSG00000106565	28959	10,14	7,47E-17
TMEM198	33704	transmembrane protein	ENSG00000188760	130612	2,52	2,04E-02
TMEM229B	20130	transmembrane protein	ENSG00000198133	161145	2,66	4,92E-02
TMEM30B	27254	transmembrane protein	ENSG00000182107	161291	4,88	8,54E-15
TMEM35	25864	transmembrane protein	ENSG00000126950	59353	3,04	8,37E-03
TMEM56	26477	transmembrane protein	ENSG00000152078	148534	4,95	1,78E-06
TMEM61	27296	transmembrane protein	ENSG00000143001	199964	5,19	3,81E-03
TMEM71	26572	transmembrane protein	ENSG00000165071	137835	4,27	1,18E-03
TMEM74	26409	transmembrane protein	ENSG00000164841	157753	3,85	3,94E-04
TMEM74B	15893	transmembrane protein	ENSG00000125895	55321	3,55	1,14E-03
TMOD1	11871	tropomodulin 1 [Source	ENSG00000136842	7111	4,32	5,75E-08
TNC	5318	tenascin C [Source:HGN	ENSG00000041982	3371	3,21	1,69E-02
TNFRSF8	11923	tumor necrosis factor re	ENSG00000120949	943	4,04	6,66E-03
TNFSF13B	11929	tumor necrosis factor (l	ENSG00000102524	10673	4,87	5,21E-05
TNFSF14	11930	tumor necrosis factor (l	ENSG00000125735	8740	6,04	4,45E-03
TNFSF8	11938	tumor necrosis factor (l	ENSG00000106952	944	5,31	1,21E-05
TNMD	17757	tenomodulin [Source:H	ENSG00000000005	64102	6,71	2,21E-07
TNNC1	11943	troponin C type 1 (slow	ENSG00000114854	7134	4,78	3,33E-04
TNNC2	11944	troponin C type 2 (fast)	ENSG00000101470	7125	3,59	2,55E-03
TNNT3	11950	troponin T type 3 (skele	ENSG00000130595	7140	8,92	4,36E-08
TNS1	11973	tensin 1 [Source:HGNC	ENSG00000079308	7145	3,64	1,24E-04
TNS4	24352	tensin 4 [Source:HGNC	ENSG00000131746	84951	5,30	3,58E-03
TNXB	11976	tenascin XB [Source:HG	ENSG00000236236	7148	6,90	5,96E-07
TOB1	11979	transducer of ERBB2, 1	ENSG00000141232	10140	3,09	5,02E-04
TPD52L1	12006	tumor protein D52-like	ENSG00000111907	7164	3,29	2,07E-05
TPM2	12011	tropomyosin 2 (beta) [S	ENSG00000198467	7169	2,49	1,45E-02
TPO	12015	thyroid peroxidase [Sou	ENSG00000115705	7173	7,50	1,69E-04
TPPP	24164	tubulin polymerization p	ENSG00000171368	11076	3,84	4,78E-03
TPPP3	24162	tubulin polymerization-p	ENSG00000159713	51673	6,68	3,06E-13
TPRG1	24759	tumor protein p63 regu	ENSG00000188001	285386	4,59	2,55E-03
TPSAB1	12019	tryptase alpha/beta 1 [S	ENSG00000172236	7177	6,99	3,61E-04
TPSB2	14120	tryptase beta 2 (gene/p	ENSG00000197253	64499	6,78	2,30E-04
TRDN	12261	triadin [Source:HGNC S	ENSG00000186439	10345	8,25	6,25E-08
TREH	12266	trehalase (brush-border	ENSG00000118094		4,72	5,73E-03

TREM1	17760	triggering receptor expr	ENSG00000124731	54210	5,46	1,34E-03
TRERF1	18273	transcriptional regulatin	ENSG00000124496	55809	2,54	1,12E-05
TRHDE	30748	thyrotropin-releasing hc	ENSG00000072657	29953	4,52	1,59E-03
TRIB1	16891	tribbles homolog 1 (Dro	ENSG00000173334	10221	3,92	6,48E-03
TRPC4	12336	transient receptor poter	ENSG00000133107	7223	2,97	7,93E-03
TRPM3	17992	transient receptor poter	ENSG00000083067	80036	3,38	2,84E-02
TRPM5	14323	transient receptor poter	ENSG00000070985	29850	5,84	7,93E-03
TSC22D3	3051	TSC22 domain family, n	ENSG00000157514	1831	3,13	5,86E-03
TSHZ2	13010	teashirt zinc finger hom	ENSG00000182463	128553	3,61	4,51E-04
TSIX	12377	TSIX transcript, XIST ar	ENSG00000270641	9383	4,37	7,94E-03
TSKS	30719	testis-specific serine kir	ENSG00000126467	60385	4,49	1,37E-03
TSPAN2	20659	tetraspanin 2 [Source:H	ENSG00000134198	10100	4,52	4,68E-03
TSPAN33	28743	tetraspanin 33 [Source:	ENSG00000158457	340348	4,80	3,45E-04
TSPAN8	11855	tetraspanin 8 [Source:H	ENSG00000127324	7103	9,74	4,34E-08
TSPYL2	24358	TSPY-like 2 [Source:HG	ENSG00000184205	64061	2,06	3,26E-03
TTC9	20267	tetratricopeptide repeat	ENSG00000133985	23508	4,38	1,34E-03
TTLL7	26242	tubulin tyrosine ligase-l	ENSG00000137941	79739	2,90	7,43E-03
TTN	12403	titin [Source:HGNC Syn	ENSG00000155657	7273	2,56	1,43E-04
TTYH2	13877	tweety homolog 2 (Dros	ENSG00000141540	94015	2,09	4,85E-03
TUB	12406	tubby homolog (mouse)	ENSG00000166402	7275	2,08	9,50E-03
TUBAL3	23534	tubulin, alpha-like 3 [Sc	ENSG00000263440	79861	7,00	2,56E-08
TUBD1	16811	tubulin, delta 1 [Source	ENSG00000108423	51174	2,09	1,84E-03
TULP2	12424	tubby like protein 2 [So	ENSG00000104804	7288	4,15	1,36E-02
TUSC5	29592	tumor suppressor candi	ENSG00000184811	286753	6,99	8,29E-03
TXNDC16	19965	thioredoxin domain con	ENSG00000087301	57544	2,32	1,01E-04
TXNIP	16952	thioredoxin interacting		ENSG00000117289	10628	2,92	3,75E-03
TYROBP	12449	TYRO protein tyrosine k	ENSG00000011600	7305	8,97	3,84E-16
TYW3	24757	tRNA-yW synthesizing p	ENSG00000162623	127253	2,24	5,48E-08
UBE2QL1	37269	ubiquitin-conjugating er	ENSG00000215218	134111	5,16	2,09E-04
UBXN10	26354	UBX domain protein 10	ENSG00000162543	127733	3,91	8,48E-03
UCP2	12518	uncoupling protein 2 (m	ENSG00000175567	7351	3,00	5,92E-04
UCP3	12519	uncoupling protein 3 (m	ENSG00000175564	7352	2,05	1,26E-02
UFSP2	25640	UFM1-specific peptidase	ENSG00000109775	55325	2,02	4,61E-03
UNC13C	23149	unc-13 homolog C (C. e	ENSG00000137766	440279	4,88	7,89E-04
UNC13D	23147	unc-13 homolog D (C. e	ENSG00000092929	201294	2,54	3,04E-02
UNC5C	12569	unc-5 homolog C (C. ele	ENSG00000182168	8633	5,49	6,60E-05
UNC5CL	21203	unc-5 homolog C (C. ele	ENSG00000124602	222643	2,67	2,16E-03
USH1C	12597	Usher syndrome 1C (au	ENSG00000006611	10083	6,13	2,80E-04
USP16	12614	ubiquitin specific peptid	ENSG00000156256	10600	2,61	1,81E-07
USP2	12618	ubiquitin specific peptid	ENSG00000036672	9099	3,80	3,61E-04
USP53	29255	ubiquitin specific peptid	ENSG00000145390	54532	2,37	1,98E-04
VANGL2	15511	VANGL planar cell polar	ENSG00000162738	57216	5,75	8,05E-08
VASH2	25723	vasohibin 2 [Source:HG	ENSG00000143494	79805	2,16	1,88E-02
VAV1	12657	vav 1 guanine nucleotid	ENSG00000141968	7409	5,35	5,67E-05
VENTX	13639	VENT homeobox [Sourc	ENSG00000151650	27287	4,56	2,32E-03
VIPR1	12694	vasoactive intestinal pe	ENSG00000114812	7433	6,28	8,37E-04
VIPR2	12695	vasoactive intestinal pe	ENSG00000106018	7434	6,11	2,23E-08
VIT	12697	vitrin [Source:HGNC Sy	ENSG00000205221	5212	7,14	2,35E-10
VMO1	30387	vitelline membrane oute	ENSG00000182853	284013	4,35	1,53E-03
VNN1	12705	vanin 1 [Source:HGNC S	ENSG00000112299	8876	5,15	1,70E-03
VNN2	12706	vanin 2 [Source:HGNC S	ENSG00000112303	8875	6,34	2,74E-03
VSIG4	17032	V-set and immunoglobu	ENSG00000155659	11326	8,60	3,04E-08
VSTM4	26470	V-set and transmembra	ENSG00000165633	196740	5,47	4,26E-05

VTN	12724	vitronectin [Source:HGI	ENSG00000262062	7448	2,56	2,26E-02
VTRNA2-1	37054	vault RNA 2-1 [Source:I	ENSG00000270123	100126299	3,68	2,29E-02
VWC2	30200	von Willebrand factor C	ENSG00000188730	375567	4,09	6,01E-03
WAS	12731	Wiskott-Aldrich syndron	ENSG00000015285	7454	3,82	2,02E-03
WBSCR17	16347	Williams-Beuren syndro	ENSG00000185274	64409	7,04	1,15E-06
WDFY4	29323	WDFY family member 4	ENSG00000128815	57705	2,07	2,81E-02
WDR17	16661	WD repeat domain 17 [	ENSG00000150627	116966	4,40	8,76E-05
WDR49	26587	WD repeat domain 49 [	ENSG00000174776	151790	4,25	1,36E-03
WDR96	26684	WD repeat domain 96 [	ENSG00000197748	80217	3,58	9,15E-03
WFDC1	15466	WAP four-disulfide core	ENSG00000103175	58189	8,76	1,99E-08
WFDC2	15939	WAP four-disulfide core	ENSG00000101443	10406	8,20	1,08E-04
WIPF3	22004	WAS/WASL interacting		ENSG00000122574	644150	3,91	6,15E-04
WISP1	12769	WNT1 inducible signalin	ENSG00000104415	8840	5,57	2,52E-06
WISP2	12770	WNT1 inducible signalin	ENSG00000064205	8839	9,02	2,41E-10
WNK2	14542	WNK lysine deficient pr	ENSG00000165238	65268	7,12	2,27E-05
WNT11	12776	wingless-type MMTV int	ENSG00000085741	7481	8,32	3,13E-08
WNT2	12780	wingless-type MMTV int	ENSG00000105989	7472	6,00	8,30E-04
WNT4	12783	wingless-type MMTV int	ENSG00000162552	54361	3,76	3,75E-03
WSCD2	29117	WSC domain containing	ENSG00000075035	9671	6,04	1,74E-04
XG	12806	Xg blood group [Source	ENSG00000124343	7499	7,20	1,55E-09
XIRP1	14301	xin actin-binding repeat	ENSG00000168334	165904	4,09	2,58E-02
XIST	12810	X inactive specific trans	ENSG00000229807	7503	9,50	2,11E-09
XK	12811	X-linked Kx blood group	ENSG00000047597	7504	3,50	2,65E-03
XPNPEP2	12823	X-prolyl aminopeptidase	ENSG00000122121	7512	4,18	7,17E-04
YBX2	17948	Y box binding protein 2	ENSG00000006047	51087	2,86	2,91E-02
YJEFN3	24785	YjeF N-terminal domain	ENSG00000250067	374887	2,44	1,86E-02
ZAK					2,10	8,41E-04
ZBTB16	12930	zinc finger and BTB don	ENSG00000109906	7704	2,70	2,72E-02
ZBTB7C	31700	zinc finger and BTB don	ENSG00000184828	201501	6,76	6,47E-08
ZC3H12B	17407	zinc finger CCCH-type c	ENSG00000102053	340554	4,01	6,53E-05
ZCCHC5	22997	zinc finger, CCHC domai	ENSG00000179300	203430	3,45	4,09E-02
ZDHHC15	20342	zinc finger, DHHC-type (	ENSG00000102383	158866	6,26	1,53E-12
ZDHHC8P1	26461	zinc finger, DHHC-type (	ENSG00000133519	150244	3,40	3,01E-02
ZFP2	26138	ZFP2 zinc finger protein	ENSG00000198939	80108	3,59	7,54E-04
ZFP36	12862	ZFP36 ring finger protei	ENSG00000128016	7538	6,42	1,69E-08
ZFP37	12863	ZFP37 zinc finger protei	ENSG00000136866	7539	4,34	1,50E-05
ZIC5	20322	Zic family member 5 [S	ENSG00000139800	85416	4,57	4,66E-02
ZMYND15	20997	zinc finger, MYND-type (	ENSG00000141497	84225	2,47	1,68E-02
ZNF10	12879	zinc finger protein 10 [S	ENSG00000256223	7556	2,72	5,88E-05
ZNF165	12953	zinc finger protein 165		ENSG00000197279	7718	2,54	4,46E-03
ZNF17	12958	zinc finger protein 17 [S	ENSG00000186272	7565	2,11	1,49E-04
ZNF204P	12995	zinc finger protein 204,	ENSG00000204789	7754	6,13	1,13E-12
ZNF208	12999	zinc finger protein 208		ENSG00000160321	7757	6,20	1,40E-05
ZNF214	13006	zinc finger protein 214		ENSG00000149050	7761	3,45	3,39E-02
ZNF25	13043	zinc finger protein 25 [S	ENSG00000175395	219749	2,16	2,74E-06
ZNF257	13498	zinc finger protein 257		ENSG00000197134	113835	2,85	1,35E-02
ZNF26	13053	zinc finger protein 26 [S	ENSG00000198393	7574	2,01	4,13E-06
ZNF331	15489	zinc finger protein 331		ENSG00000130844	55422	2,48	3,42E-02
ZNF334	15806	zinc finger protein 334		ENSG00000198185	55713	3,54	1,06E-03
ZNF34	13098	zinc finger protein 34 [S	ENSG00000196378	80778	2,01	2,52E-03
ZNF385B	26332	zinc finger protein 385B	ENSG00000144331	151126	4,31	1,60E-03
ZNF385C	33722	zinc finger protein 385C	ENSG00000260320	201181	3,05	1,13E-02
ZNF396	18824	zinc finger protein 396		ENSG00000186496	252884	3,46	3,44E-03

ZNF415	20636	zinc finger protein 415 |	ENSG00000170954	55786	3,75	7,54E-03
ZNF420	20649	zinc finger protein 420 |	ENSG00000197050	147923	2,27	2,92E-03
ZNF439	20873	zinc finger protein 439 |	ENSG00000171291	90594	3,31	2,35E-02
ZNF44	13110	zinc finger protein 44 [S	ENSG00000197857	51710	2,04	3,94E-04
ZNF441	20875	zinc finger protein 441 |	ENSG00000197044	126068	2,65	2,19E-02
ZNF442	20877	zinc finger protein 442 |	ENSG00000198342	79973	3,44	1,34E-04
ZNF483	23384	zinc finger protein 483 |	ENSG00000173258	158399	3,21	2,14E-03
ZNF487P					2,56	2,12E-04
ZNF503	23589	zinc finger protein 503 |	ENSG00000165655	84858	2,36	1,82E-03
ZNF519	30574	zinc finger protein 519 |	ENSG00000175322	162655	2,53	9,31E-03
ZNF536	29025	zinc finger protein 536 |	ENSG00000198597	9745	4,60	1,17E-04
ZNF540	25331	zinc finger protein 540 |	ENSG00000171817	163255	2,18	3,34E-02
ZNF559	28197	zinc finger protein 559 |	ENSG00000188321	84527	3,31	2,04E-07
ZNF563	30498	zinc finger protein 563 |	ENSG00000188868	147837	2,53	3,19E-02
ZNF571	25000	zinc finger protein 571 |	ENSG00000180479	51276	2,32	2,73E-04
ZNF577	28673	zinc finger protein 577 |	ENSG00000161551	84765	2,05	3,63E-04
ZNF595	27196	zinc finger protein 595 |	ENSG00000197701		2,59	7,83E-05
ZNF655	30899	zinc finger protein 655 |	ENSG00000197343	79027	2,03	1,23E-04
ZNF660	26720	zinc finger protein 660 |	ENSG00000144792	285349	2,68	1,40E-03
ZNF662	31930	zinc finger protein 662 |	ENSG00000182983	389114	2,14	1,71E-02
ZNF676	20429	zinc finger protein 676 |	ENSG00000262942	163223	4,42	2,92E-04
ZNF684	28418	zinc finger protein 684 |	ENSG00000117010	127396	2,13	1,96E-02
ZNF700	25292	zinc finger protein 700 |	ENSG00000196757	90592	2,13	2,44E-04
ZNF721	29425	zinc finger protein 721 |	ENSG00000182903	170960	2,05	1,39E-02
ZNF727	22785	zinc finger protein 727 |	ENSG00000257482	442319	4,50	1,16E-04
ZNF781	26745	zinc finger protein 781 |	ENSG00000196381	163115	2,74	2,25E-04
ZNF799	28071	zinc finger protein 799 |	ENSG00000196466	90576	2,77	7,94E-05
ZNF812	33242	zinc finger protein 812 |	ENSG00000224689	729648	4,91	6,34E-03
ZNF823	30936	zinc finger protein 823 |	ENSG00000197933	55552	2,48	3,81E-03
ZNF833P	33819	zinc finger protein 833,	ENSG00000197332	401898	3,76	4,81E-03
ZNF853	21767	zinc finger protein 853 |	ENSG00000236609	54753	2,14	1,37E-03
ZSCAN4	23709	zinc finger and SCAN dc	ENSG00000180532	201516	5,45	5,29E-05
